# Supplementary figures and images for: RBM7 deficiency promotes breast cancer metastasis by coordinating MFGE8 splicing switch and NF-kB pathway (part 1 of 2)
Source: eLife. 2024 Jul 12;13:RP95318. doi: 10.7554/eLife.95318 (PMC11245308; doi:10.7554/eLife.95318)

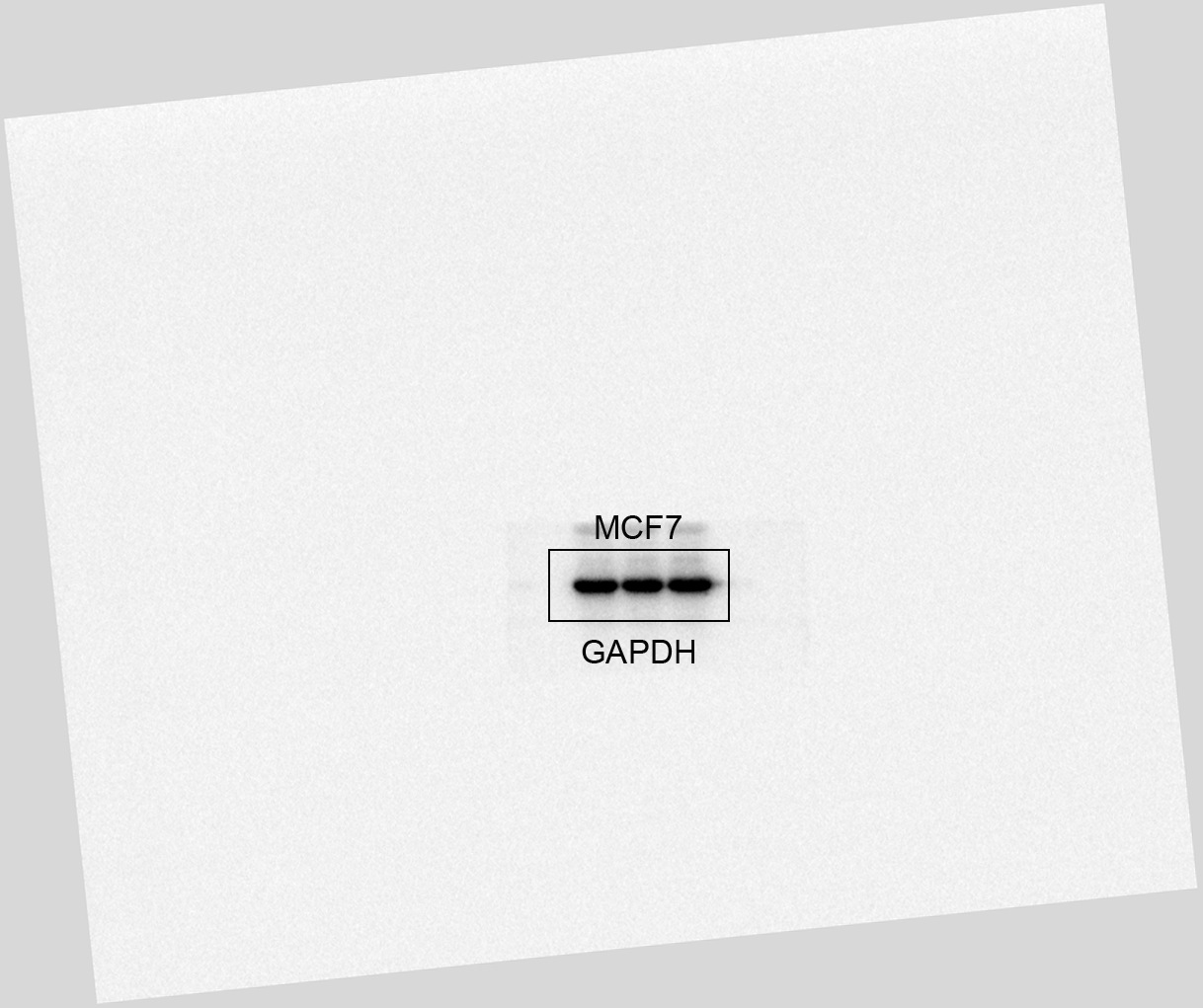

Supplement: Figure 2—figure supplement 1—source data 1. [file elife-95318-fig2-figsupp1-data1.zip › Figure 2-figure supplement 1-Source data 1/MCF7 GAPDH.tif]

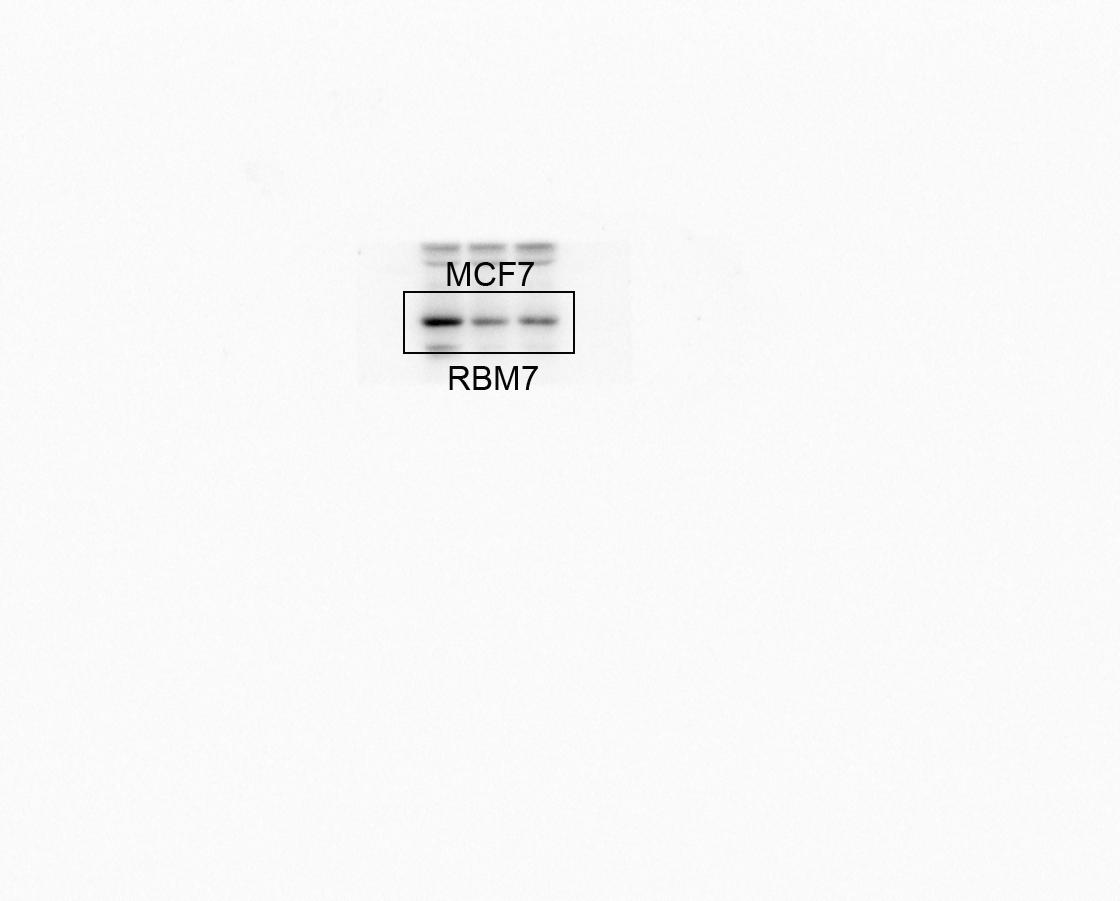

Supplement: Figure 2—figure supplement 1—source data 1. [file elife-95318-fig2-figsupp1-data1.zip › Figure 2-figure supplement 1-Source data 1/MCF7 RBM7.tif]

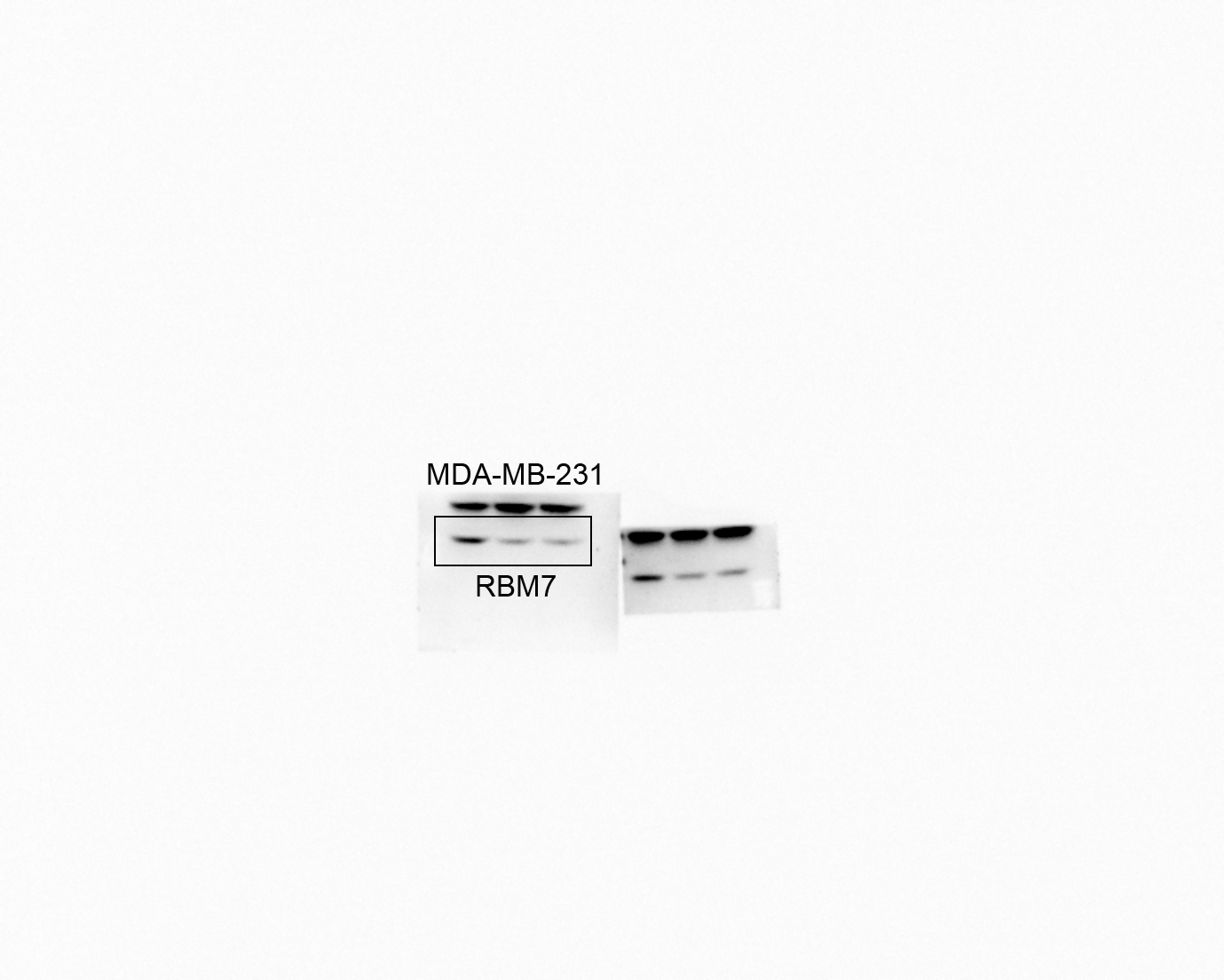

Supplement: Figure 2—figure supplement 1—source data 1. [file elife-95318-fig2-figsupp1-data1.zip › Figure 2-figure supplement 1-Source data 1/MDA-MB-231 RBM7.tif]

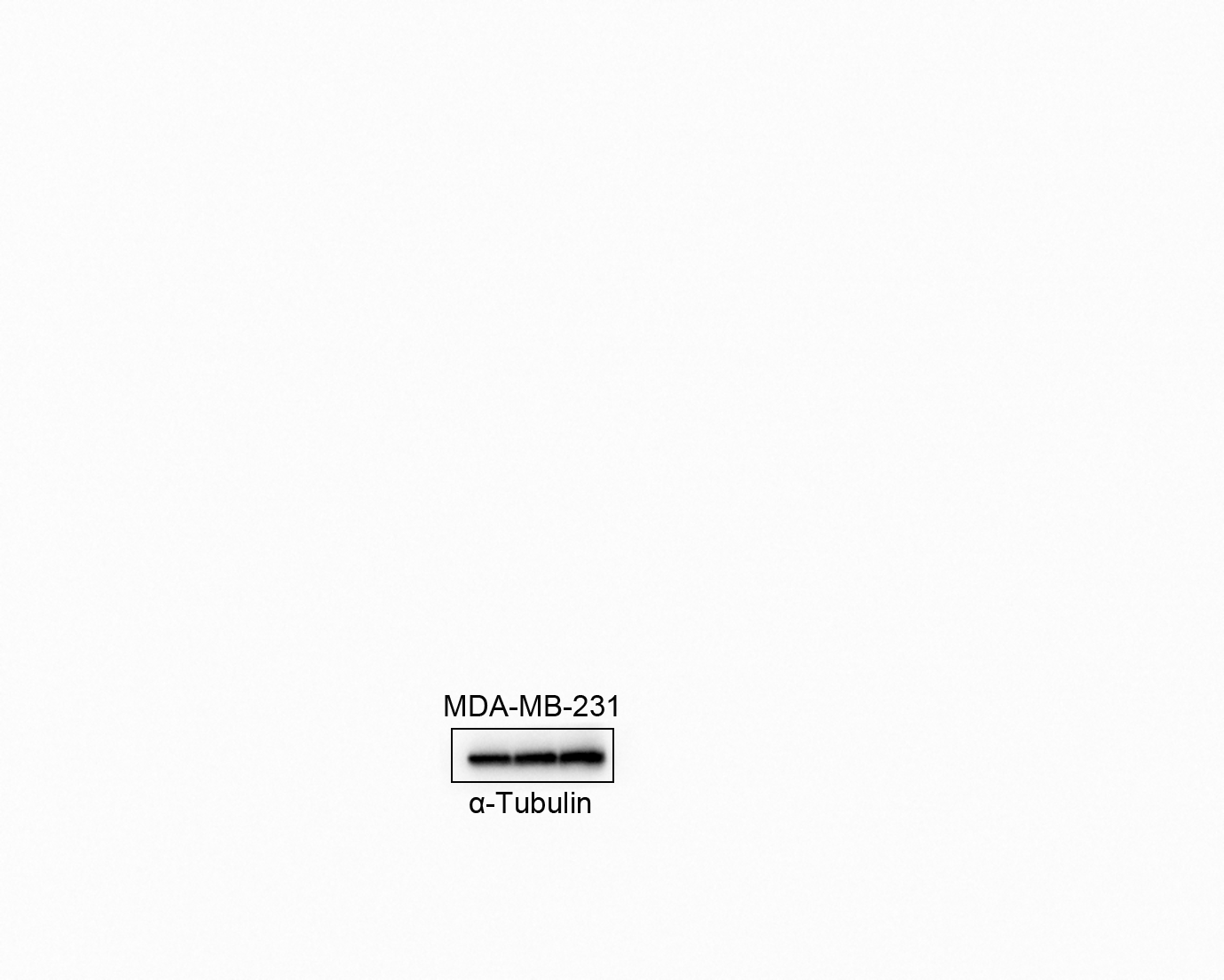

Supplement: Figure 2—figure supplement 1—source data 1. [file elife-95318-fig2-figsupp1-data1.zip › Figure 2-figure supplement 1-Source data 1/MDA-MB-231 α-Tubulin.tif]

Sup Figure2D

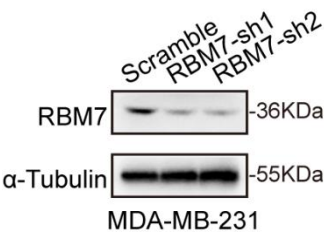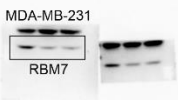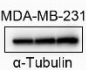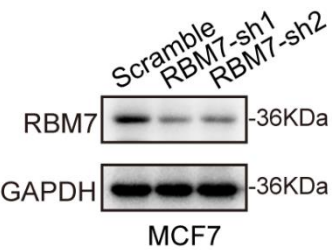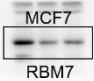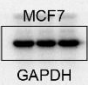

Supplement: Figure 2—figure supplement 1—source data 2. [file elife-95318-fig2-figsupp1-data2.zip › Figure 2-figure supplement 1-Source data 2/Sup Figure2D.pdf]

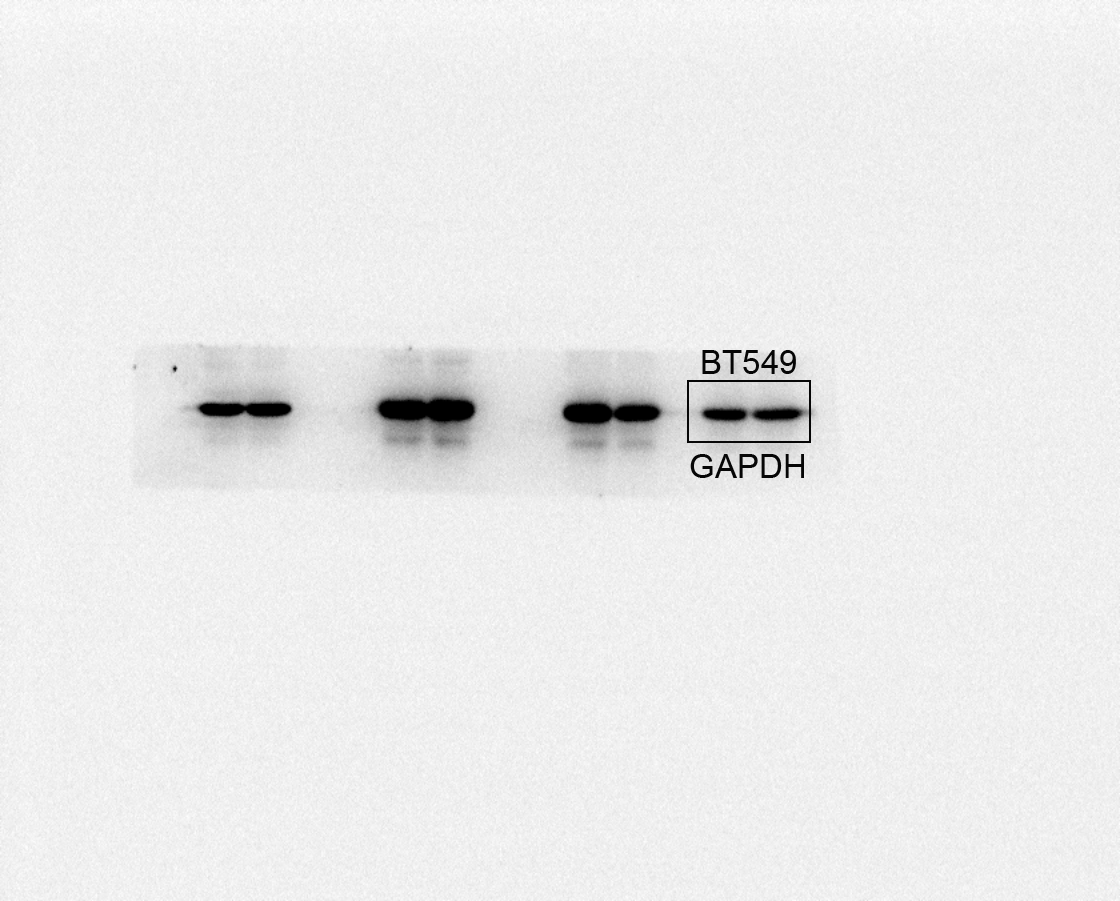

Supplement: Figure 2—figure supplement 1—source data 3. [file elife-95318-fig2-figsupp1-data3.zip › Figure 2-figure supplement 1-Source data 3/BT549-GAPDH.tif]

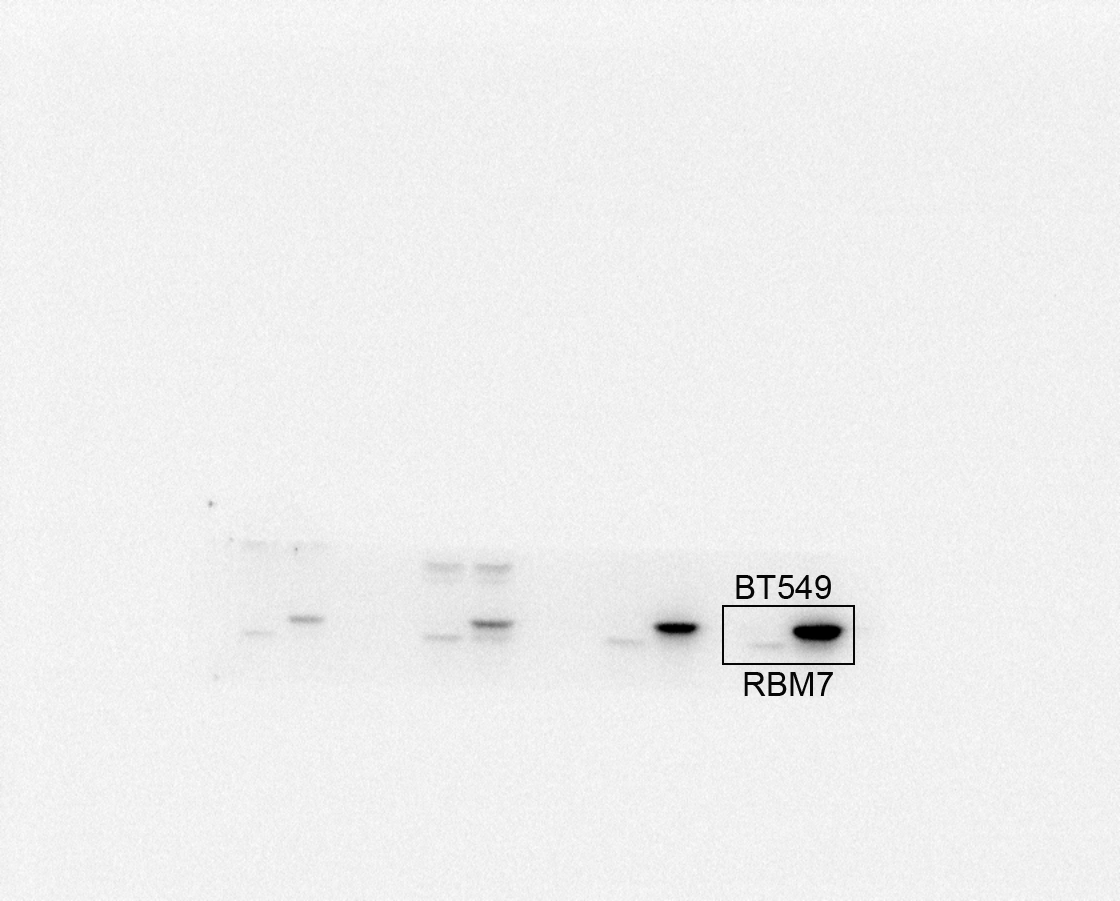

Supplement: Figure 2—figure supplement 1—source data 3. [file elife-95318-fig2-figsupp1-data3.zip › Figure 2-figure supplement 1-Source data 3/BT549-RBM7.tif]

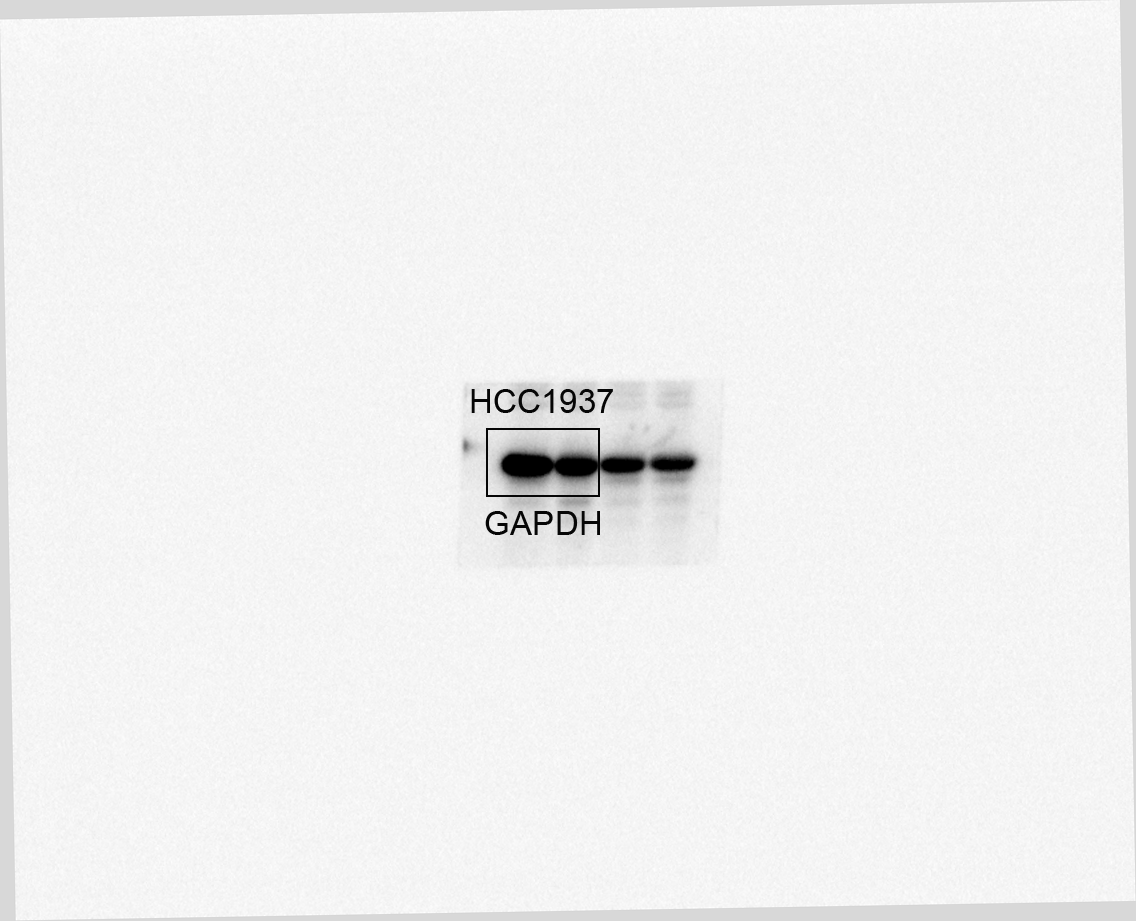

Supplement: Figure 2—figure supplement 1—source data 3. [file elife-95318-fig2-figsupp1-data3.zip › Figure 2-figure supplement 1-Source data 3/HCC1937 GAPDH.tif]

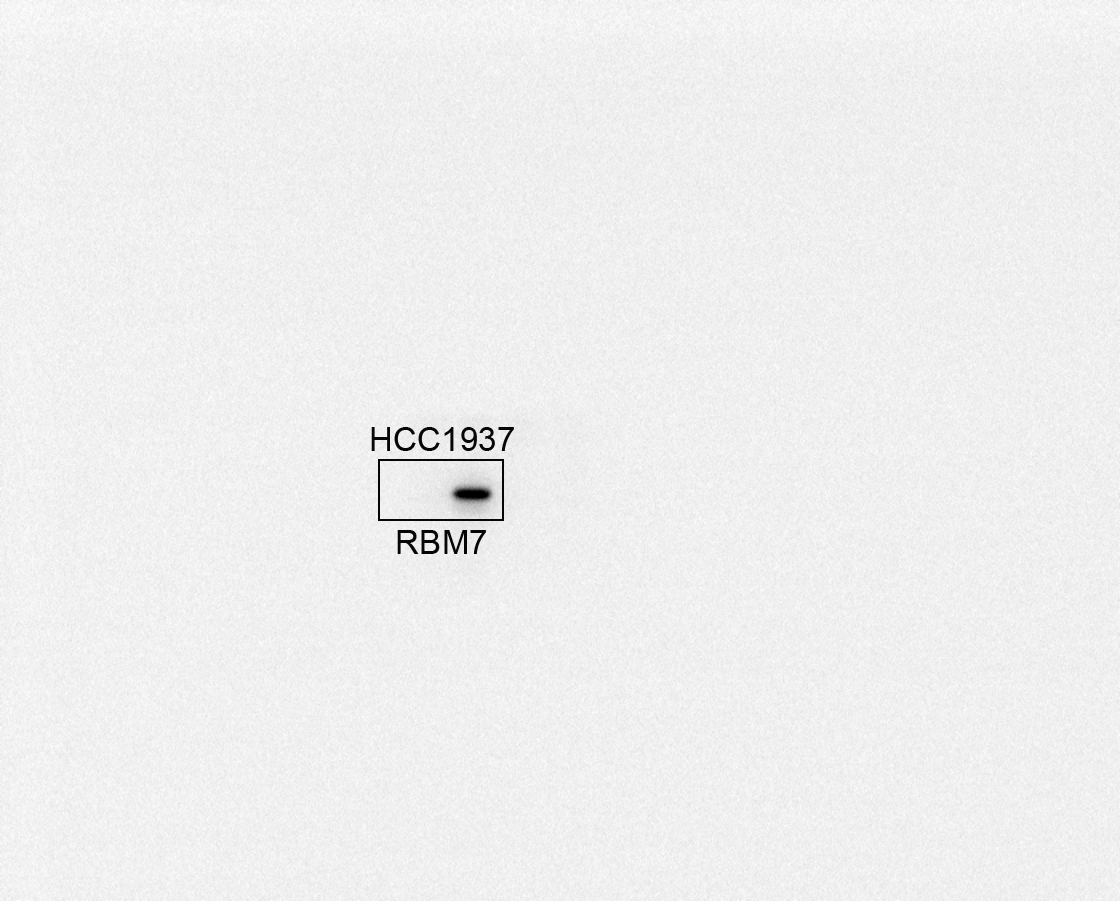

Supplement: Figure 2—figure supplement 1—source data 3. [file elife-95318-fig2-figsupp1-data3.zip › Figure 2-figure supplement 1-Source data 3/HCC1937 RBM7.tif]

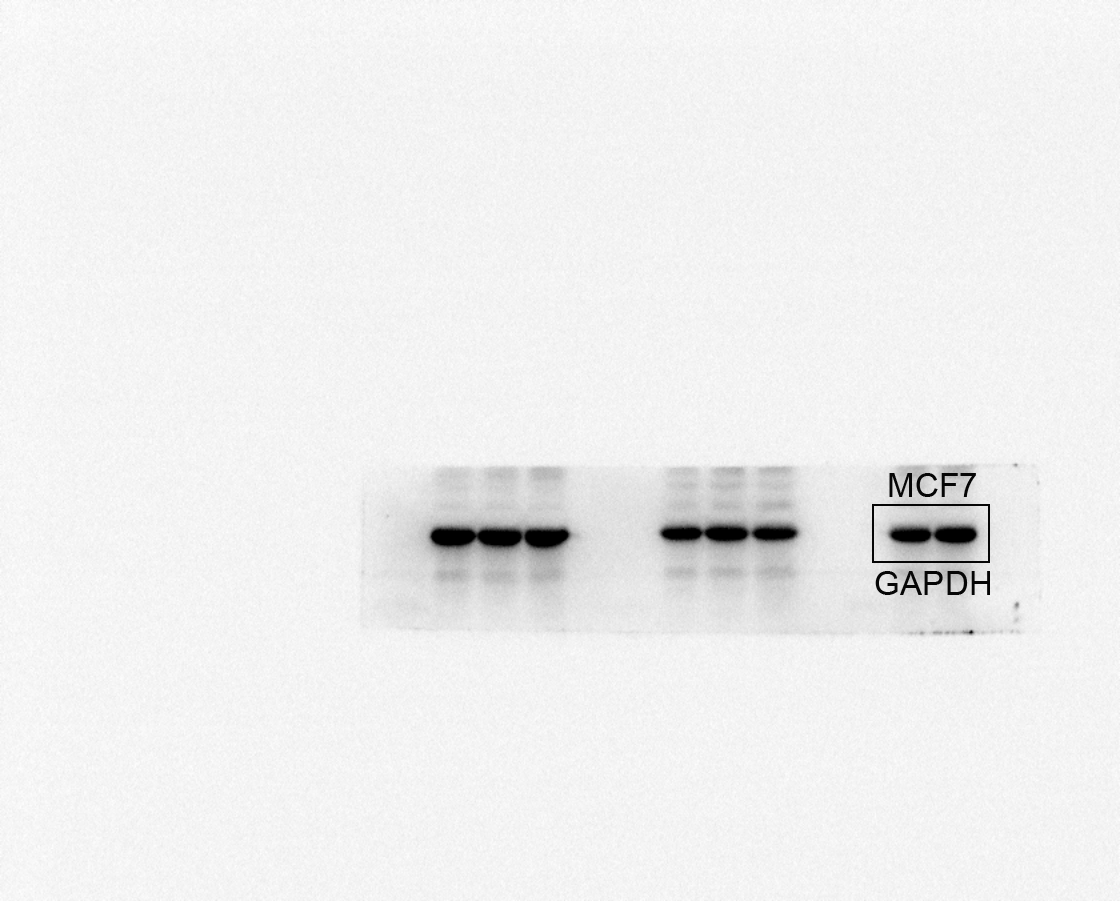

Supplement: Figure 2—figure supplement 1—source data 3. [file elife-95318-fig2-figsupp1-data3.zip › Figure 2-figure supplement 1-Source data 3/MCF7-GAPDH.tif]

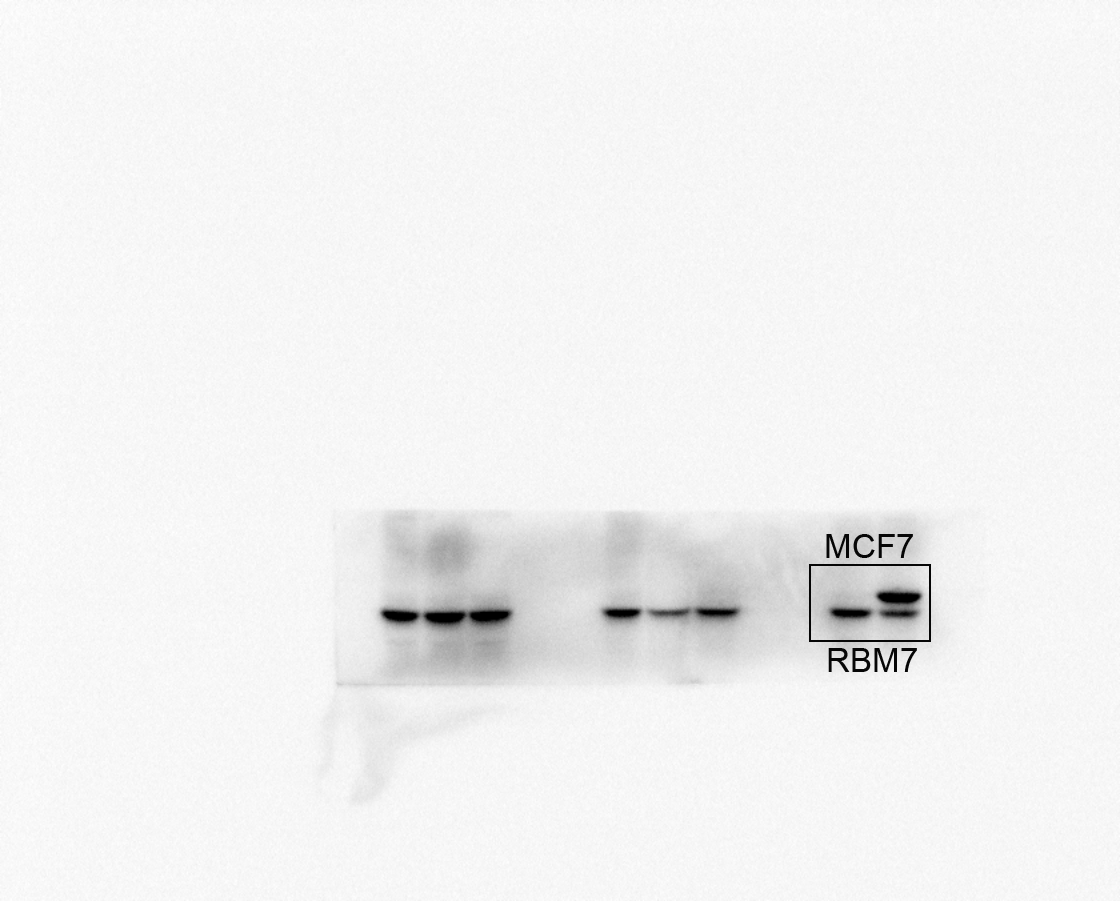

Supplement: Figure 2—figure supplement 1—source data 3. [file elife-95318-fig2-figsupp1-data3.zip › Figure 2-figure supplement 1-Source data 3/MCF7-RBM7.tif]

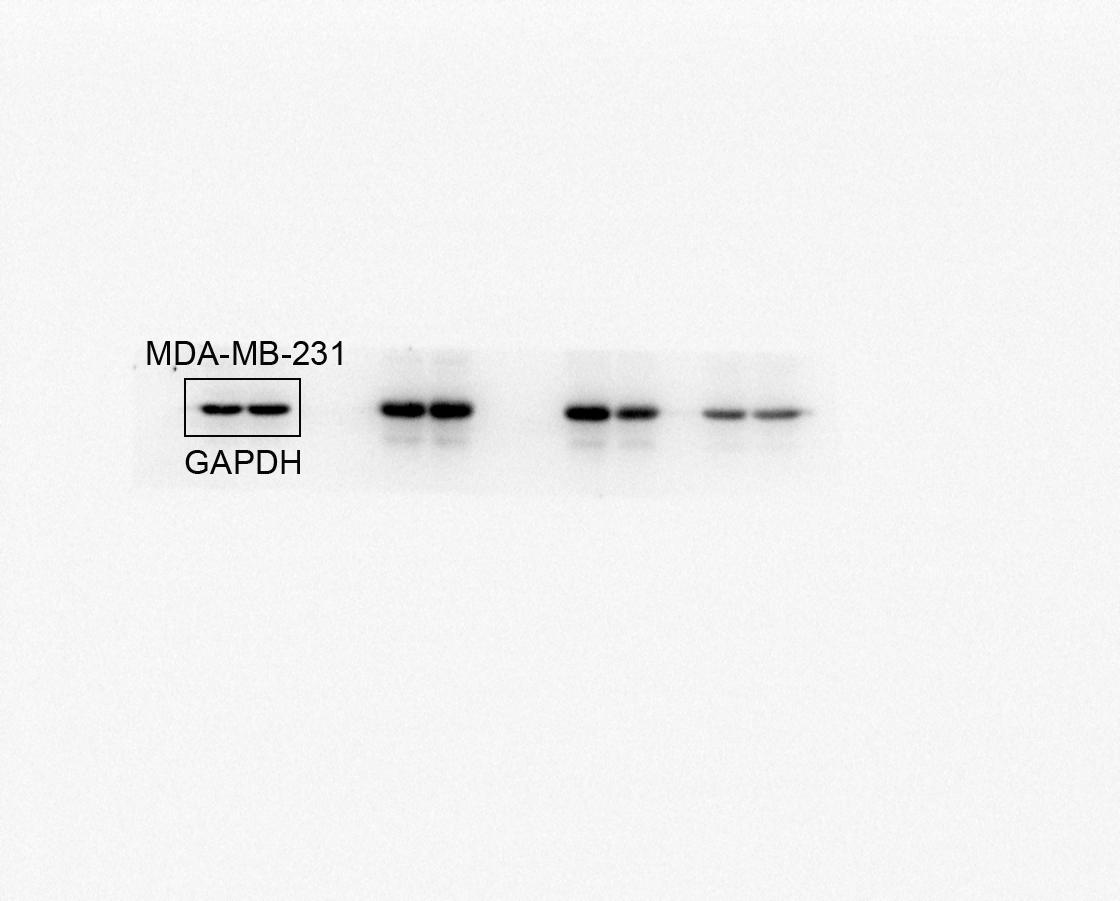

Supplement: Figure 2—figure supplement 1—source data 3. [file elife-95318-fig2-figsupp1-data3.zip › Figure 2-figure supplement 1-Source data 3/MDA-MB-231 GAPDH.tif]

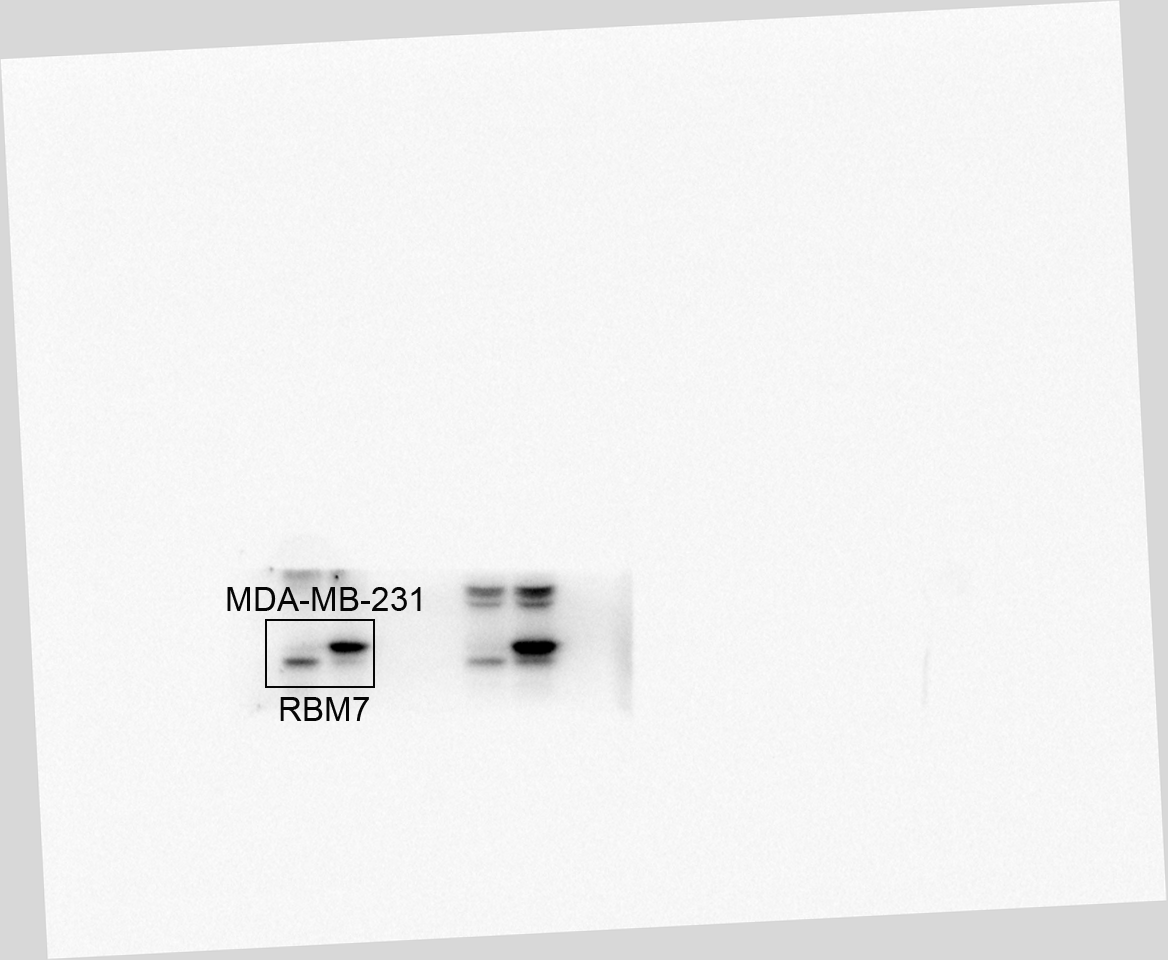

Supplement: Figure 2—figure supplement 1—source data 3. [file elife-95318-fig2-figsupp1-data3.zip › Figure 2-figure supplement 1-Source data 3/MDA-MB-231 RBM7.tif]

## Sup Figure2F

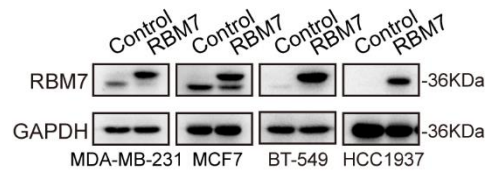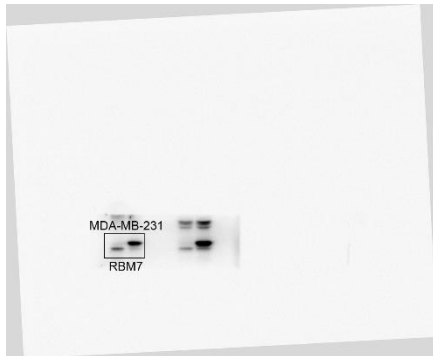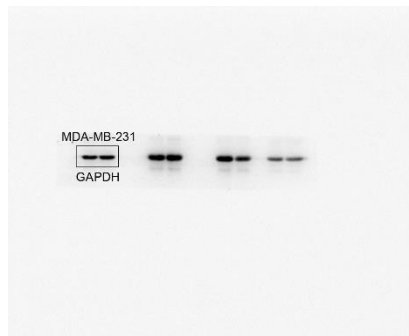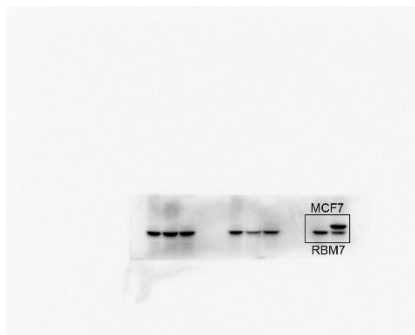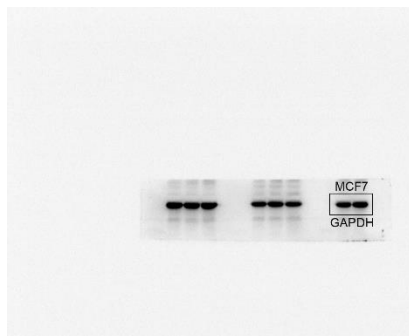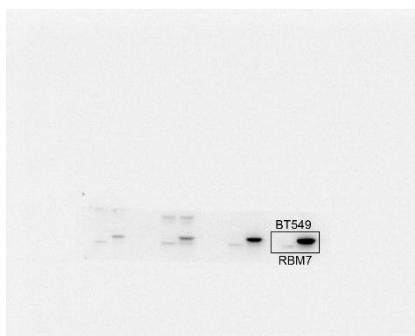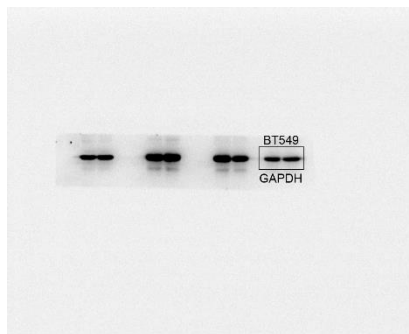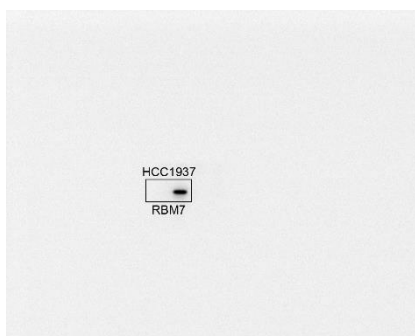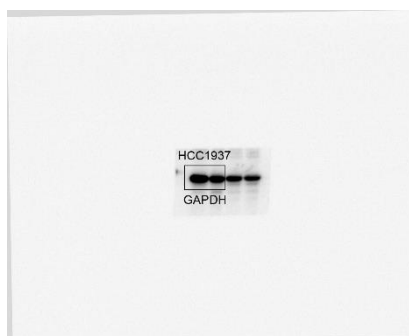

Supplement: Figure 2—figure supplement 1—source data 4. [file elife-95318-fig2-figsupp1-data4.zip › Figure 2-figure supplement 1-Source data 4/Sup Fig2F.pdf]

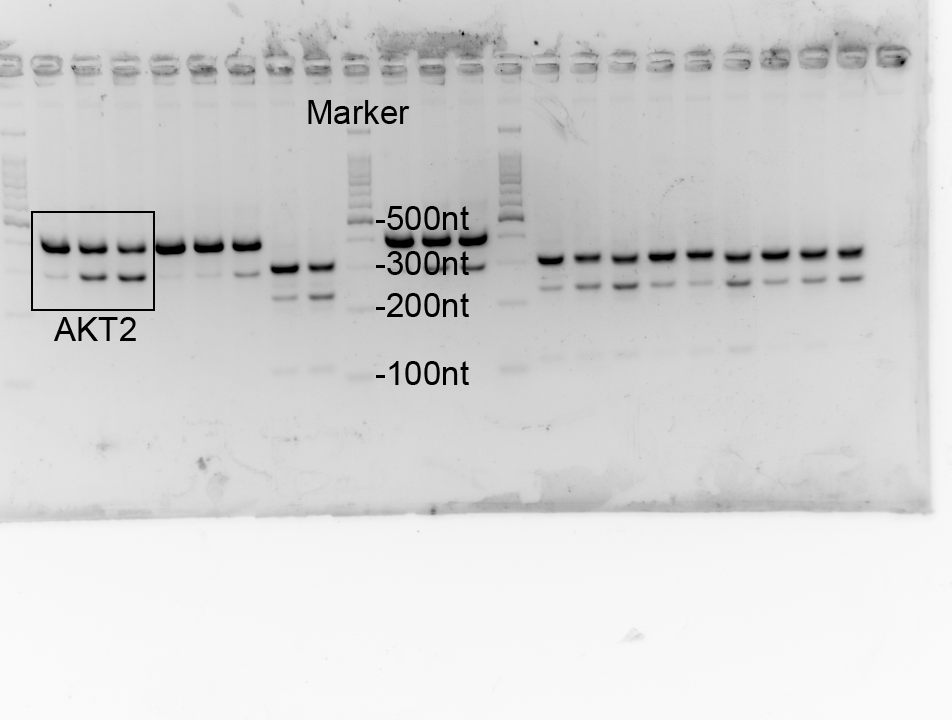

Supplement: Figure 3—source data 1. [file elife-95318-fig3-data1.zip › Figure3-Source data 1/Uncropped RT-PCR gels-Fig3F/AKT2.tif]

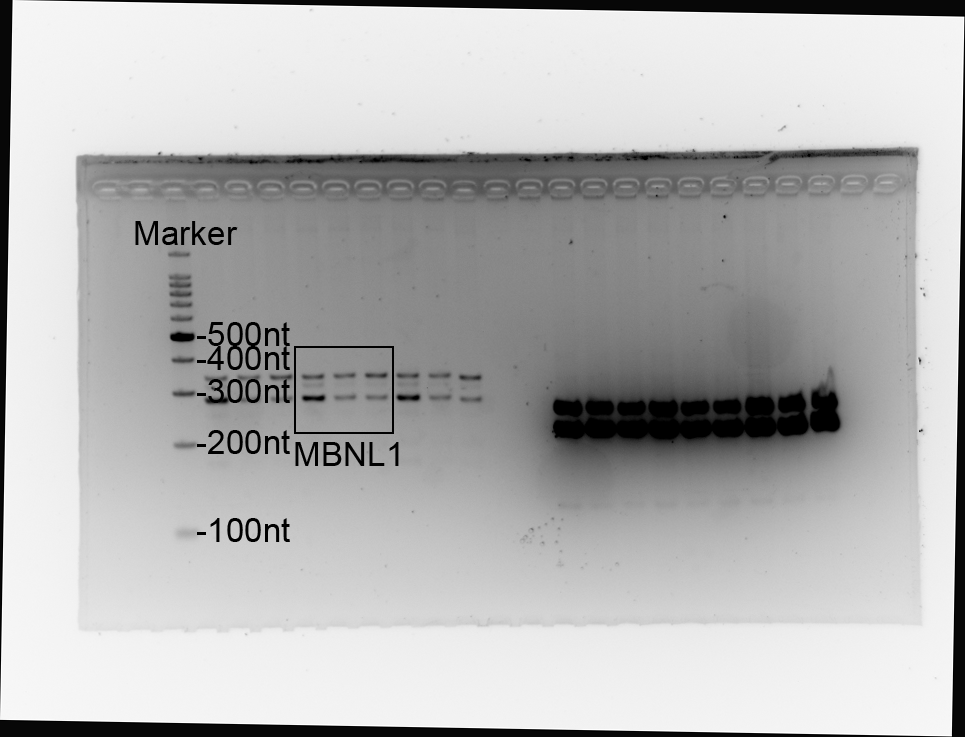

Supplement: Figure 3—source data 1. [file elife-95318-fig3-data1.zip › Figure3-Source data 1/Uncropped RT-PCR gels-Fig3F/MBNL1.tif]

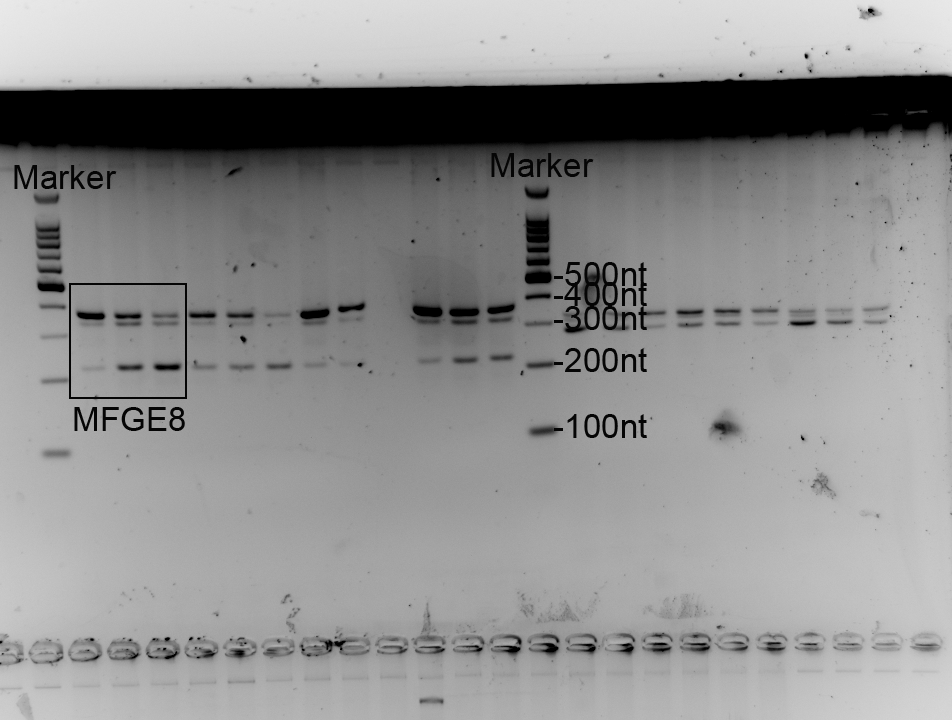

Supplement: Figure 3—source data 1. [file elife-95318-fig3-data1.zip › Figure3-Source data 1/Uncropped RT-PCR gels-Fig3F/MFGE8.tif]

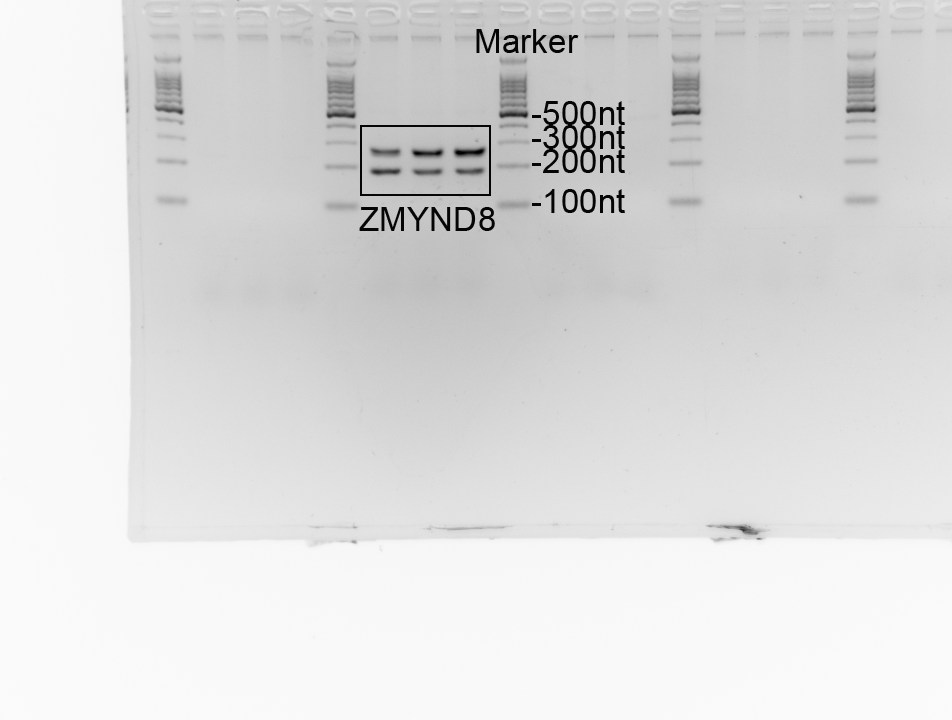

Supplement: Figure 3—source data 1. [file elife-95318-fig3-data1.zip › Figure3-Source data 1/Uncropped RT-PCR gels-Fig3F/ZMYND8.tif]

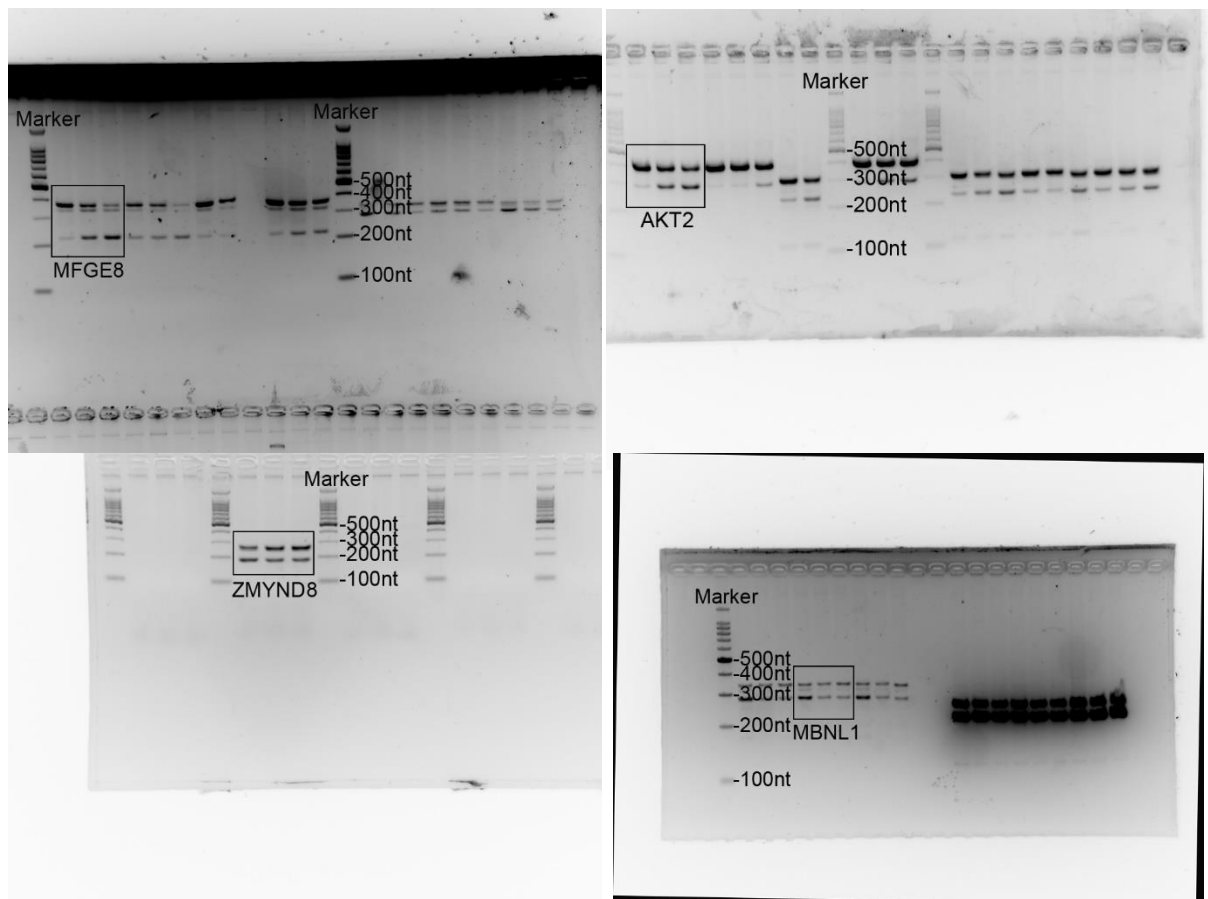

Figure 3F

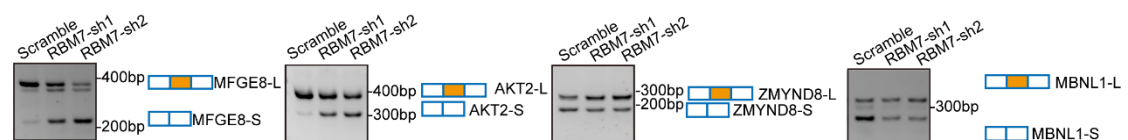

Supplement: Figure 3—source data 2. [file elife-95318-fig3-data2.zip › Figure3-Source data 2/Figure 3 source data 2.pdf]

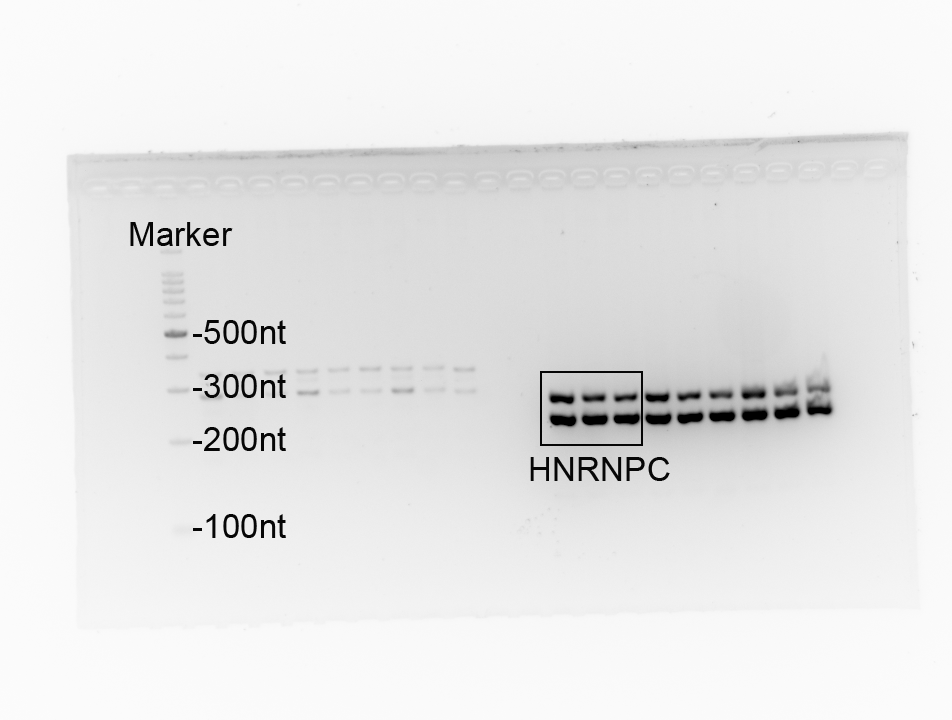

Supplement: Figure 3—figure supplement 1—source data 1. [file elife-95318-fig3-figsupp1-data1.zip › Figure3-figure supplement 1-Source data 1/Uncropped gels-Sup Fig3B/HNRNPC.tif]

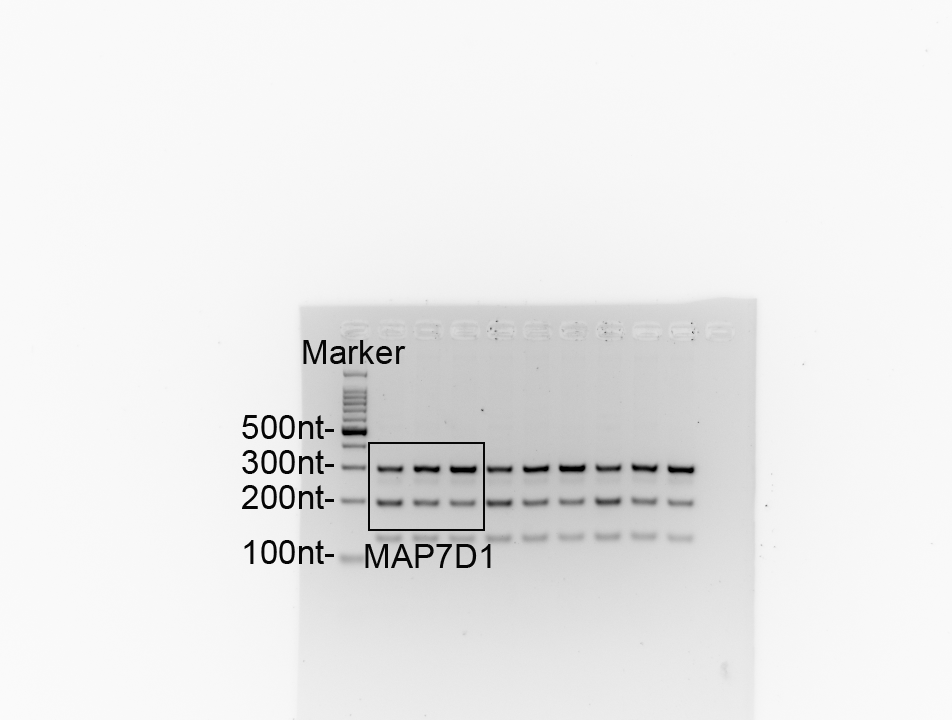

Supplement: Figure 3—figure supplement 1—source data 1. [file elife-95318-fig3-figsupp1-data1.zip › Figure3-figure supplement 1-Source data 1/Uncropped gels-Sup Fig3B/MAP7D1.tif]

# Sup Figure3B

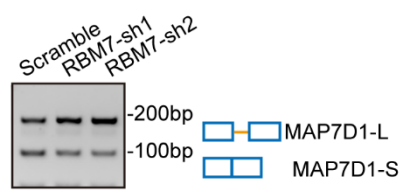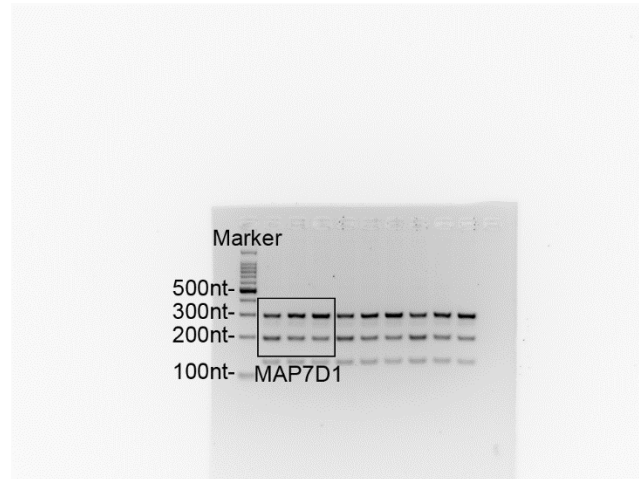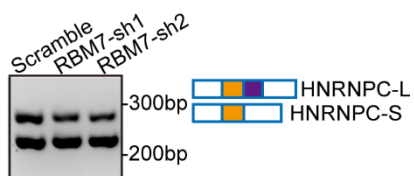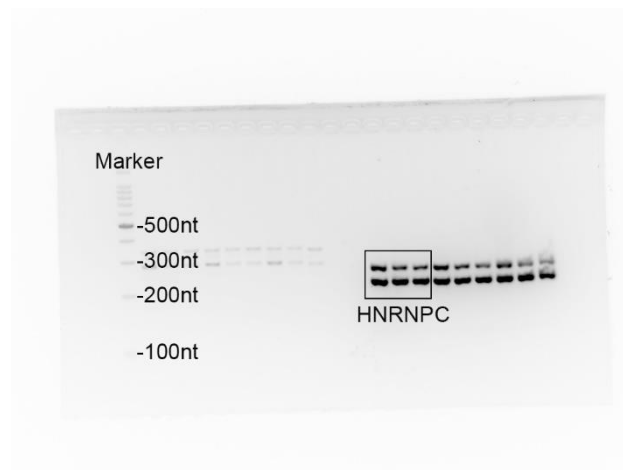

Supplement: Figure 3—figure supplement 1—source data 2. [file elife-95318-fig3-figsupp1-data2.zip › Figure3-figure supplement 1-Source data 2/Sup Fig3B.pdf]

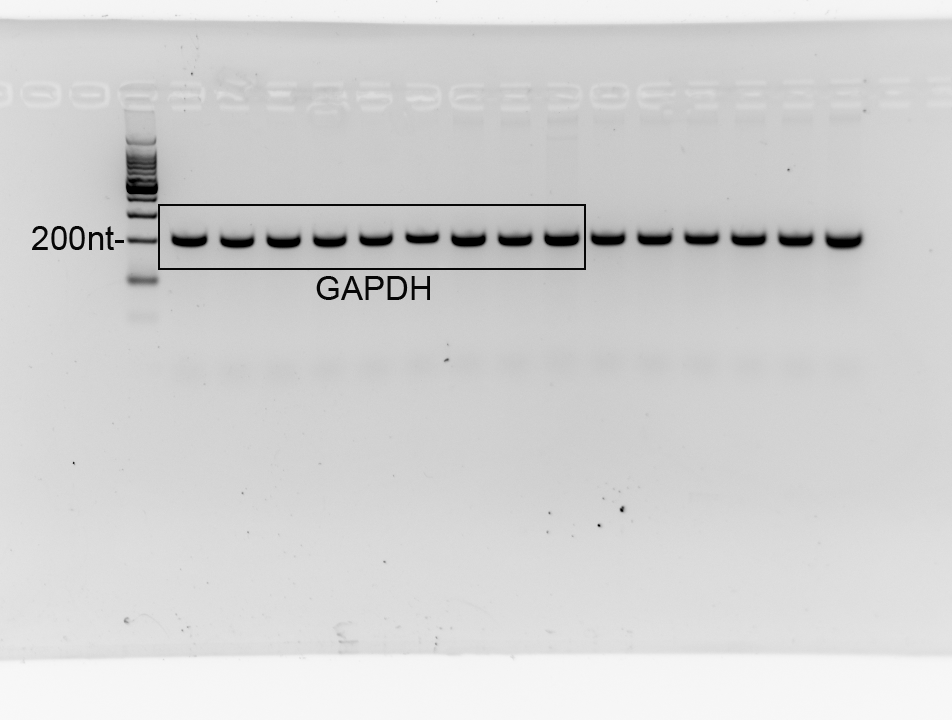

Supplement: Figure 3—figure supplement 1—source data 3. [file elife-95318-fig3-figsupp1-data3.zip › Figure3-figure supplement 1-Source data 3/Uncropped gels-Sup Fig3C/GAPDH.tif]

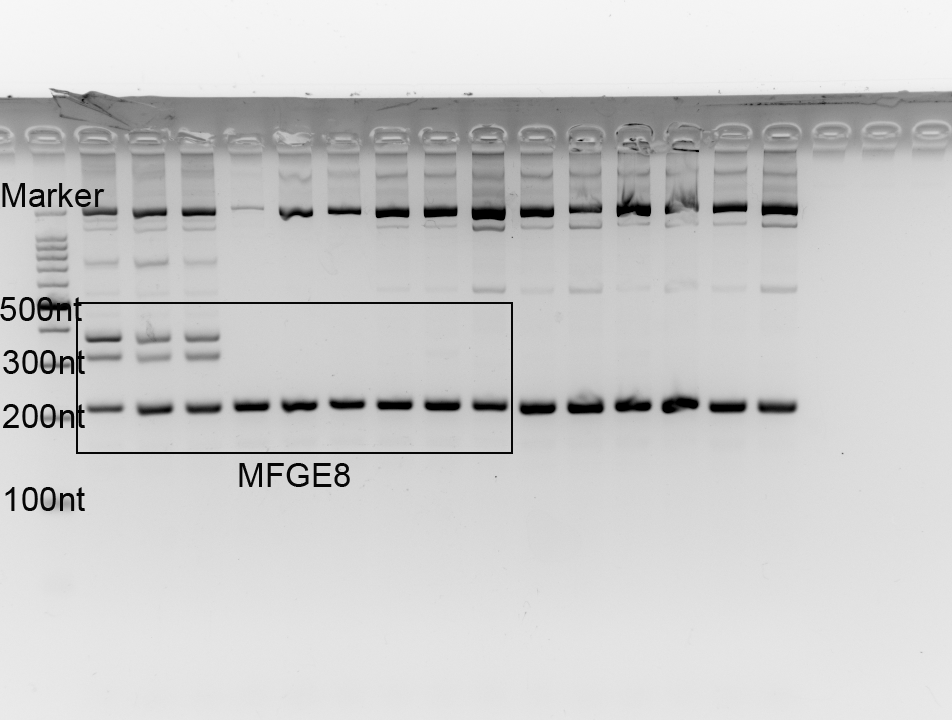

Supplement: Figure 3—figure supplement 1—source data 3. [file elife-95318-fig3-figsupp1-data3.zip › Figure3-figure supplement 1-Source data 3/Uncropped gels-Sup Fig3C/MFGE8.tif]

Sup Figure3C

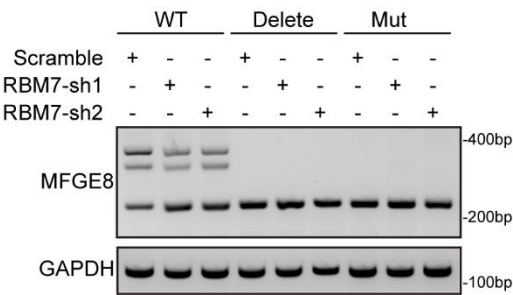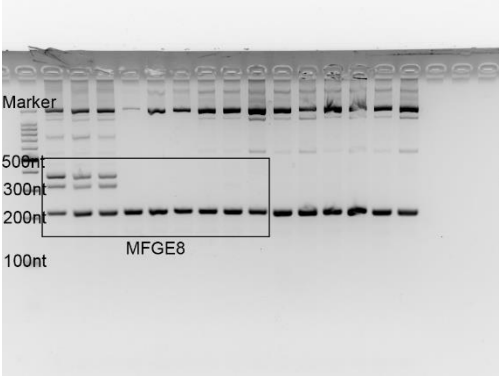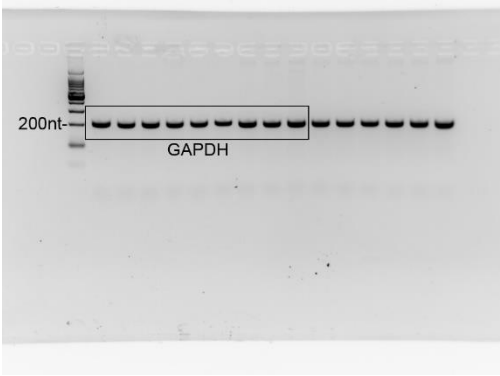

Supplement: Figure 3—figure supplement 1—source data 4. [file elife-95318-fig3-figsupp1-data4.zip › Figure3-figure supplement 1-Source data 4/Sup Fig3C.pdf]

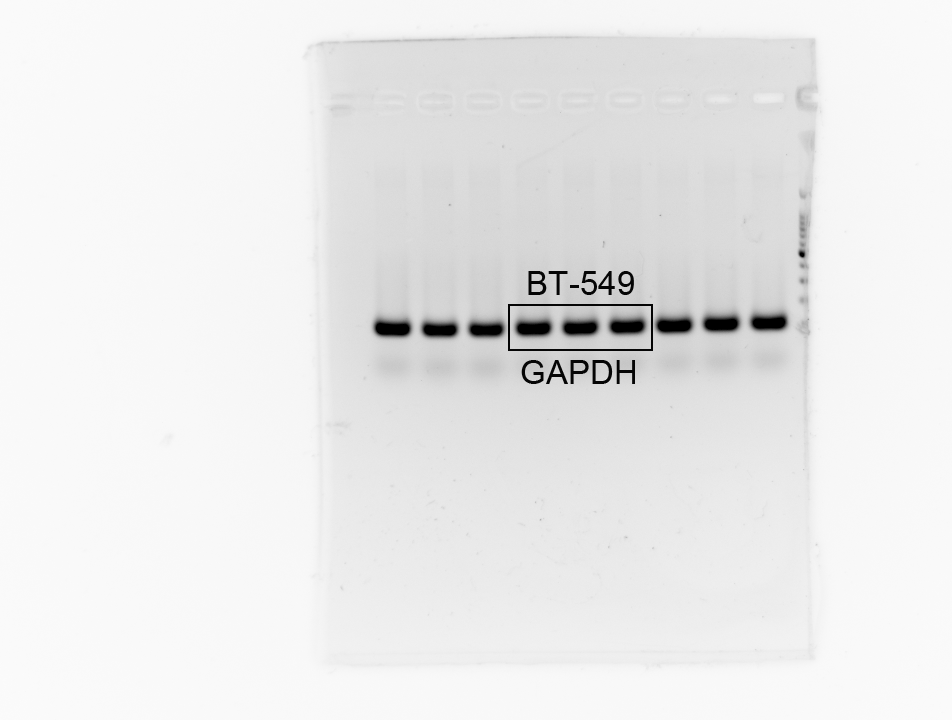

Supplement: Figure 4—source data 1. [file elife-95318-fig4-data1.zip › Figure4-Source data 1/Uncropped RT-PCR gels-Fig4C/BT-549/BT549-GAPDH.tif]

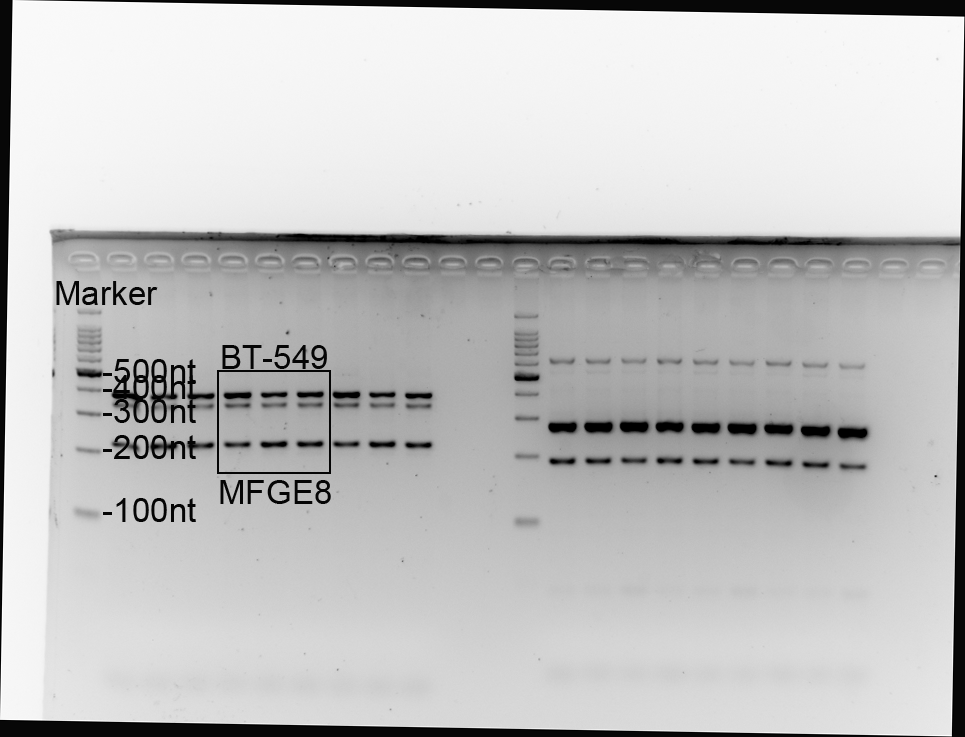

Supplement: Figure 4—source data 1. [file elife-95318-fig4-data1.zip › Figure4-Source data 1/Uncropped RT-PCR gels-Fig4C/BT-549/BT549-MFGE8.tif]

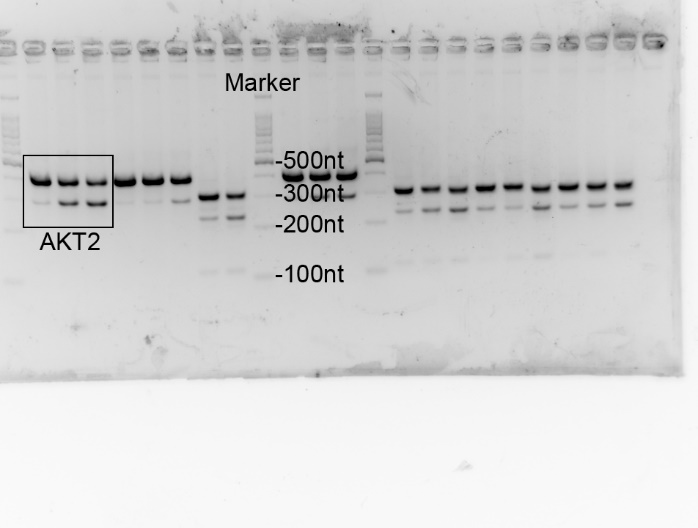

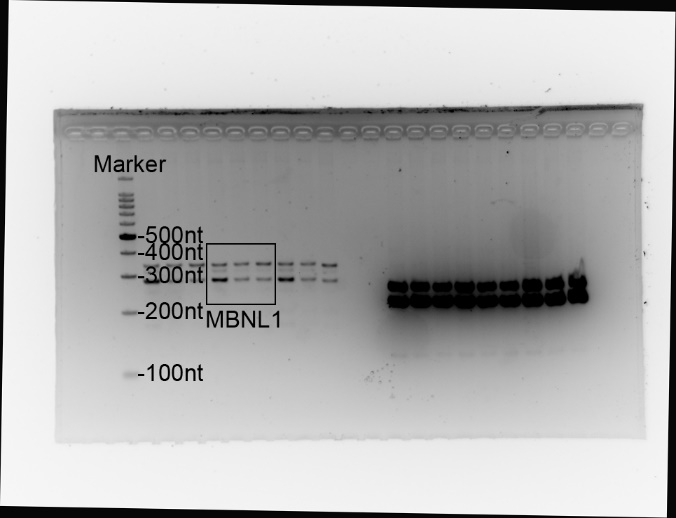

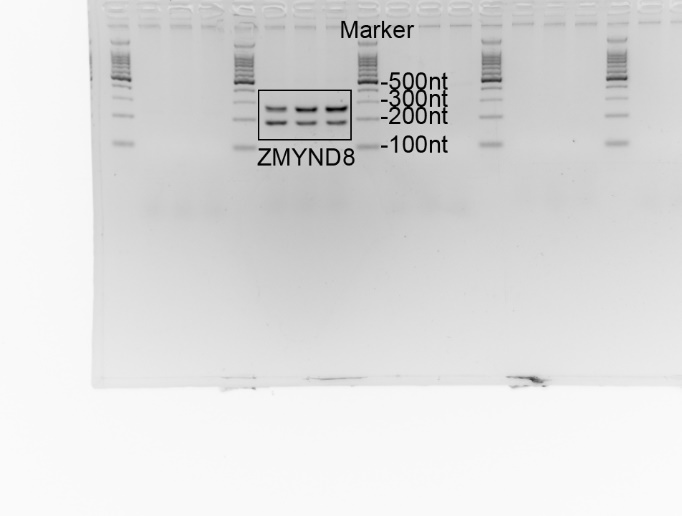

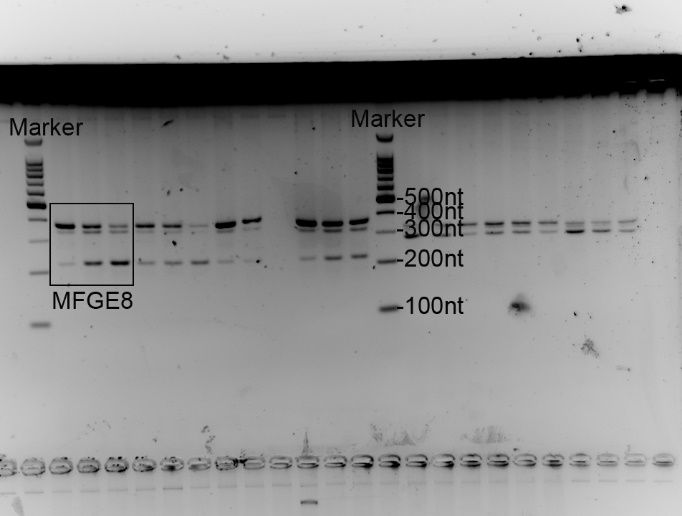
Figure 3F


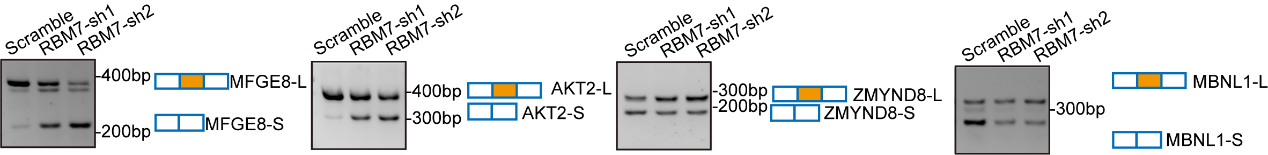

Supplement: Figure 4—source data 1. [file elife-95318-fig4-data1.zip › Figure4-Source data 1/Uncropped RT-PCR gels-Fig4C/Figure3-Source data 2/Figure 3F.docx]

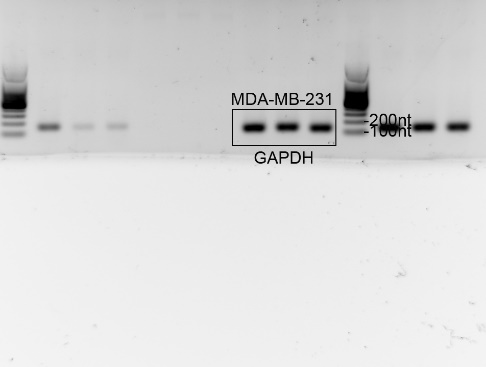

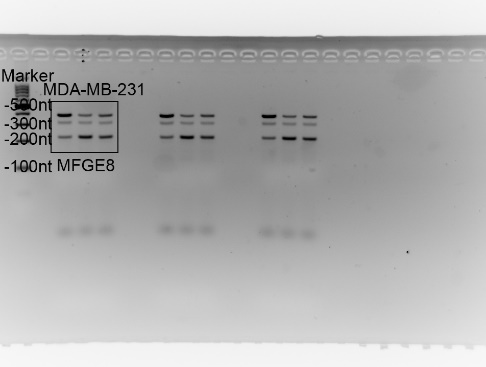




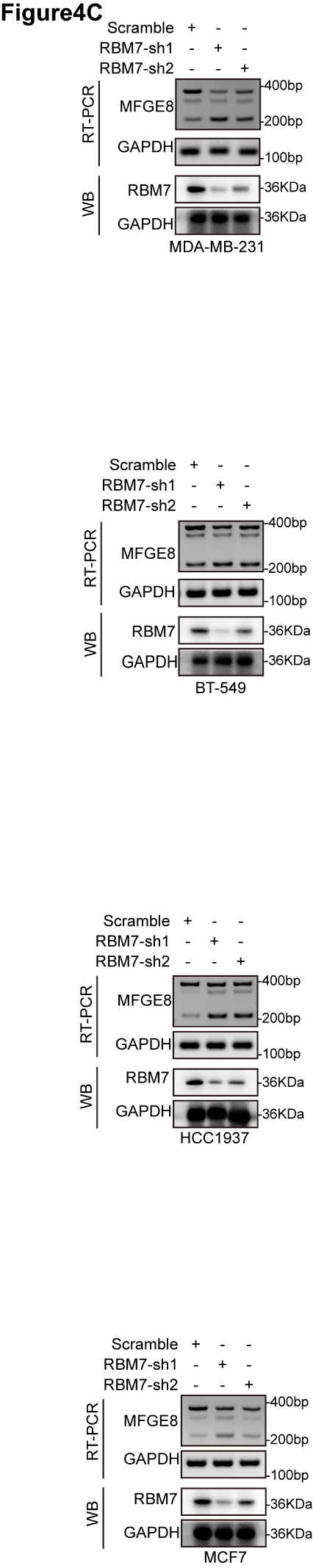





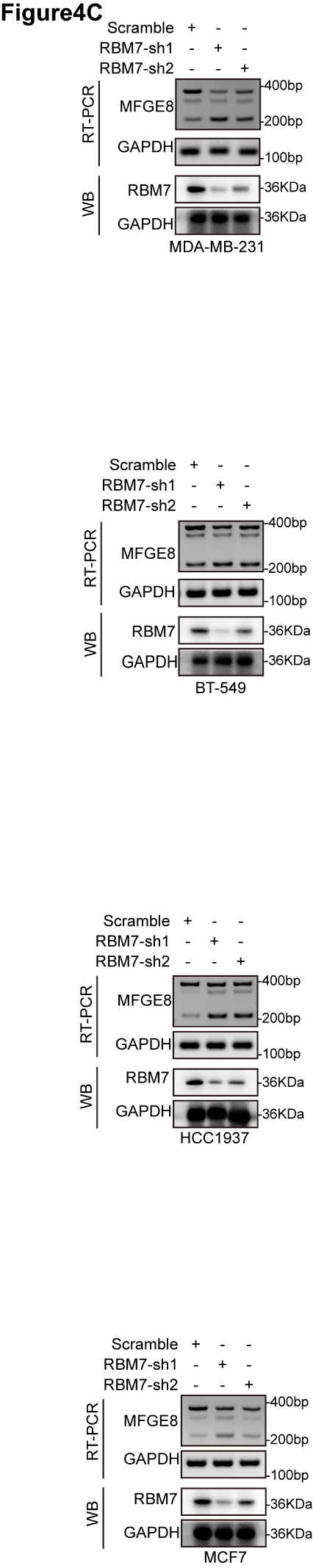




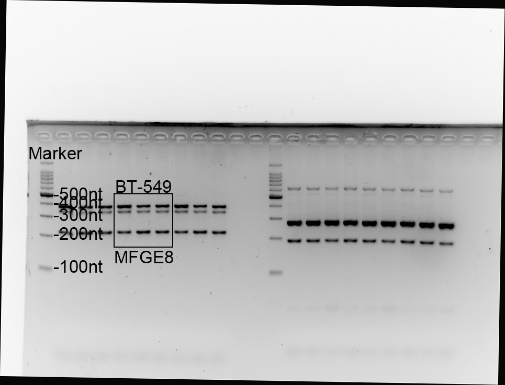








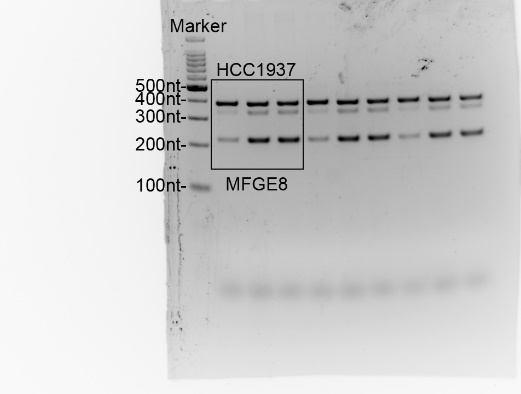

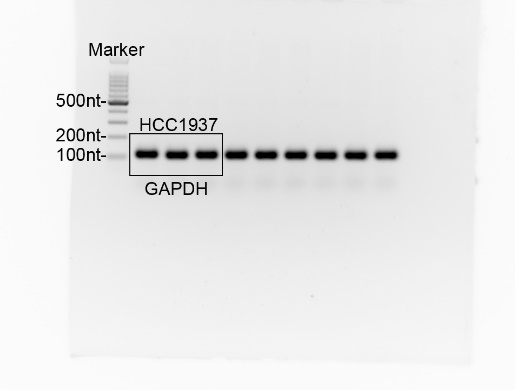



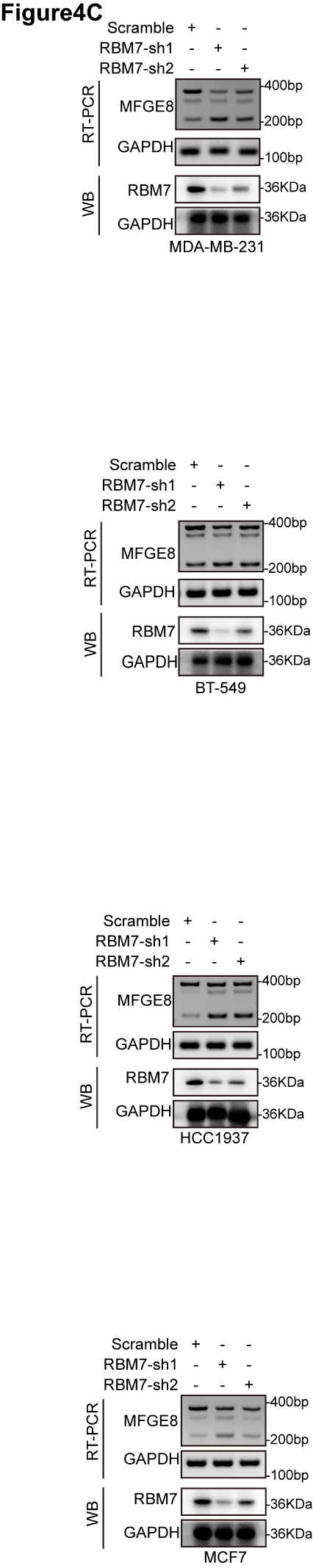





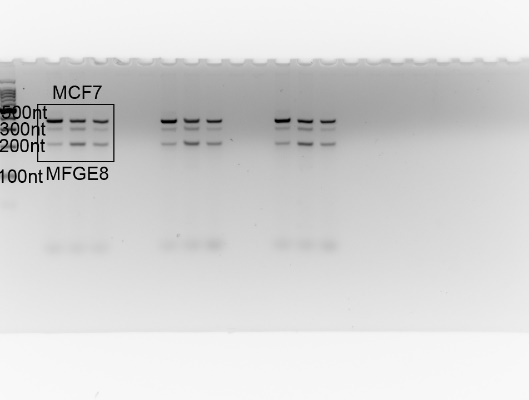




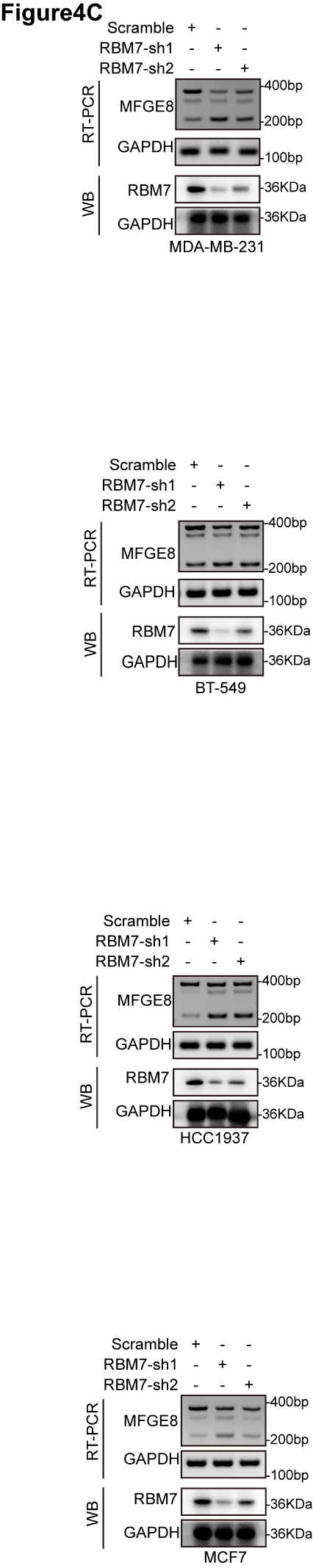

Supplement: Figure 4—source data 1. [file elife-95318-fig4-data1.zip › Figure4-Source data 1/Uncropped RT-PCR gels-Fig4C/Figure4-Source data 2/Figure4C.docx]

Figure4C

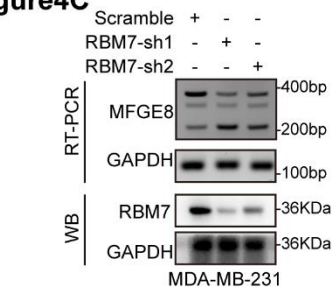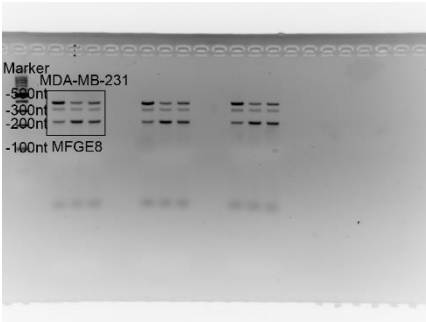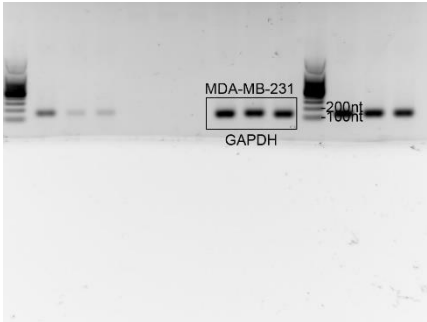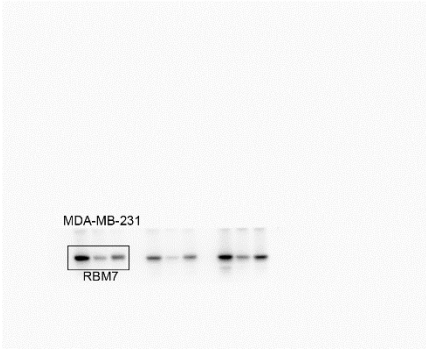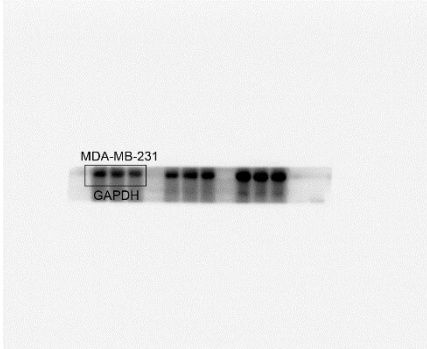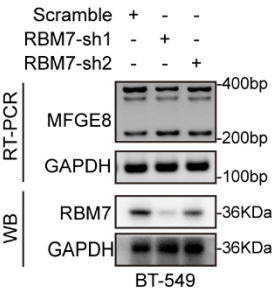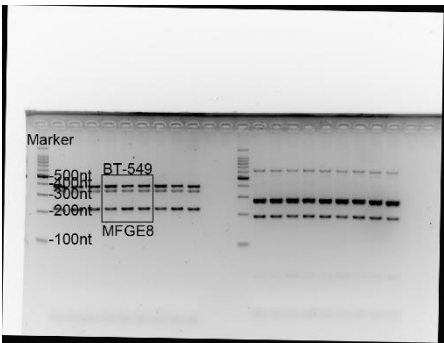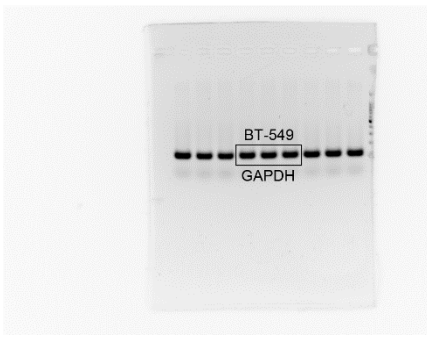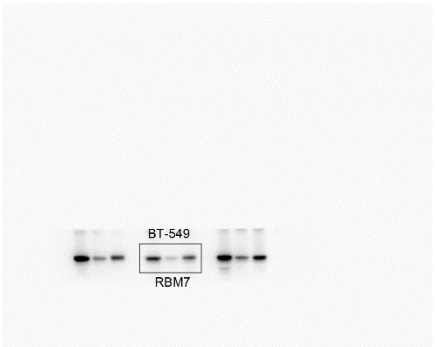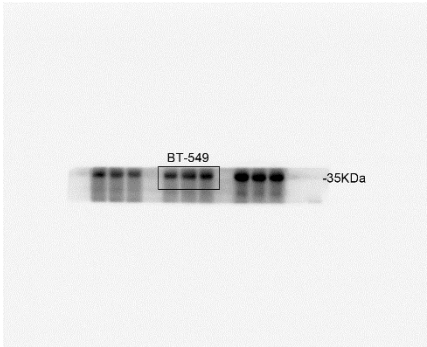

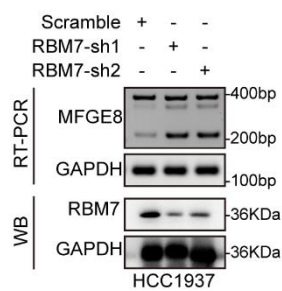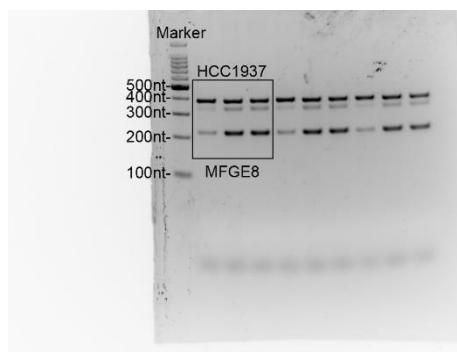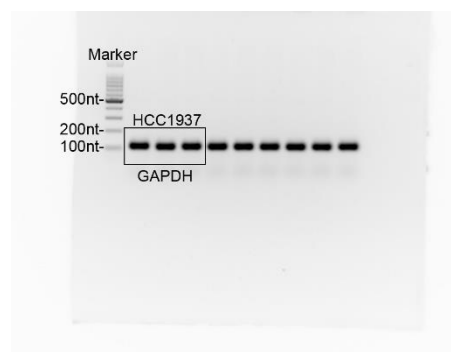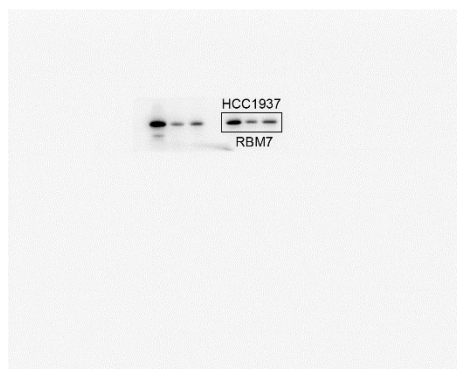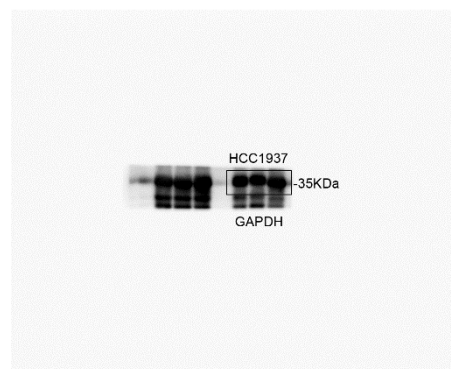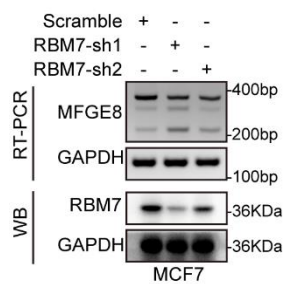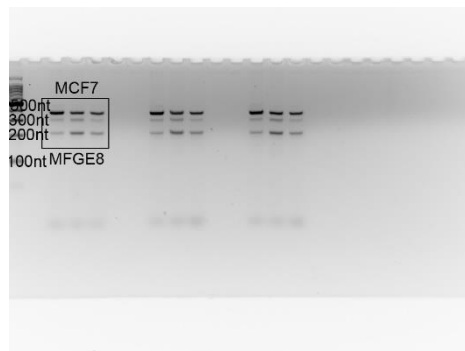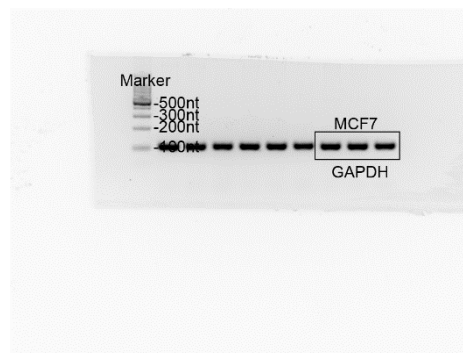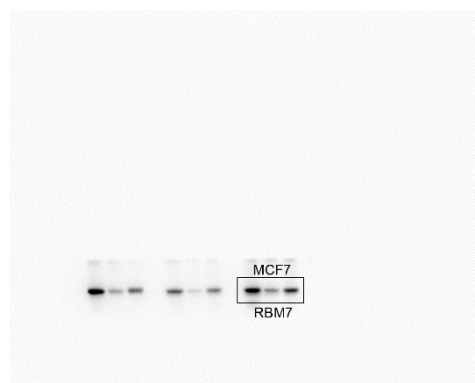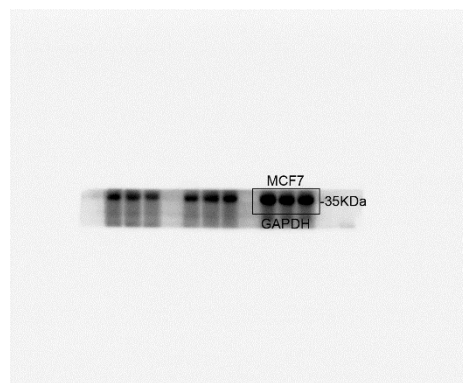

Supplement: Figure 4—source data 1. [file elife-95318-fig4-data1.zip › Figure4-Source data 1/Uncropped RT-PCR gels-Fig4C/Figure4-Source data 2/Figure4C.pdf]

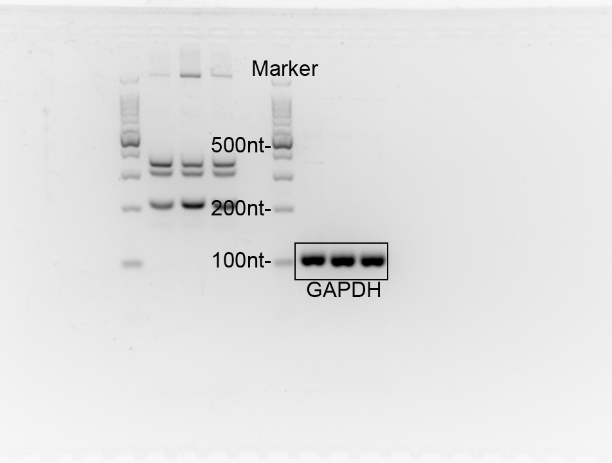

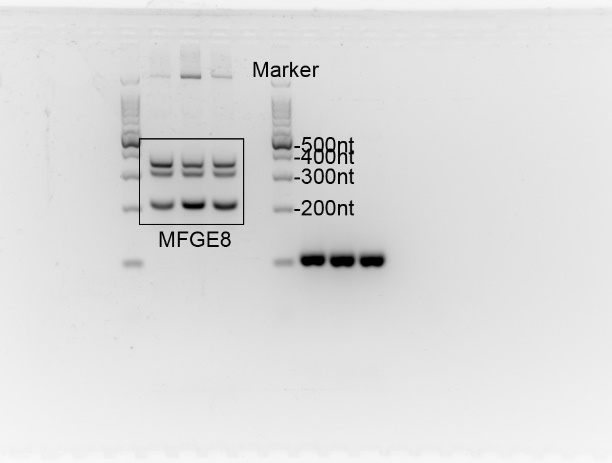

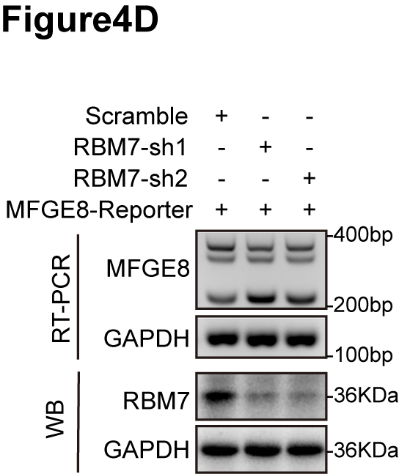

Supplement: Figure 4—source data 1. [file elife-95318-fig4-data1.zip › Figure4-Source data 1/Uncropped RT-PCR gels-Fig4C/Figure4-Source data 4/Figure4D.docx]

Figure4D

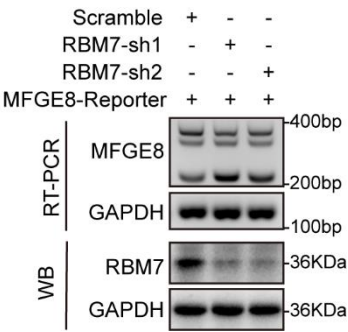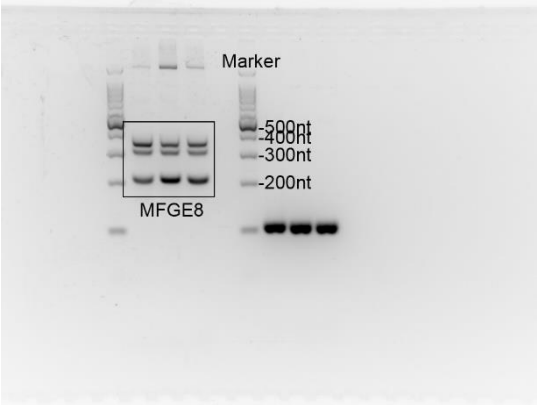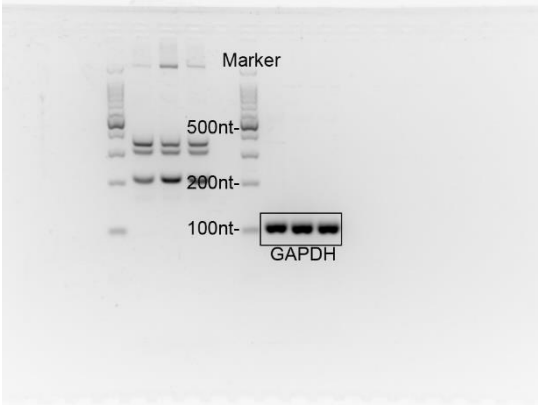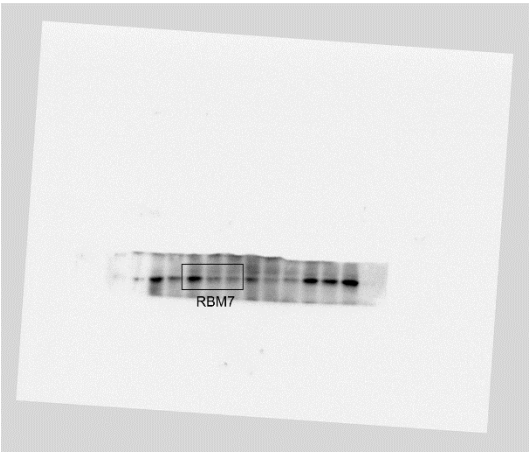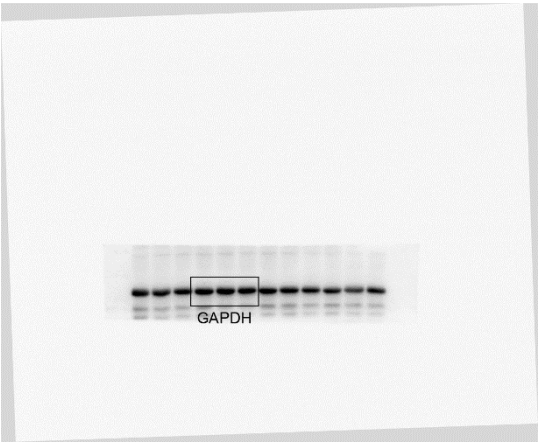

Supplement: Figure 4—source data 1. [file elife-95318-fig4-data1.zip › Figure4-Source data 1/Uncropped RT-PCR gels-Fig4C/Figure4-Source data 4/Figure4D.pdf]

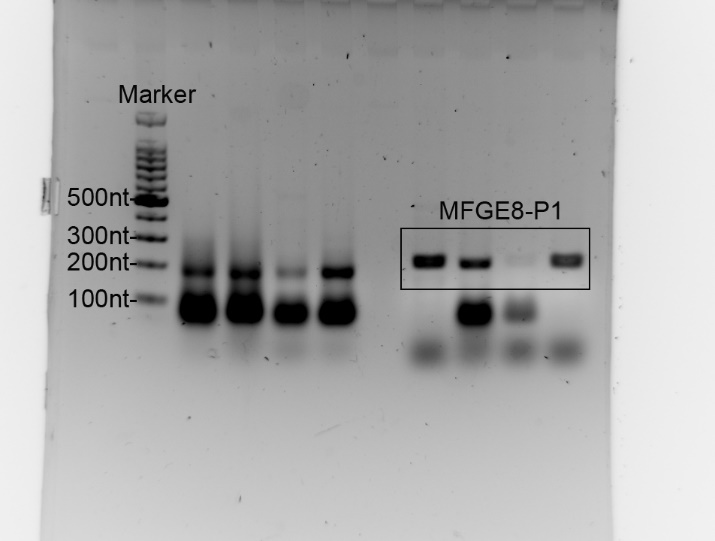

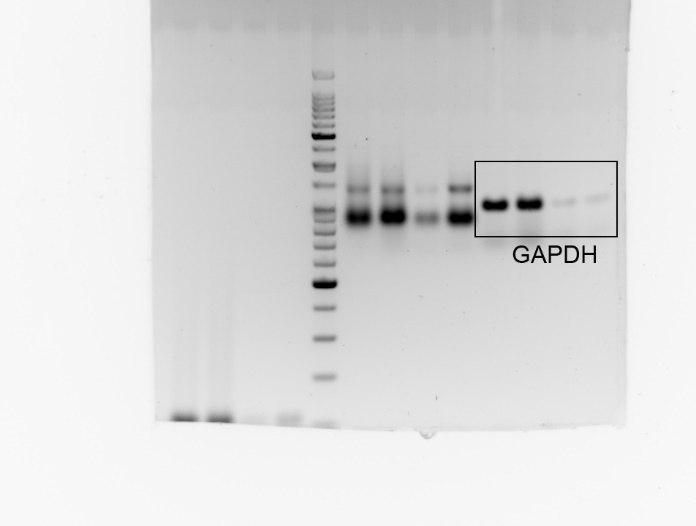

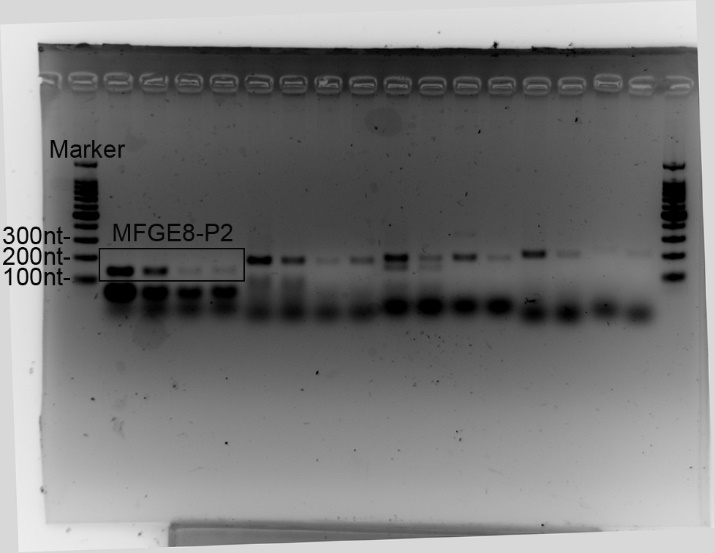

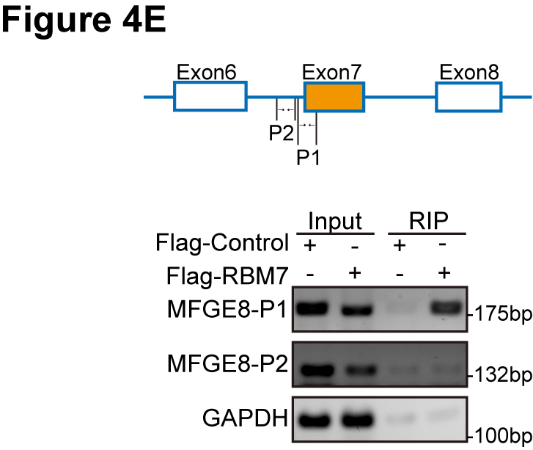

Supplement: Figure 4—source data 1. [file elife-95318-fig4-data1.zip › Figure4-Source data 1/Uncropped RT-PCR gels-Fig4C/Figure4-Source data 6/Figure4E.docx]

Figure 4E

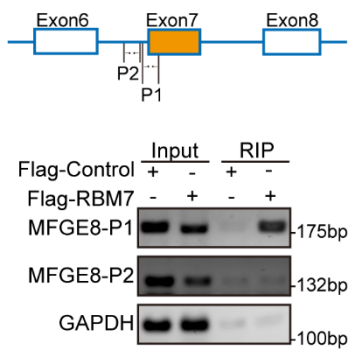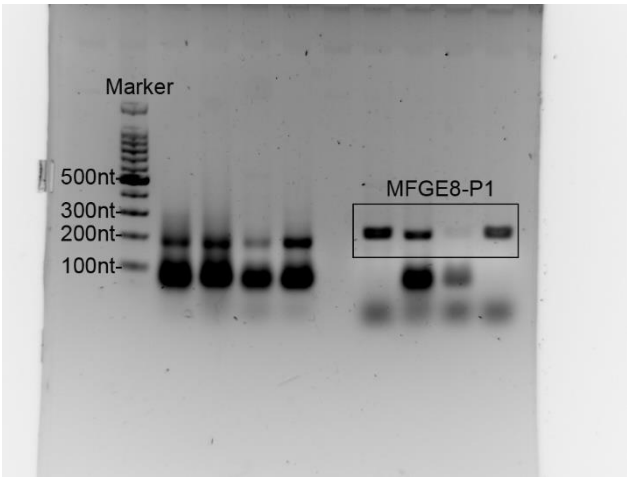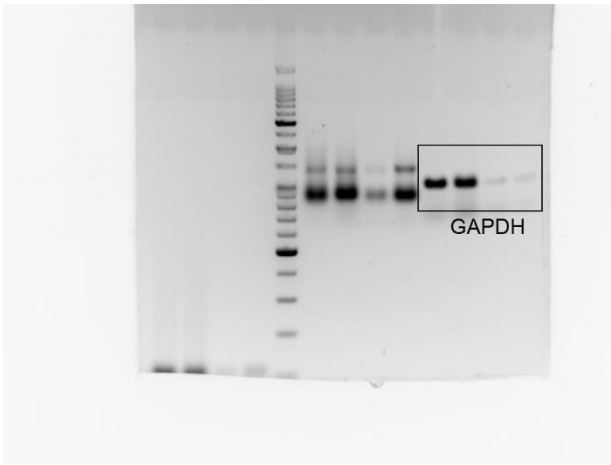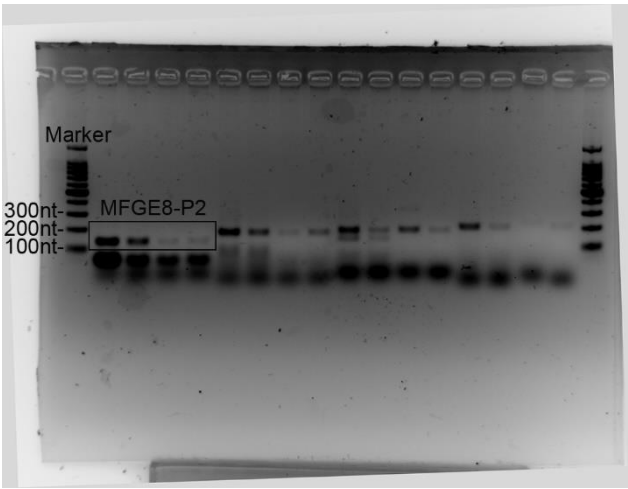

Supplement: Figure 4—source data 1. [file elife-95318-fig4-data1.zip › Figure4-Source data 1/Uncropped RT-PCR gels-Fig4C/Figure4-Source data 6/Figure4E.pdf]

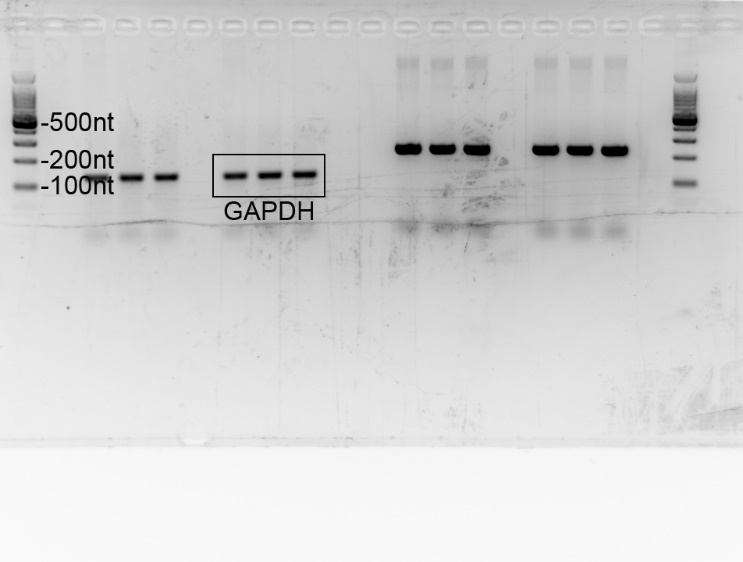

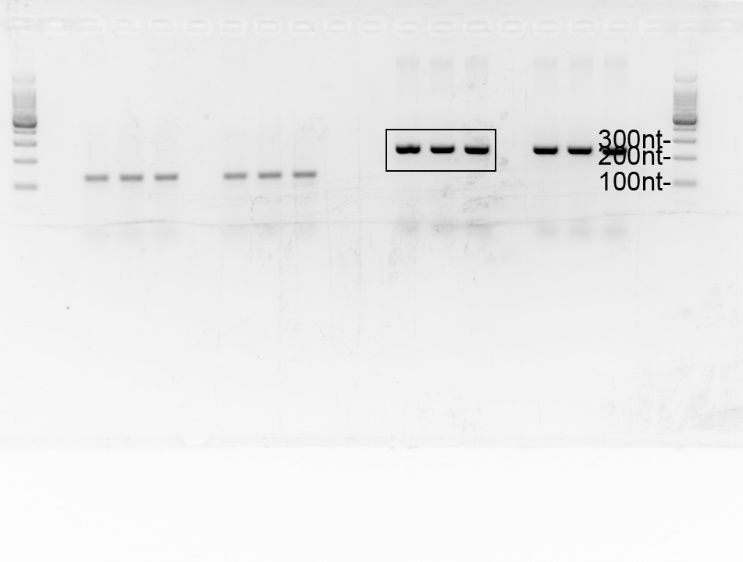

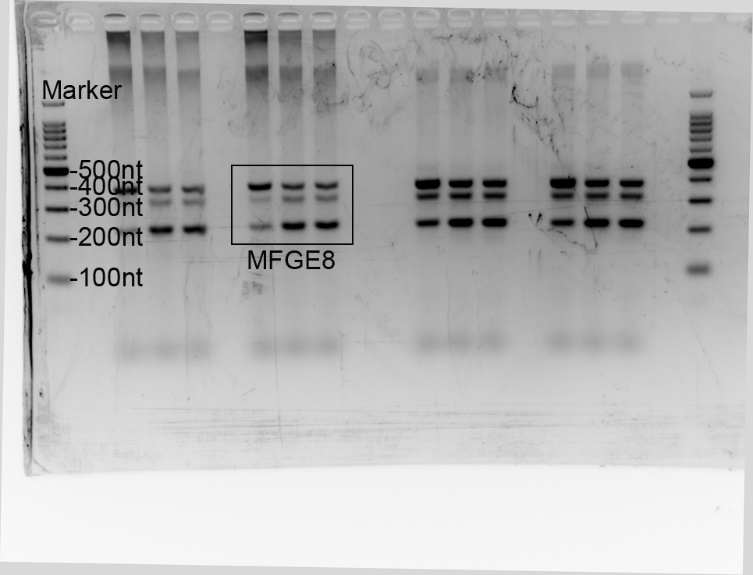

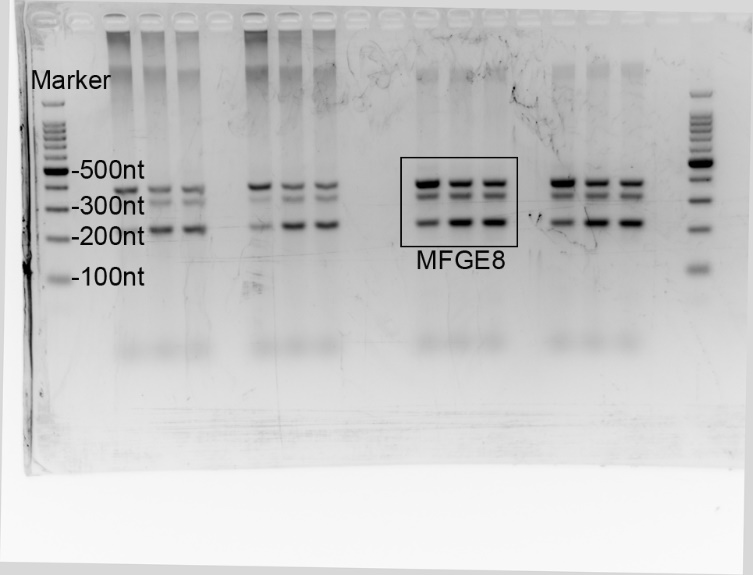

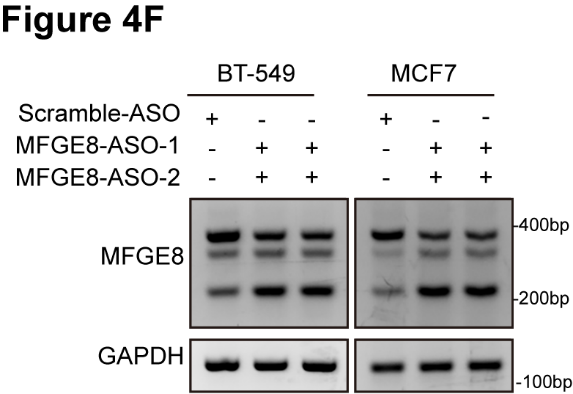

Supplement: Figure 4—source data 1. [file elife-95318-fig4-data1.zip › Figure4-Source data 1/Uncropped RT-PCR gels-Fig4C/Figure4-Source data 8/Figure4F.docx]

Figure 4F

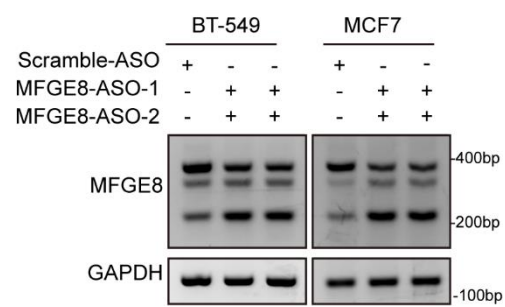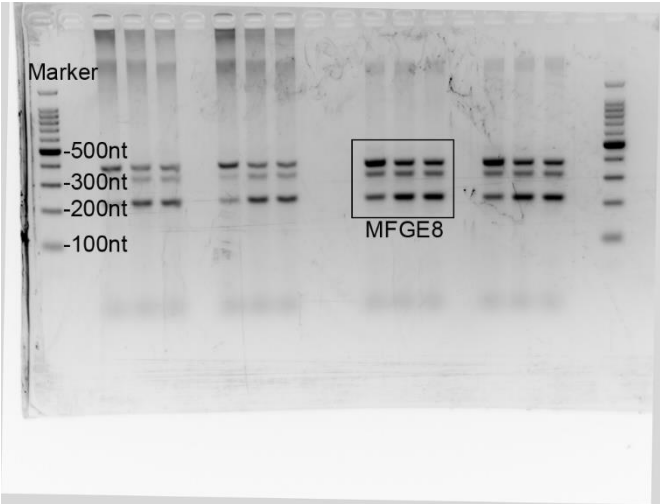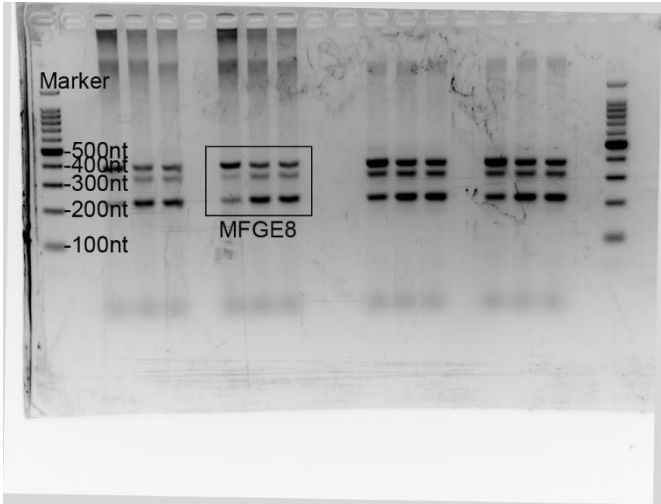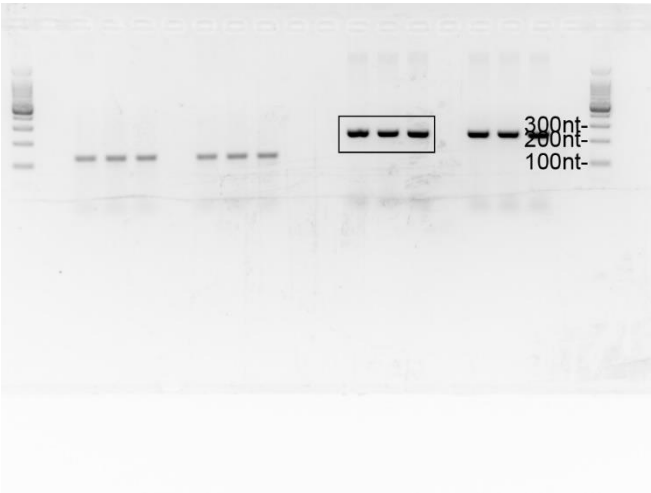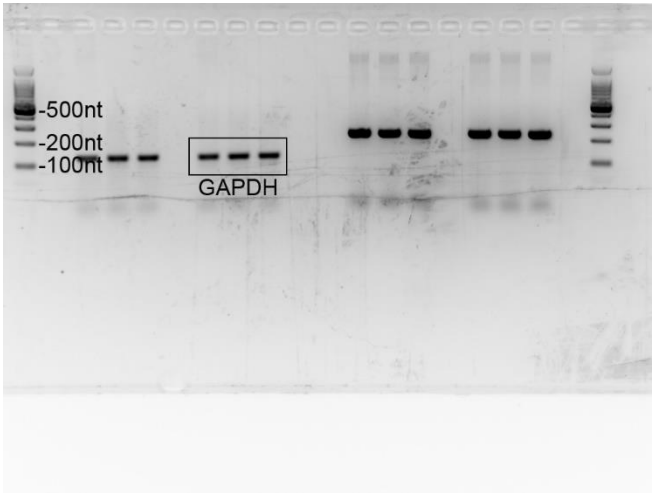

Supplement: Figure 4—source data 1. [file elife-95318-fig4-data1.zip › Figure4-Source data 1/Uncropped RT-PCR gels-Fig4C/Figure4-Source data 8/Figure4F.pdf]

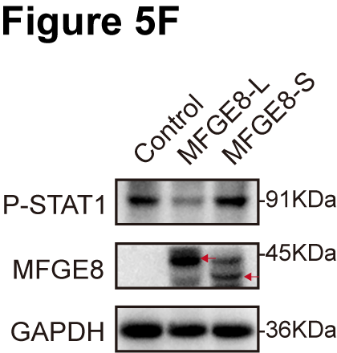

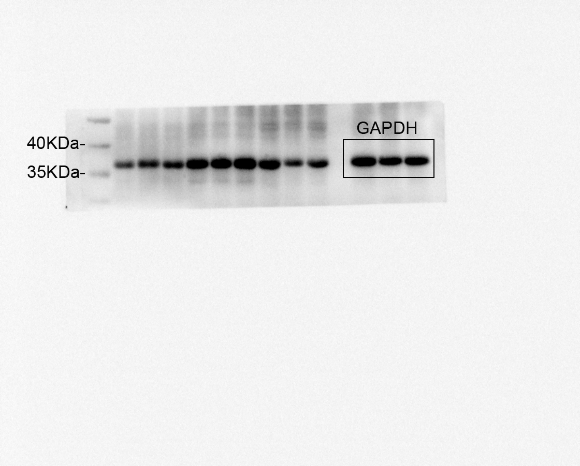

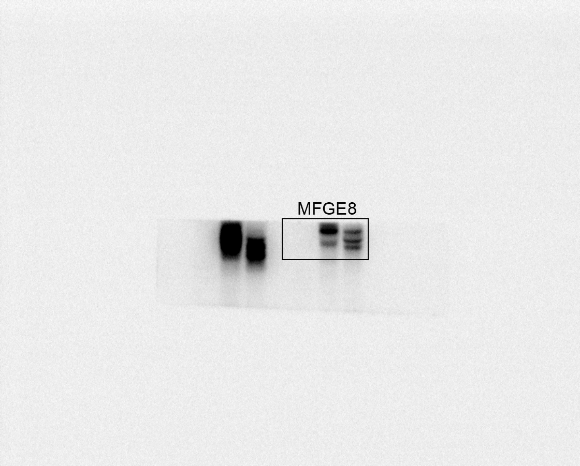

Supplement: Figure 4—source data 1. [file elife-95318-fig4-data1.zip › Figure4-Source data 1/Uncropped RT-PCR gels-Fig4C/Figure5-Source data 2/Figure5F.docx]

Figure 5F

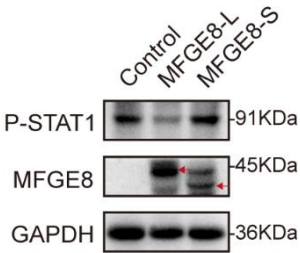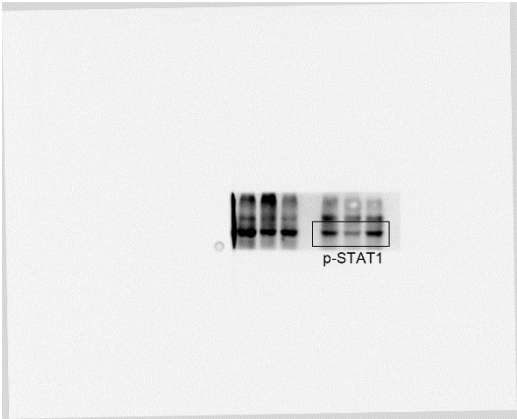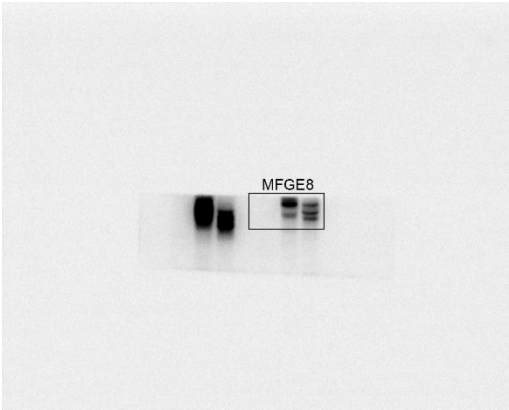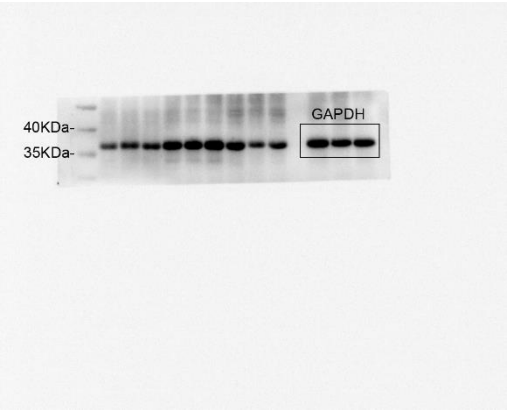

Supplement: Figure 4—source data 1. [file elife-95318-fig4-data1.zip › Figure4-Source data 1/Uncropped RT-PCR gels-Fig4C/Figure5-Source data 2/Figure5F.pdf]

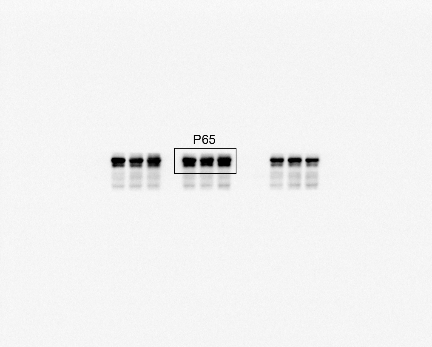

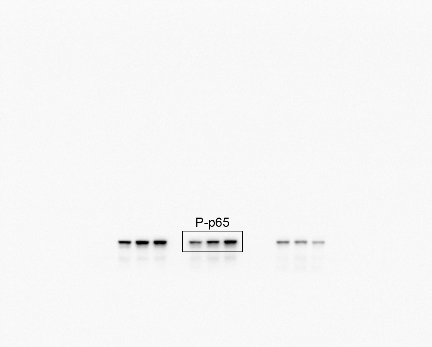

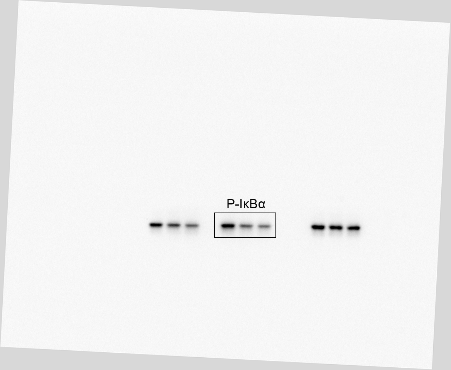

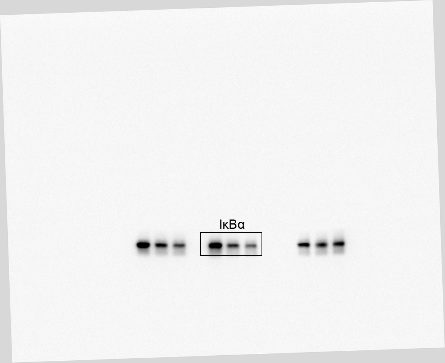

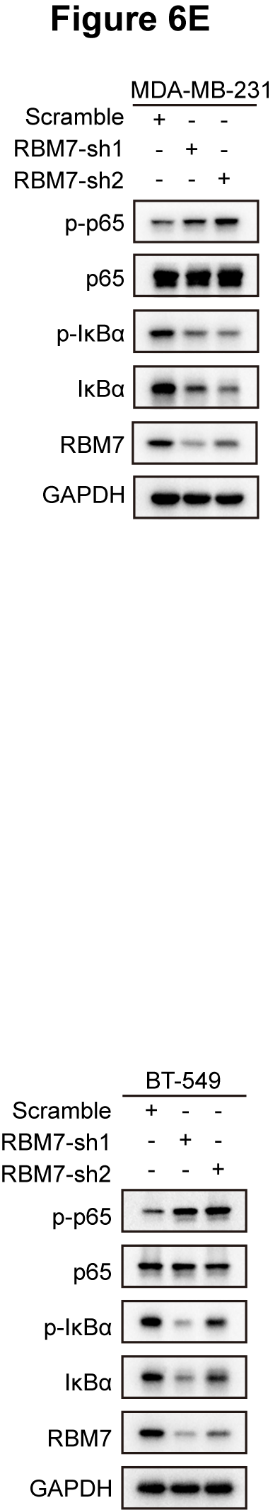


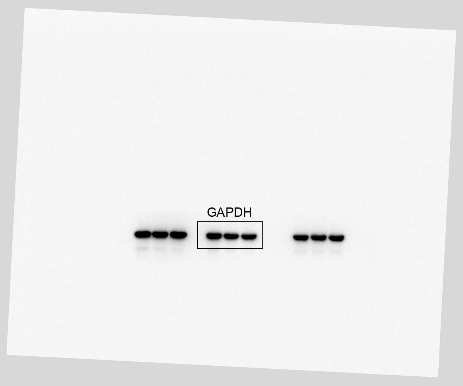











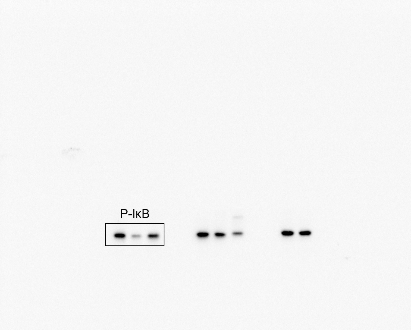

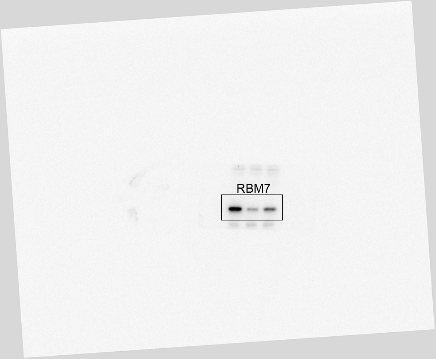



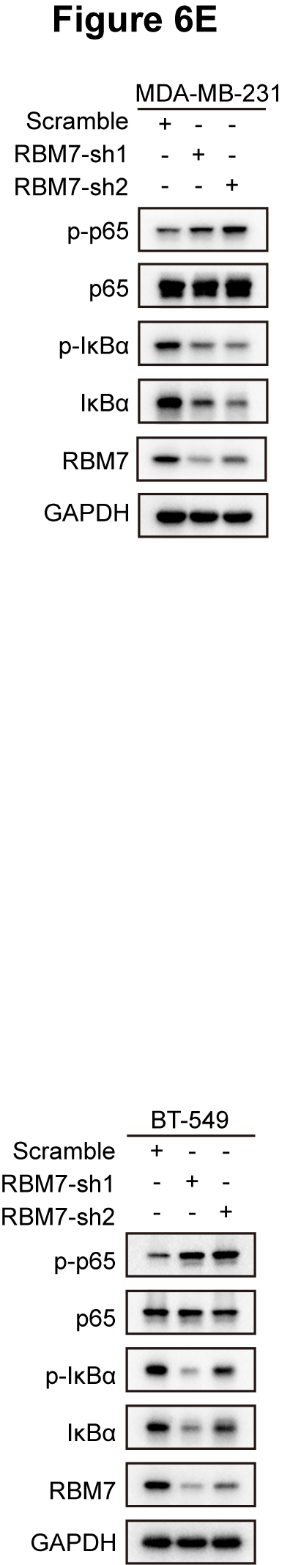

Supplement: Figure 4—source data 1. [file elife-95318-fig4-data1.zip › Figure4-Source data 1/Uncropped RT-PCR gels-Fig4C/Figure6-Source data 2/Figure 6E.docx]

Figure 6E

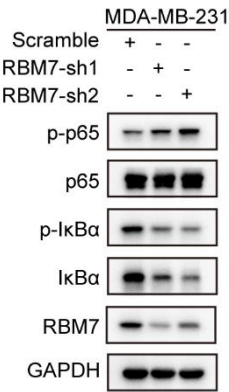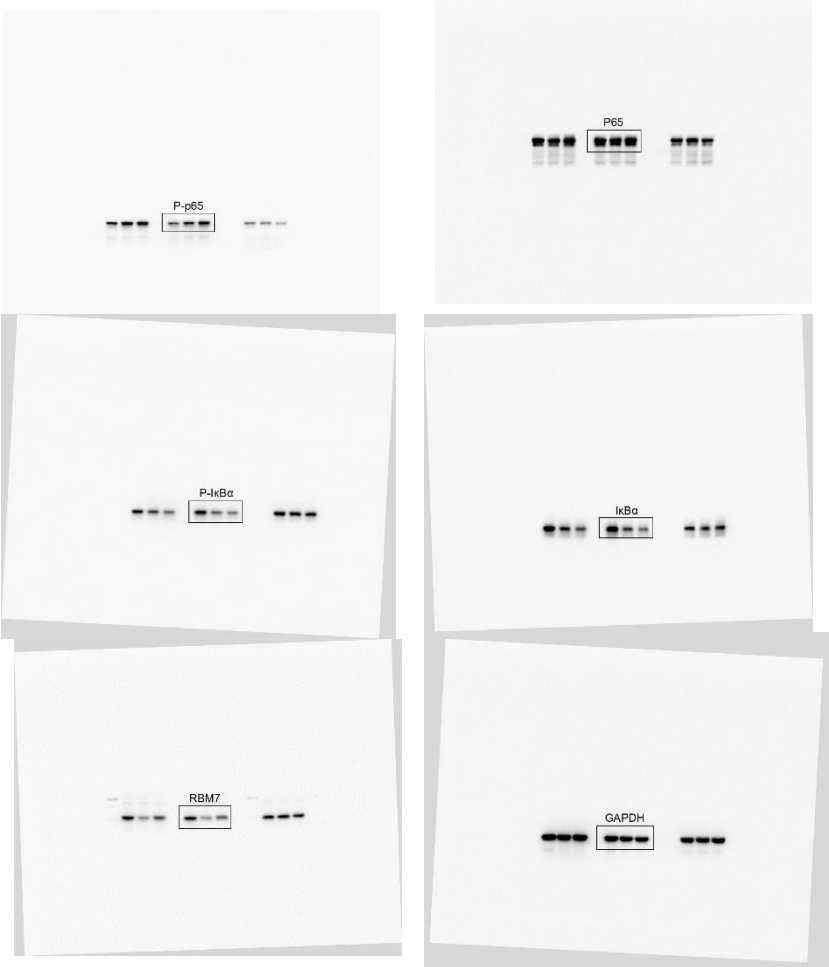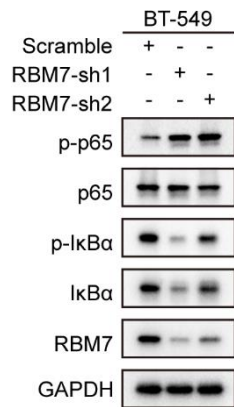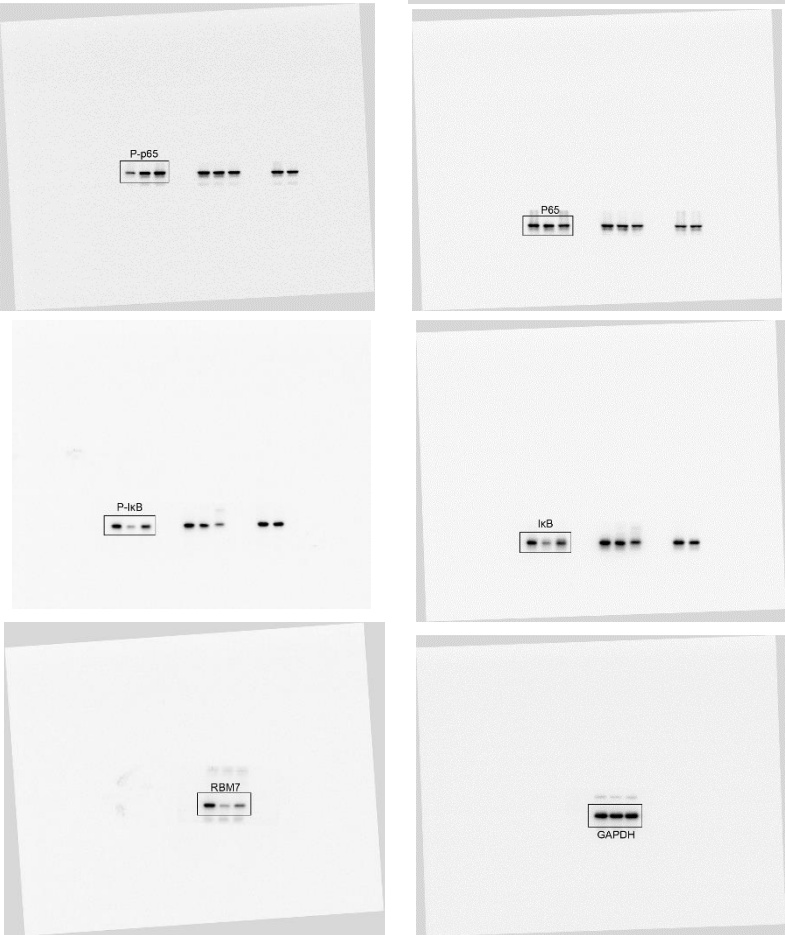

Supplement: Figure 4—source data 1. [file elife-95318-fig4-data1.zip › Figure4-Source data 1/Uncropped RT-PCR gels-Fig4C/Figure6-Source data 2/Figure 6E.pdf]

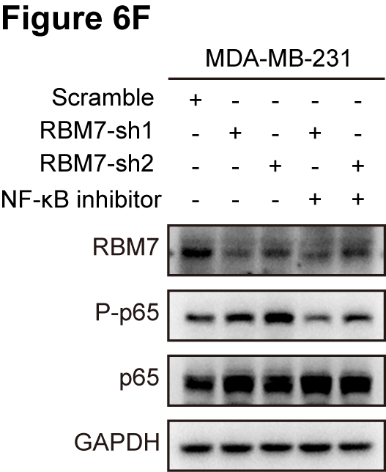

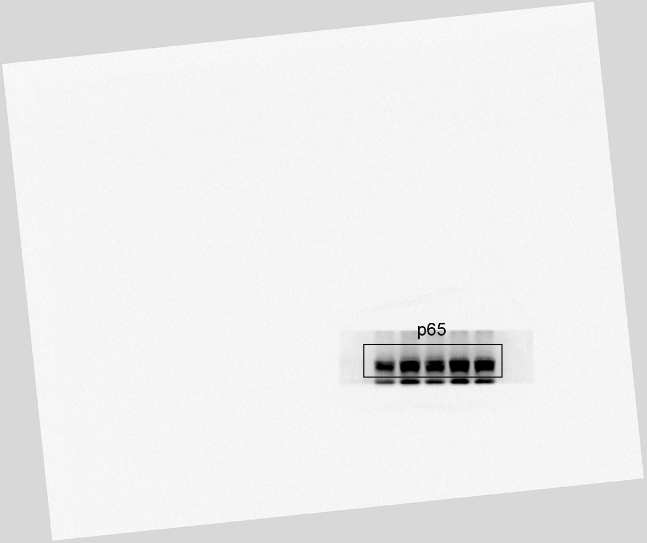

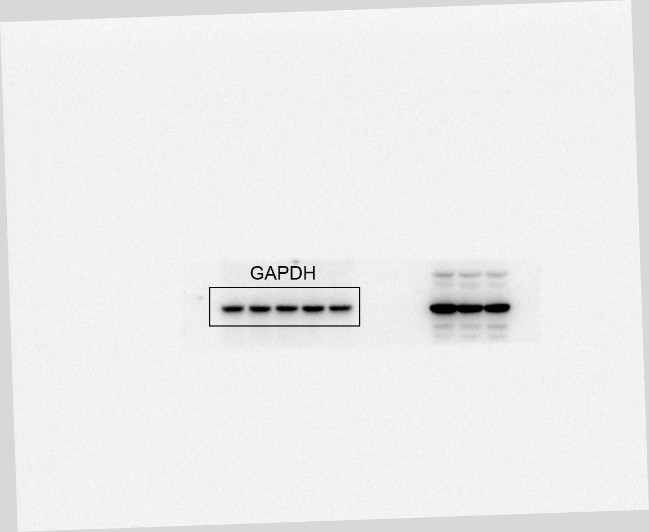

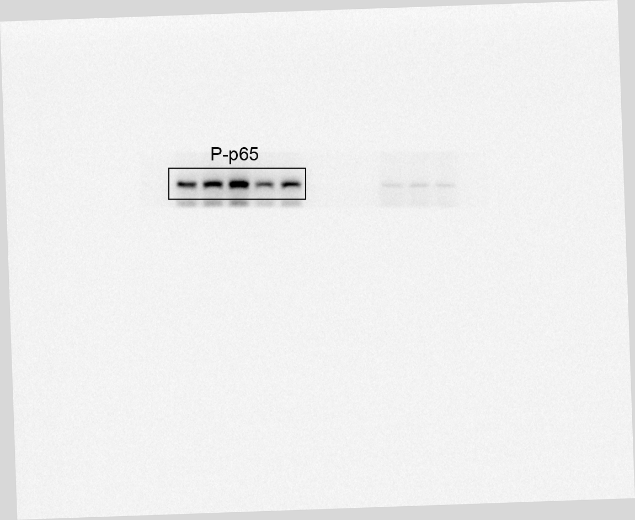

Supplement: Figure 4—source data 1. [file elife-95318-fig4-data1.zip › Figure4-Source data 1/Uncropped RT-PCR gels-Fig4C/Figure6-Source data 4/Figure6F.docx]

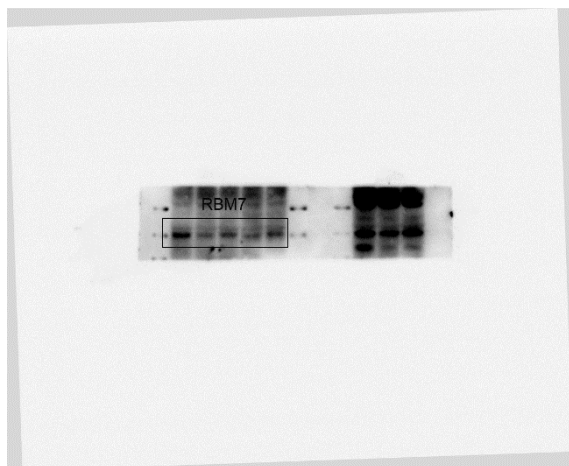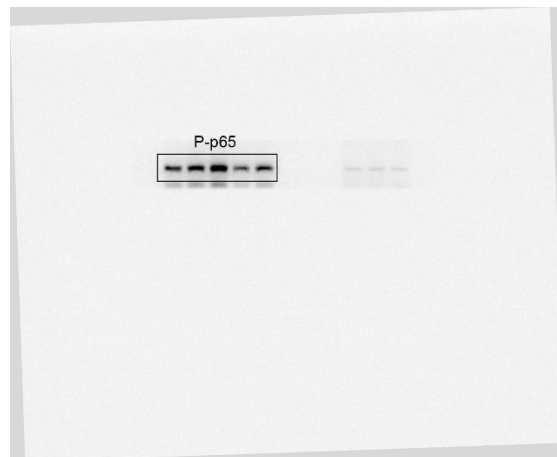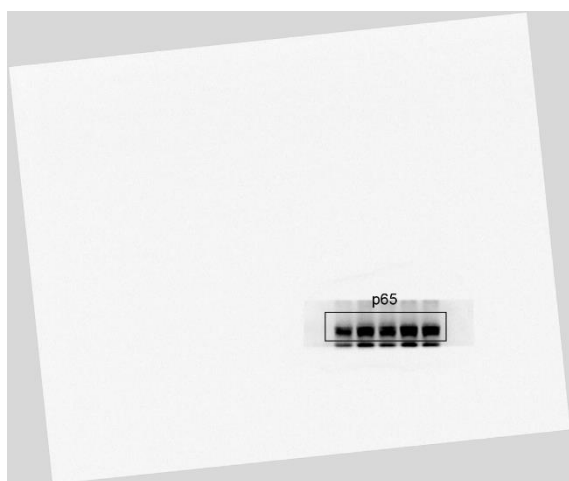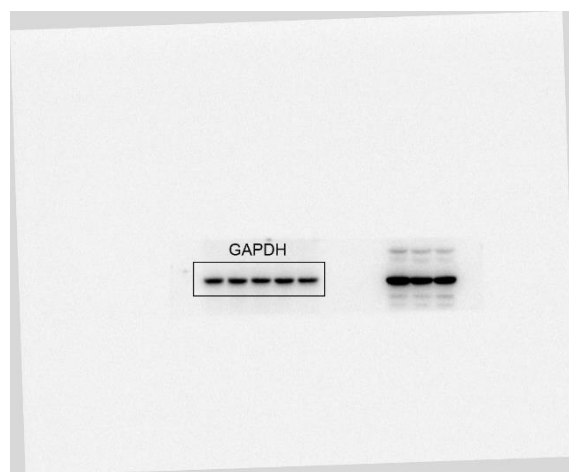

**Figure 6F**

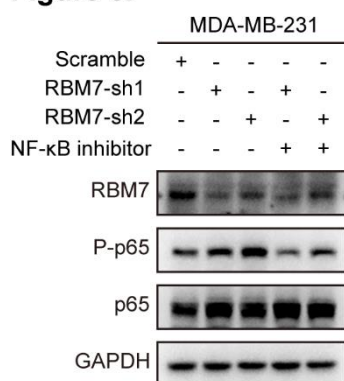

Supplement: Figure 4—source data 1. [file elife-95318-fig4-data1.zip › Figure4-Source data 1/Uncropped RT-PCR gels-Fig4C/Figure6-Source data 4/Figure6F.pdf]

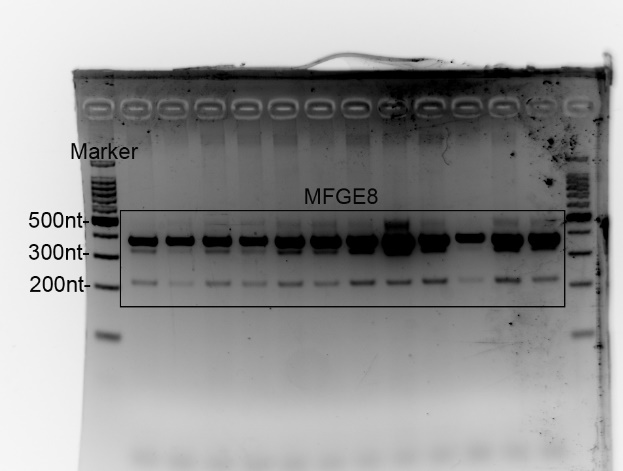

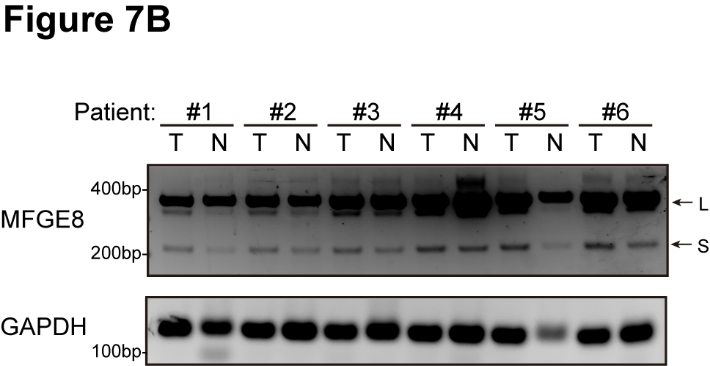

Supplement: Figure 4—source data 1. [file elife-95318-fig4-data1.zip › Figure4-Source data 1/Uncropped RT-PCR gels-Fig4C/Figure7-Source data 2/Figure7B.docx]

Figure 7B

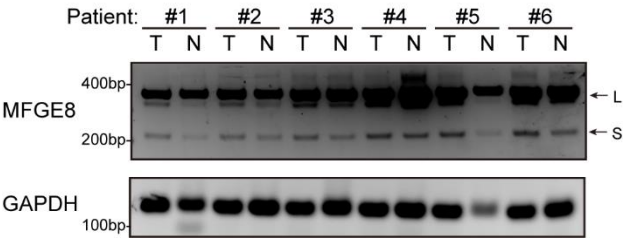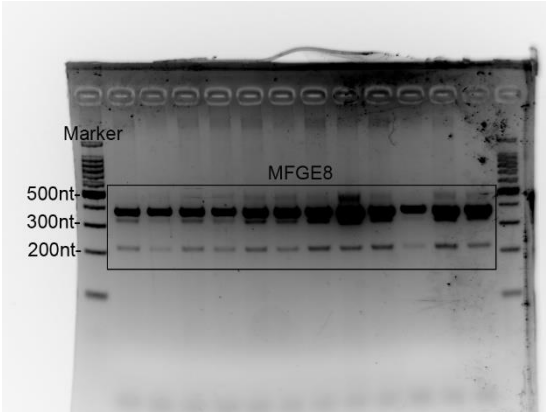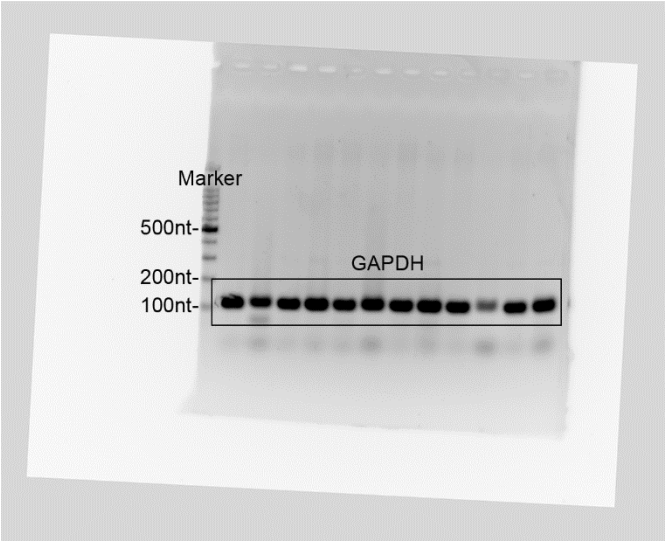

Supplement: Figure 4—source data 1. [file elife-95318-fig4-data1.zip › Figure4-Source data 1/Uncropped RT-PCR gels-Fig4C/Figure7-Source data 2/Figure7B.pdf]

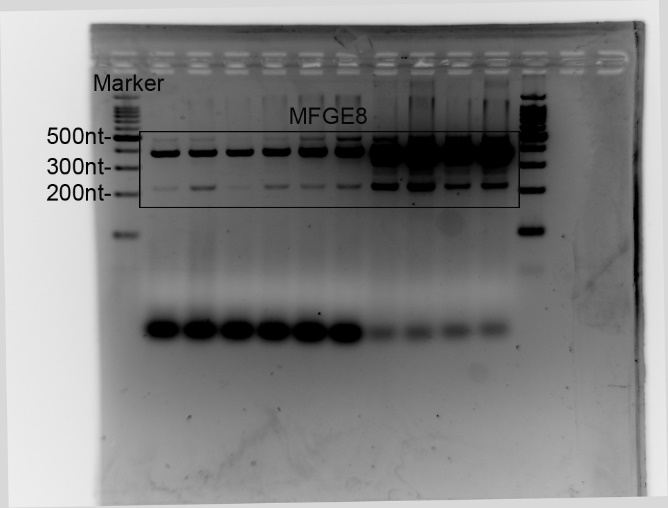

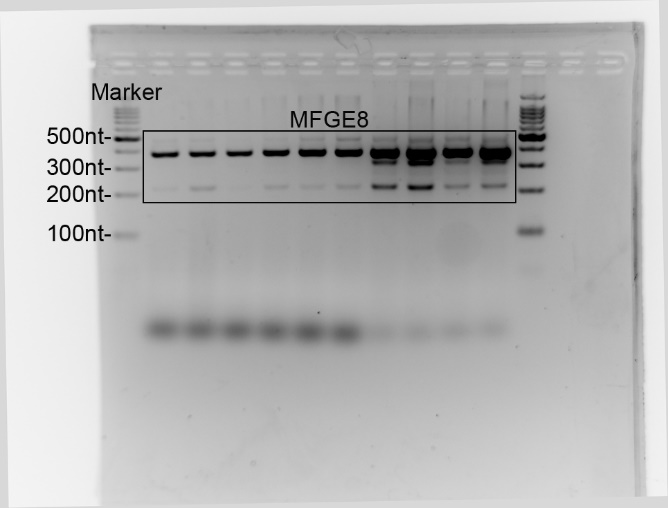

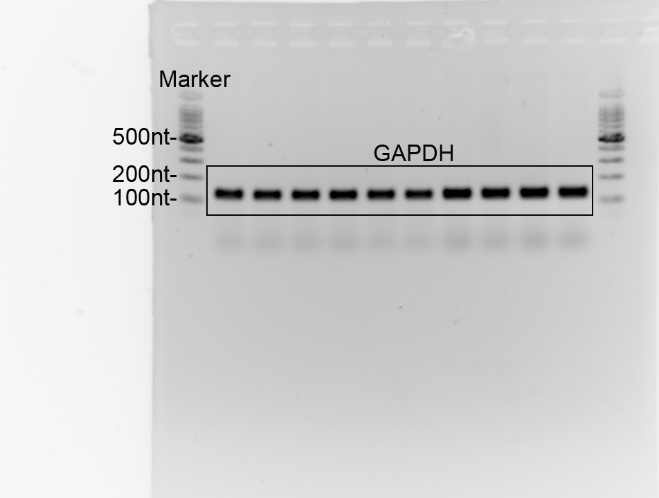

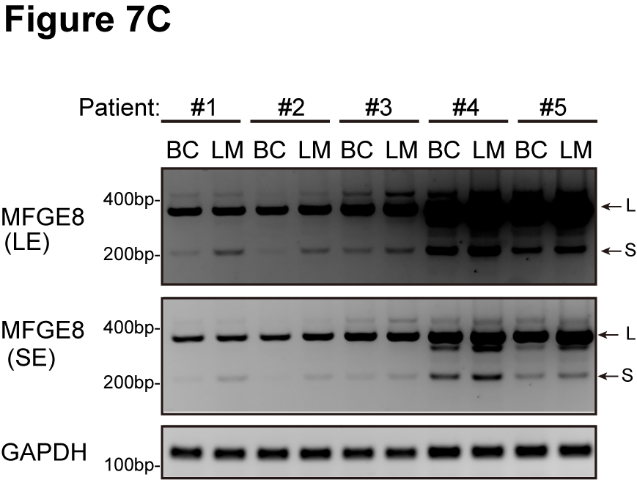

Supplement: Figure 4—source data 1. [file elife-95318-fig4-data1.zip › Figure4-Source data 1/Uncropped RT-PCR gels-Fig4C/Figure7-Source data 4/Figure7C.docx]

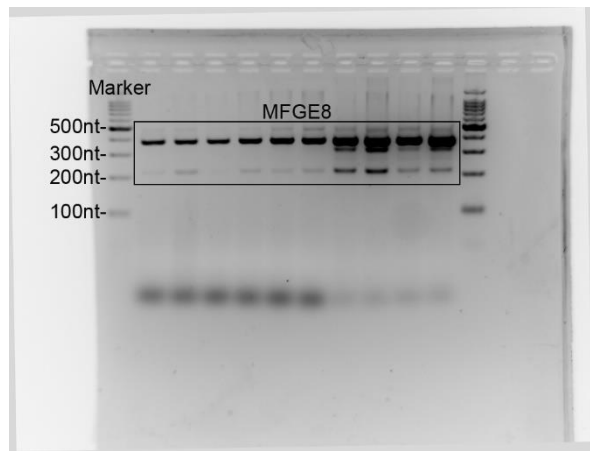

Supplement: Figure 4—source data 1. [file elife-95318-fig4-data1.zip › Figure4-Source data 1/Uncropped RT-PCR gels-Fig4C/Figure7-Source data 4/Figure7C.pdf]

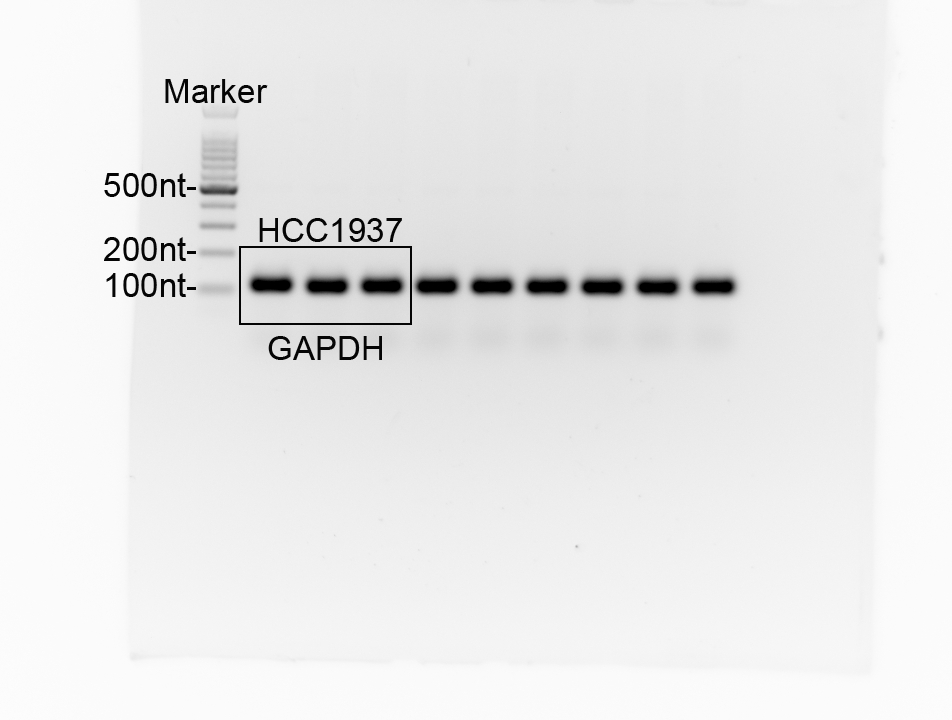

Supplement: Figure 4—source data 1. [file elife-95318-fig4-data1.zip › Figure4-Source data 1/Uncropped RT-PCR gels-Fig4C/HCC1937/HCC1937-GAPDH.tif]

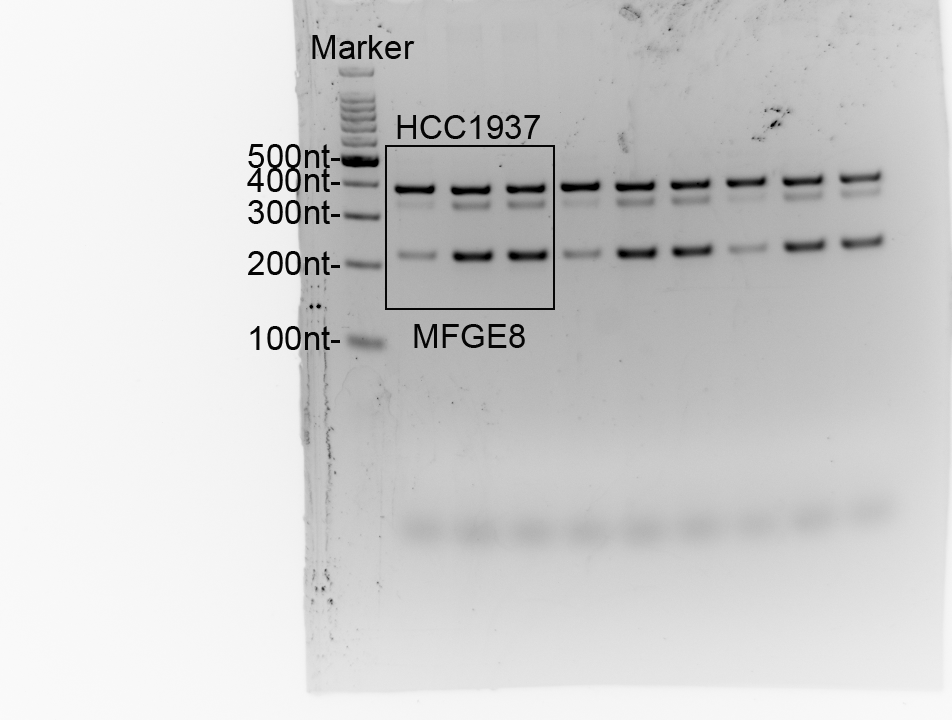

Supplement: Figure 4—source data 1. [file elife-95318-fig4-data1.zip › Figure4-Source data 1/Uncropped RT-PCR gels-Fig4C/HCC1937/HCC1937-MFGE8.tif]

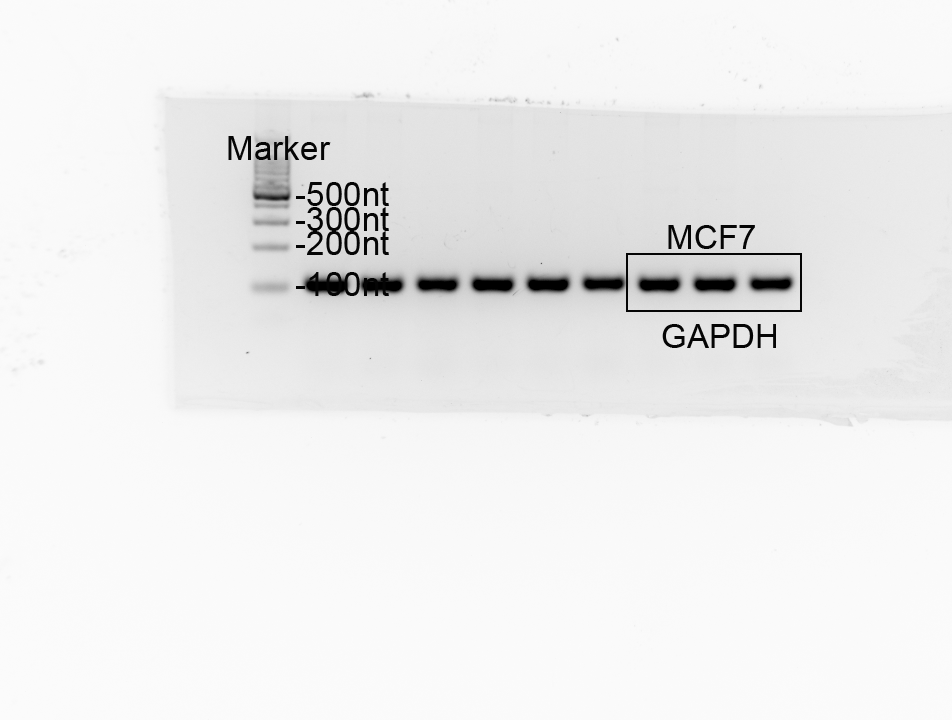

Supplement: Figure 4—source data 1. [file elife-95318-fig4-data1.zip › Figure4-Source data 1/Uncropped RT-PCR gels-Fig4C/MCF7/MCF7-GAPDH.tif]

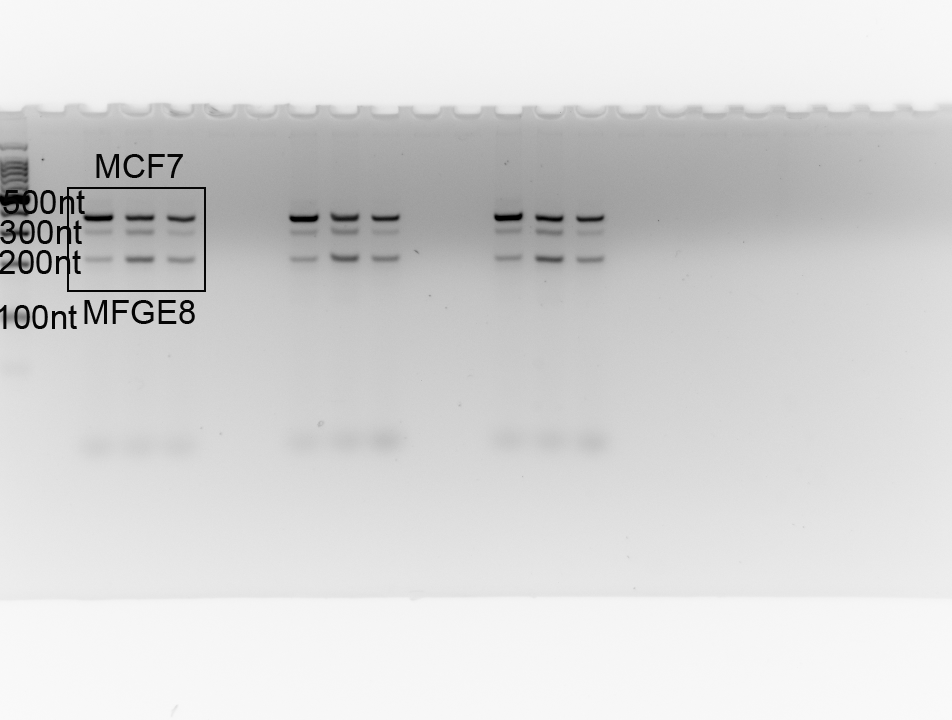

Supplement: Figure 4—source data 1. [file elife-95318-fig4-data1.zip › Figure4-Source data 1/Uncropped RT-PCR gels-Fig4C/MCF7/MCF7-MFGE8.tif]

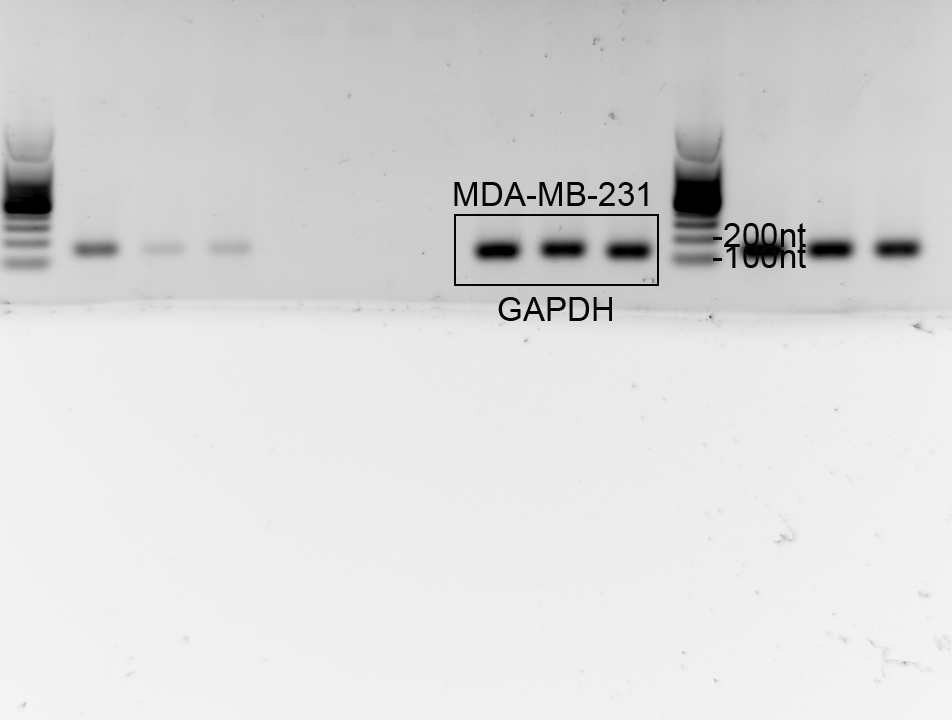

Supplement: Figure 4—source data 1. [file elife-95318-fig4-data1.zip › Figure4-Source data 1/Uncropped RT-PCR gels-Fig4C/MDA-MB-231/MDA-MB-231 GAPDH.tif]

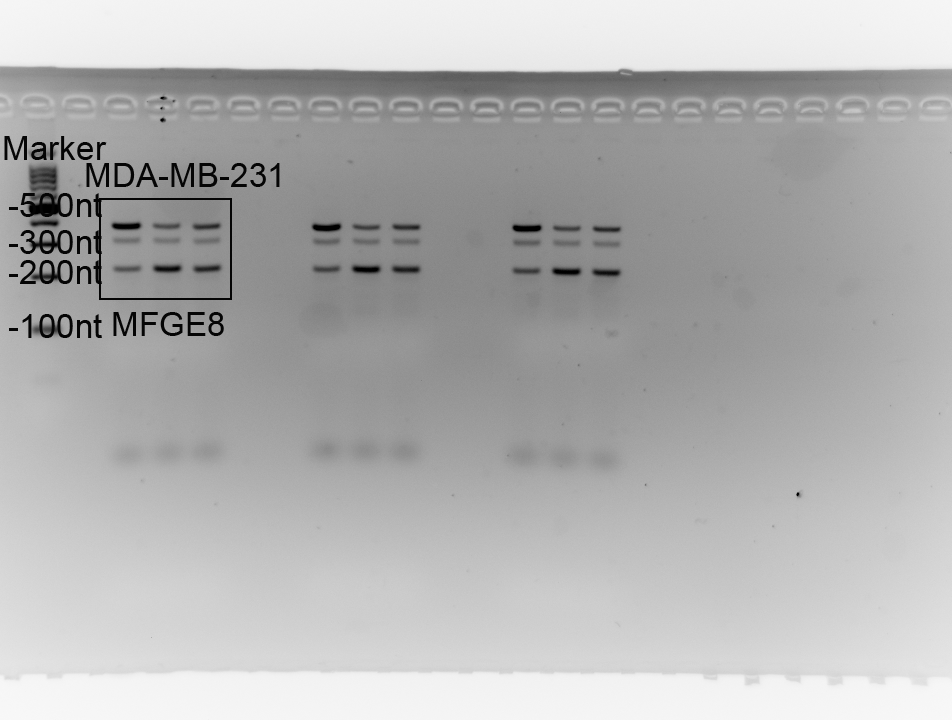

Supplement: Figure 4—source data 1. [file elife-95318-fig4-data1.zip › Figure4-Source data 1/Uncropped RT-PCR gels-Fig4C/MDA-MB-231/MDA-MB-231 MFGE8.tif]

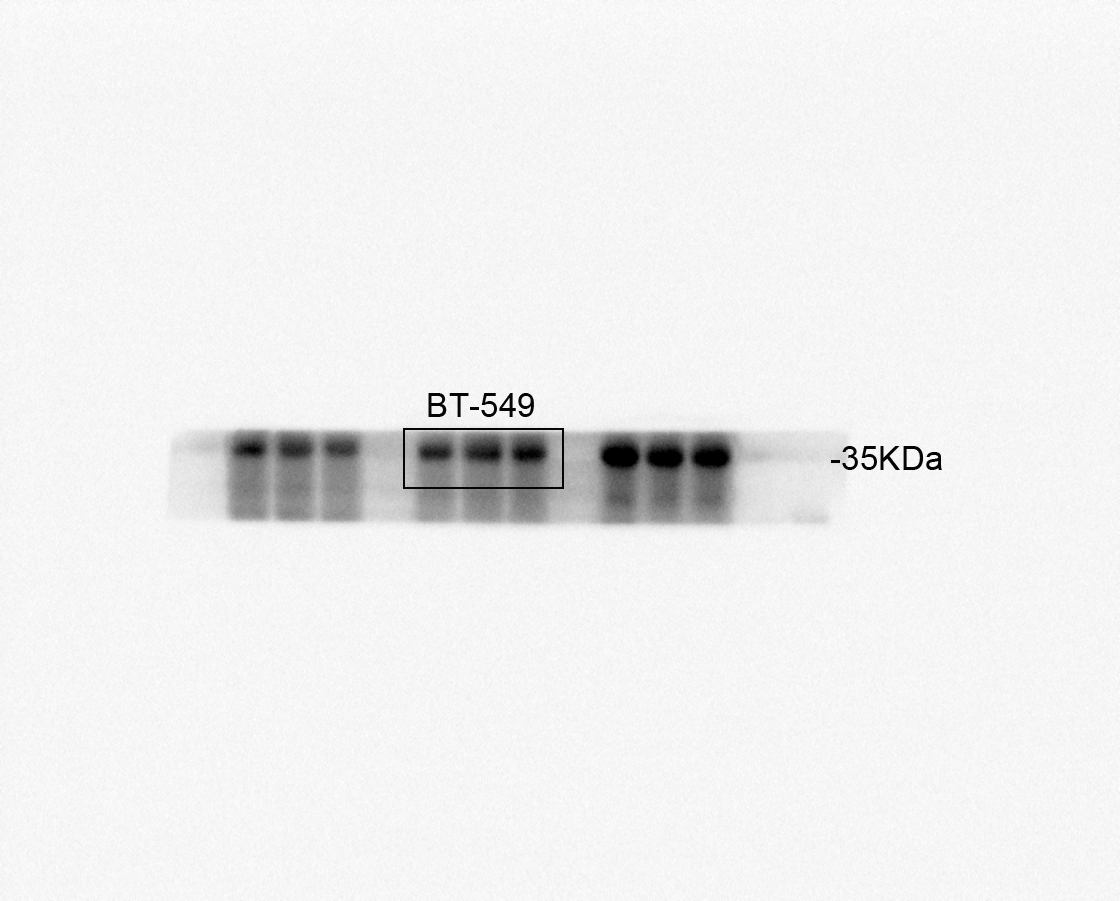

Supplement: Figure 4—source data 1. [file elife-95318-fig4-data1.zip › Figure4-Source data 1/Uncropped western blots-Fig4C/BT549/BT549 GAPDH.tif]

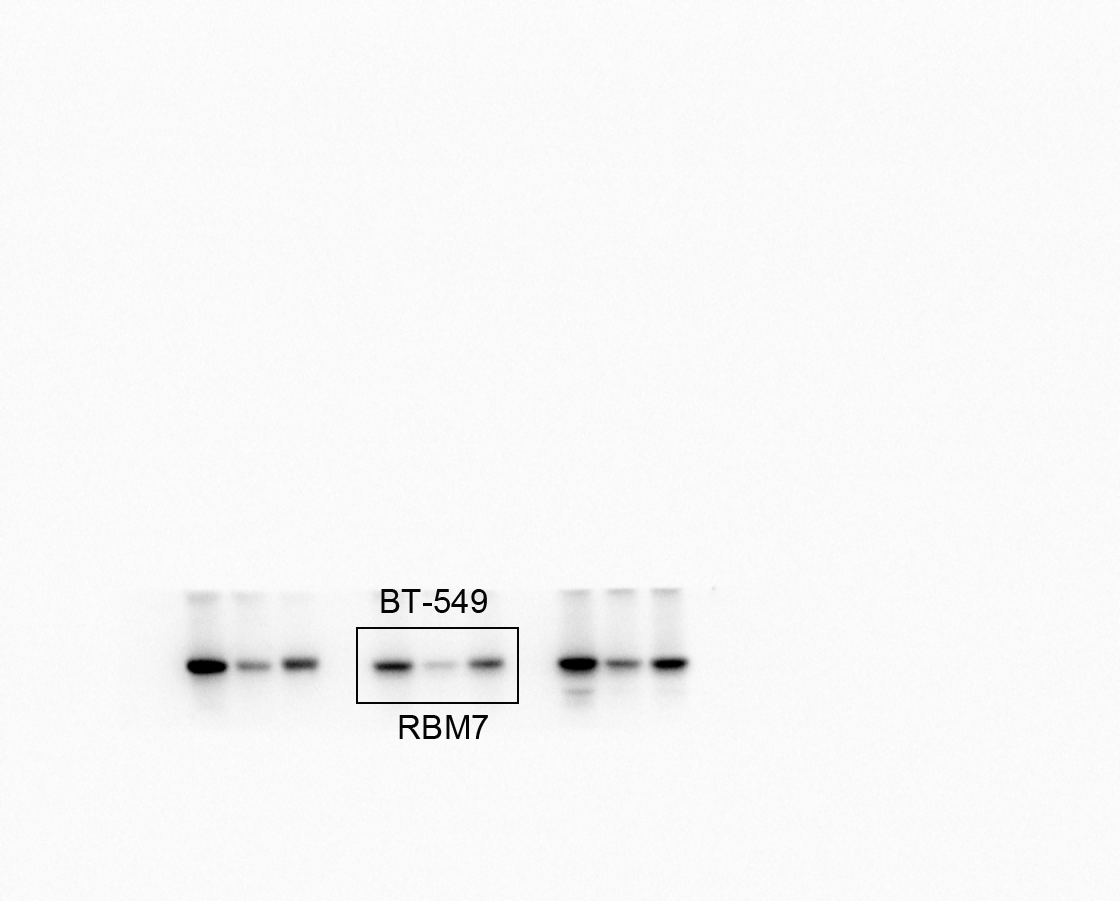

Supplement: Figure 4—source data 1. [file elife-95318-fig4-data1.zip › Figure4-Source data 1/Uncropped western blots-Fig4C/BT549/BT549 RBM7.tif]

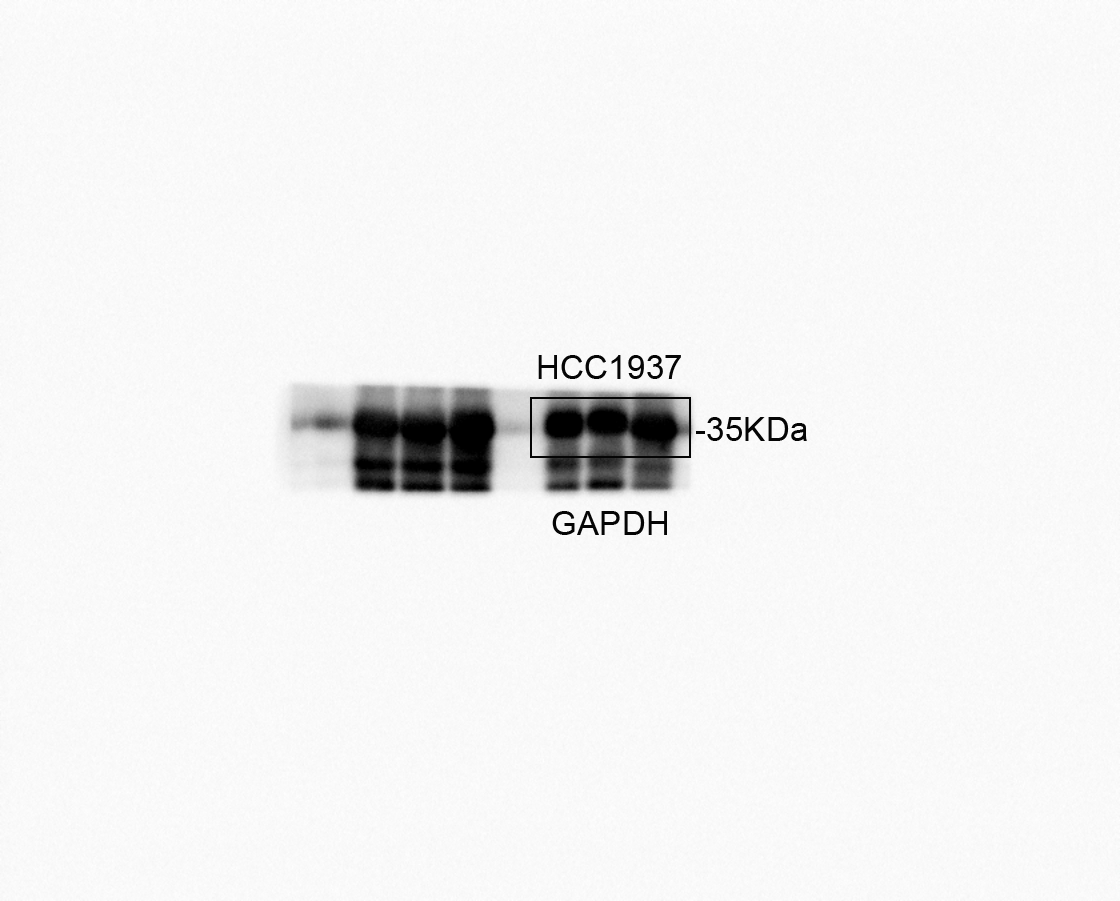

Supplement: Figure 4—source data 1. [file elife-95318-fig4-data1.zip › Figure4-Source data 1/Uncropped western blots-Fig4C/HCC1937/HCC1937 GAPDH.tif]

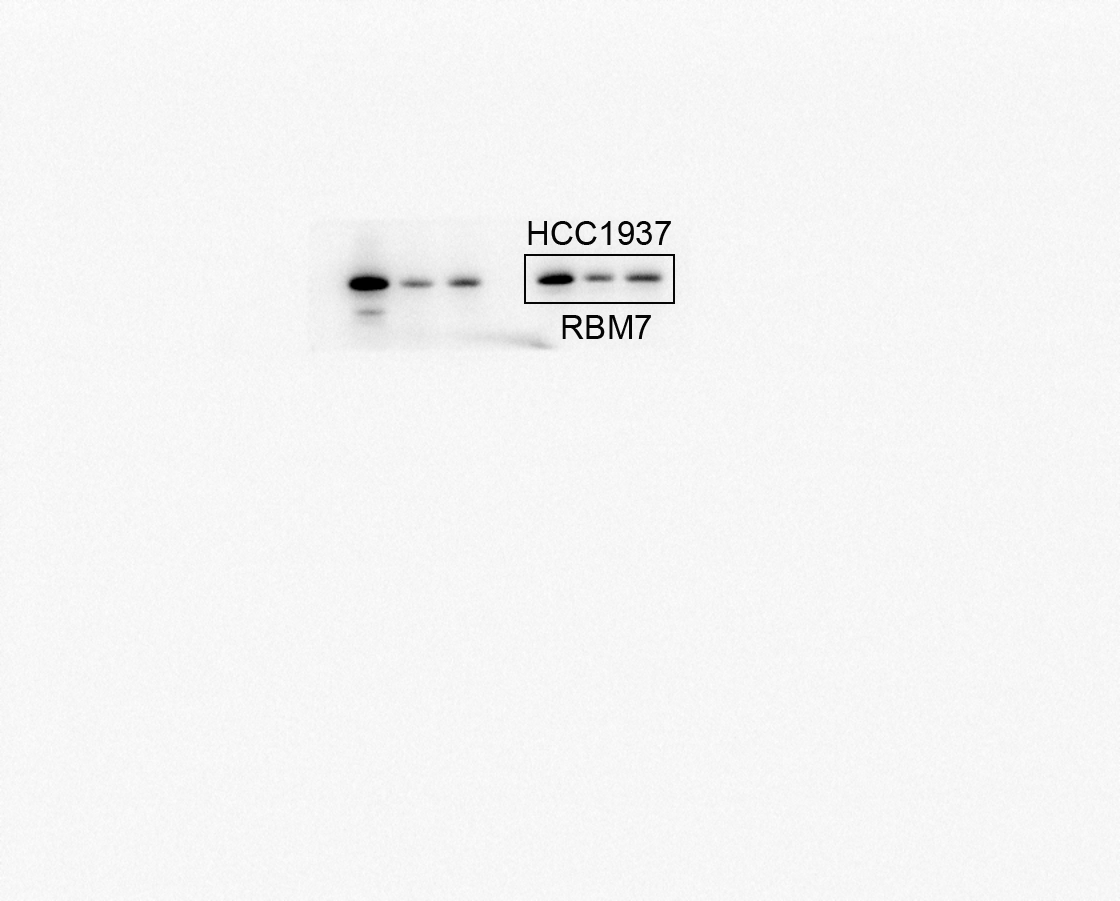

Supplement: Figure 4—source data 1. [file elife-95318-fig4-data1.zip › Figure4-Source data 1/Uncropped western blots-Fig4C/HCC1937/HCC1937 RBM7.tif]

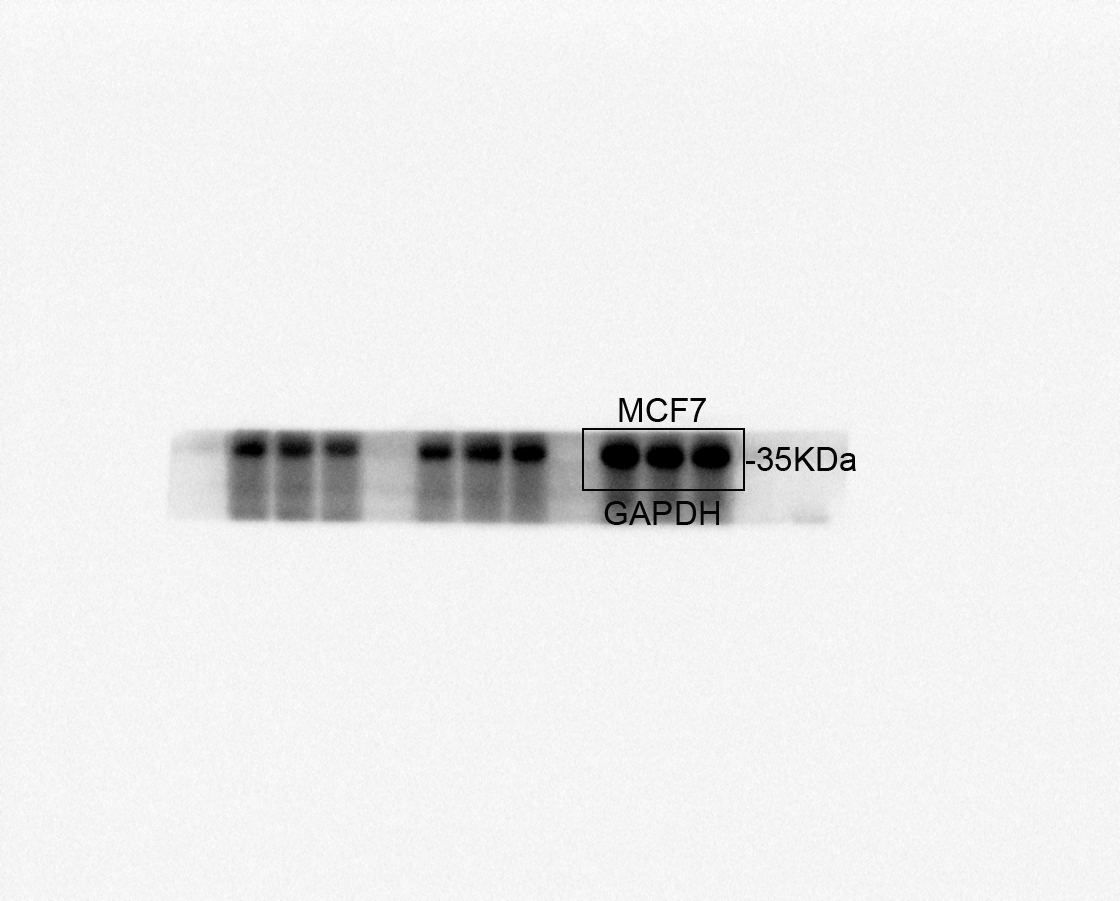

Supplement: Figure 4—source data 1. [file elife-95318-fig4-data1.zip › Figure4-Source data 1/Uncropped western blots-Fig4C/MCF7/MCF7 GAPDH.tif]

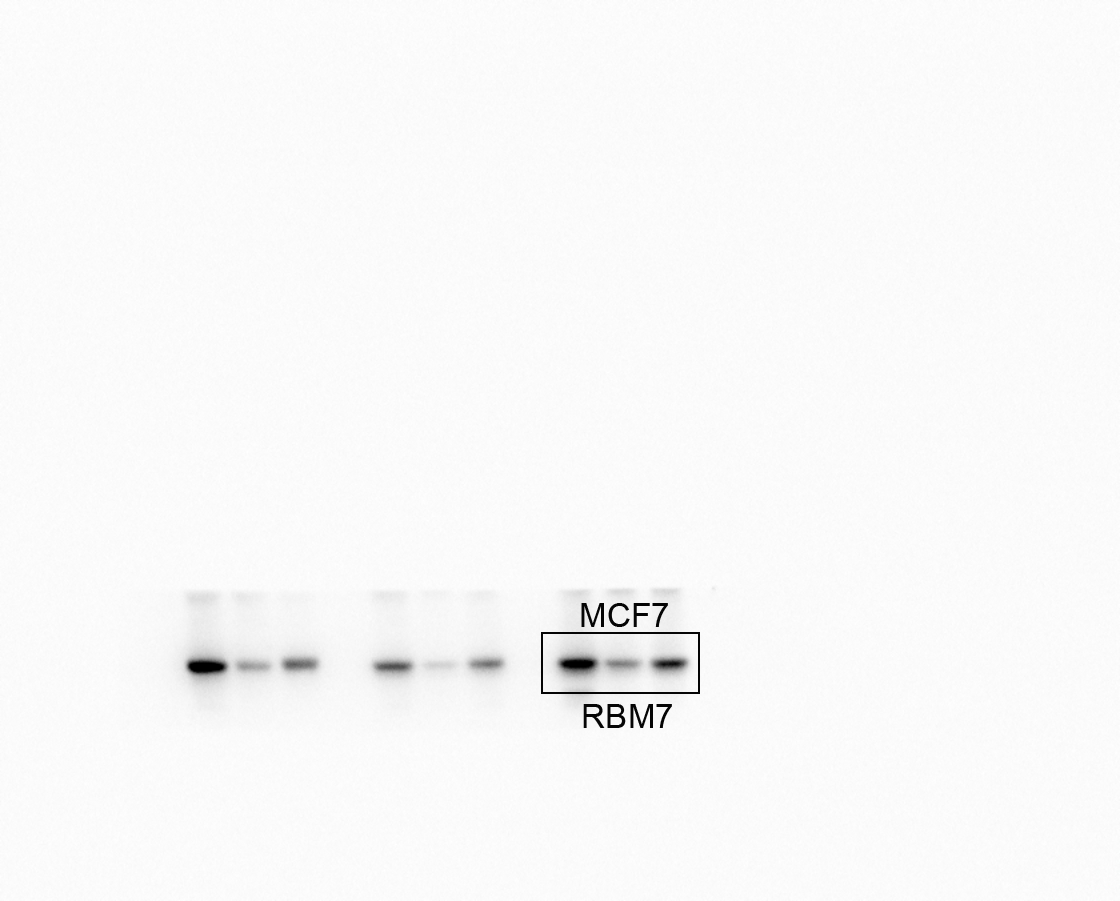

Supplement: Figure 4—source data 1. [file elife-95318-fig4-data1.zip › Figure4-Source data 1/Uncropped western blots-Fig4C/MCF7/MCF7 RBM7.tif]

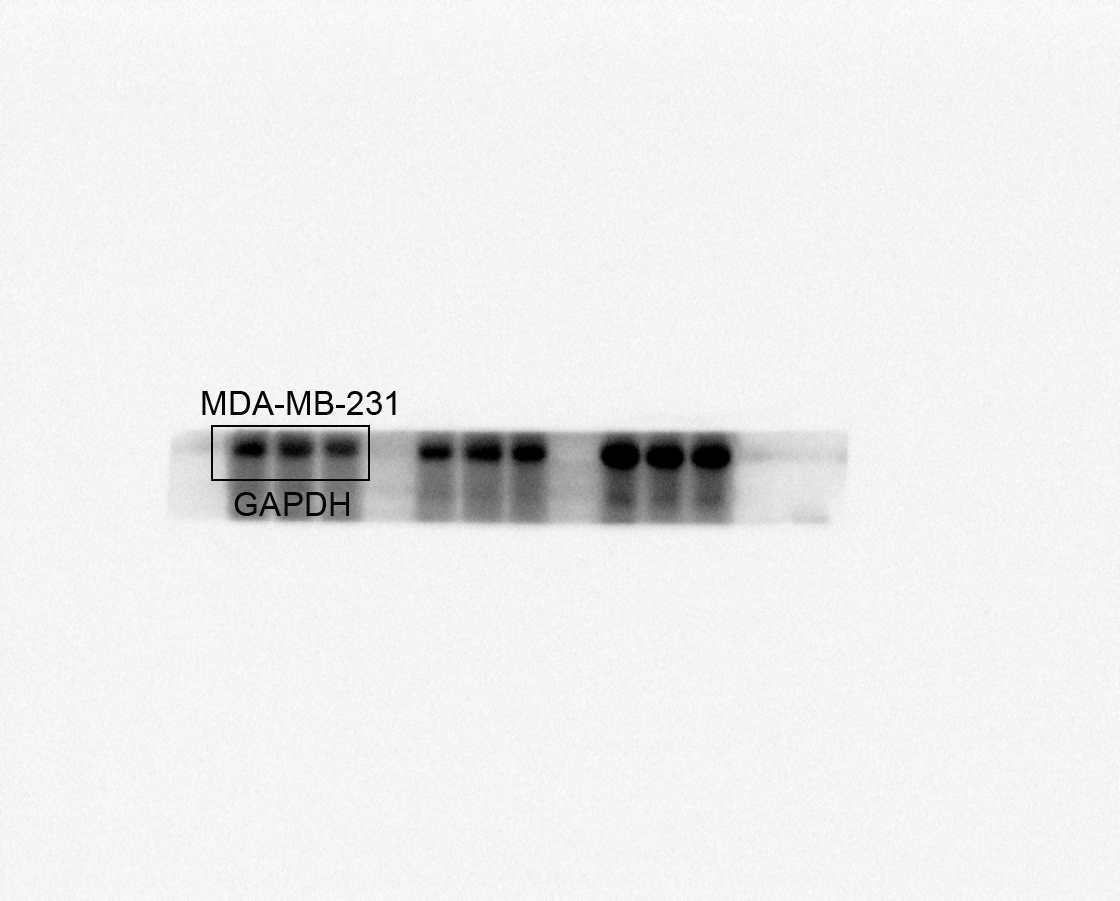

Supplement: Figure 4—source data 1. [file elife-95318-fig4-data1.zip › Figure4-Source data 1/Uncropped western blots-Fig4C/MDA-MB-231/MDA-MB-231 GAPDH.tif]

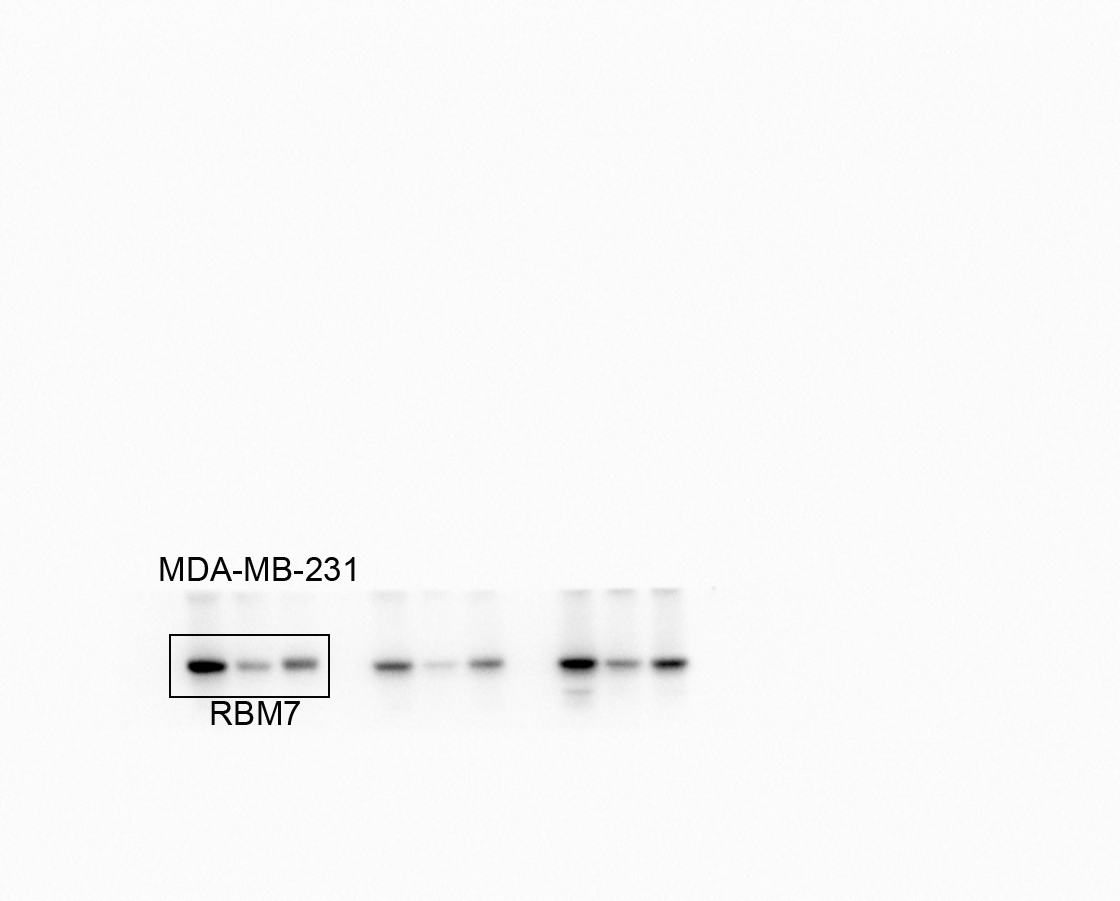

Supplement: Figure 4—source data 1. [file elife-95318-fig4-data1.zip › Figure4-Source data 1/Uncropped western blots-Fig4C/MDA-MB-231/MDA-MB-231 RBM7.tif]

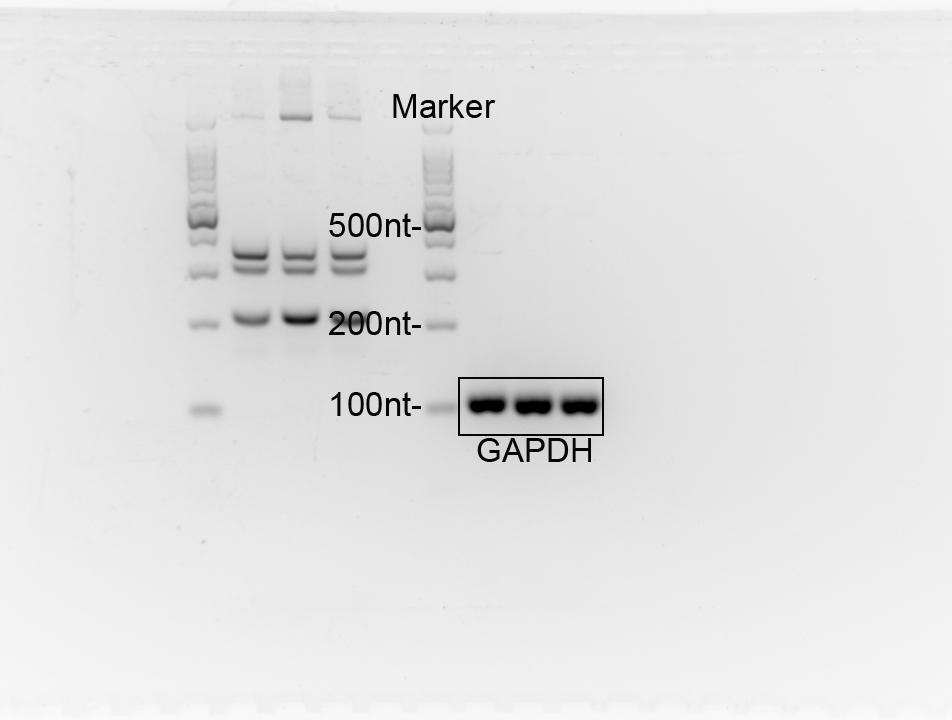

Supplement: Figure 4—source data 3. [file elife-95318-fig4-data3.zip › Figure4-Source data 3/Uncropped RT-PCR gels-Fig4D/GAPDH.tif]

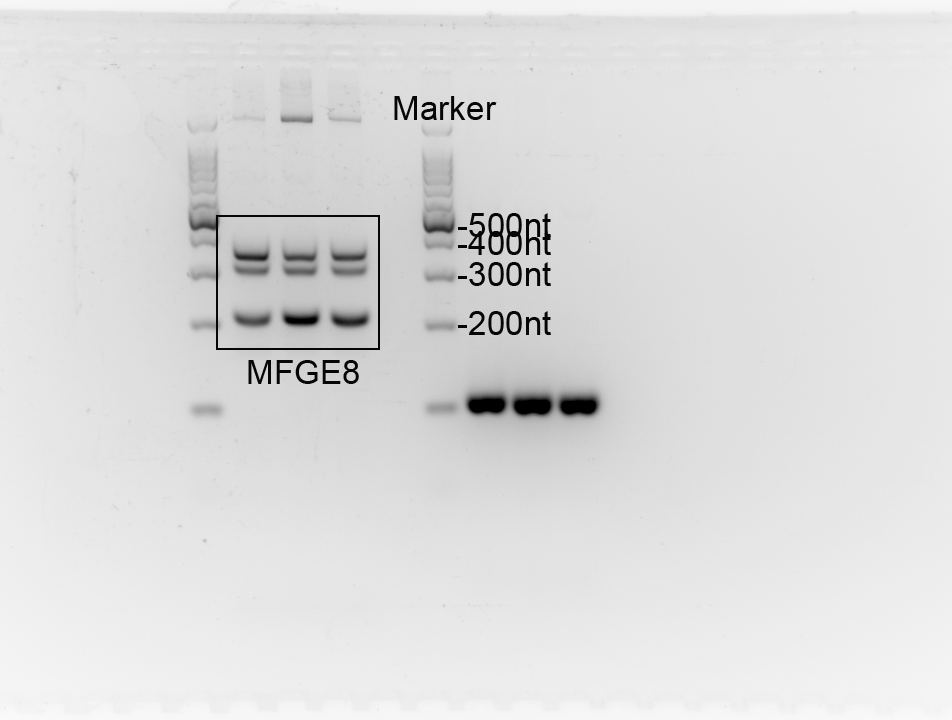

Supplement: Figure 4—source data 3. [file elife-95318-fig4-data3.zip › Figure4-Source data 3/Uncropped RT-PCR gels-Fig4D/MFGE8.tif]

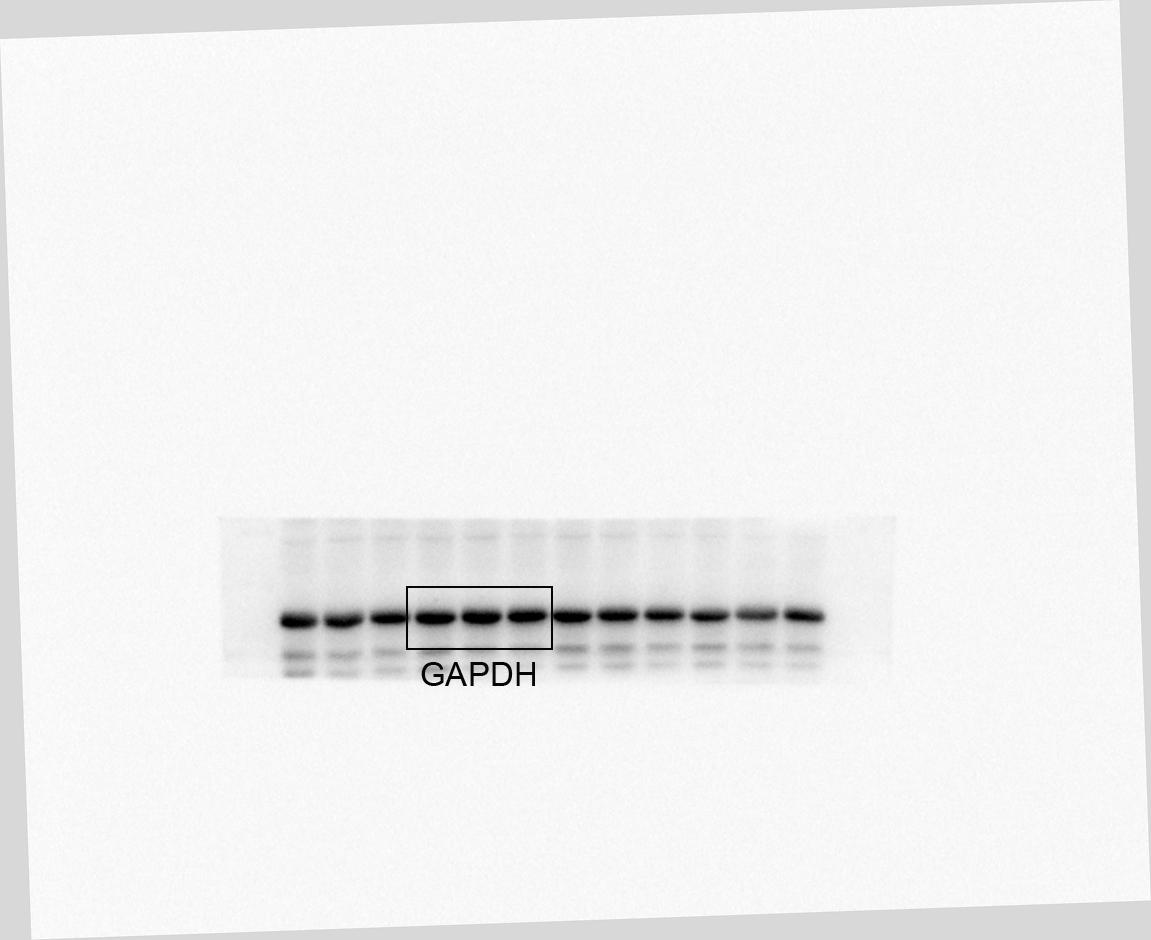

Supplement: Figure 4—source data 3. [file elife-95318-fig4-data3.zip › Figure4-Source data 3/Uncropped western blots-Fig4D/GAPDH.tif]

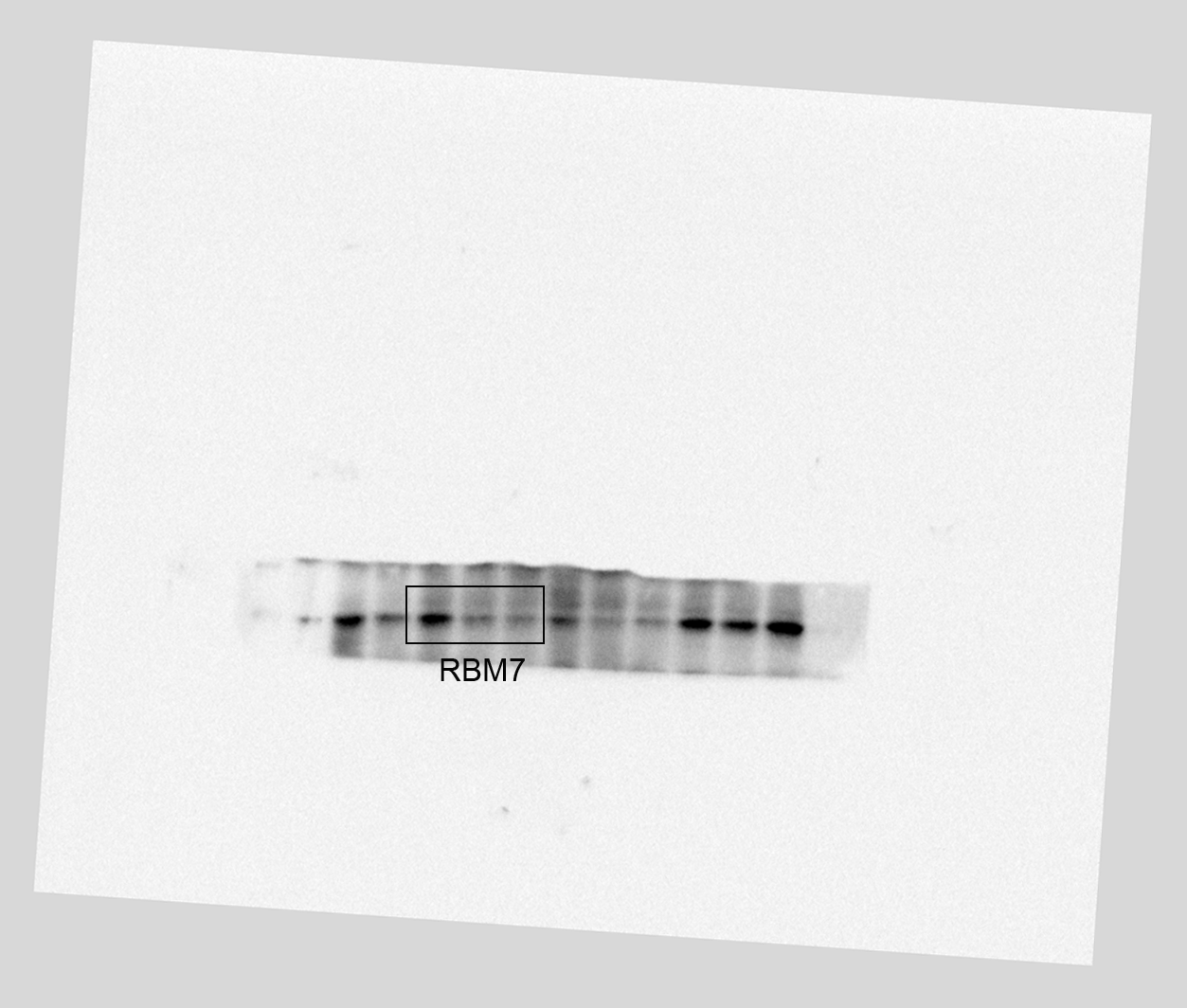

Supplement: Figure 4—source data 3. [file elife-95318-fig4-data3.zip › Figure4-Source data 3/Uncropped western blots-Fig4D/RBM7.tif]

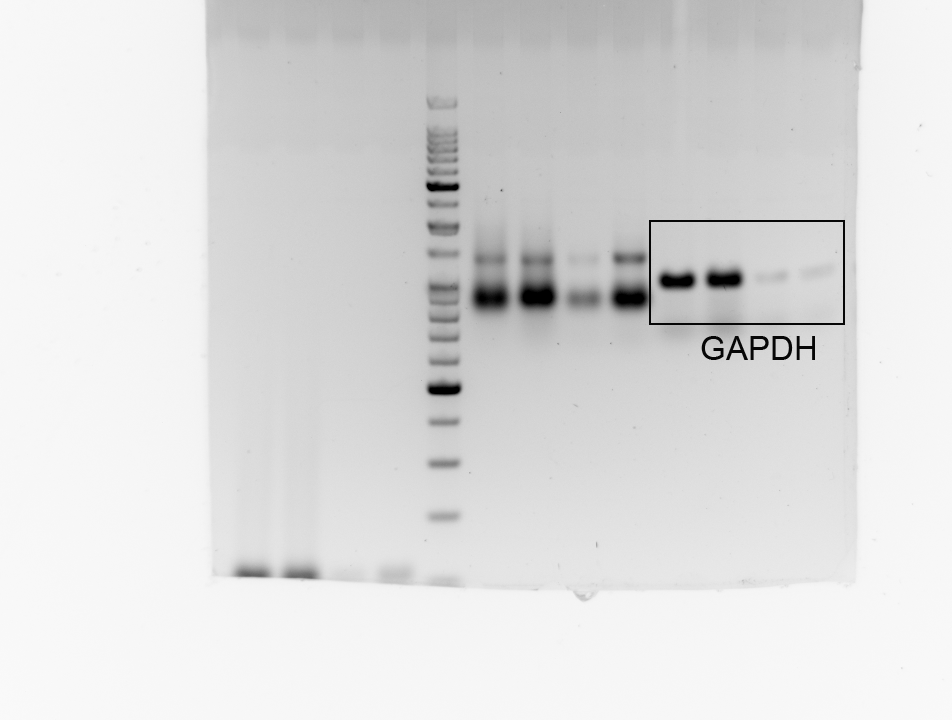

Supplement: Figure 4—source data 5. [file elife-95318-fig4-data5.zip › Figure4-Source data 5/Uncropped RT-PCR gels-Fig4E/GAPDH.tif]

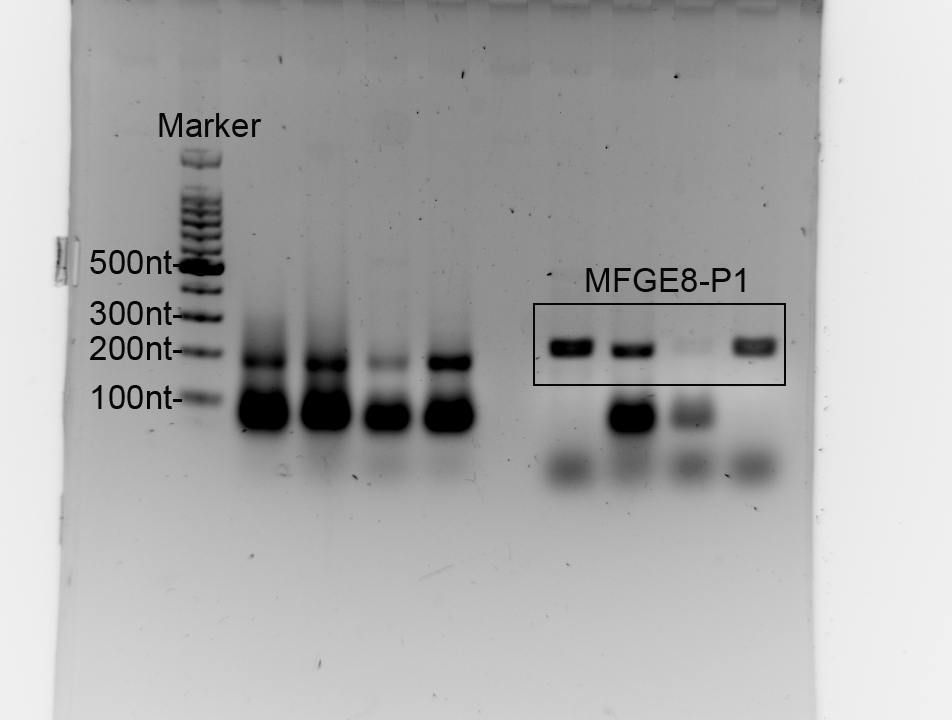

Supplement: Figure 4—source data 5. [file elife-95318-fig4-data5.zip › Figure4-Source data 5/Uncropped RT-PCR gels-Fig4E/MFGE8-P1.tif]

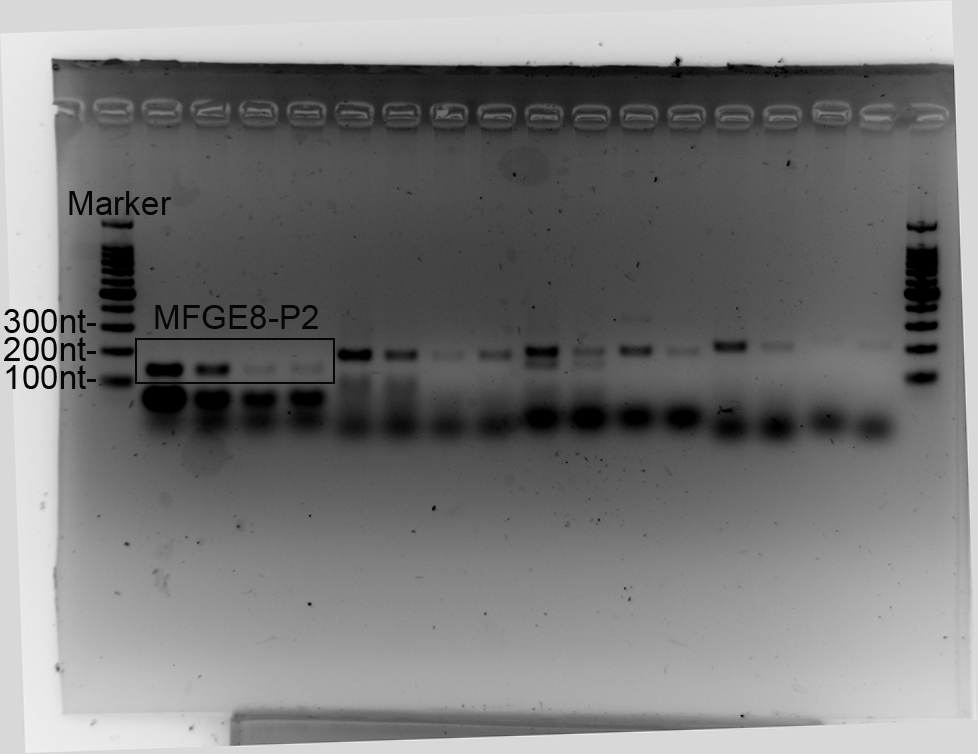

Supplement: Figure 4—source data 5. [file elife-95318-fig4-data5.zip › Figure4-Source data 5/Uncropped RT-PCR gels-Fig4E/MFGE8-P2.tif]

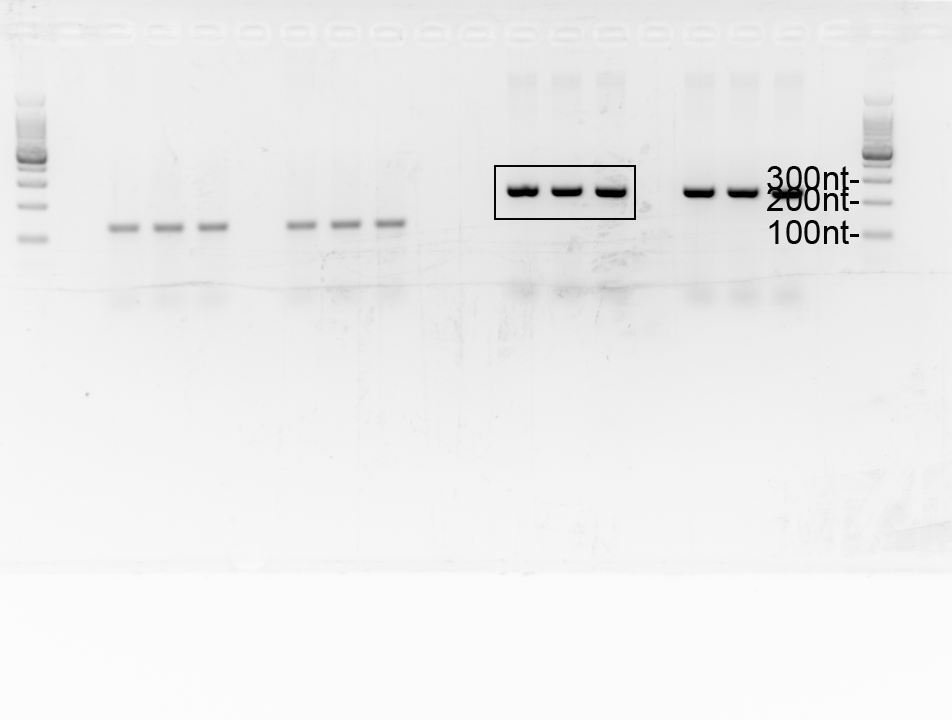

Supplement: Figure 4—source data 7. [file elife-95318-fig4-data7.zip › Figure4-Source data 7/Uncropped RT-PCR gels-Fig4F/BT549-GAPDH.tif]

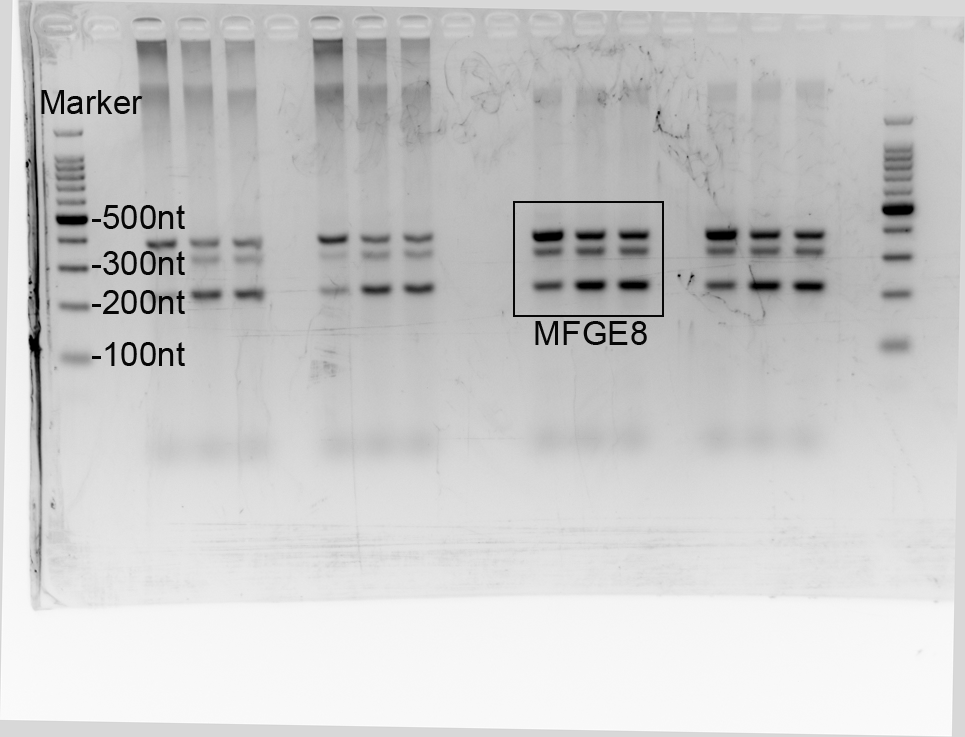

Supplement: Figure 4—source data 7. [file elife-95318-fig4-data7.zip › Figure4-Source data 7/Uncropped RT-PCR gels-Fig4F/BT549-MFGE8.tif]

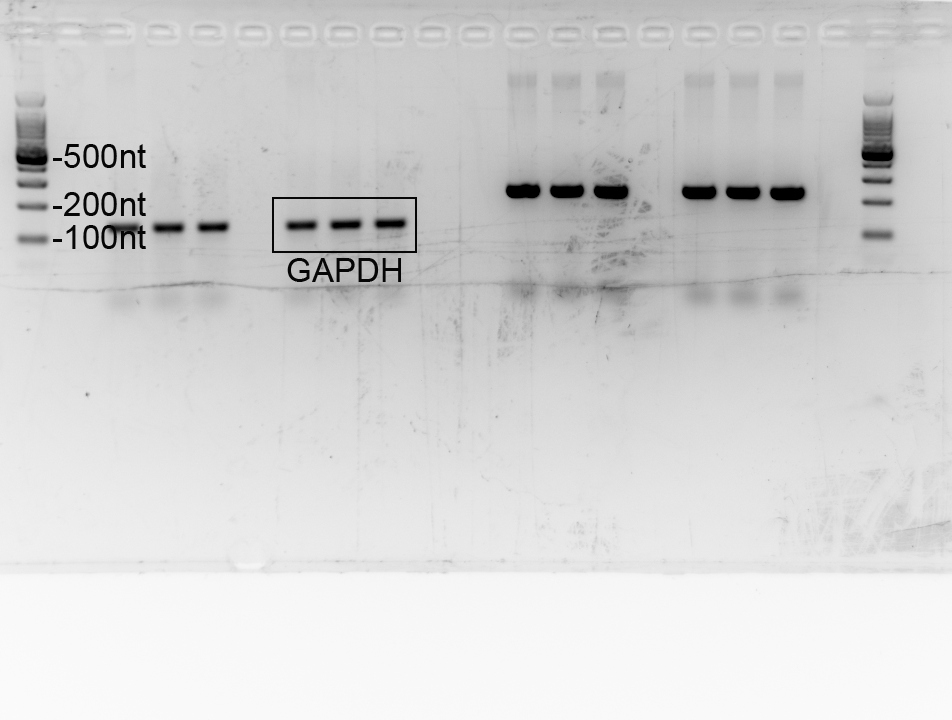

Supplement: Figure 4—source data 7. [file elife-95318-fig4-data7.zip › Figure4-Source data 7/Uncropped RT-PCR gels-Fig4F/MCF7 GAPDH.tif]

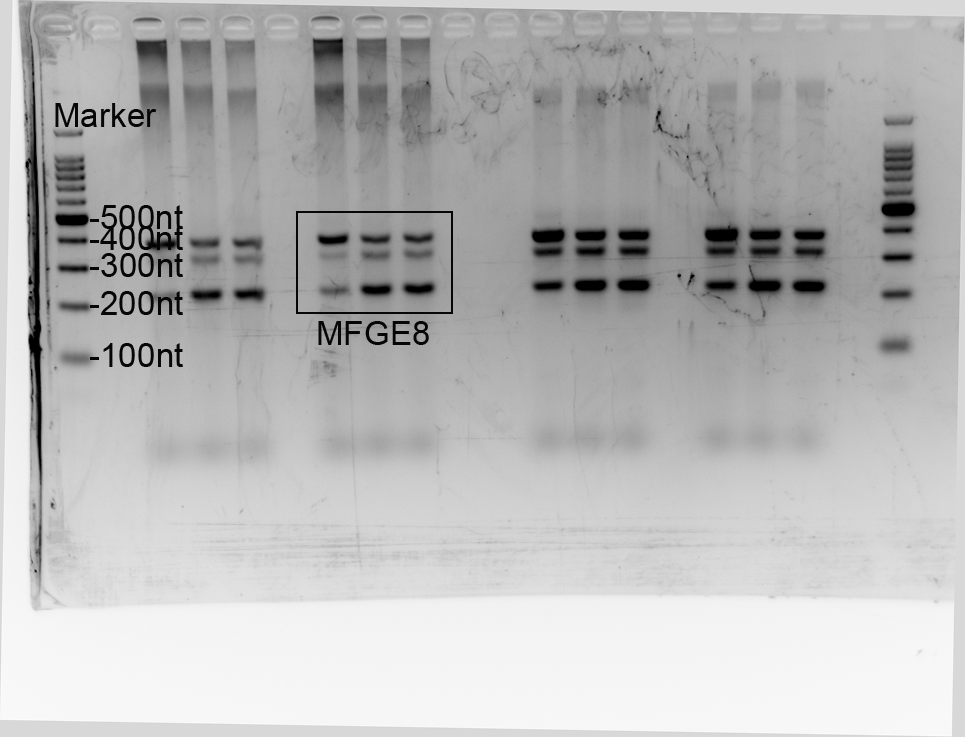

Supplement: Figure 4—source data 7. [file elife-95318-fig4-data7.zip › Figure4-Source data 7/Uncropped RT-PCR gels-Fig4F/MCF7-MFGE8.tif]

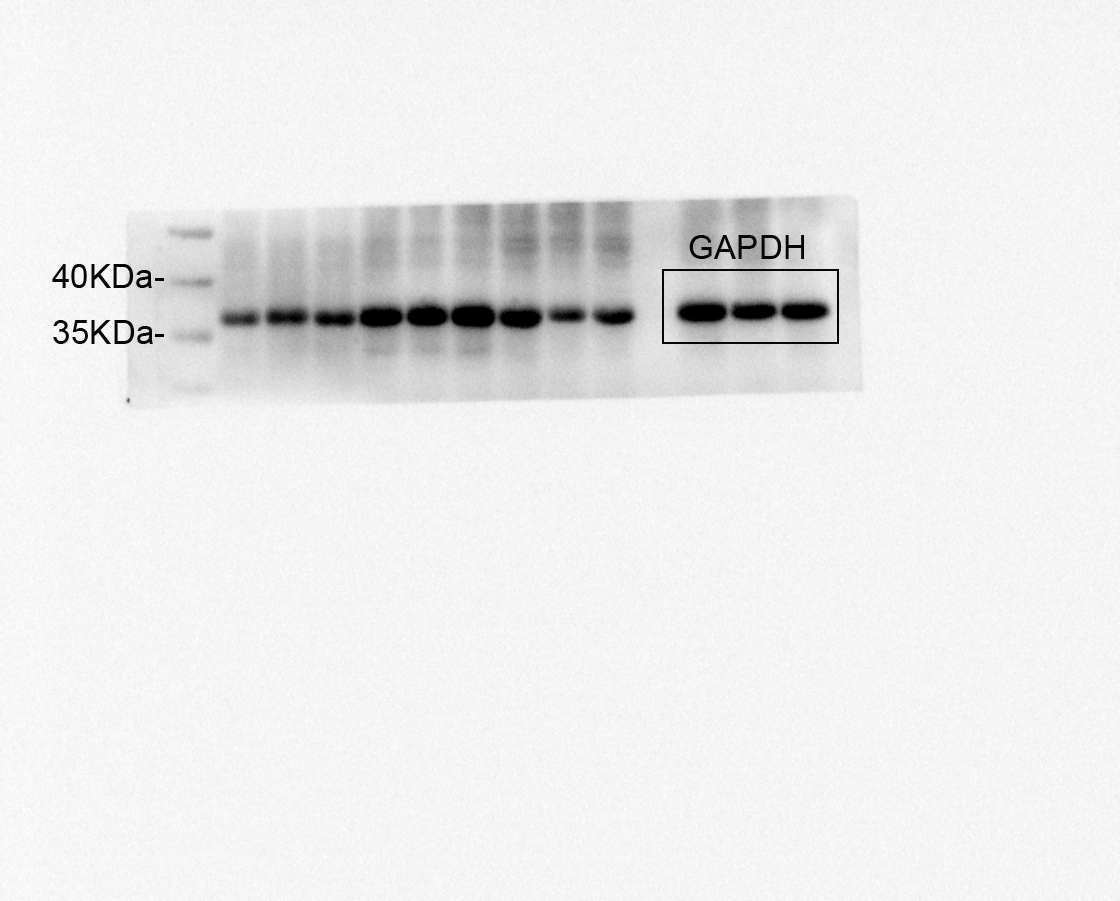

Supplement: Figure 5—source data 1. [file elife-95318-fig5-data1.zip › Figure5-Source data 1/Uncropped western blots-Fig5F/GAPDH.tif]

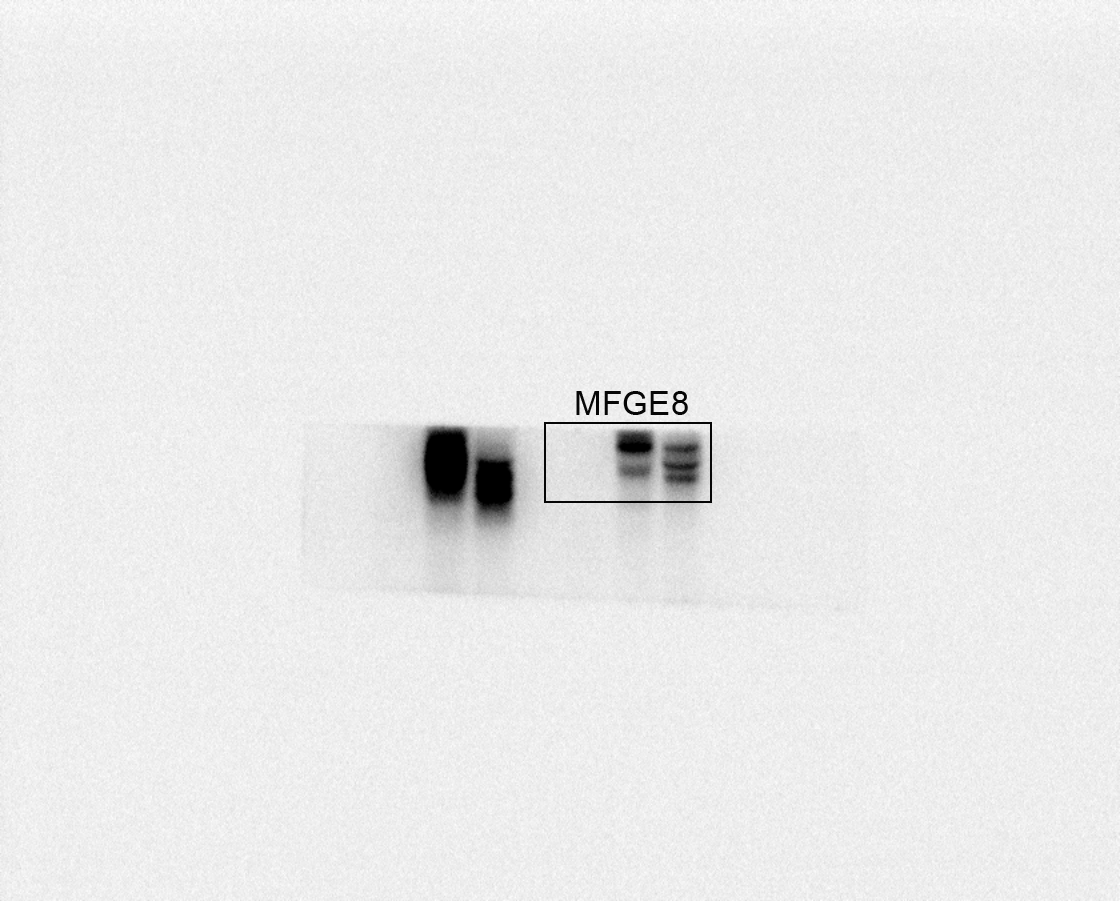

Supplement: Figure 5—source data 1. [file elife-95318-fig5-data1.zip › Figure5-Source data 1/Uncropped western blots-Fig5F/MFGE8.tif]

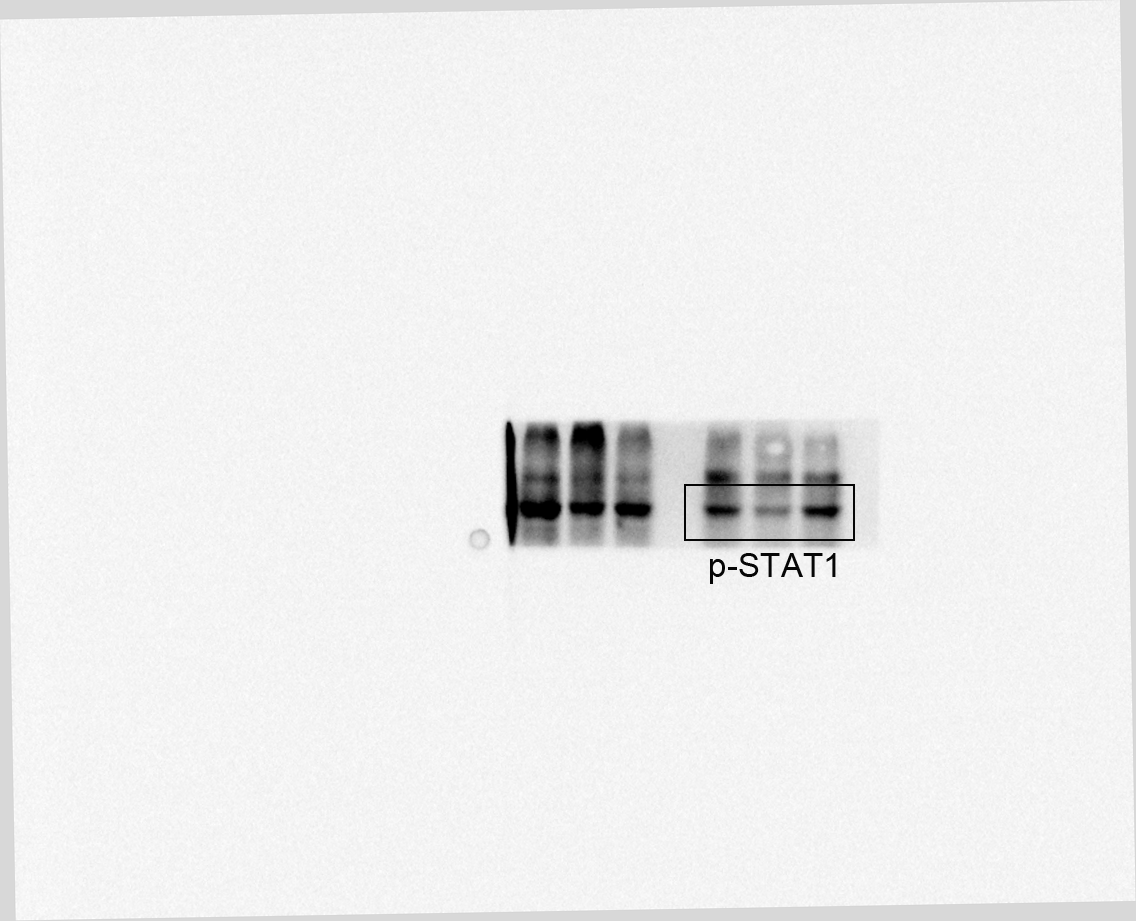

Supplement: Figure 5—source data 1. [file elife-95318-fig5-data1.zip › Figure5-Source data 1/Uncropped western blots-Fig5F/P-STAT1.tif]

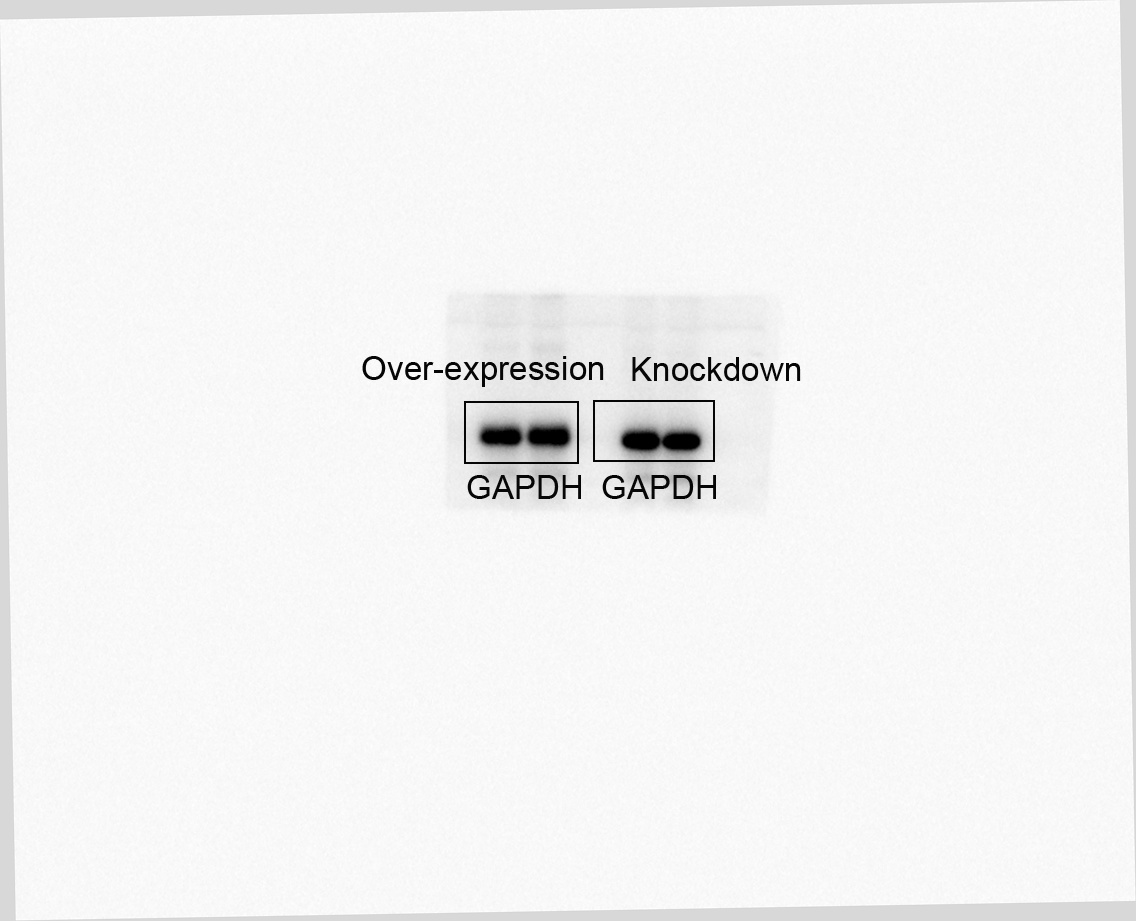

Supplement: Figure 5—figure supplement 1—source data 1. [file elife-95318-fig5-figsupp1-data1.zip › Figure5-figure supplement 1-Source data 1/Uncropped blots-Sup Fig4A/GAPDH-OV+sh.tif]

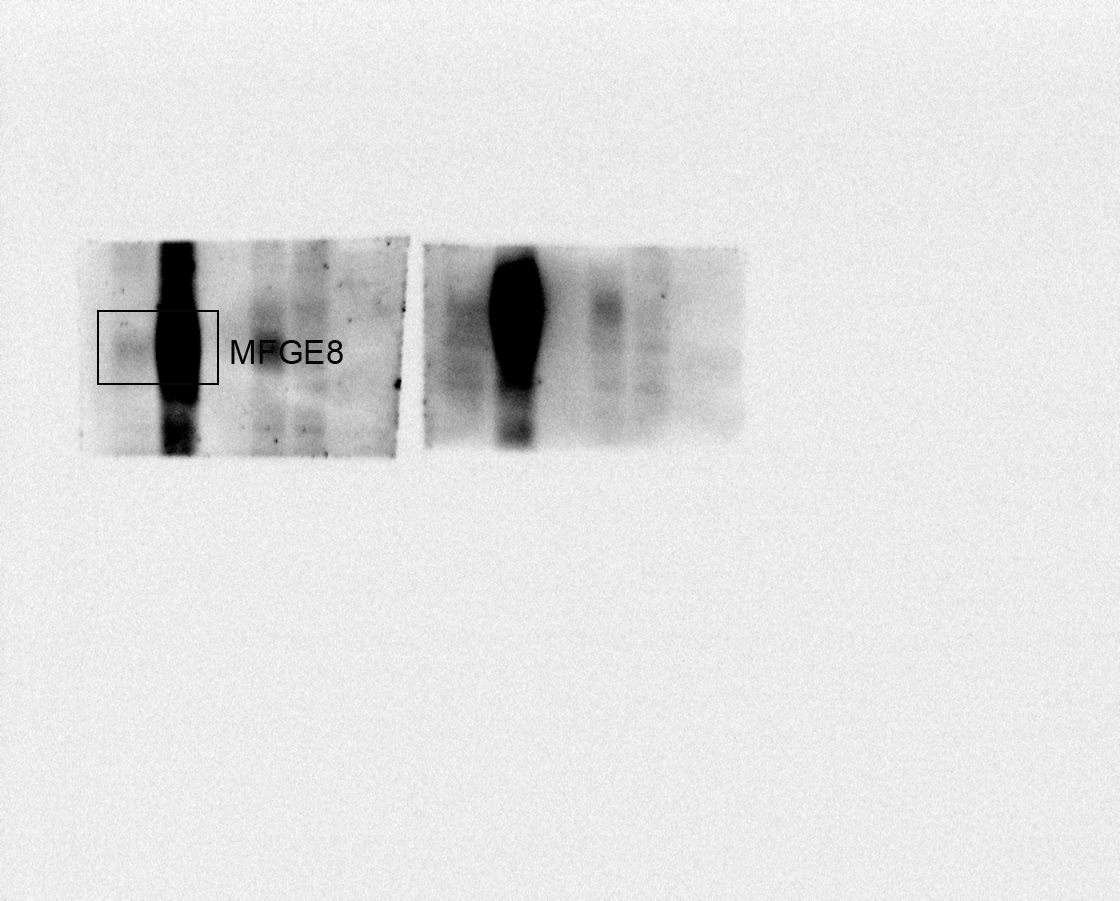

Supplement: Figure 5—figure supplement 1—source data 1. [file elife-95318-fig5-figsupp1-data1.zip › Figure5-figure supplement 1-Source data 1/Uncropped blots-Sup Fig4A/MFGE8-OV (LE).tif]

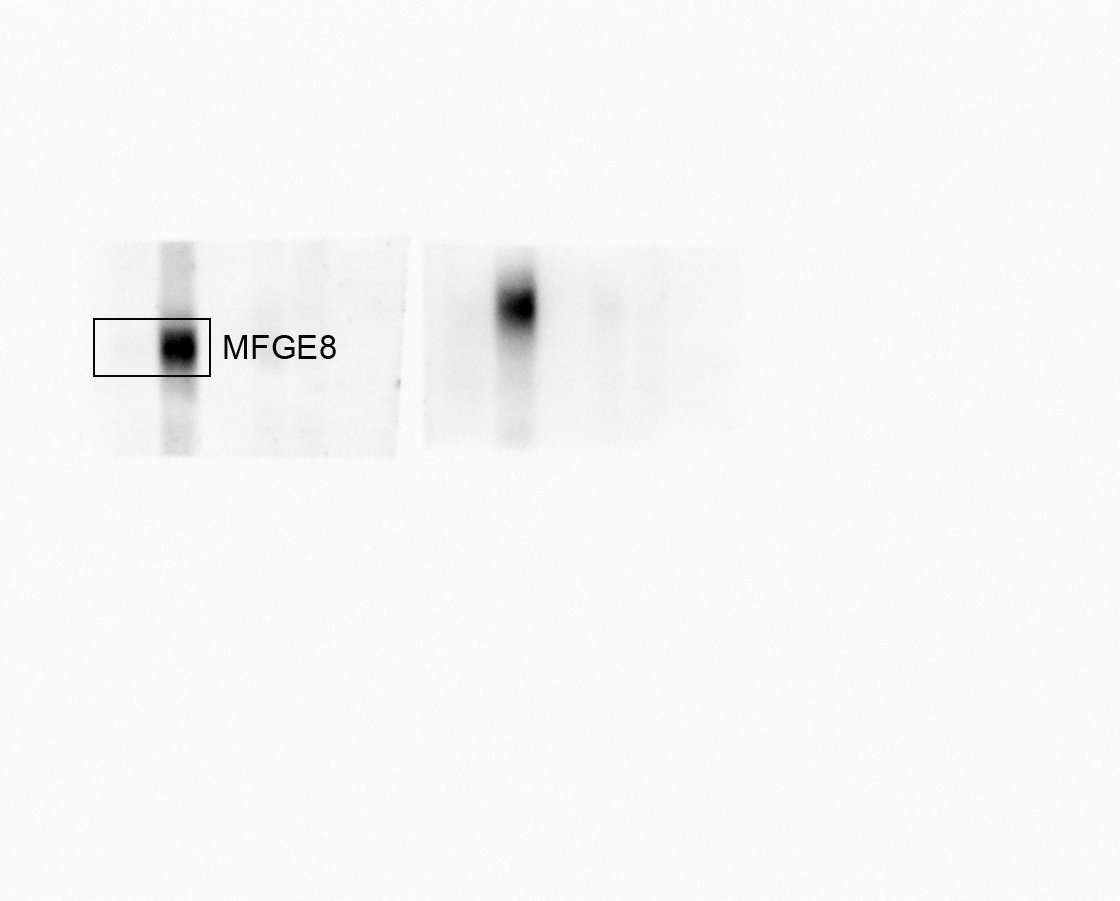

Supplement: Figure 5—figure supplement 1—source data 1. [file elife-95318-fig5-figsupp1-data1.zip › Figure5-figure supplement 1-Source data 1/Uncropped blots-Sup Fig4A/MFGE8-OV.tif]

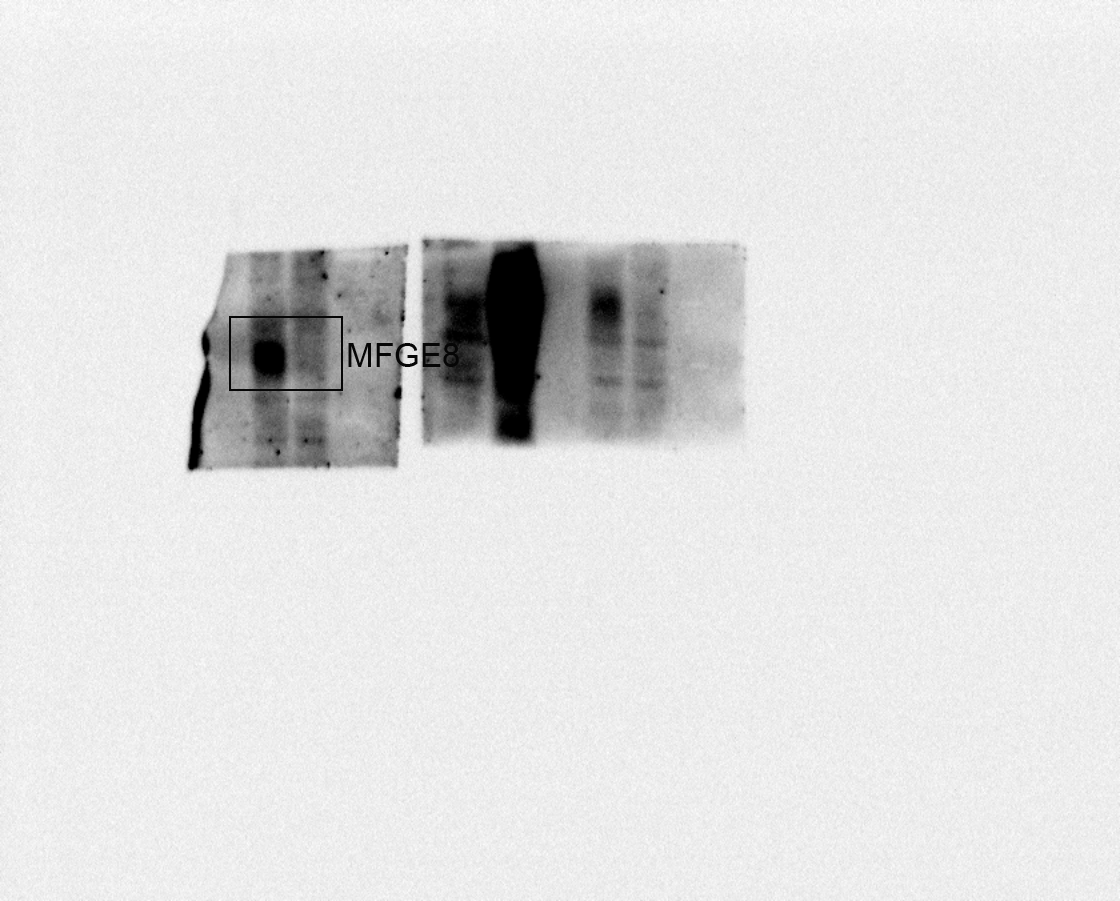

Supplement: Figure 5—figure supplement 1—source data 1. [file elife-95318-fig5-figsupp1-data1.zip › Figure5-figure supplement 1-Source data 1/Uncropped blots-Sup Fig4A/MFGE8-sh (LE).tif]

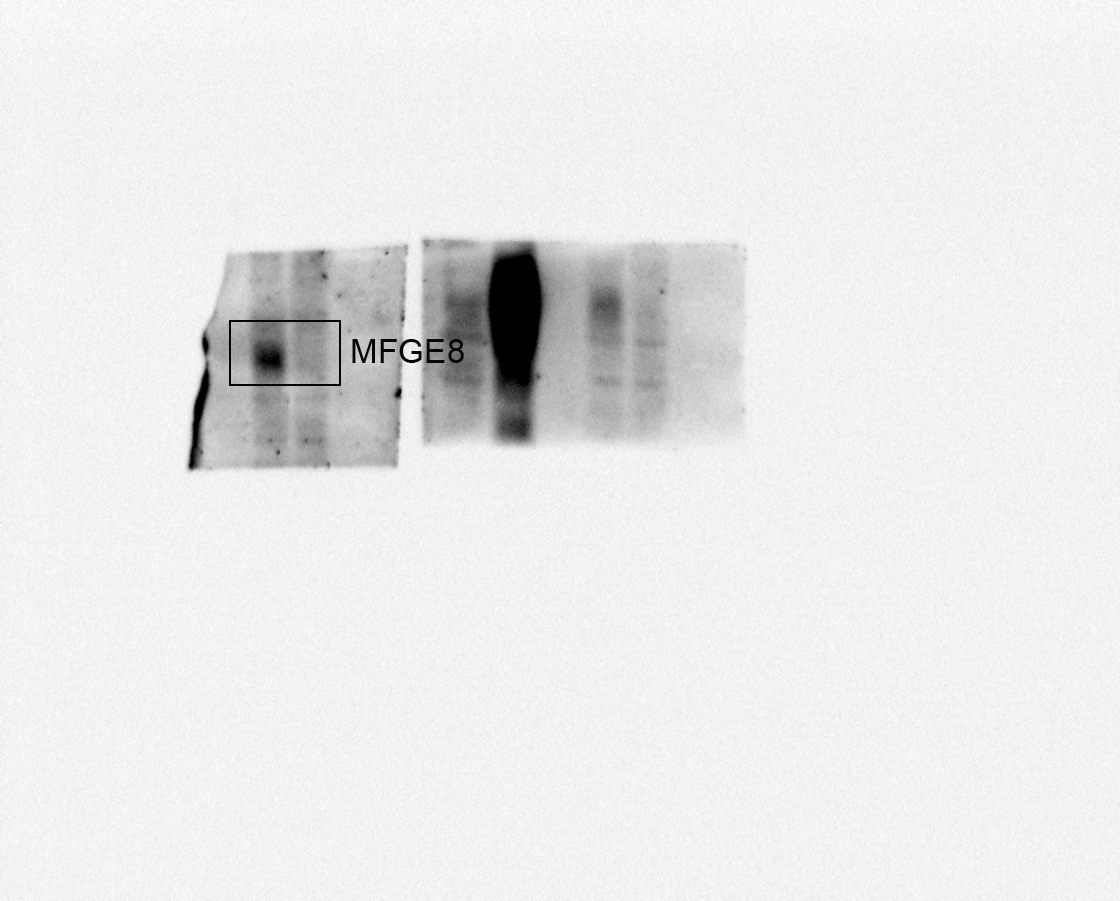

Supplement: Figure 5—figure supplement 1—source data 1. [file elife-95318-fig5-figsupp1-data1.zip › Figure5-figure supplement 1-Source data 1/Uncropped blots-Sup Fig4A/MFGE8-sh.tif]

Sup Figure4A

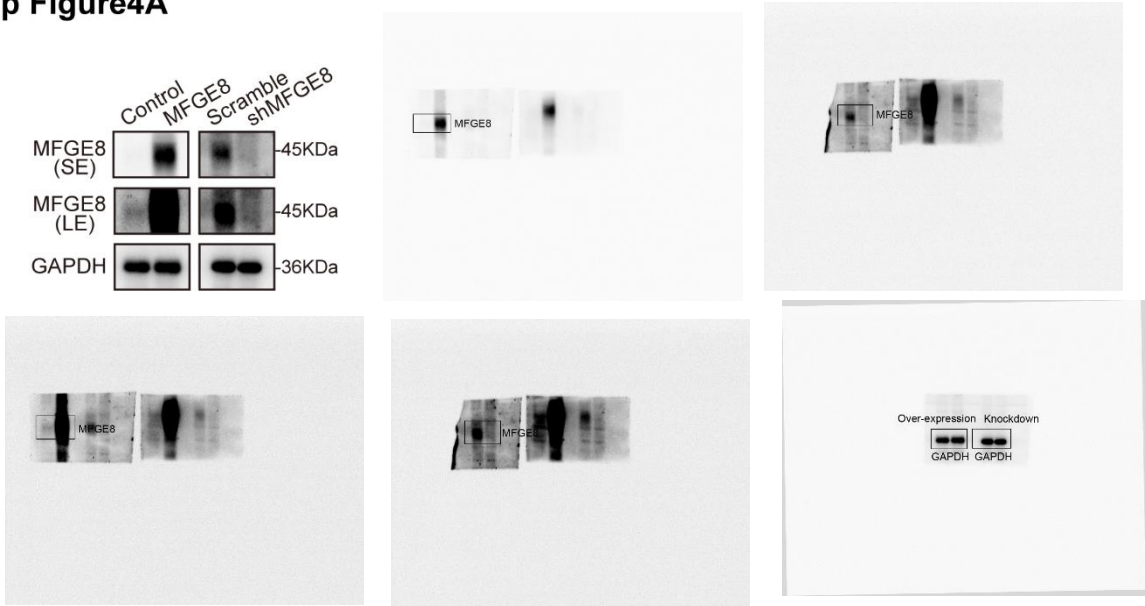

Supplement: Figure 5—figure supplement 1—source data 2. [file elife-95318-fig5-figsupp1-data2.zip › Figure5-figure supplement 1-Source data 2/Sup Fig4A.pdf]

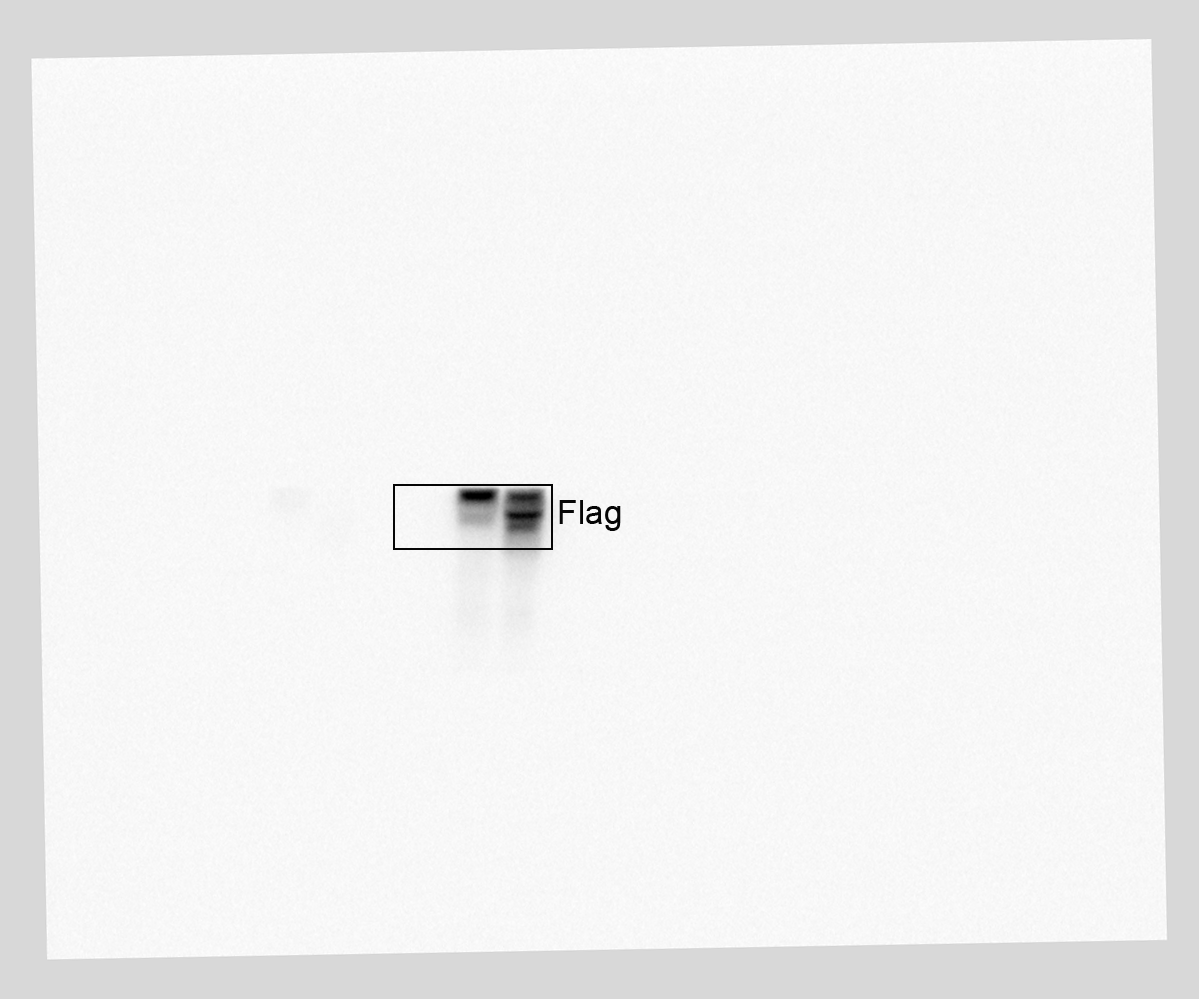

Supplement: Figure 5—figure supplement 1—source data 3. [file elife-95318-fig5-figsupp1-data3.zip › Figure5-figure supplement 1-Source data 3/Uncropped blots-Sup Fig4D/HCC1937 Flag.tif]

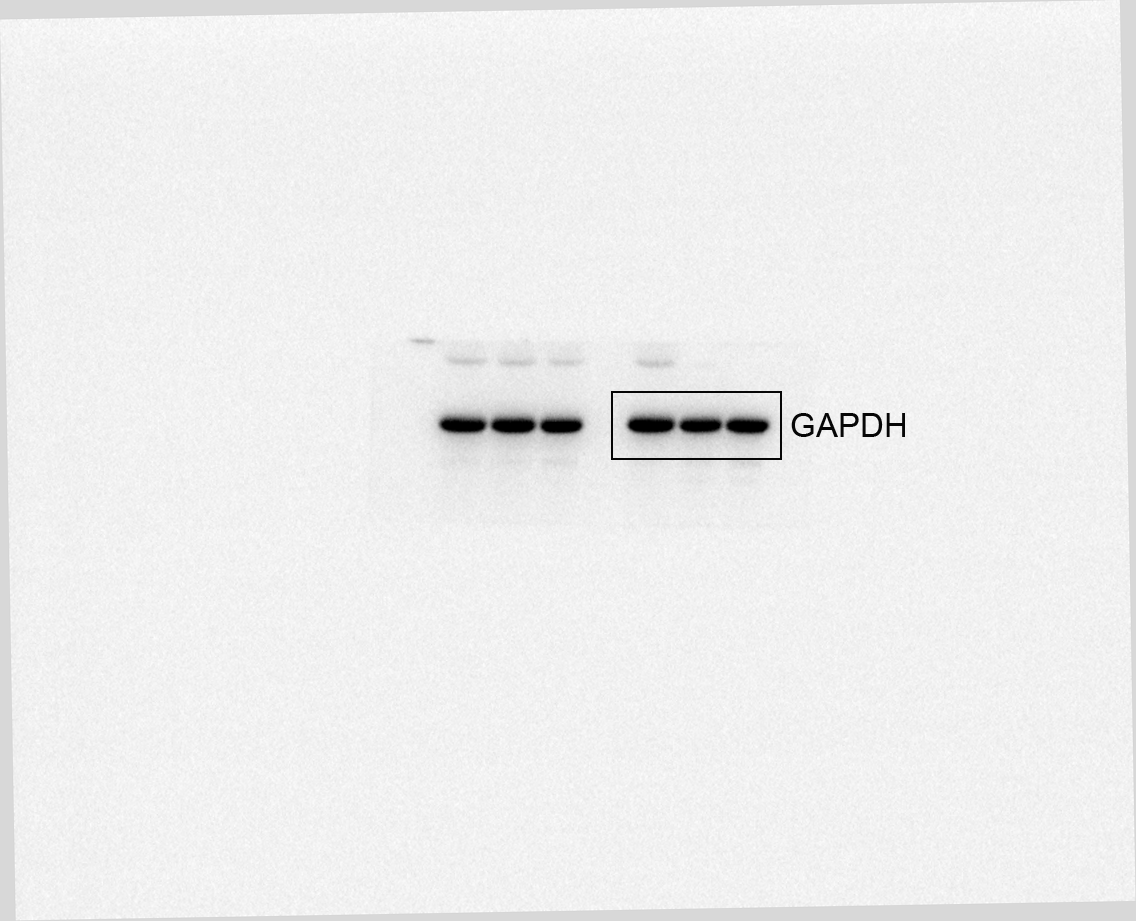

Supplement: Figure 5—figure supplement 1—source data 3. [file elife-95318-fig5-figsupp1-data3.zip › Figure5-figure supplement 1-Source data 3/Uncropped blots-Sup Fig4D/HCC1937 GAPDH.tif]

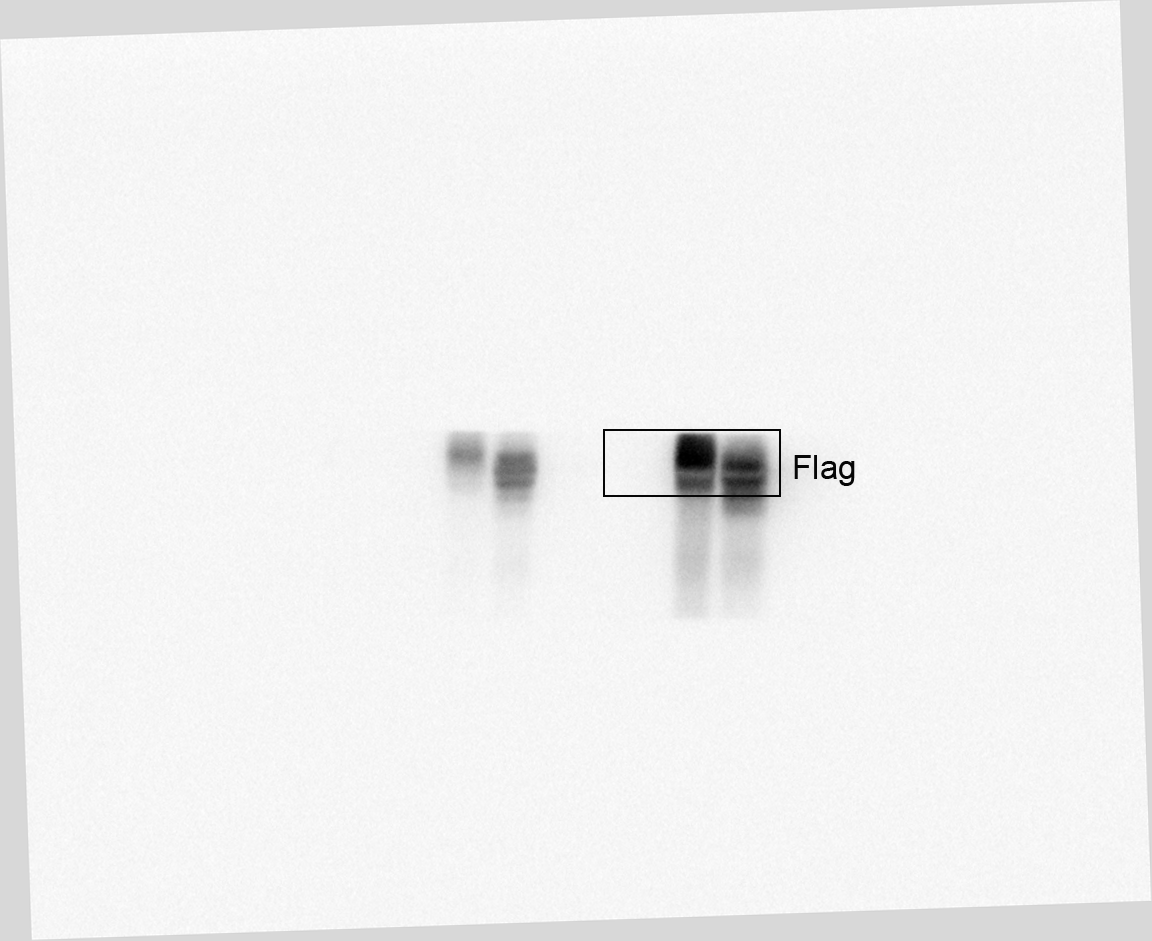

Supplement: Figure 5—figure supplement 1—source data 3. [file elife-95318-fig5-figsupp1-data3.zip › Figure5-figure supplement 1-Source data 3/Uncropped blots-Sup Fig4D/MDA-MB-231 FLAG.tif]

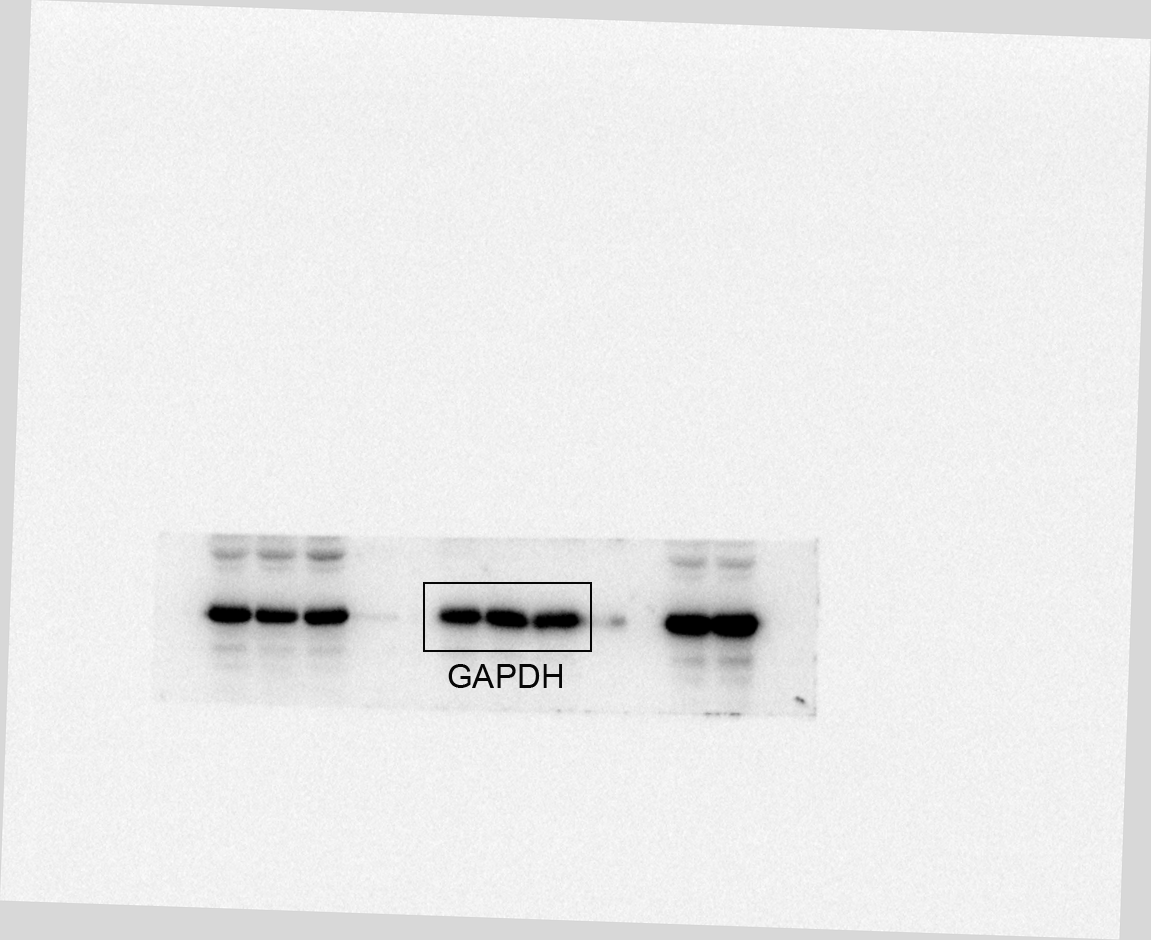

Supplement: Figure 5—figure supplement 1—source data 3. [file elife-95318-fig5-figsupp1-data3.zip › Figure5-figure supplement 1-Source data 3/Uncropped blots-Sup Fig4D/MDA-MB-231 GAPDH.tif]

Sup Figure4D

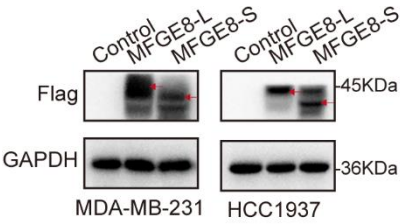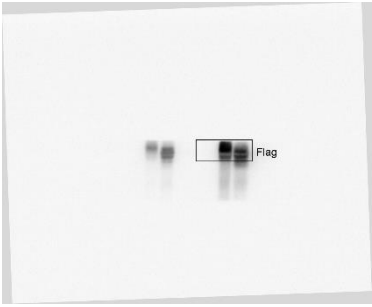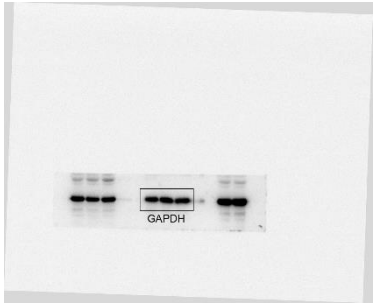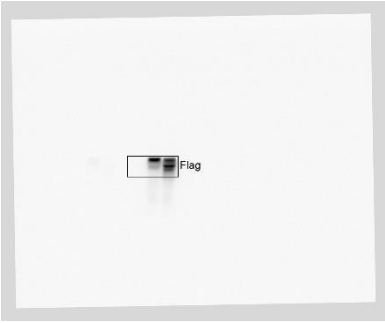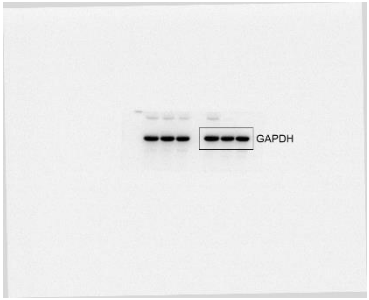

Supplement: Figure 5—figure supplement 1—source data 4. [file elife-95318-fig5-figsupp1-data4.zip › Figure5-figure supplement 1-Source data 4/Sup Fig4D.pdf]

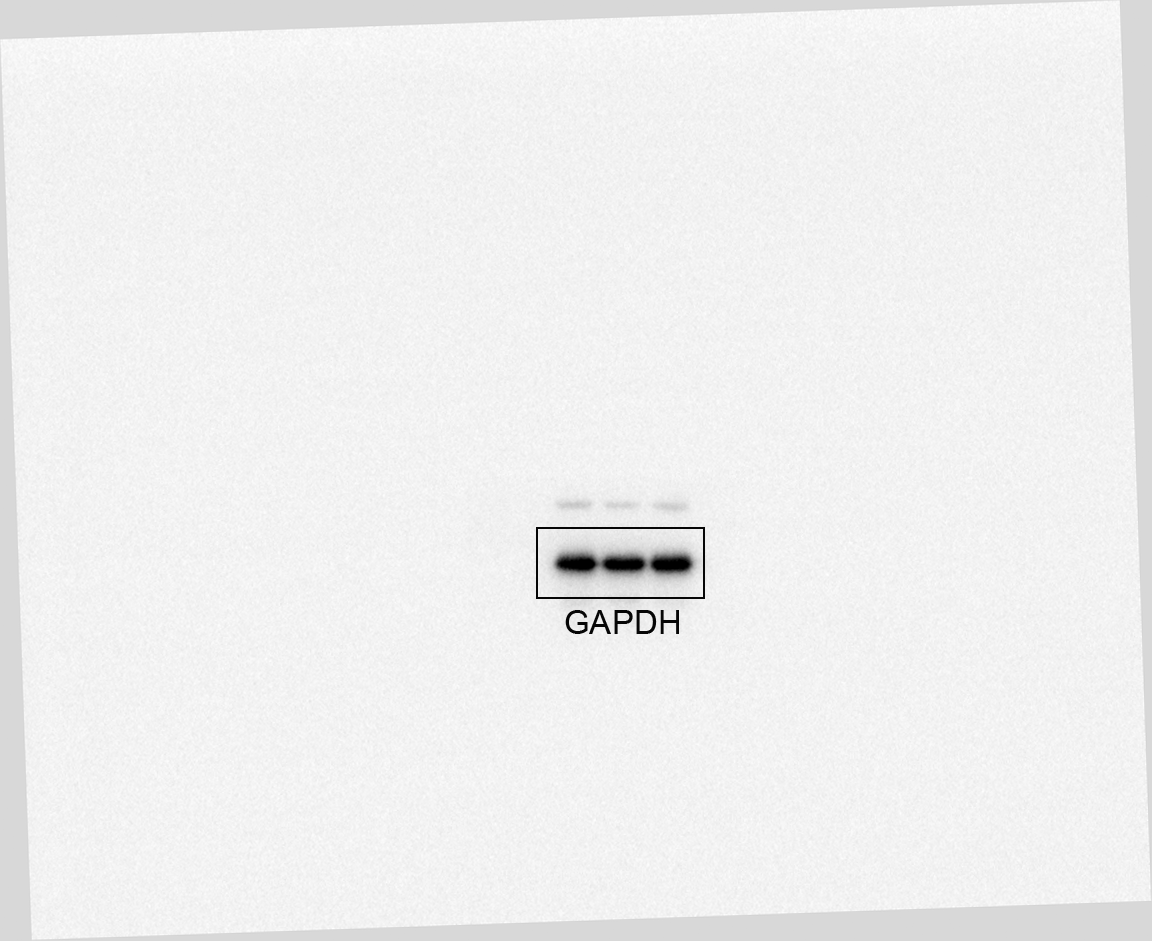

Supplement: Figure 6—source data 1. [file elife-95318-fig6-data1.zip › Figure6-Source data 1/Uncropped western blots-Fig6E/BT549/BT549-GAPDH.tif]

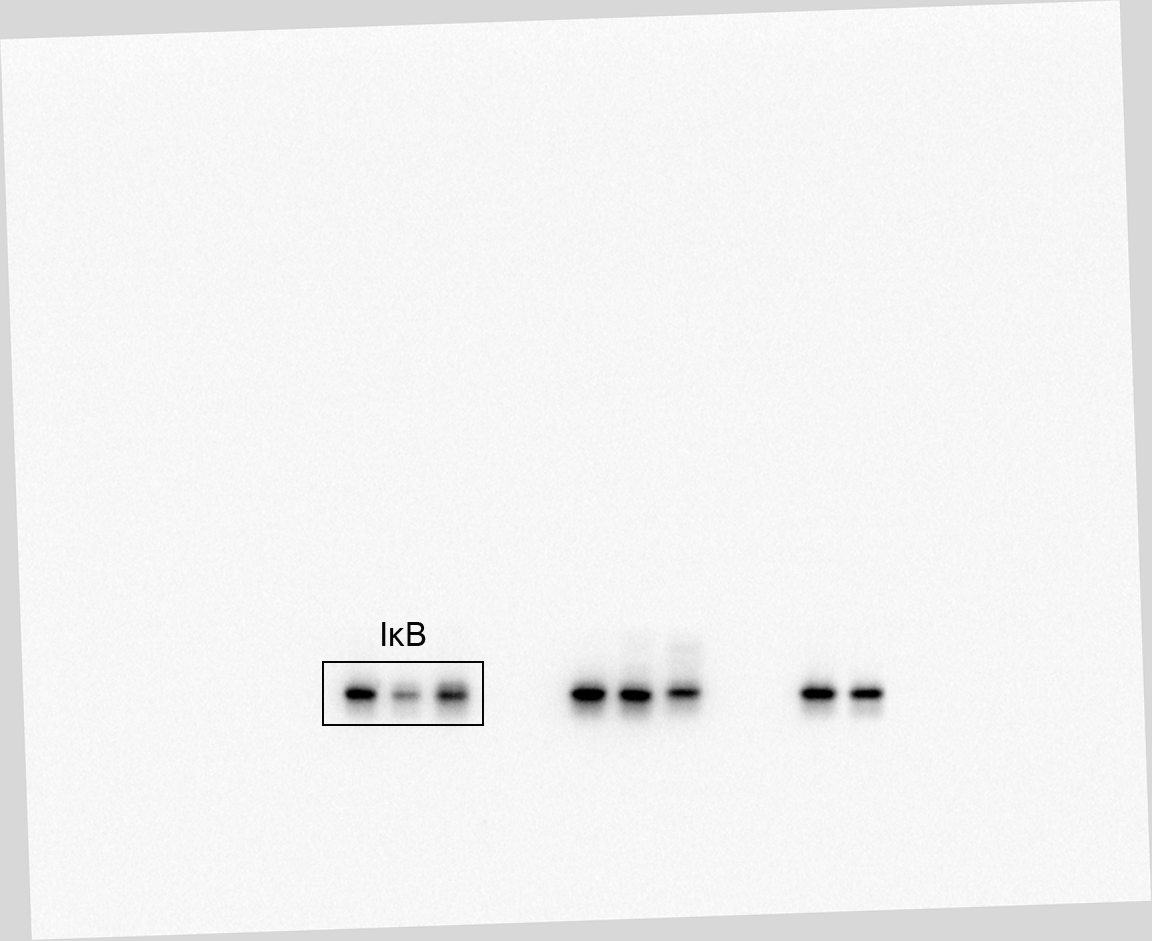

Supplement: Figure 6—source data 1. [file elife-95318-fig6-data1.zip › Figure6-Source data 1/Uncropped western blots-Fig6E/BT549/BT549-IκB.tif]

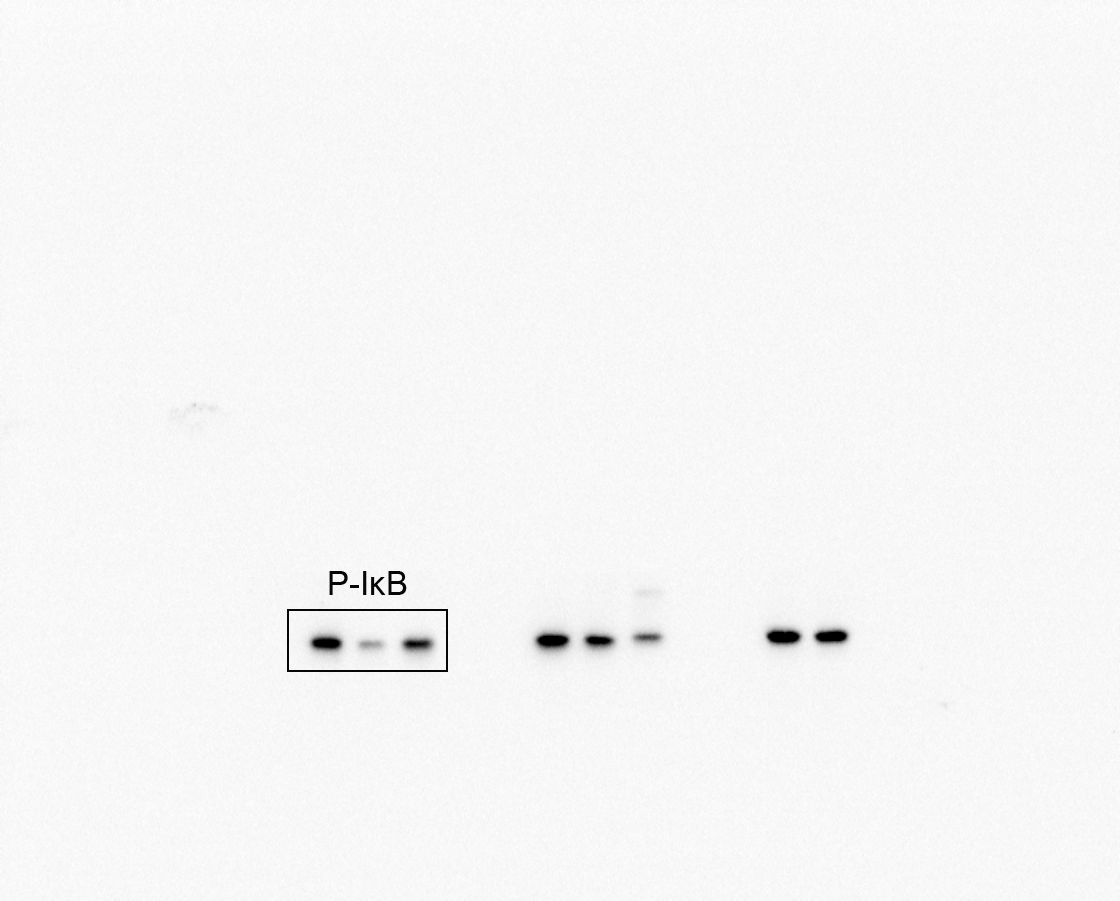

Supplement: Figure 6—source data 1. [file elife-95318-fig6-data1.zip › Figure6-Source data 1/Uncropped western blots-Fig6E/BT549/BT549-P-IκB.tif]

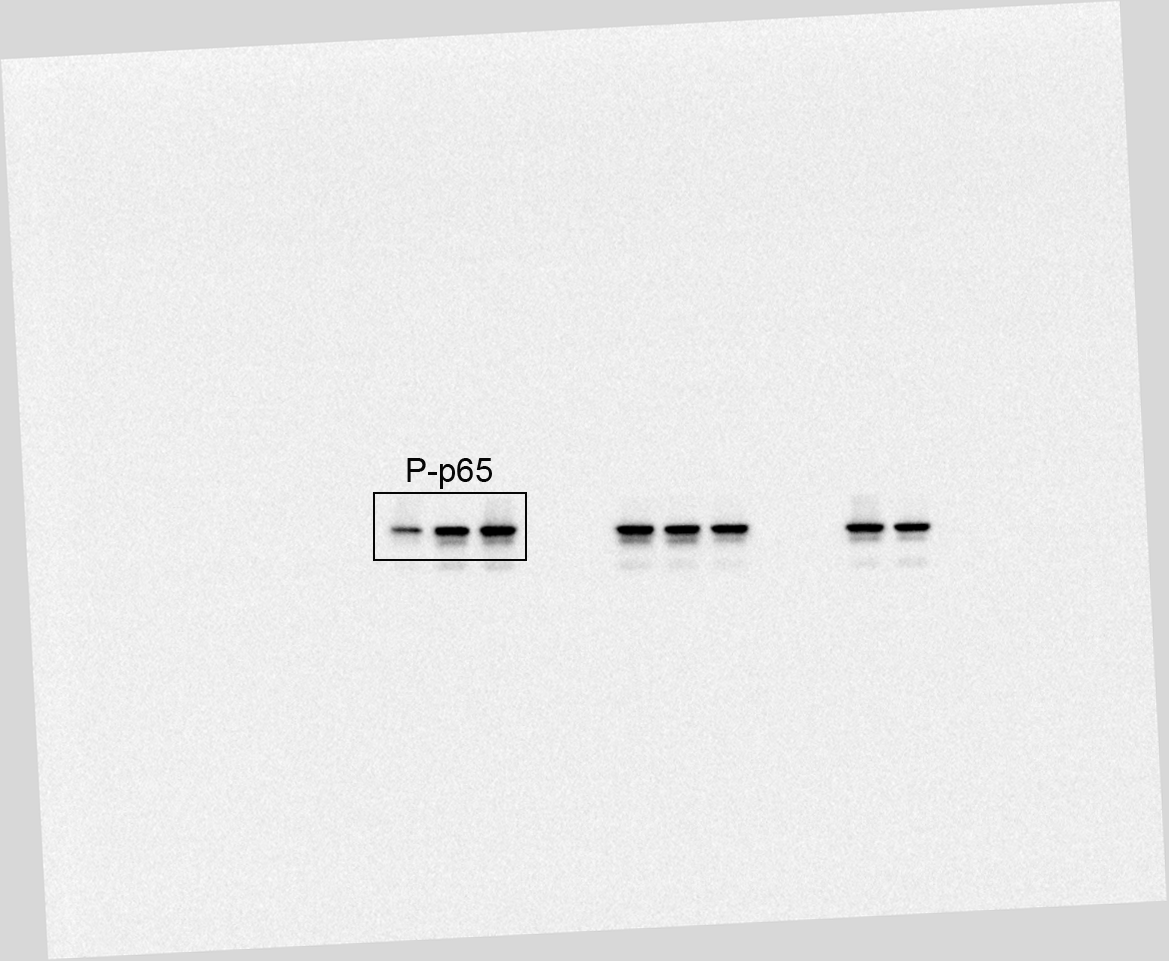

Supplement: Figure 6—source data 1. [file elife-95318-fig6-data1.zip › Figure6-Source data 1/Uncropped western blots-Fig6E/BT549/BT549-P-p65.tif]

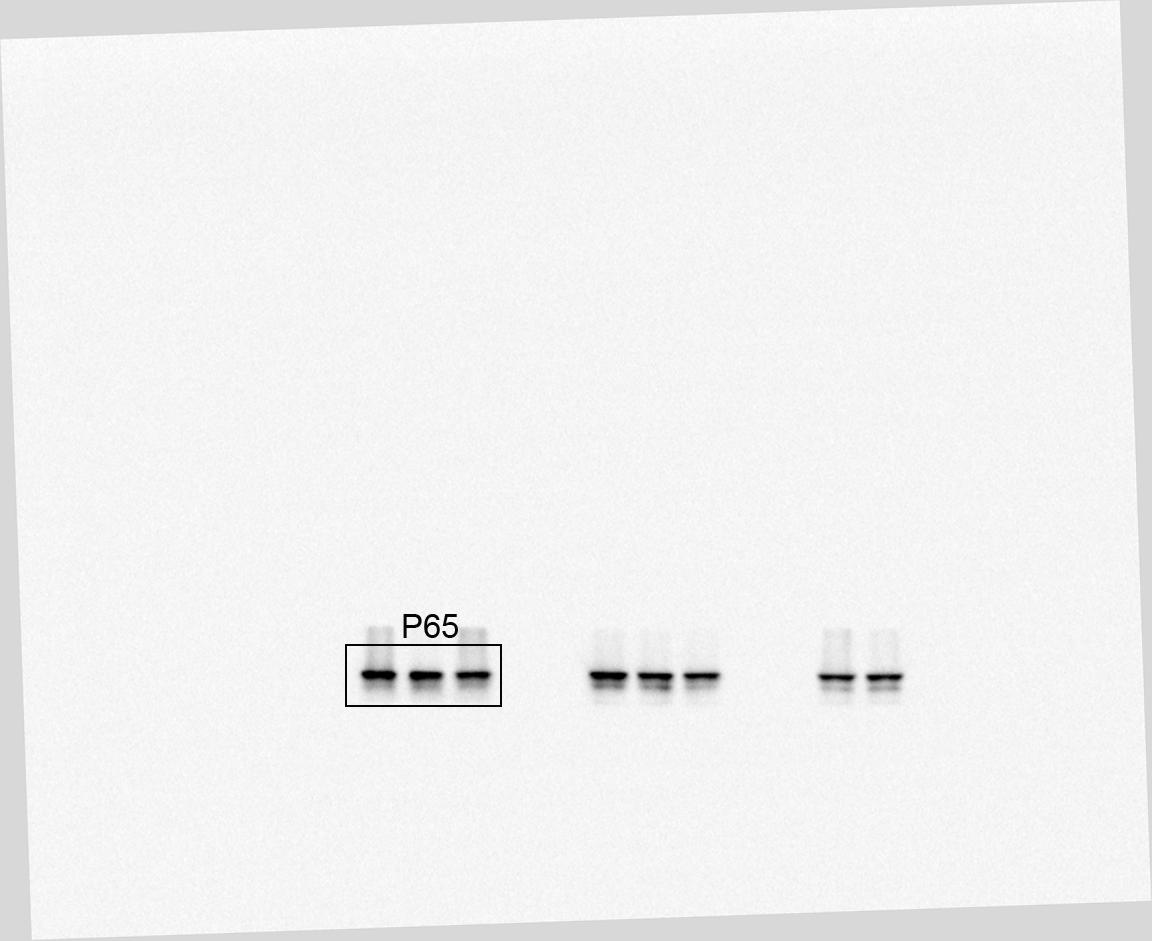

Supplement: Figure 6—source data 1. [file elife-95318-fig6-data1.zip › Figure6-Source data 1/Uncropped western blots-Fig6E/BT549/BT549-P65.tif]

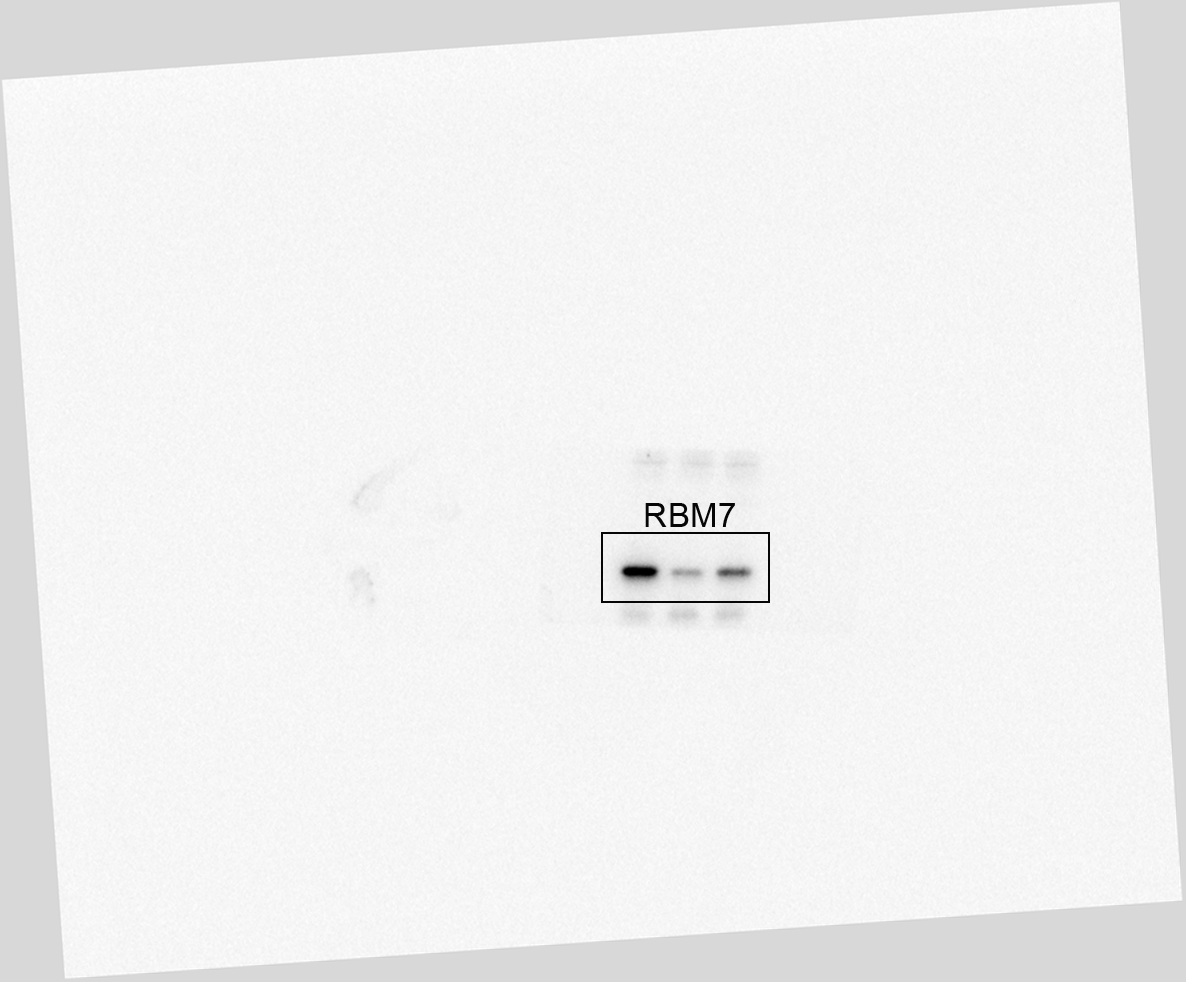

Supplement: Figure 6—source data 1. [file elife-95318-fig6-data1.zip › Figure6-Source data 1/Uncropped western blots-Fig6E/BT549/BT549-RBM7.tif]

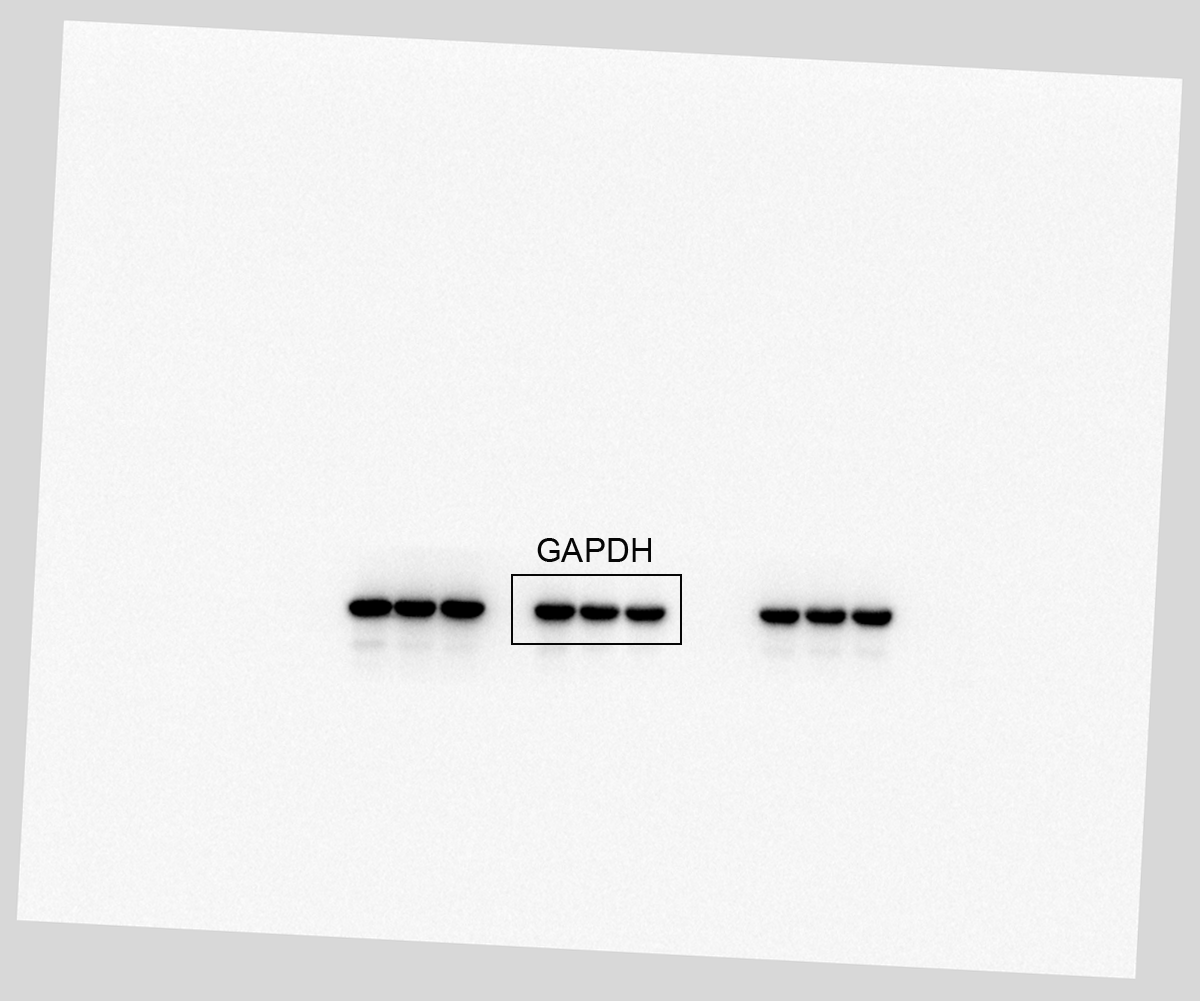

Supplement: Figure 6—source data 1. [file elife-95318-fig6-data1.zip › Figure6-Source data 1/Uncropped western blots-Fig6E/MDA-MB-231/MDA-MB-231 GAPDH.tif]

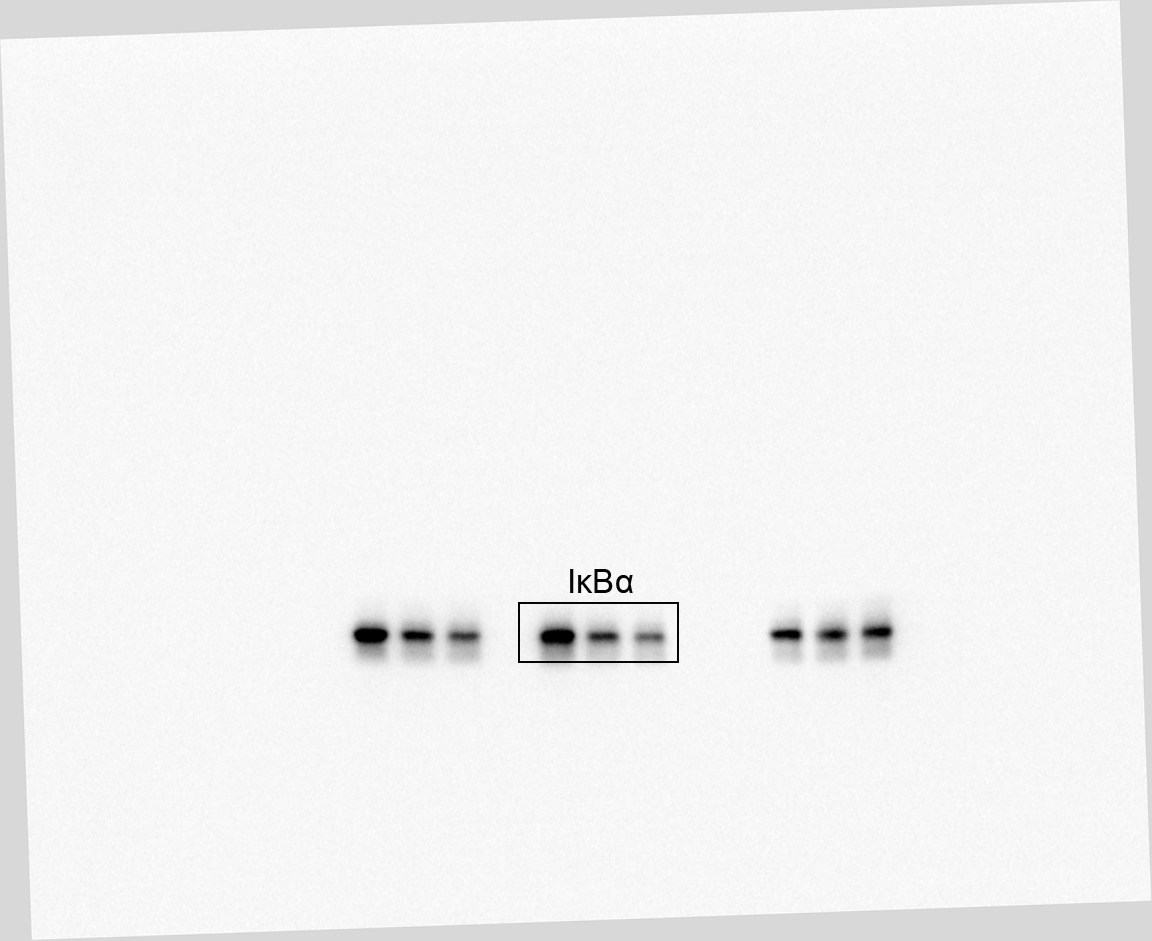

Supplement: Figure 6—source data 1. [file elife-95318-fig6-data1.zip › Figure6-Source data 1/Uncropped western blots-Fig6E/MDA-MB-231/MDA-MB-231 IκBα.tif]

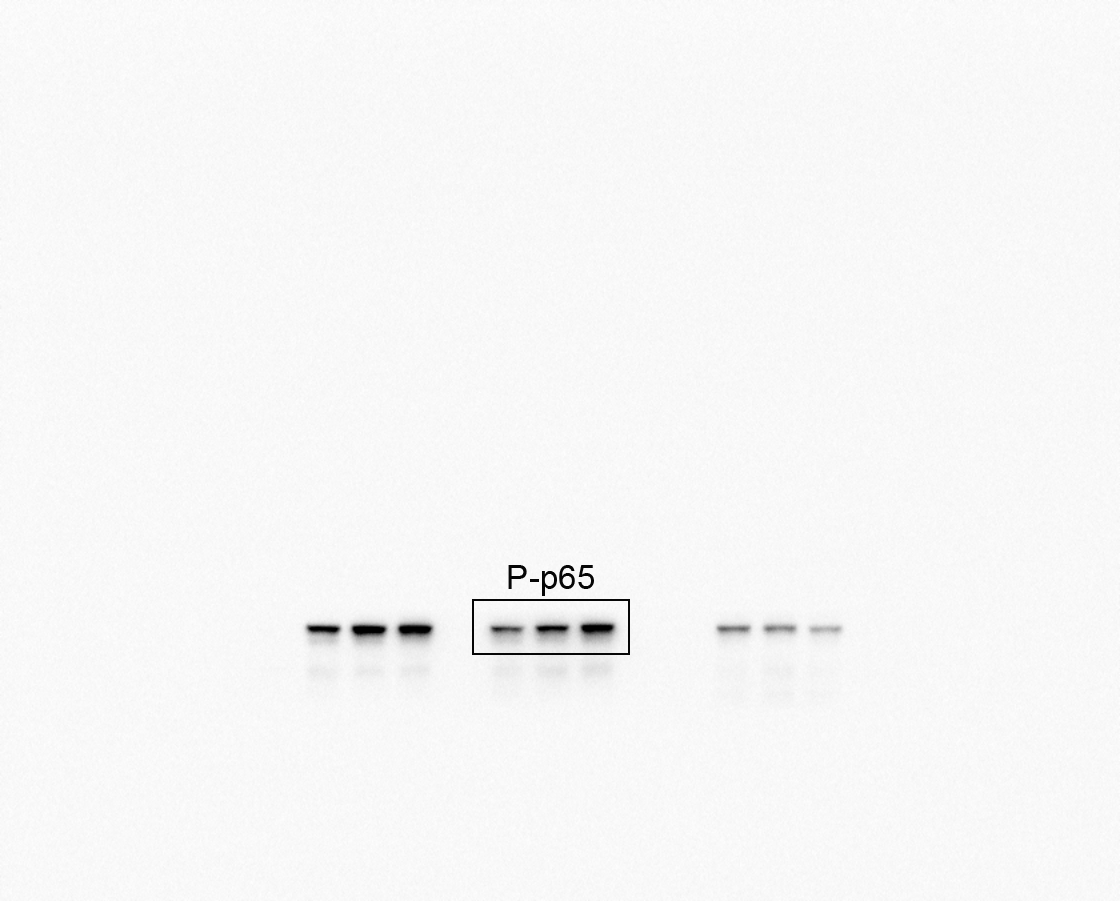

Supplement: Figure 6—source data 1. [file elife-95318-fig6-data1.zip › Figure6-Source data 1/Uncropped western blots-Fig6E/MDA-MB-231/MDA-MB-231 P-65.tif]

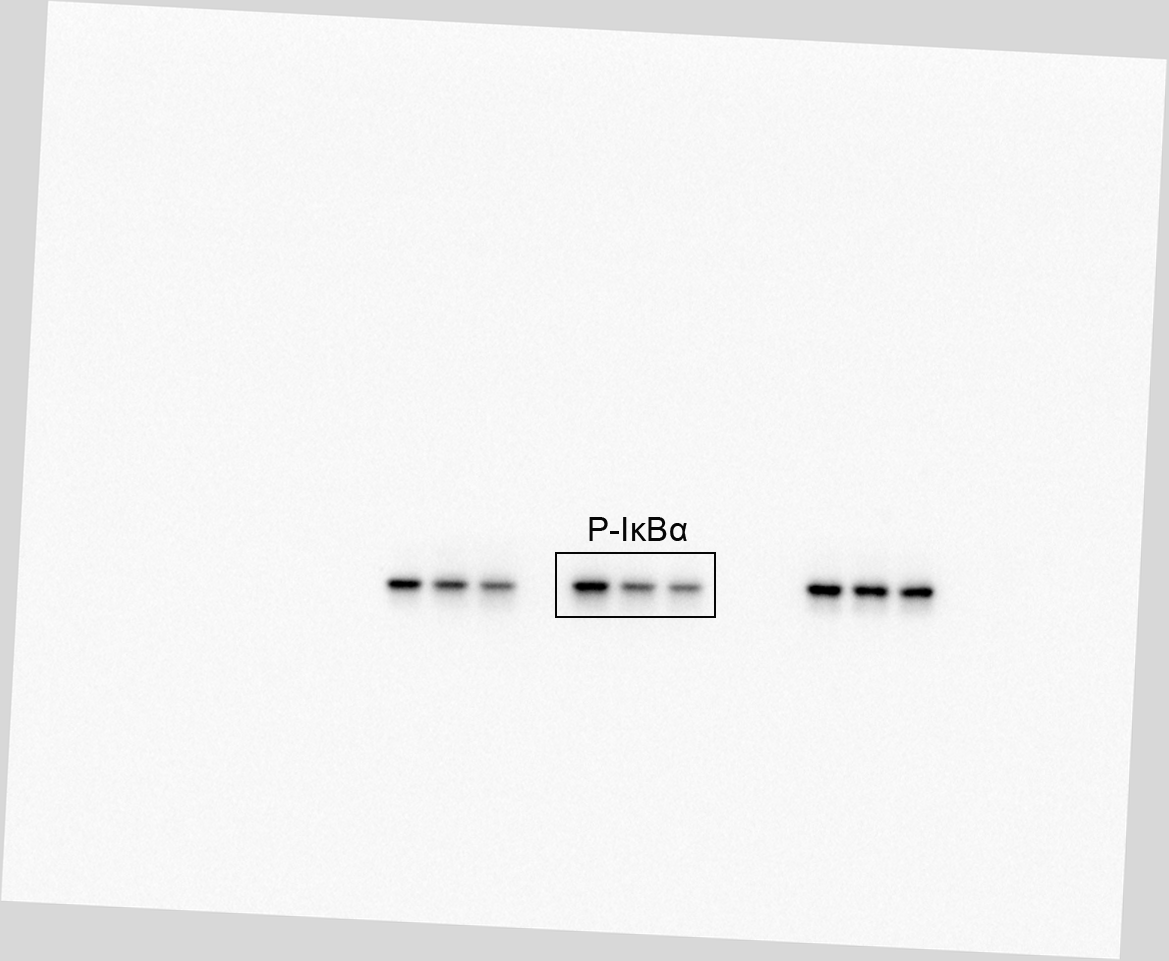

Supplement: Figure 6—source data 1. [file elife-95318-fig6-data1.zip › Figure6-Source data 1/Uncropped western blots-Fig6E/MDA-MB-231/MDA-MB-231 P-IκBα.tif]

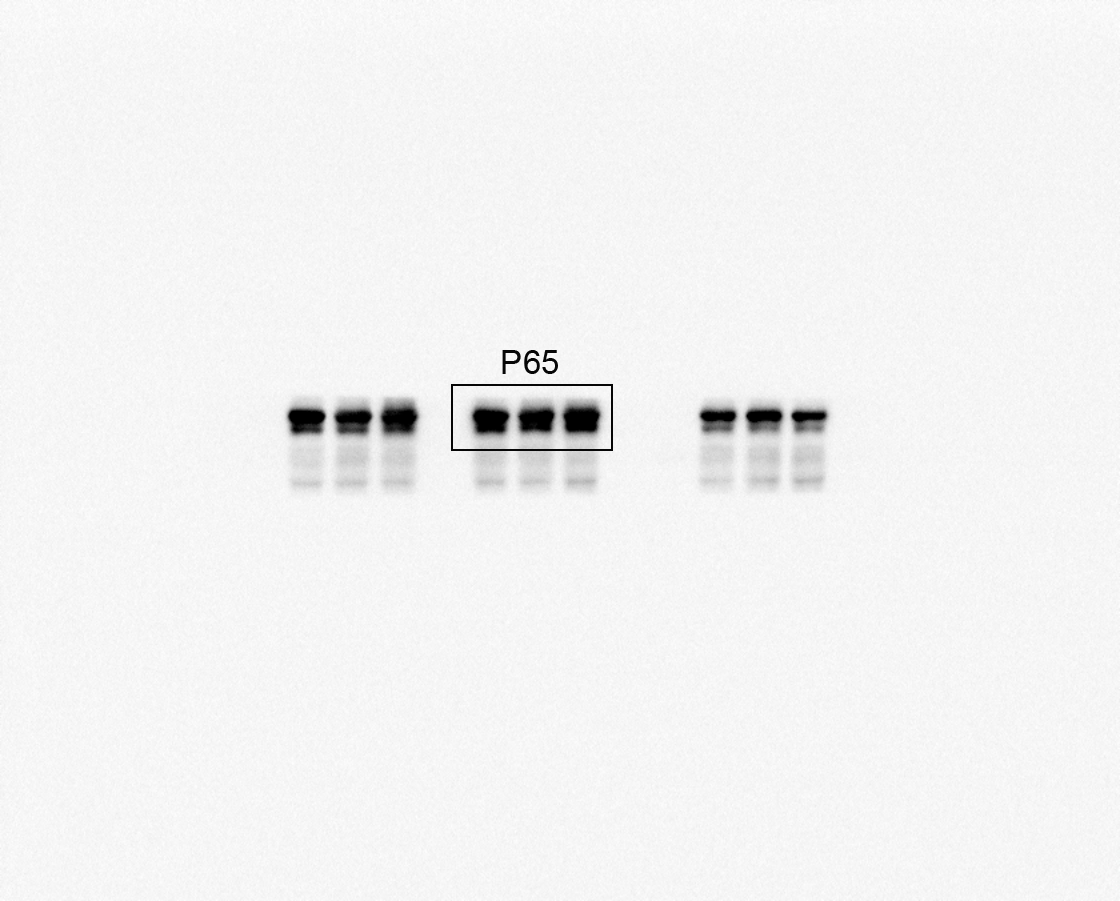

Supplement: Figure 6—source data 1. [file elife-95318-fig6-data1.zip › Figure6-Source data 1/Uncropped western blots-Fig6E/MDA-MB-231/MDA-MB-231 P65.tif]

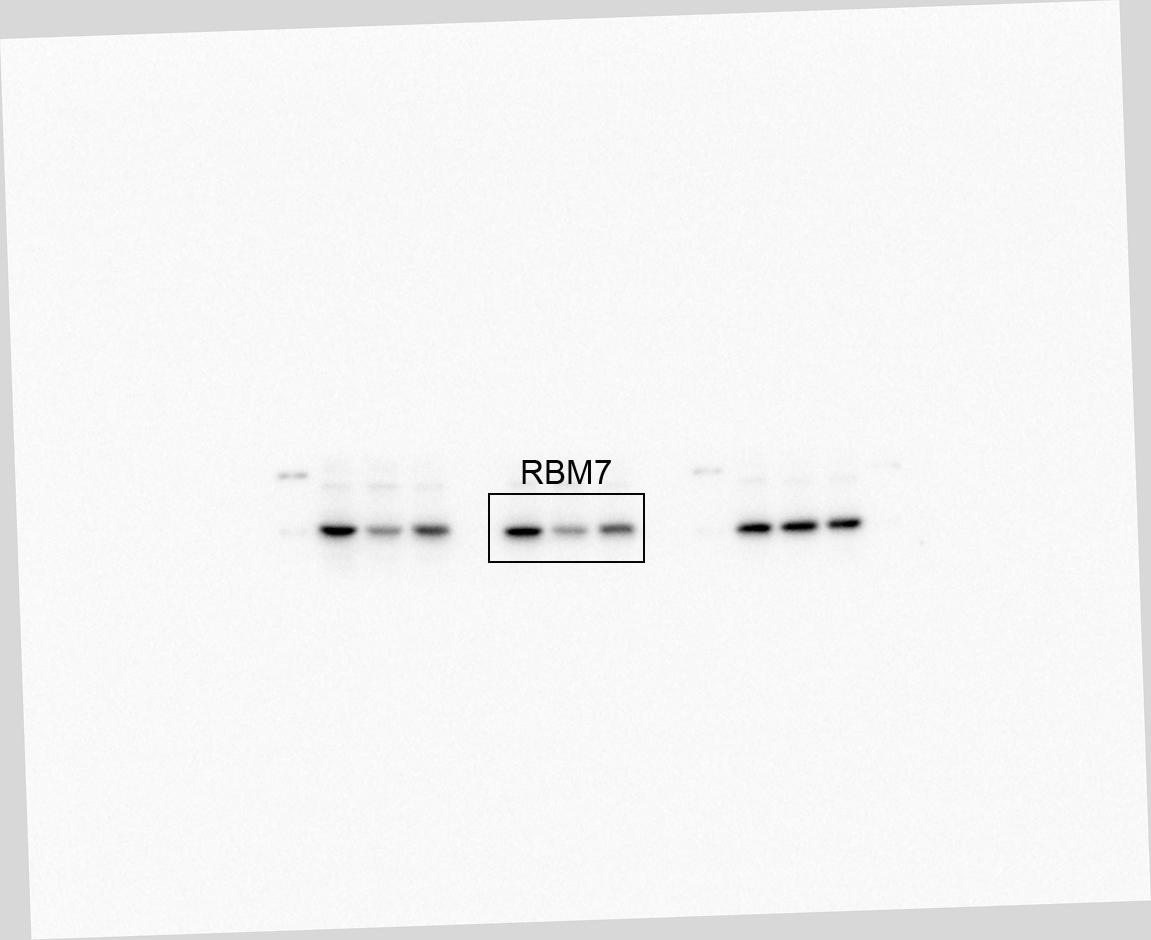

Supplement: Figure 6—source data 1. [file elife-95318-fig6-data1.zip › Figure6-Source data 1/Uncropped western blots-Fig6E/MDA-MB-231/MDA-MB-231 RBM7.tif]

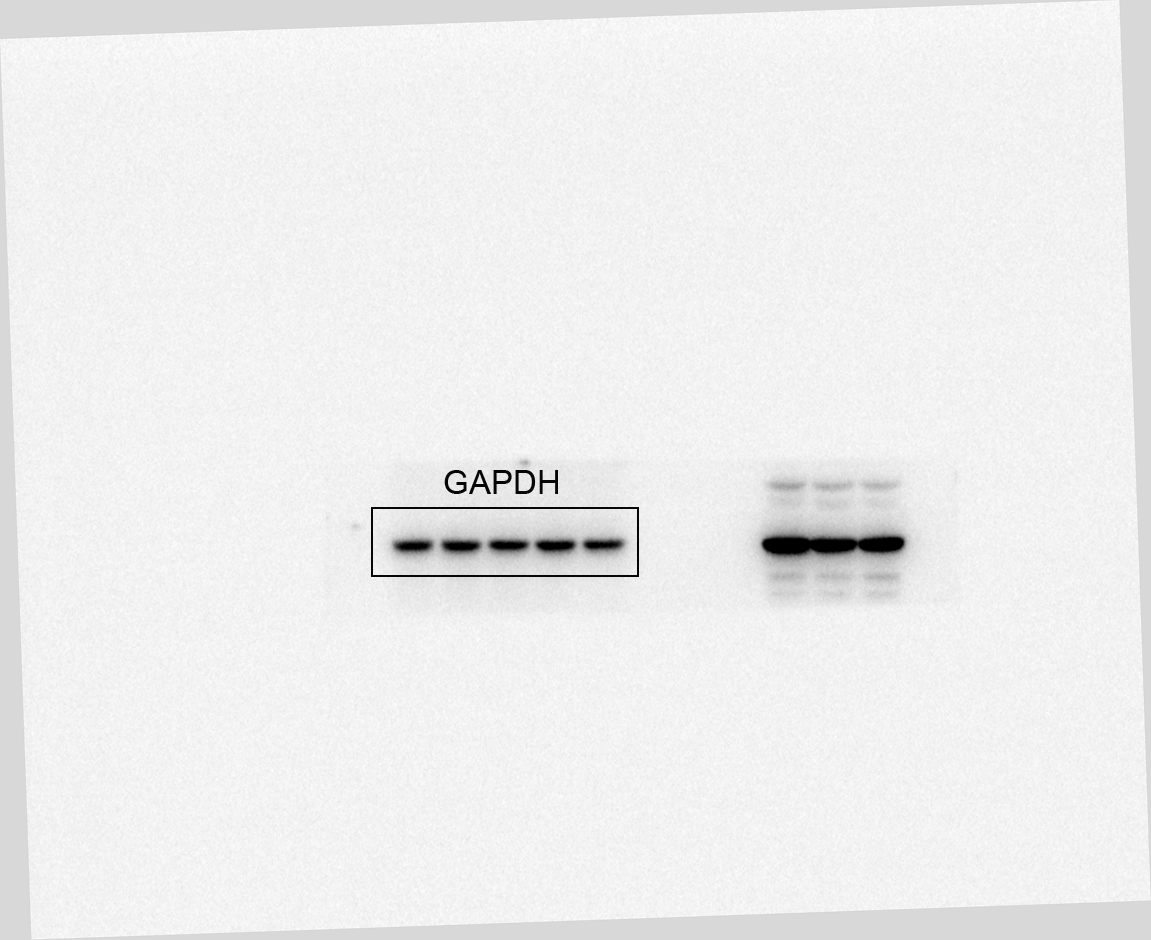

Supplement: Figure 6—source data 3. [file elife-95318-fig6-data3.zip › Figure6-Source data 3/Uncropped western blots-Fig6F/GAPDH.tif]

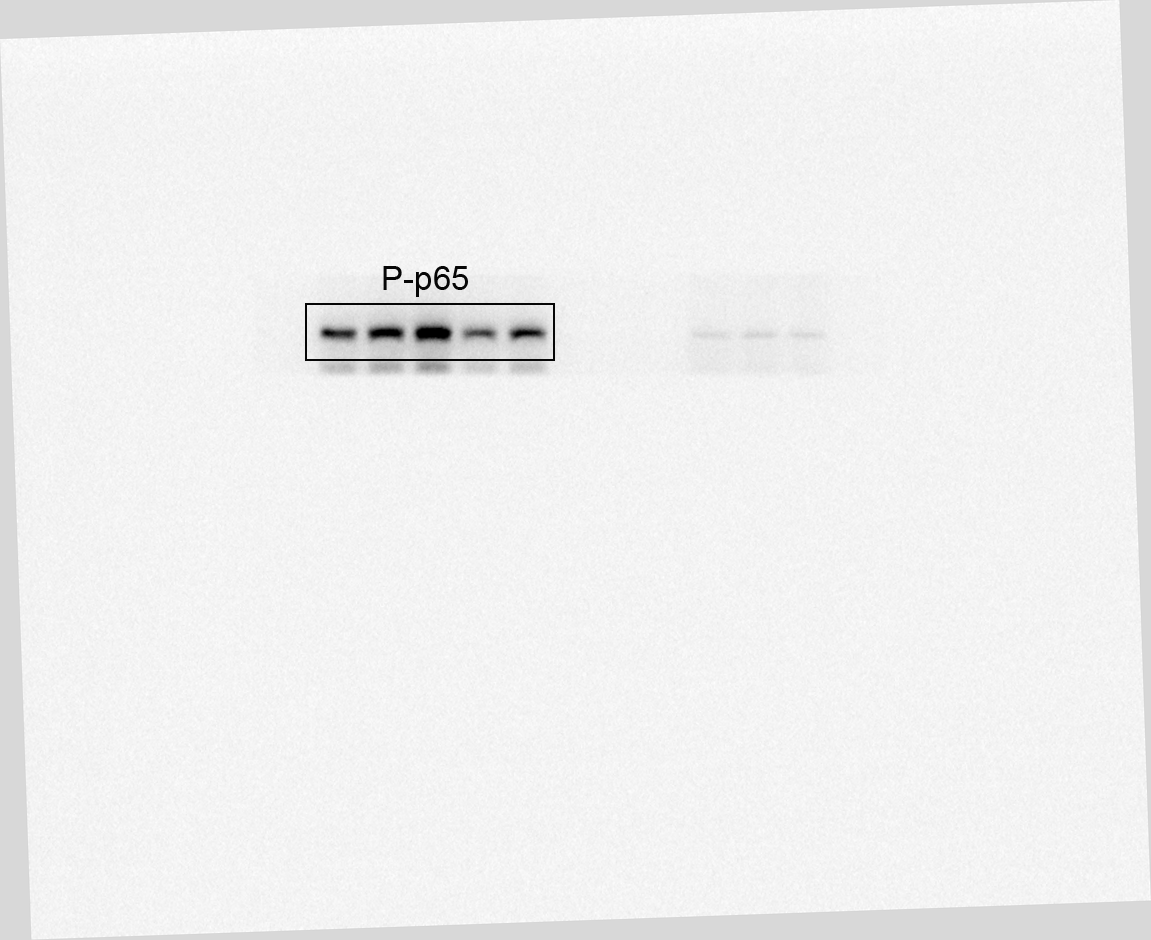

Supplement: Figure 6—source data 3. [file elife-95318-fig6-data3.zip › Figure6-Source data 3/Uncropped western blots-Fig6F/P-p65.tif]

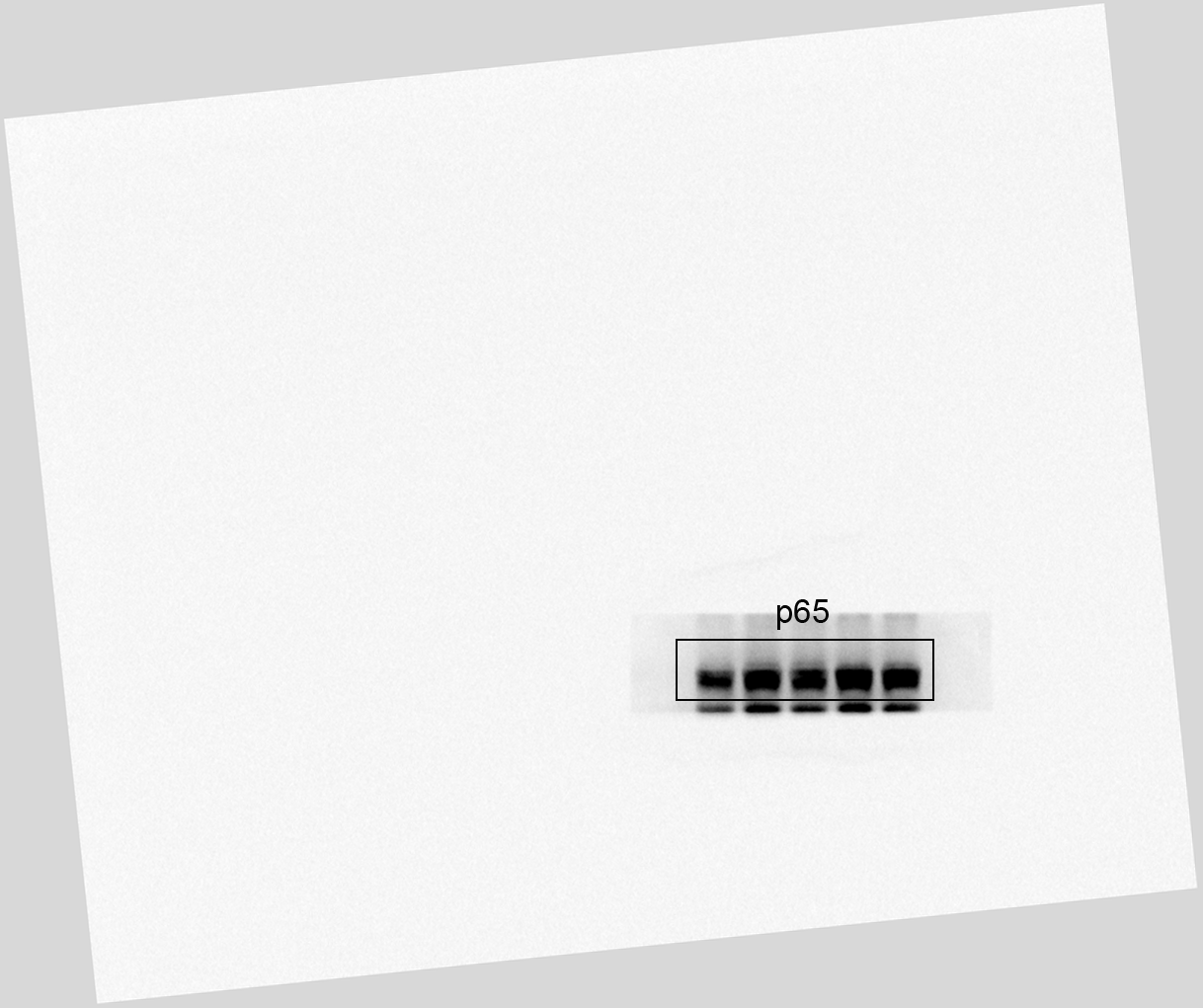

Supplement: Figure 6—source data 3. [file elife-95318-fig6-data3.zip › Figure6-Source data 3/Uncropped western blots-Fig6F/p65.tif]
