# Supplementary figures and images for: RBM7 deficiency promotes breast cancer metastasis by coordinating MFGE8 splicing switch and NF-kB pathway (part 2 of 2)
Source: eLife. 2024 Jul 12;13:RP95318. doi: 10.7554/eLife.95318 (PMC11245308; doi:10.7554/eLife.95318)

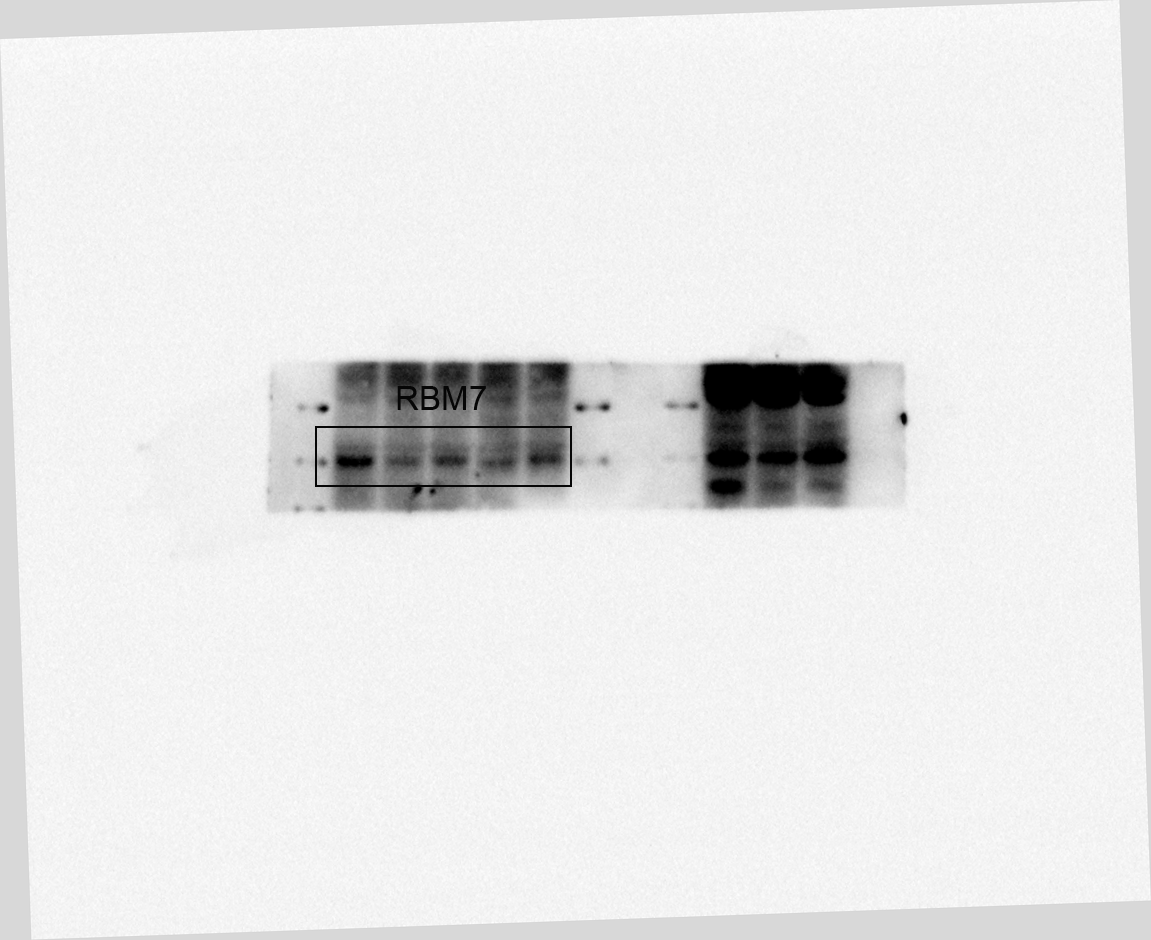

Supplement: Figure 6—source data 3. [file elife-95318-fig6-data3.zip › Figure6-Source data 3/Uncropped western blots-Fig6F/RBM7.tif]

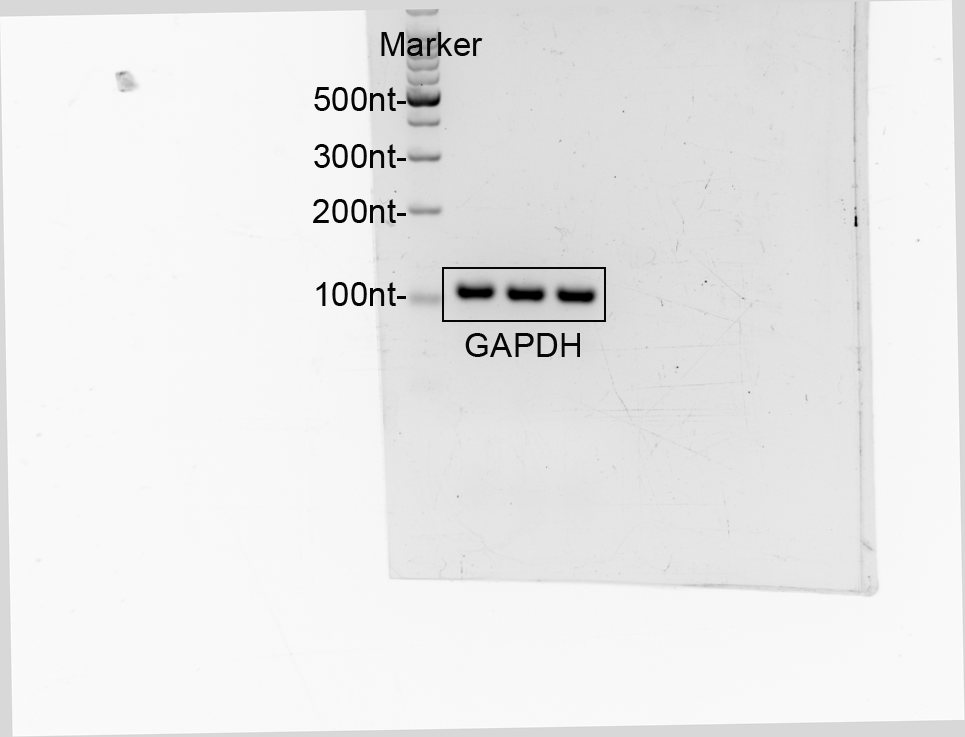

Supplement: Figure 6—figure supplement 1—source data 1. [file elife-95318-fig6-figsupp1-data1.zip › Figure6-figure supplement 1-Source data 1/Uncropped gels-Sup Fig 5B/BT549-GAPDH.tif]

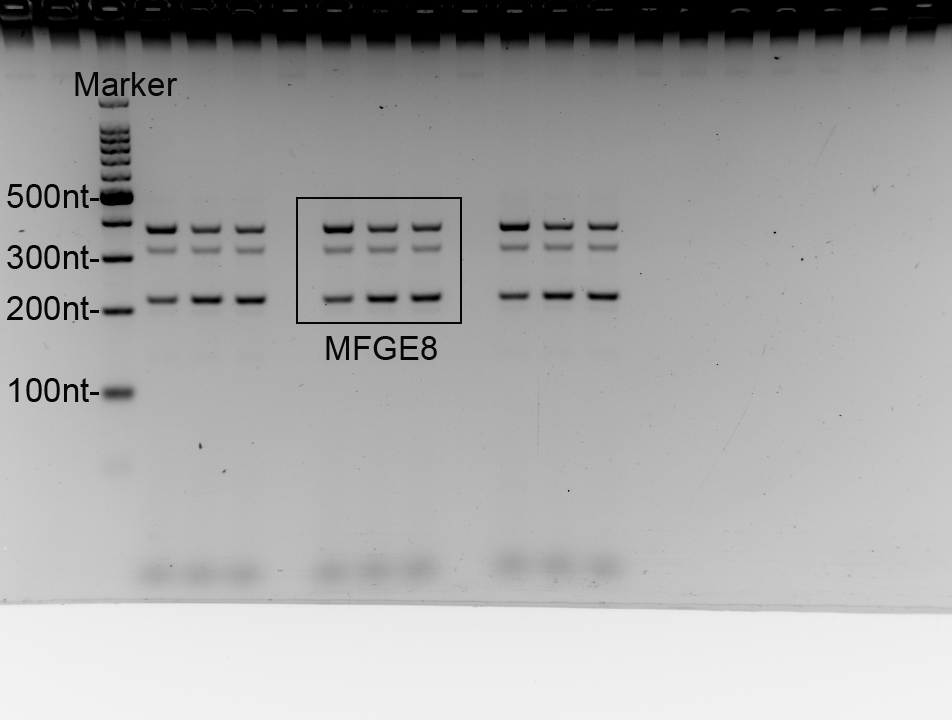

Supplement: Figure 6—figure supplement 1—source data 1. [file elife-95318-fig6-figsupp1-data1.zip › Figure6-figure supplement 1-Source data 1/Uncropped gels-Sup Fig 5B/BT549-MFGE8.tif]

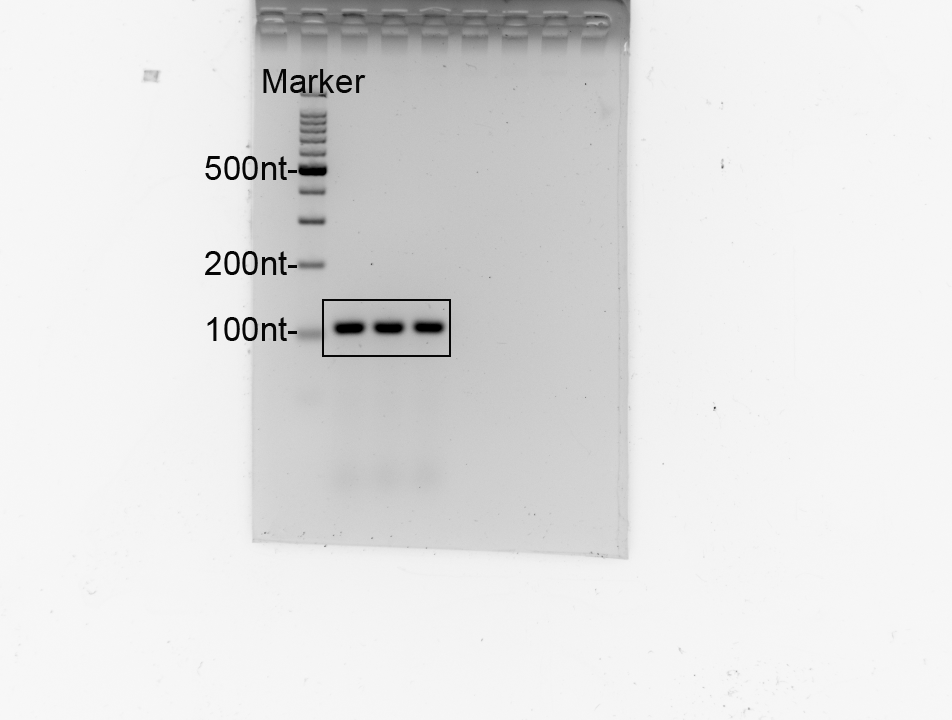

Supplement: Figure 6—figure supplement 1—source data 1. [file elife-95318-fig6-figsupp1-data1.zip › Figure6-figure supplement 1-Source data 1/Uncropped gels-Sup Fig 5B/HCC1937-GAPDH.tif]

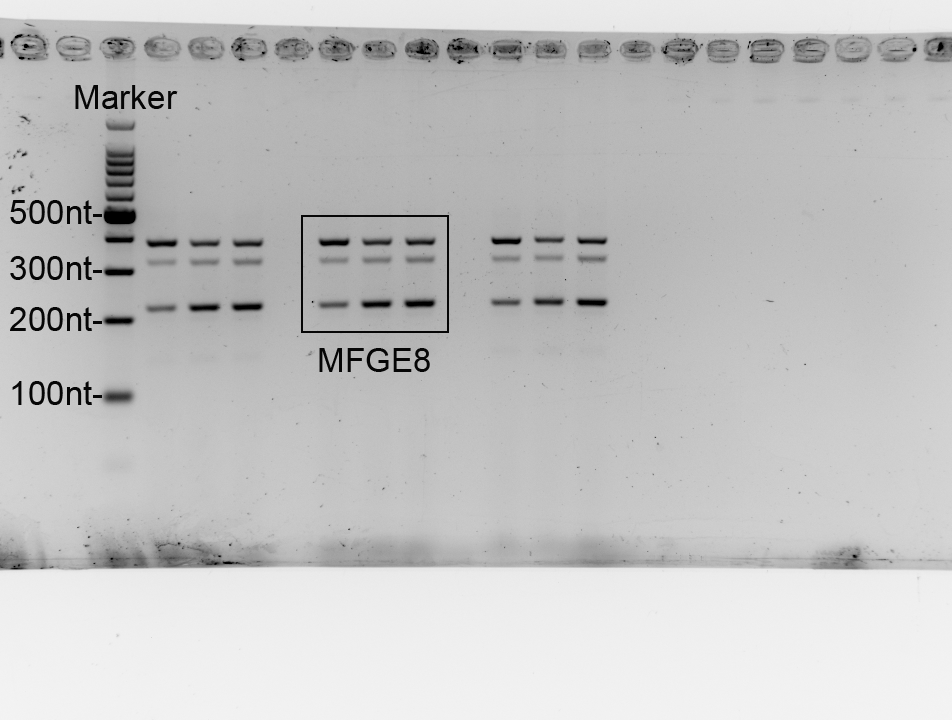

Supplement: Figure 6—figure supplement 1—source data 1. [file elife-95318-fig6-figsupp1-data1.zip › Figure6-figure supplement 1-Source data 1/Uncropped gels-Sup Fig 5B/HCC1937-MFGE8.tif]

# Sup Figure5B

|              | BT-549 |   |   | HCC1937 |   |   |
|--------------|--------|---|---|---------|---|---|
| Scramble-ASO | +      | - | - | +       | - | - |
| MFGE8-ASO-1  | -      | + | - | -       | + | - |
| MFGE8-ASO-2  | -      | - | + | -       | - | + |

  

|       |  |  |
|-------|--|--|
| MFGE8 |  |  |
| GAPDH |  |  |

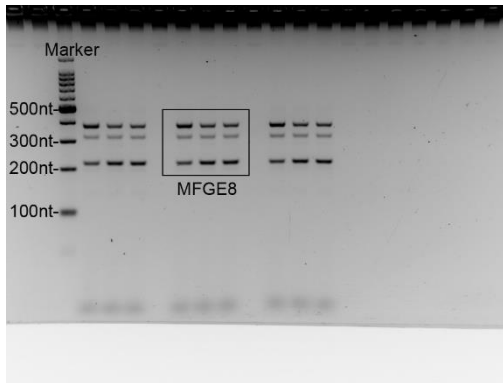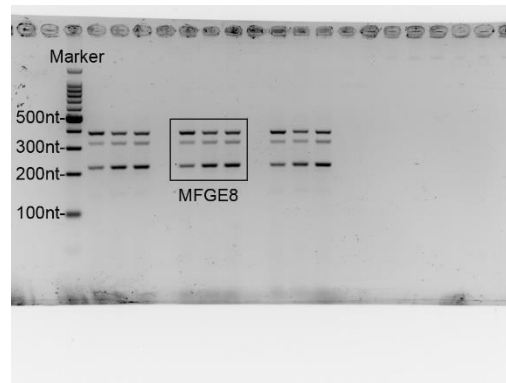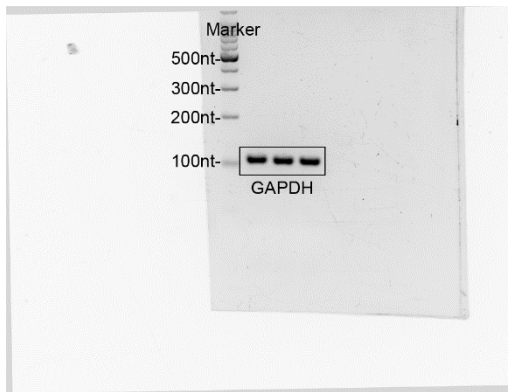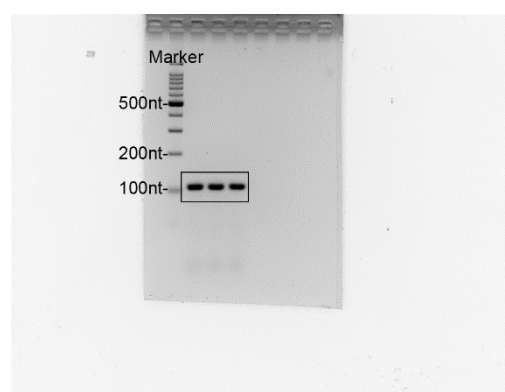

Supplement: Figure 6—figure supplement 1—source data 2. [file elife-95318-fig6-figsupp1-data2.zip › Figure6-figure supplement 1-Source data 2/Sup Fig5B.pdf]

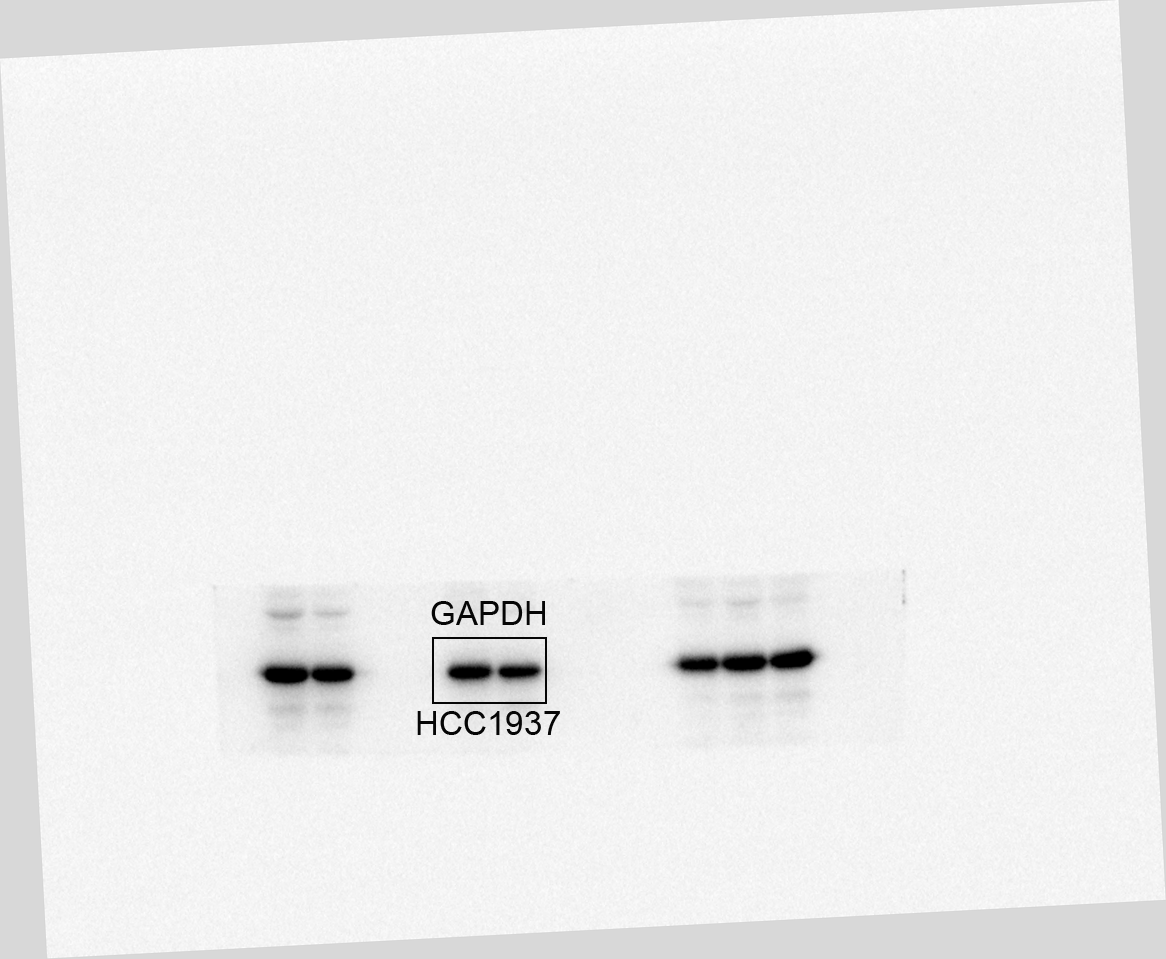

Supplement: Figure 6—figure supplement 1—source data 3. [file elife-95318-fig6-figsupp1-data3.zip › Figure6-figure supplement 1-Source data 3/Uncropped blots-Sup Fig5E/HCC1937-GAPDH.tif]

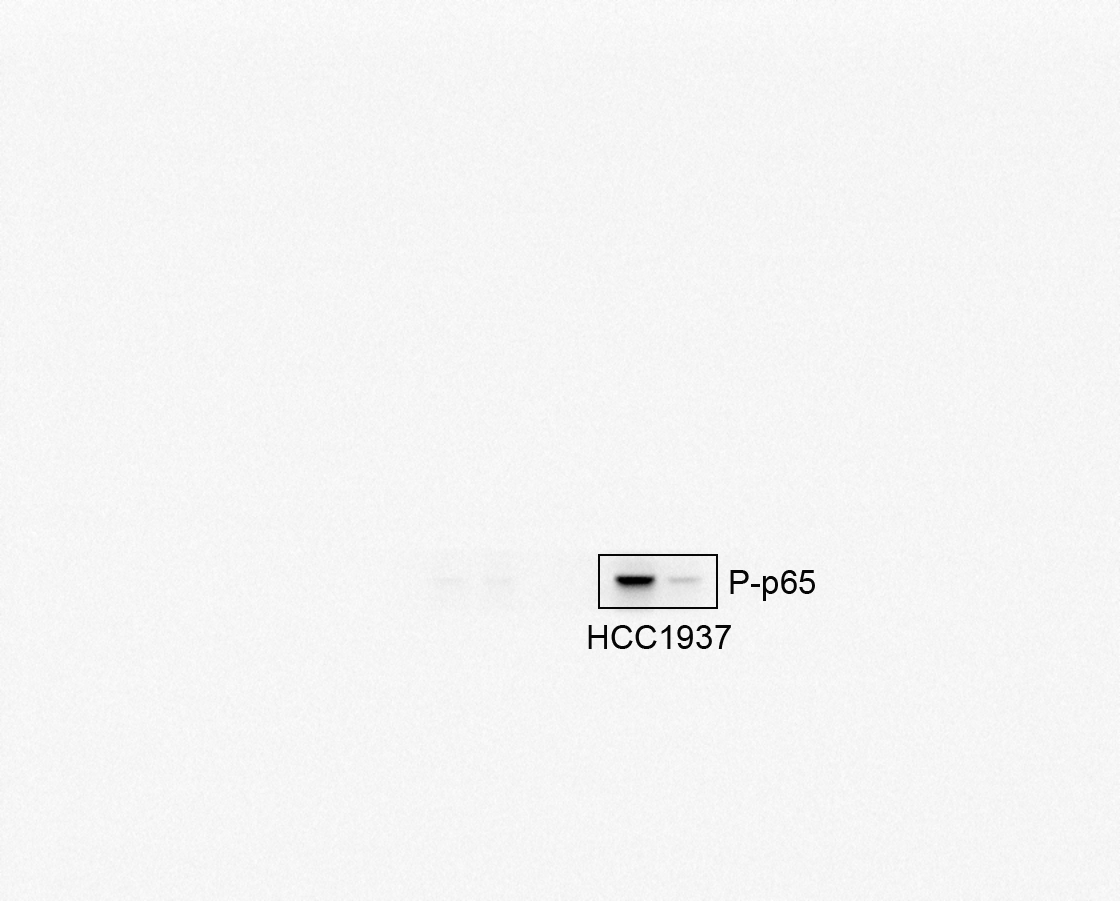

Supplement: Figure 6—figure supplement 1—source data 3. [file elife-95318-fig6-figsupp1-data3.zip › Figure6-figure supplement 1-Source data 3/Uncropped blots-Sup Fig5E/HCC1937-P-p65.tif]

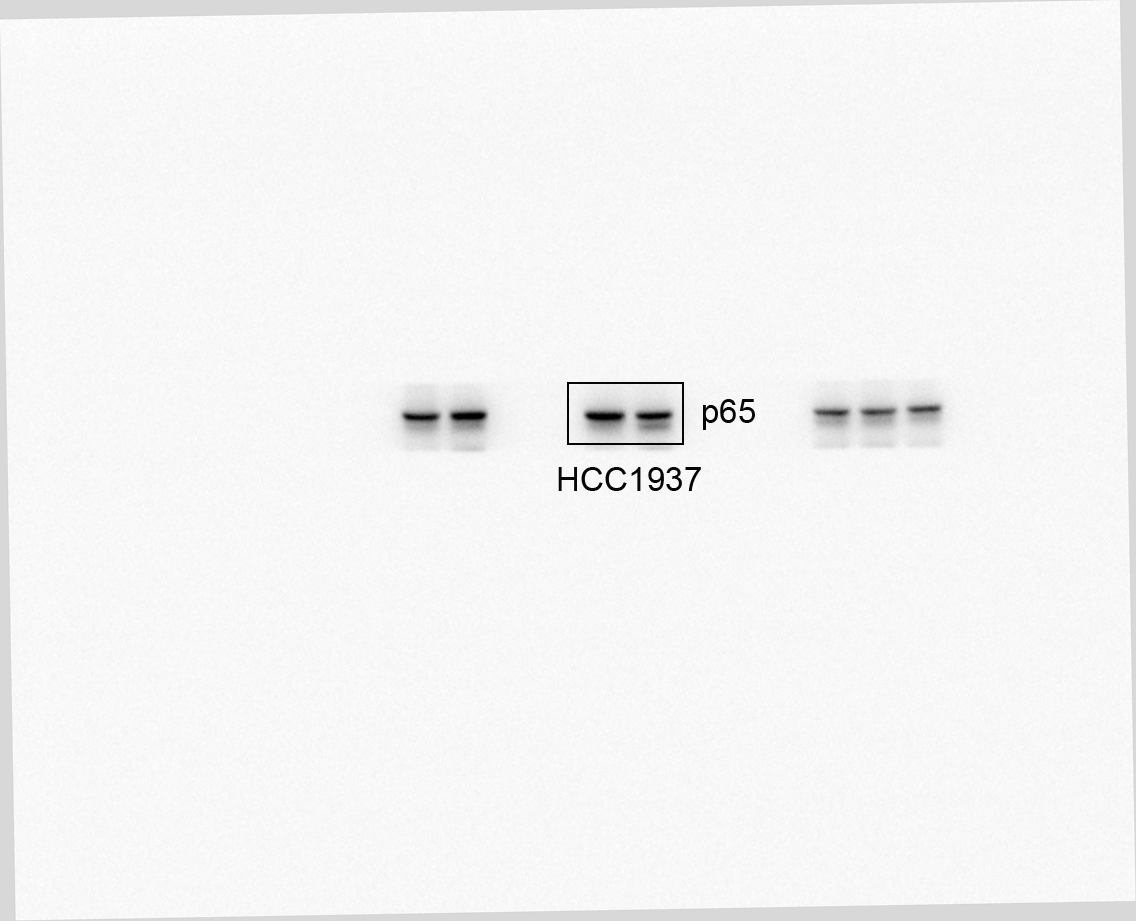

Supplement: Figure 6—figure supplement 1—source data 3. [file elife-95318-fig6-figsupp1-data3.zip › Figure6-figure supplement 1-Source data 3/Uncropped blots-Sup Fig5E/HCC1937-p65.tif]

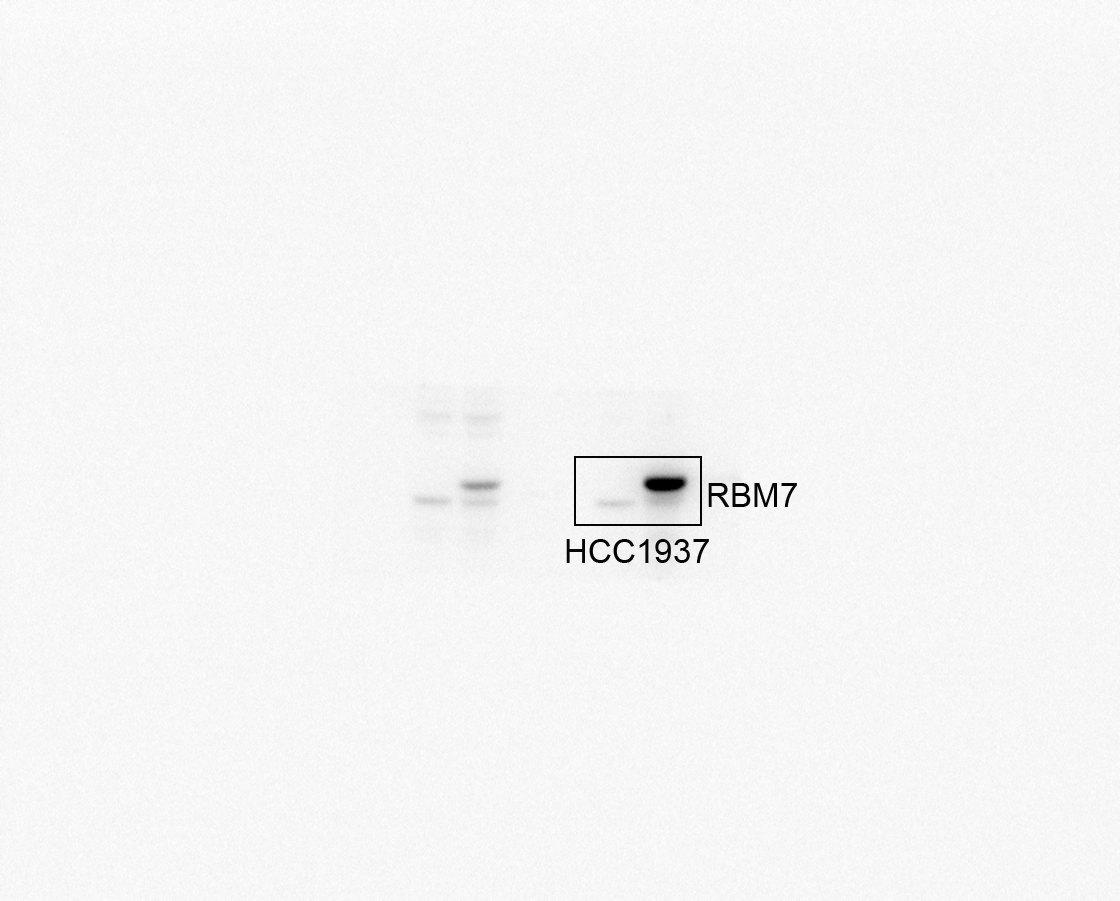

Supplement: Figure 6—figure supplement 1—source data 3. [file elife-95318-fig6-figsupp1-data3.zip › Figure6-figure supplement 1-Source data 3/Uncropped blots-Sup Fig5E/HCC1937-RBM7.tif]

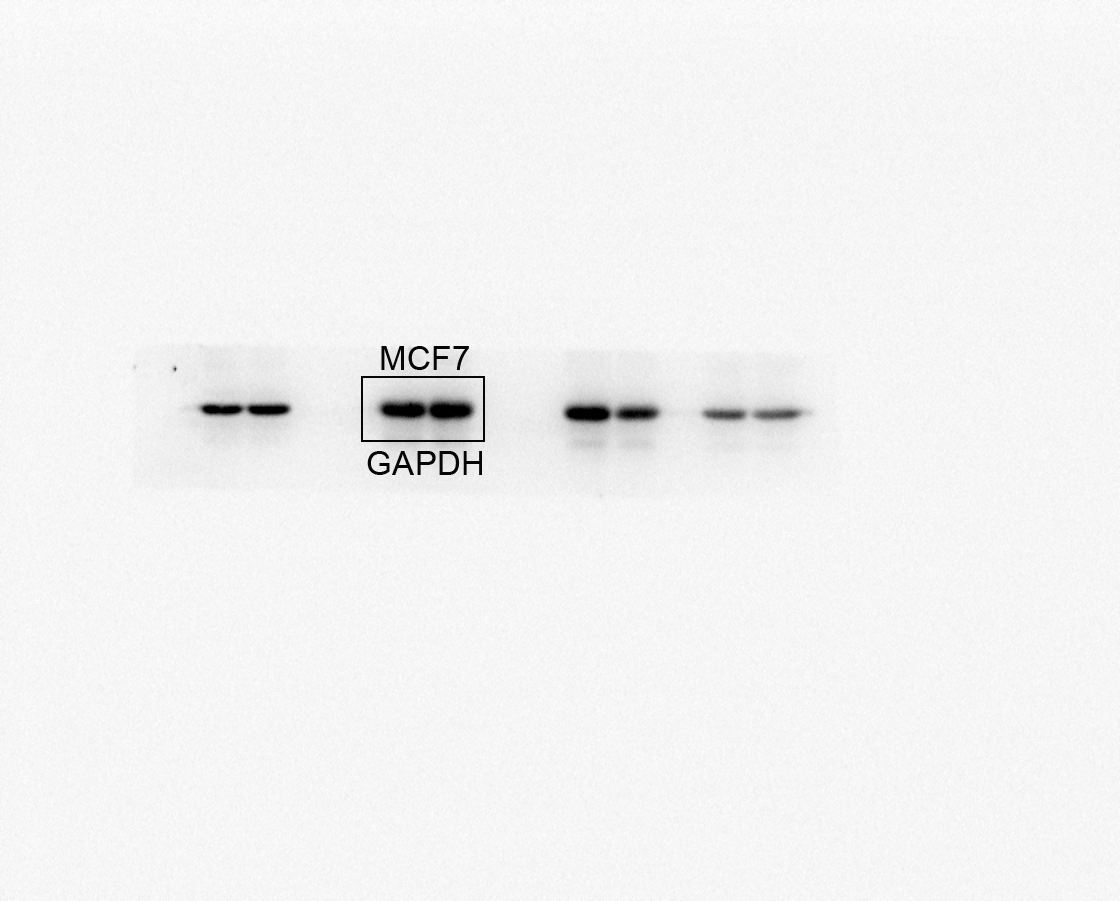

Supplement: Figure 6—figure supplement 1—source data 3. [file elife-95318-fig6-figsupp1-data3.zip › Figure6-figure supplement 1-Source data 3/Uncropped blots-Sup Fig5E/MCF7-GAPDH.tif]

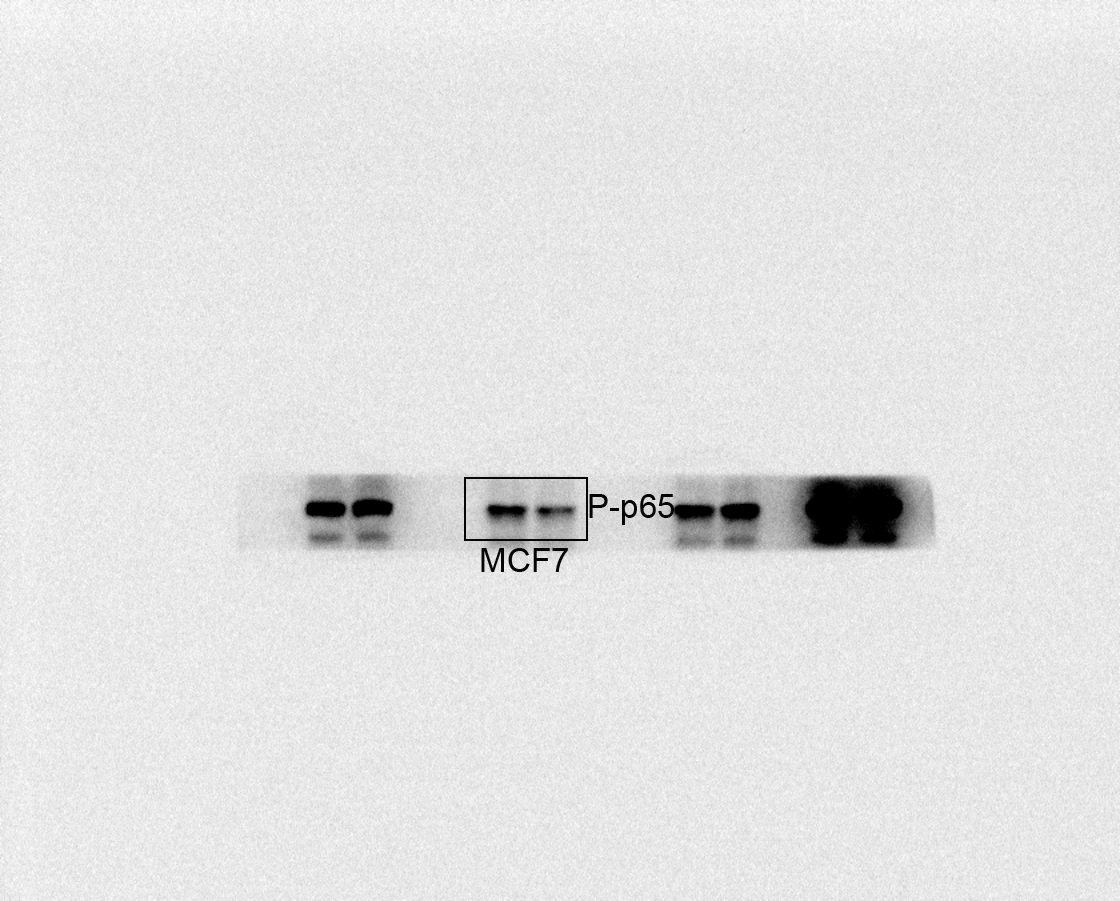

Supplement: Figure 6—figure supplement 1—source data 3. [file elife-95318-fig6-figsupp1-data3.zip › Figure6-figure supplement 1-Source data 3/Uncropped blots-Sup Fig5E/MCF7-P-p65.tif]

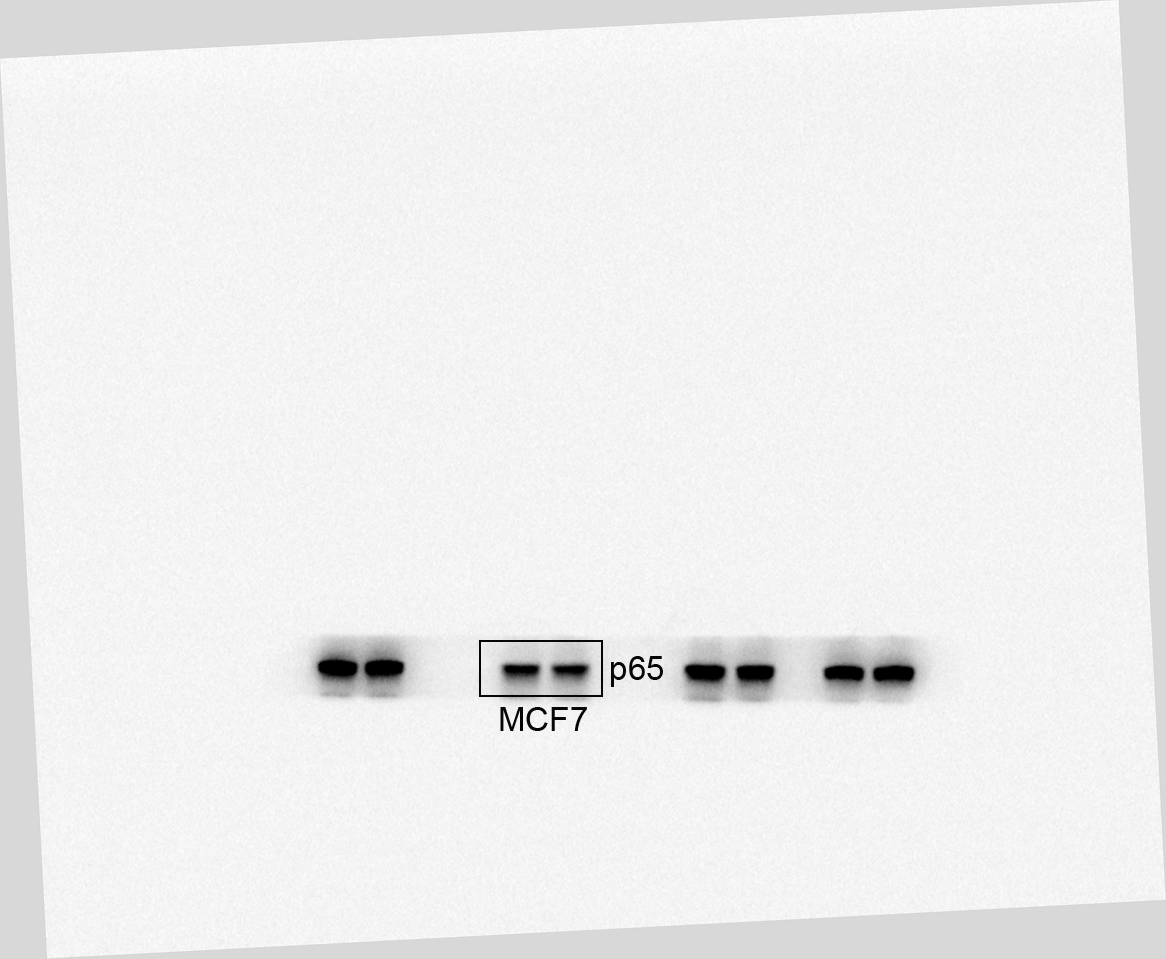

Supplement: Figure 6—figure supplement 1—source data 3. [file elife-95318-fig6-figsupp1-data3.zip › Figure6-figure supplement 1-Source data 3/Uncropped blots-Sup Fig5E/MCF7-p65.tif]

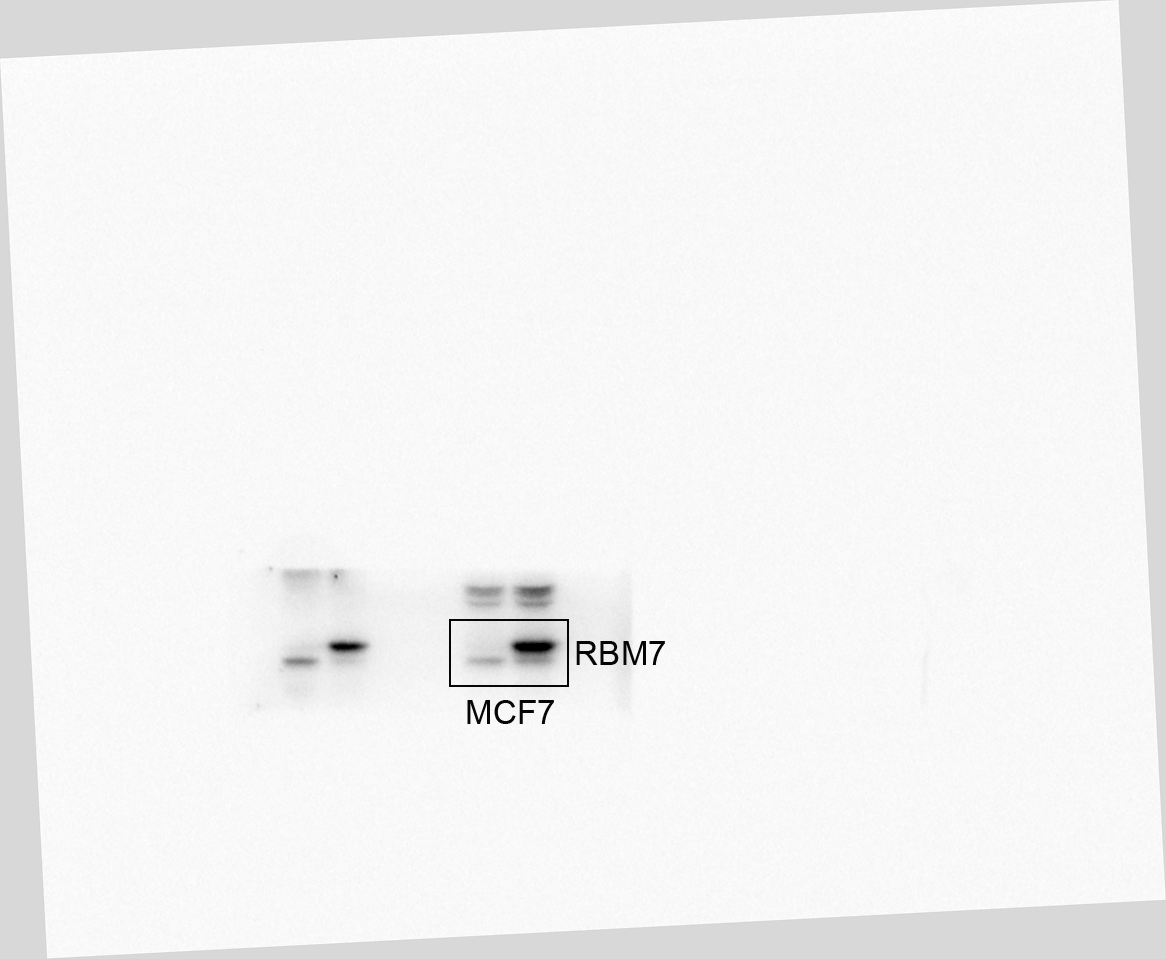

Supplement: Figure 6—figure supplement 1—source data 3. [file elife-95318-fig6-figsupp1-data3.zip › Figure6-figure supplement 1-Source data 3/Uncropped blots-Sup Fig5E/MCF7-RBM7.tif]

Sup Figure 5E

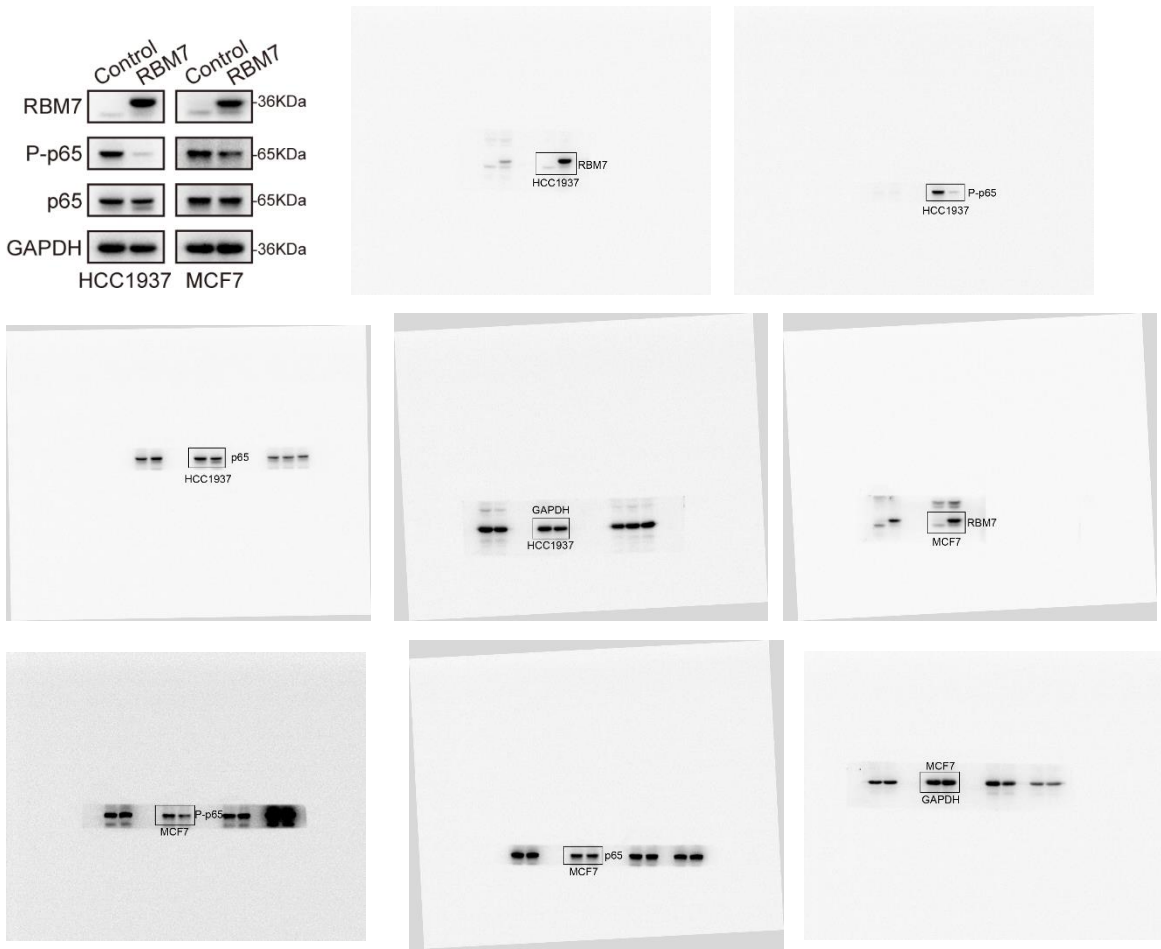

Supplement: Figure 6—figure supplement 1—source data 4. [file elife-95318-fig6-figsupp1-data4.zip › Figure6-figure supplement 1-Source data 4/Sup Fig5E.pdf]

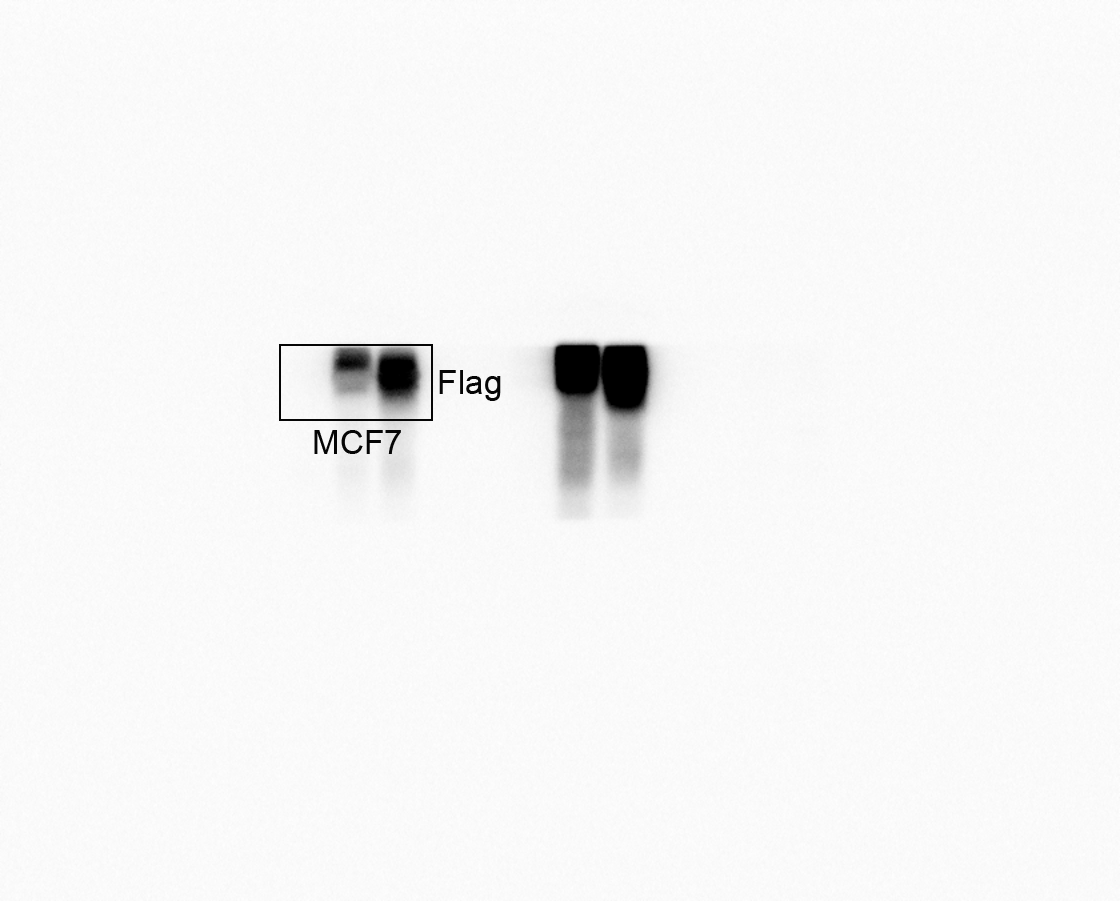

Supplement: Figure 6—figure supplement 1—source data 5. [file elife-95318-fig6-figsupp1-data5.zip › Figure6-figure supplement 1-Source data 5/Uncropped blots-Sup Fig5F/MCF7/MCF7-Flag.tif]

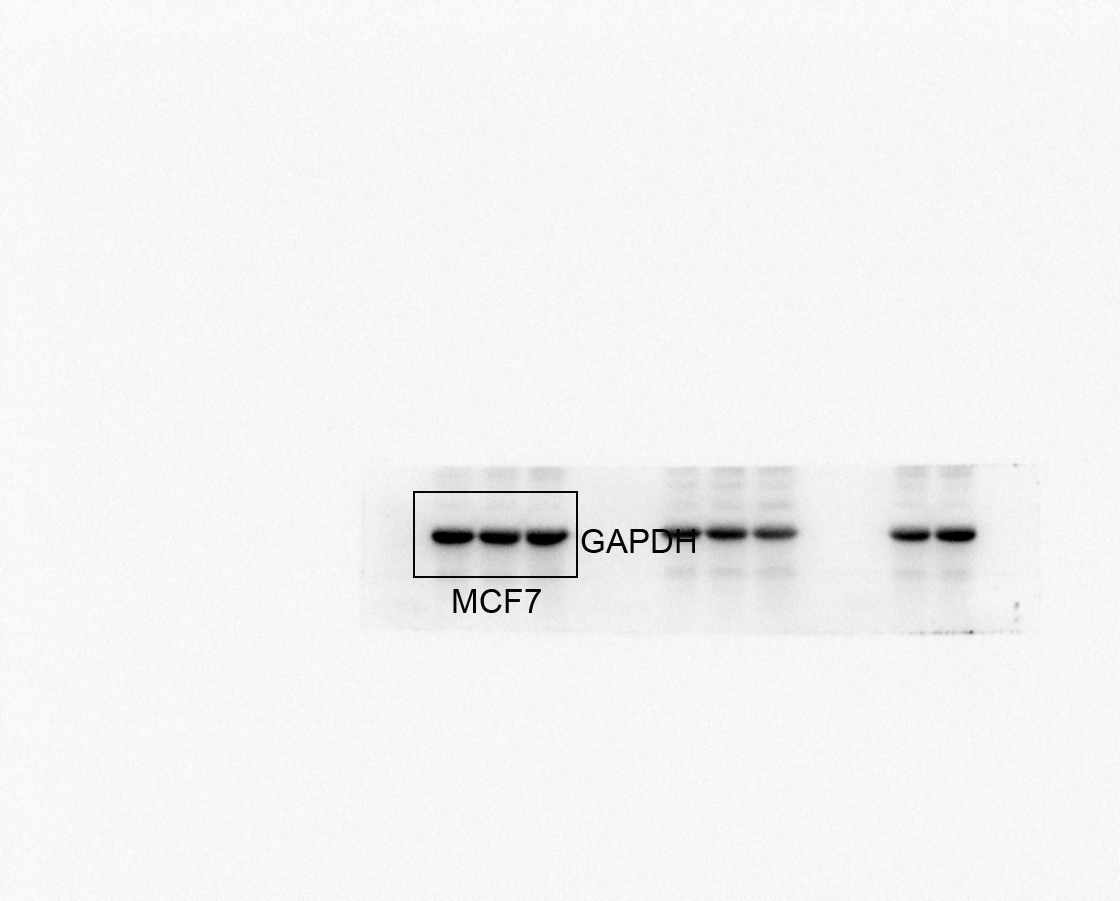

Supplement: Figure 6—figure supplement 1—source data 5. [file elife-95318-fig6-figsupp1-data5.zip › Figure6-figure supplement 1-Source data 5/Uncropped blots-Sup Fig5F/MCF7/MCF7-GAPDH.tif]

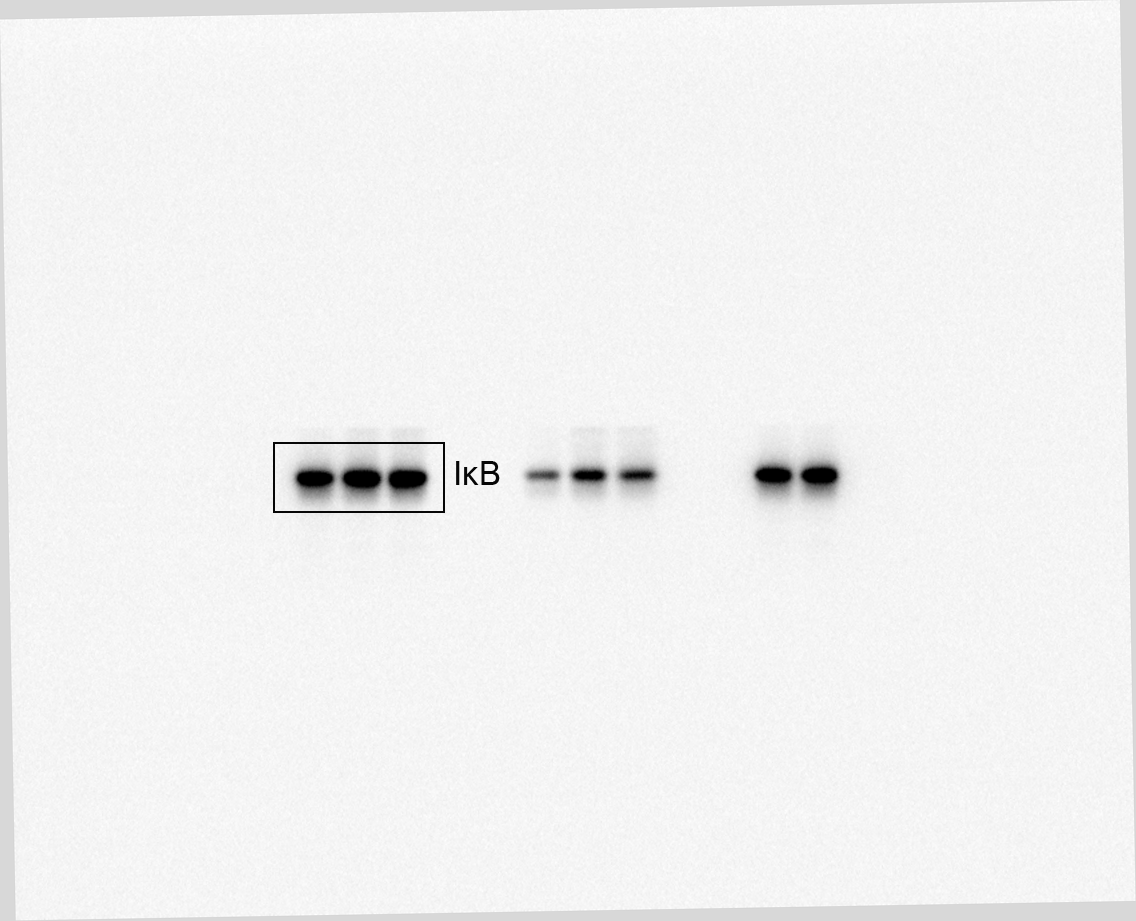

Supplement: Figure 6—figure supplement 1—source data 5. [file elife-95318-fig6-figsupp1-data5.zip › Figure6-figure supplement 1-Source data 5/Uncropped blots-Sup Fig5F/MCF7/MCF7-IκB.tif]

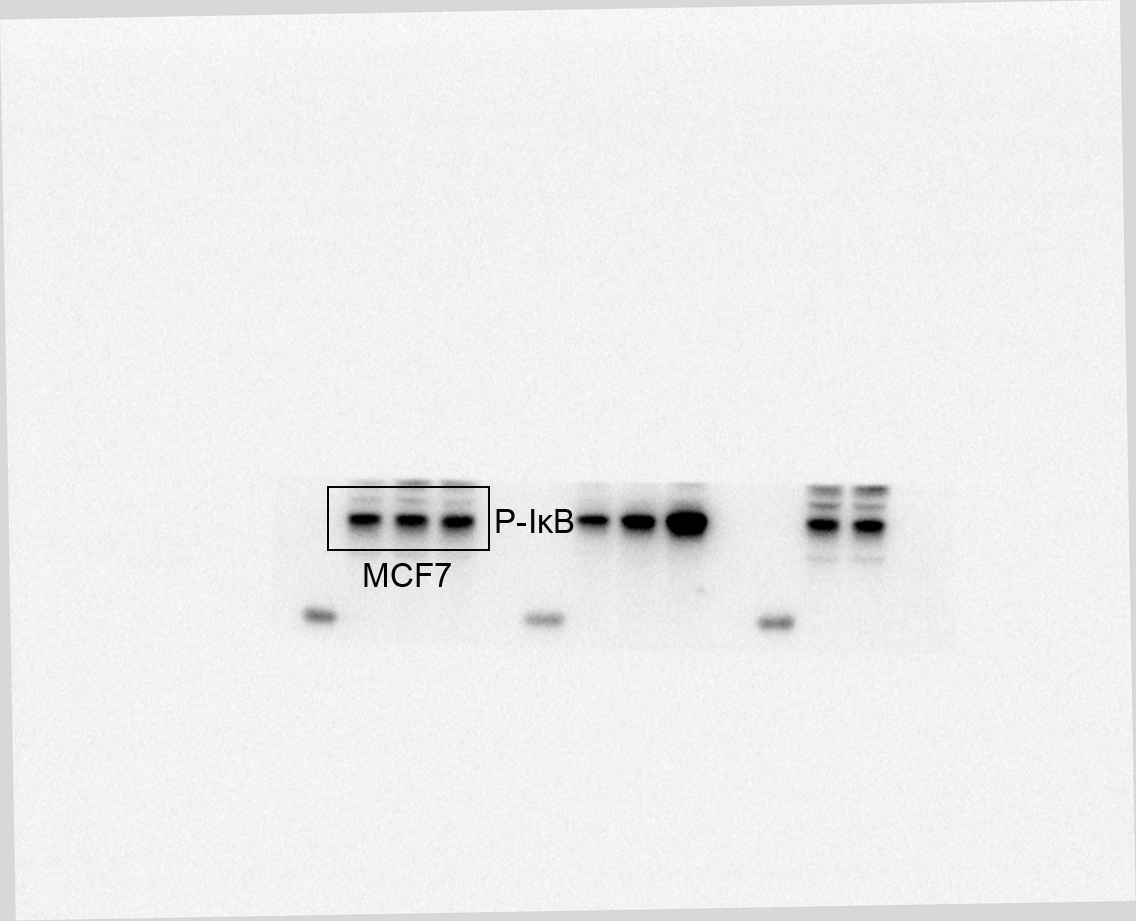

Supplement: Figure 6—figure supplement 1—source data 5. [file elife-95318-fig6-figsupp1-data5.zip › Figure6-figure supplement 1-Source data 5/Uncropped blots-Sup Fig5F/MCF7/MCF7-P-IκB.tif]

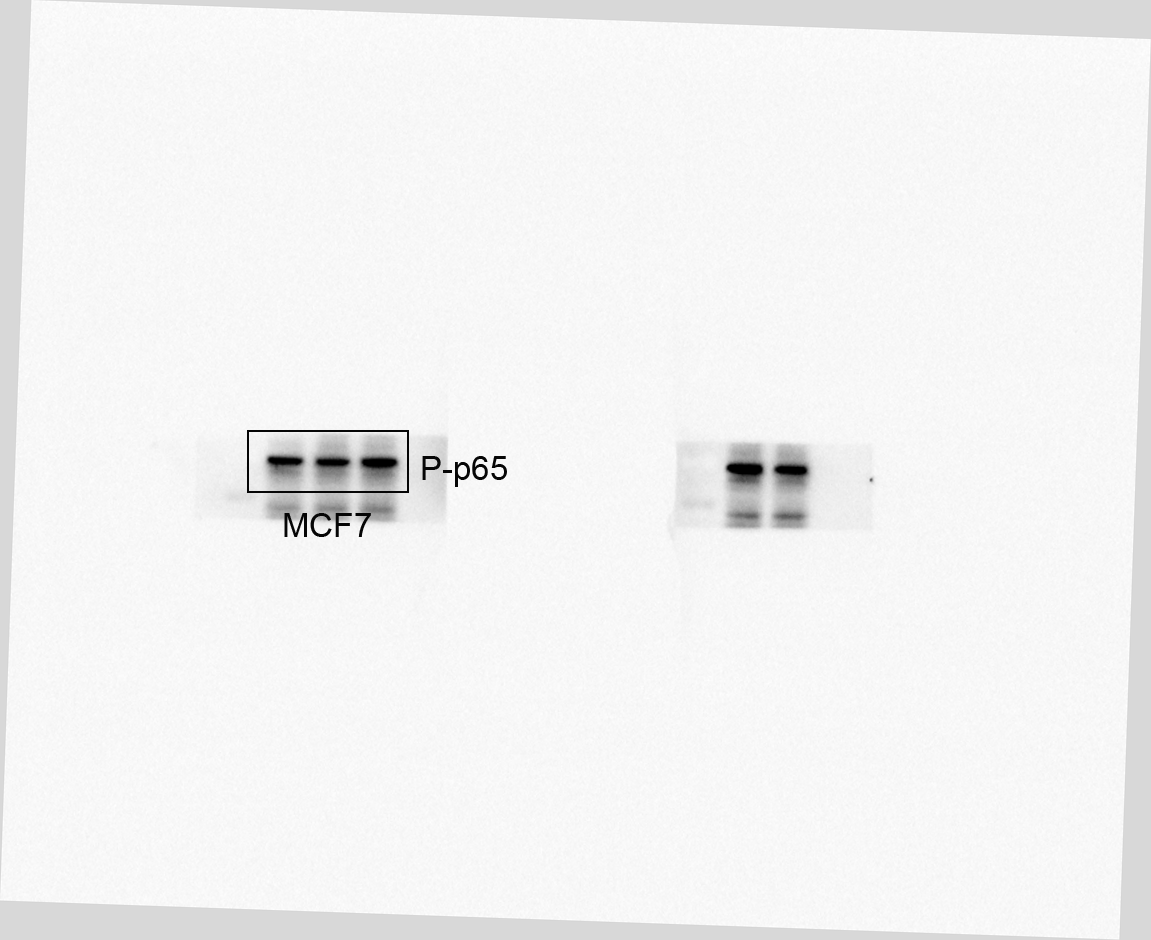

Supplement: Figure 6—figure supplement 1—source data 5. [file elife-95318-fig6-figsupp1-data5.zip › Figure6-figure supplement 1-Source data 5/Uncropped blots-Sup Fig5F/MCF7/MCF7-P-p65.tif]

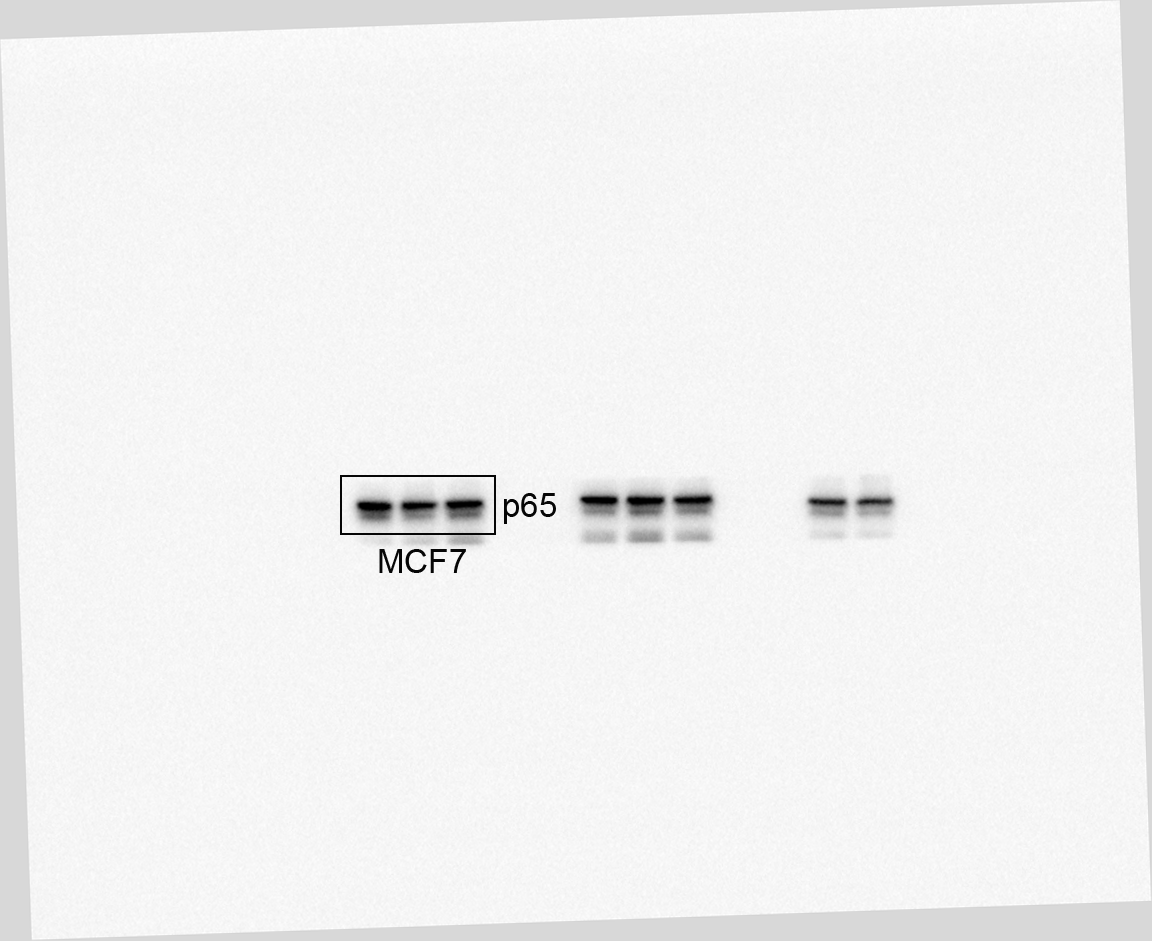

Supplement: Figure 6—figure supplement 1—source data 5. [file elife-95318-fig6-figsupp1-data5.zip › Figure6-figure supplement 1-Source data 5/Uncropped blots-Sup Fig5F/MCF7/MCF7-p65.tif]

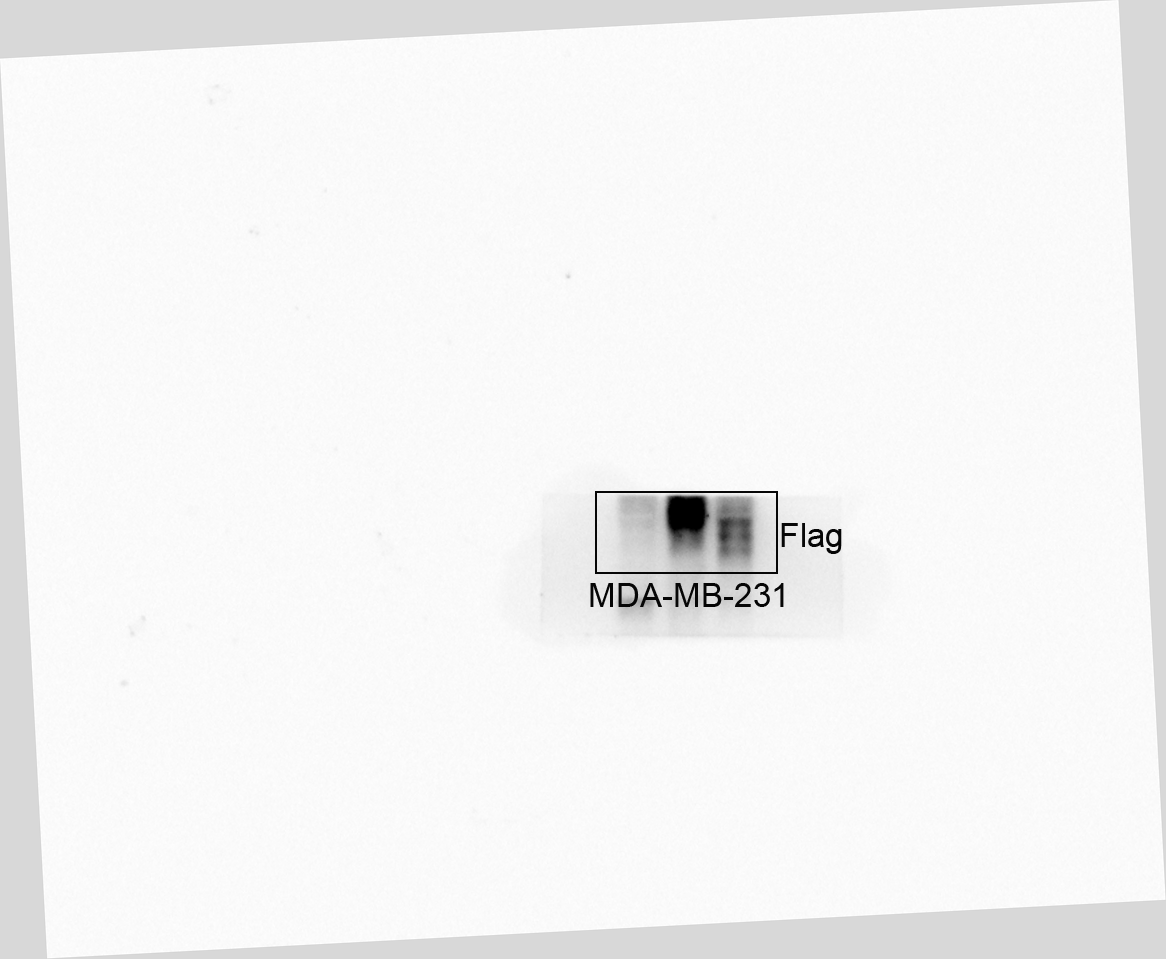

Supplement: Figure 6—figure supplement 1—source data 5. [file elife-95318-fig6-figsupp1-data5.zip › Figure6-figure supplement 1-Source data 5/Uncropped blots-Sup Fig5F/MDA-MB-231/MDA-MB-231 FLAG.tif]

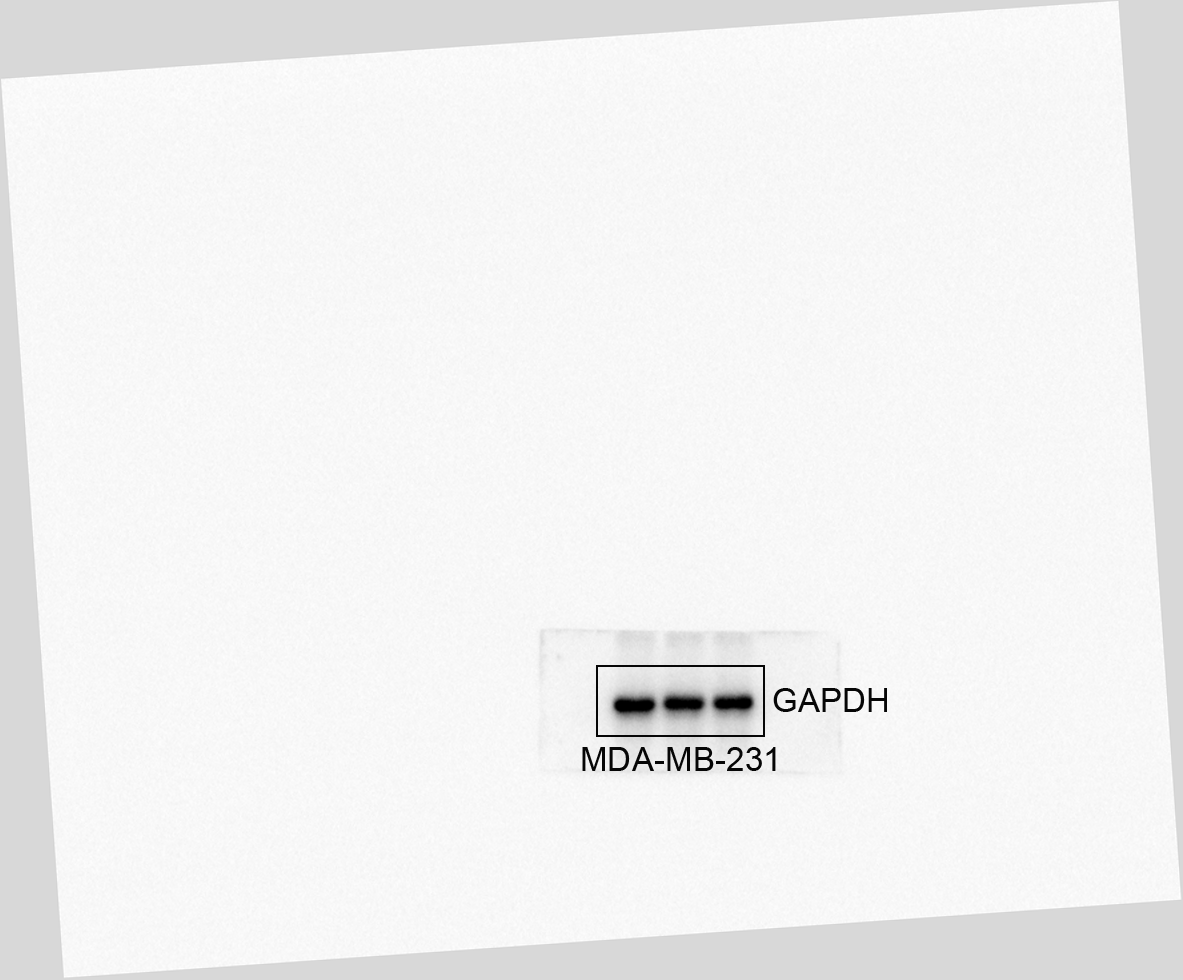

Supplement: Figure 6—figure supplement 1—source data 5. [file elife-95318-fig6-figsupp1-data5.zip › Figure6-figure supplement 1-Source data 5/Uncropped blots-Sup Fig5F/MDA-MB-231/MDA-MB-231 GAPDH.tif]

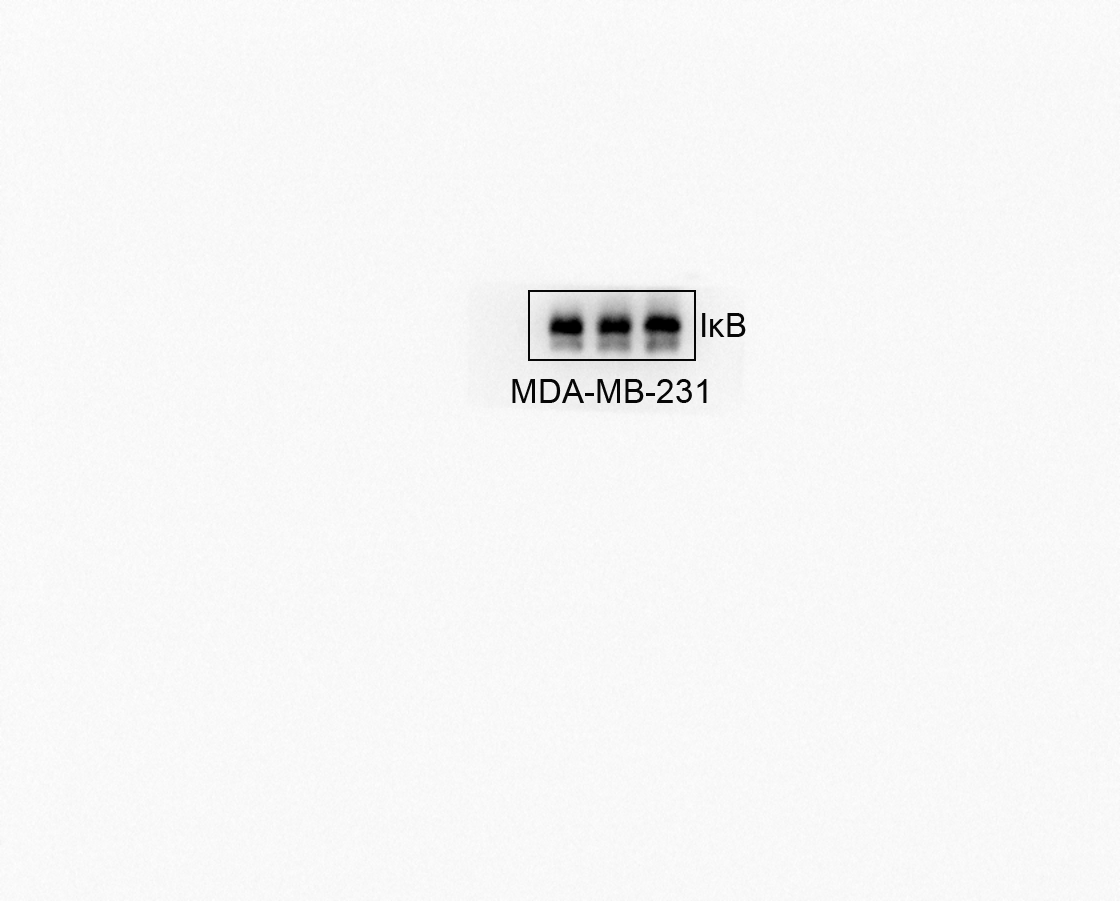

Supplement: Figure 6—figure supplement 1—source data 5. [file elife-95318-fig6-figsupp1-data5.zip › Figure6-figure supplement 1-Source data 5/Uncropped blots-Sup Fig5F/MDA-MB-231/MDA-MB-231 IκB.tif]

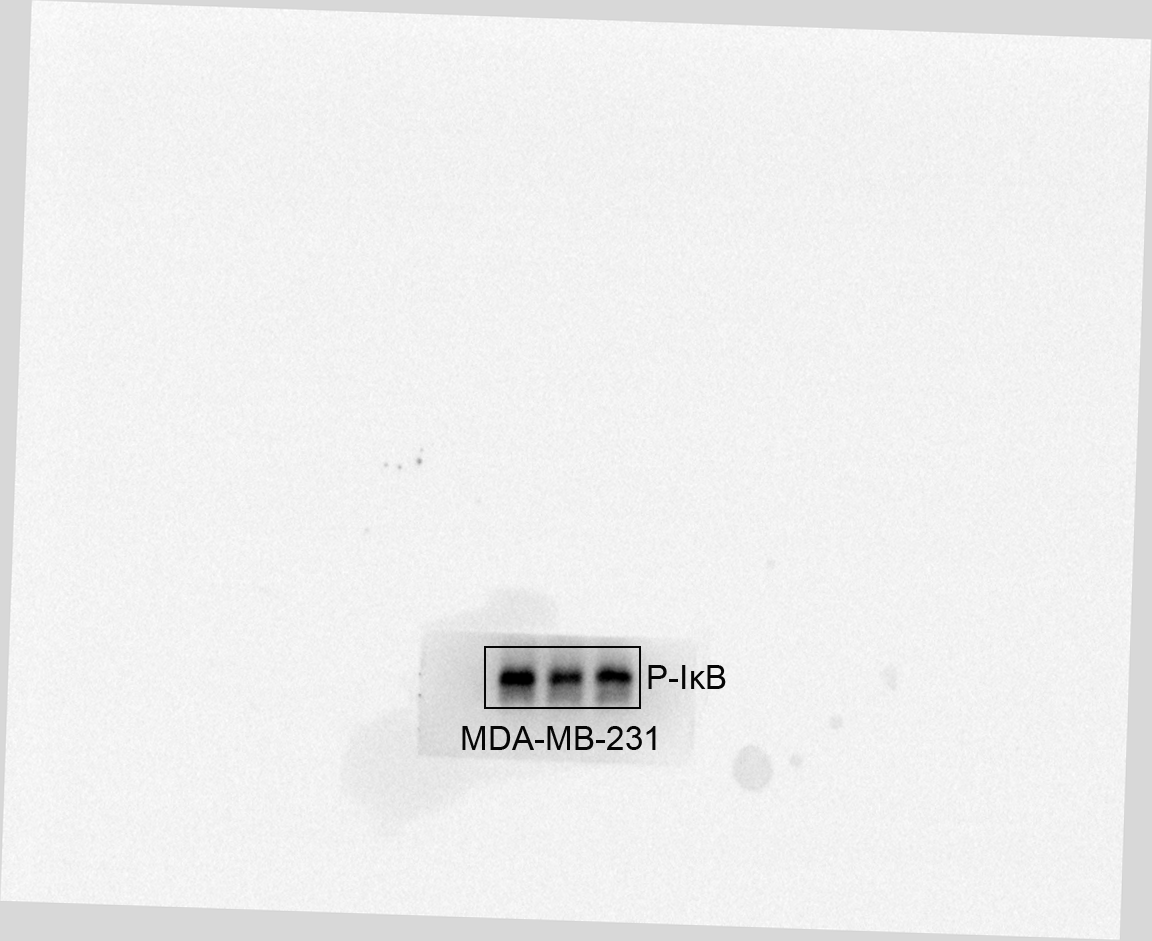

Supplement: Figure 6—figure supplement 1—source data 5. [file elife-95318-fig6-figsupp1-data5.zip › Figure6-figure supplement 1-Source data 5/Uncropped blots-Sup Fig5F/MDA-MB-231/MDA-MB-231 P-IκB.tif]

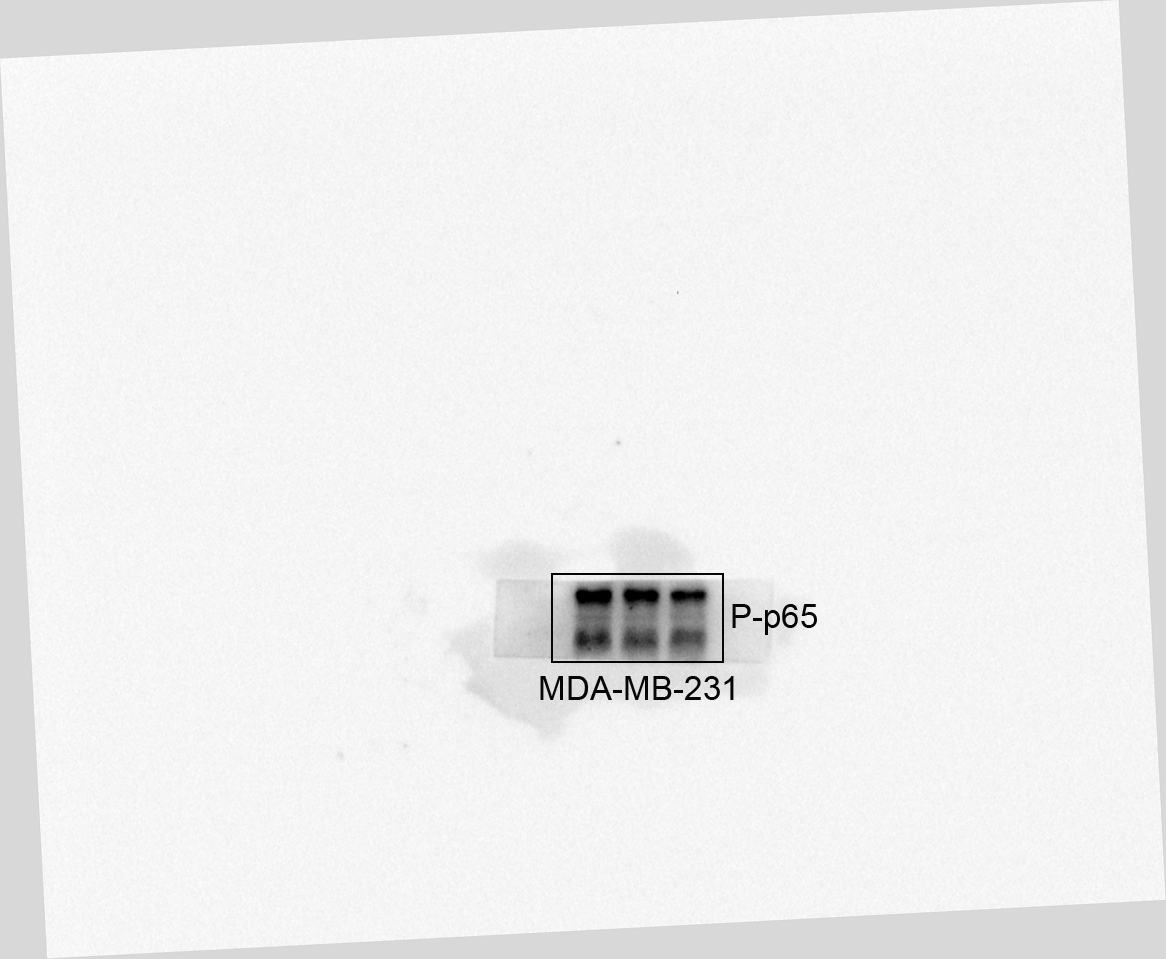

Supplement: Figure 6—figure supplement 1—source data 5. [file elife-95318-fig6-figsupp1-data5.zip › Figure6-figure supplement 1-Source data 5/Uncropped blots-Sup Fig5F/MDA-MB-231/MDA-MB-231 P-p65.tif]

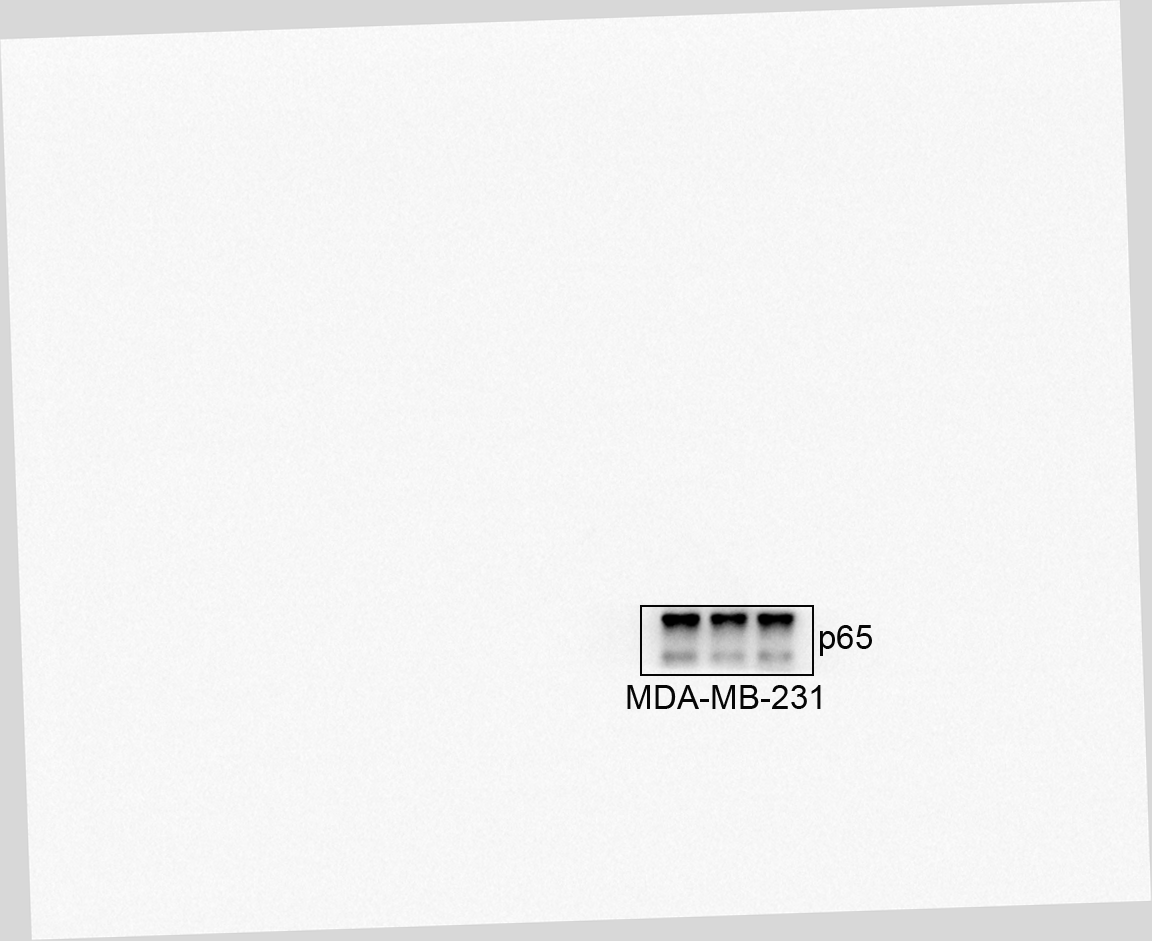

Supplement: Figure 6—figure supplement 1—source data 5. [file elife-95318-fig6-figsupp1-data5.zip › Figure6-figure supplement 1-Source data 5/Uncropped blots-Sup Fig5F/MDA-MB-231/MDA-MB-231 p65.tif]

Sup Figure 5F

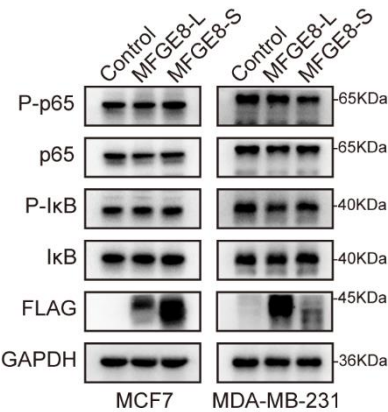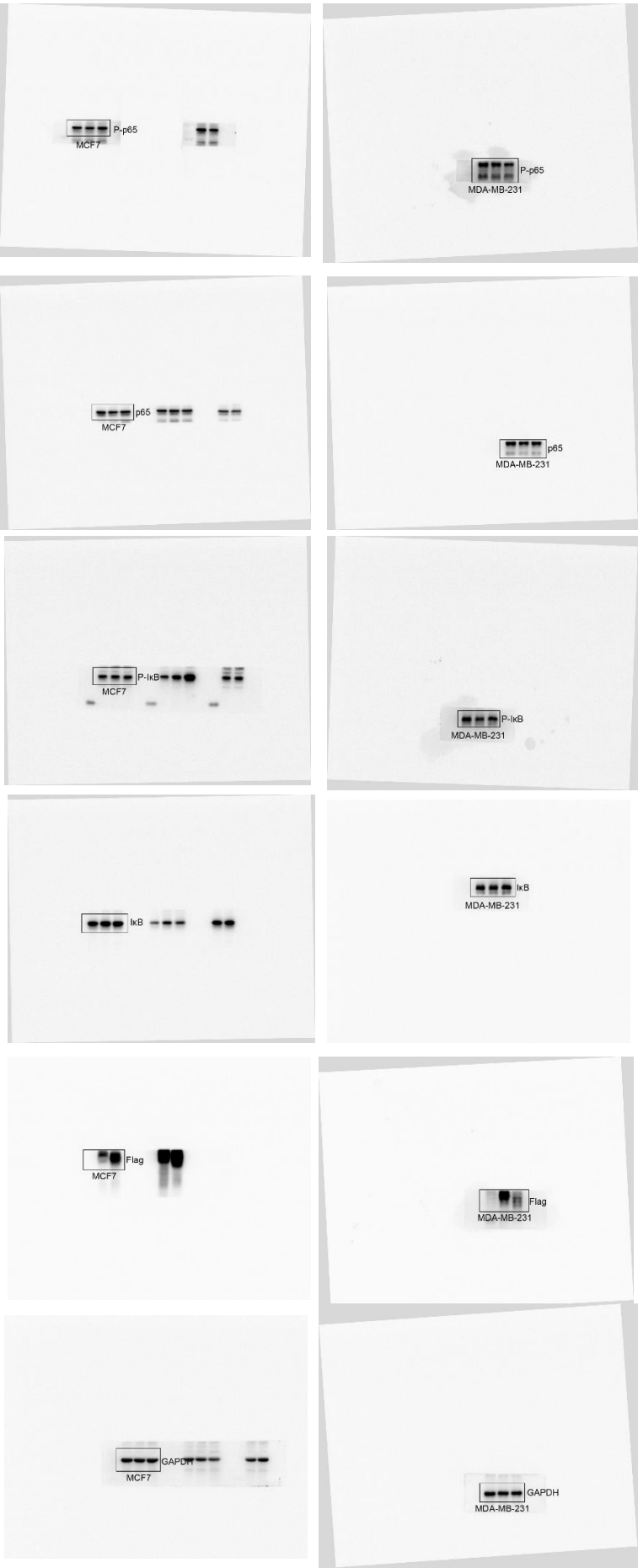

Supplement: Figure 6—figure supplement 1—source data 6. [file elife-95318-fig6-figsupp1-data6.zip › Figure6-figure supplement 1-Source data 6/Sup Fig5F.pdf]

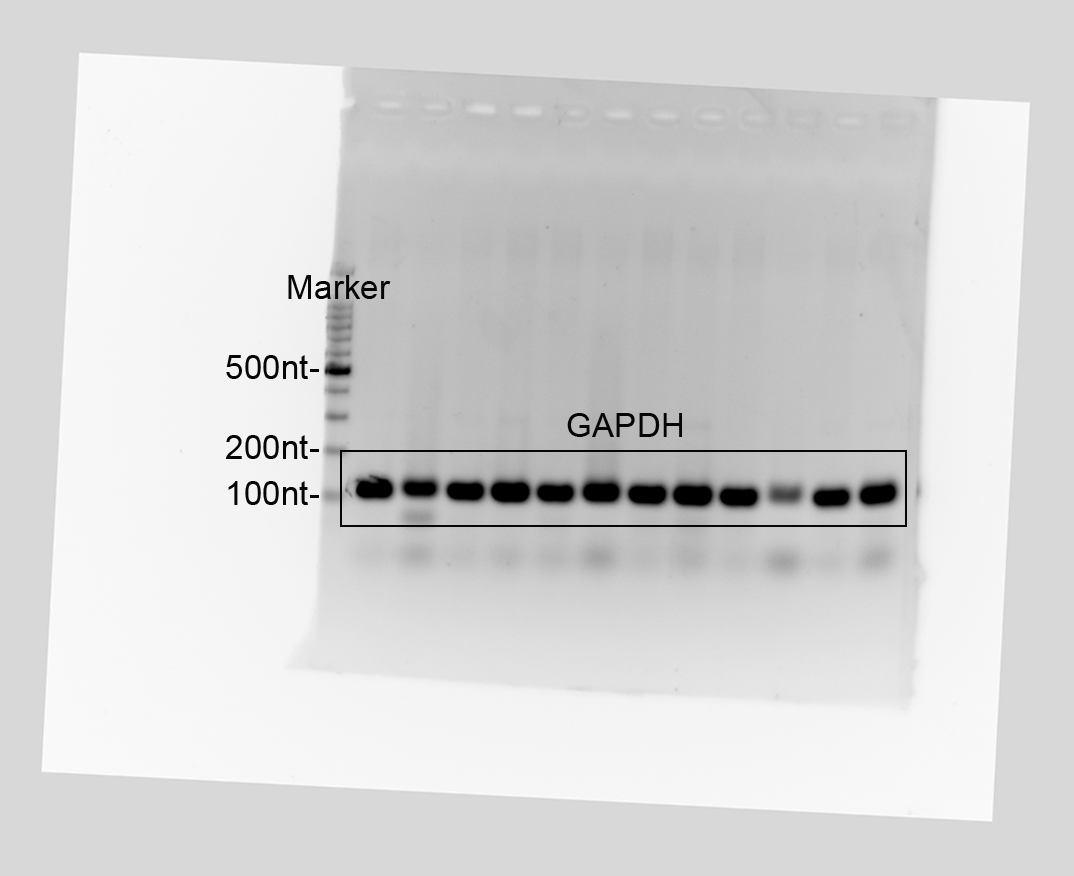

Supplement: Figure 7—source data 1. [file elife-95318-fig7-data1.zip › Figure7-Source data 1/Uncropped RT-PCR gels-Fig7B/GAPDH.tif]

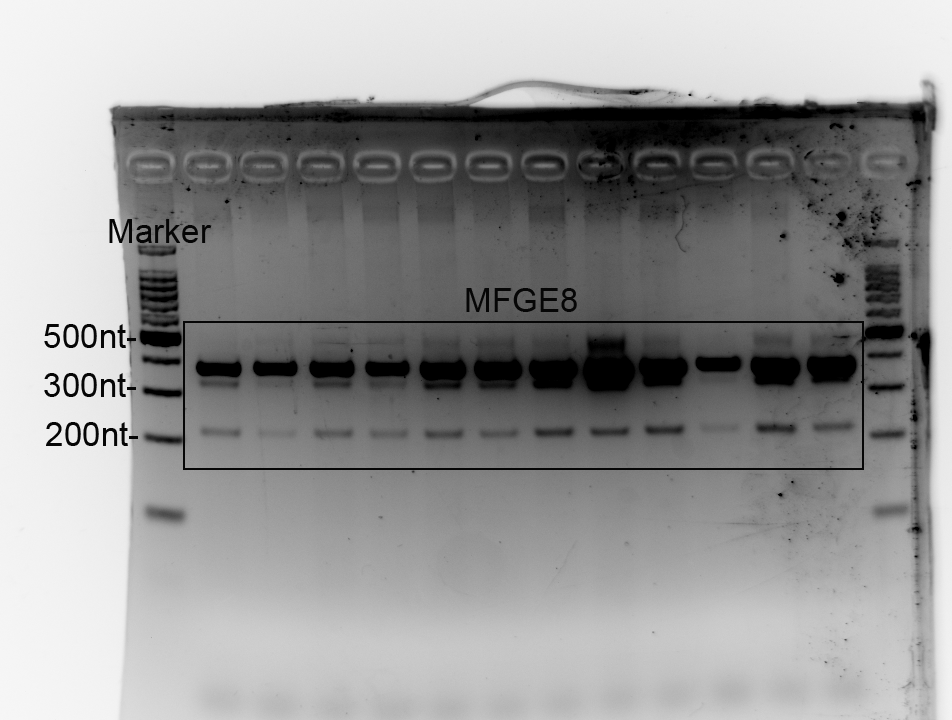

Supplement: Figure 7—source data 1. [file elife-95318-fig7-data1.zip › Figure7-Source data 1/Uncropped RT-PCR gels-Fig7B/MFGE8.tif]

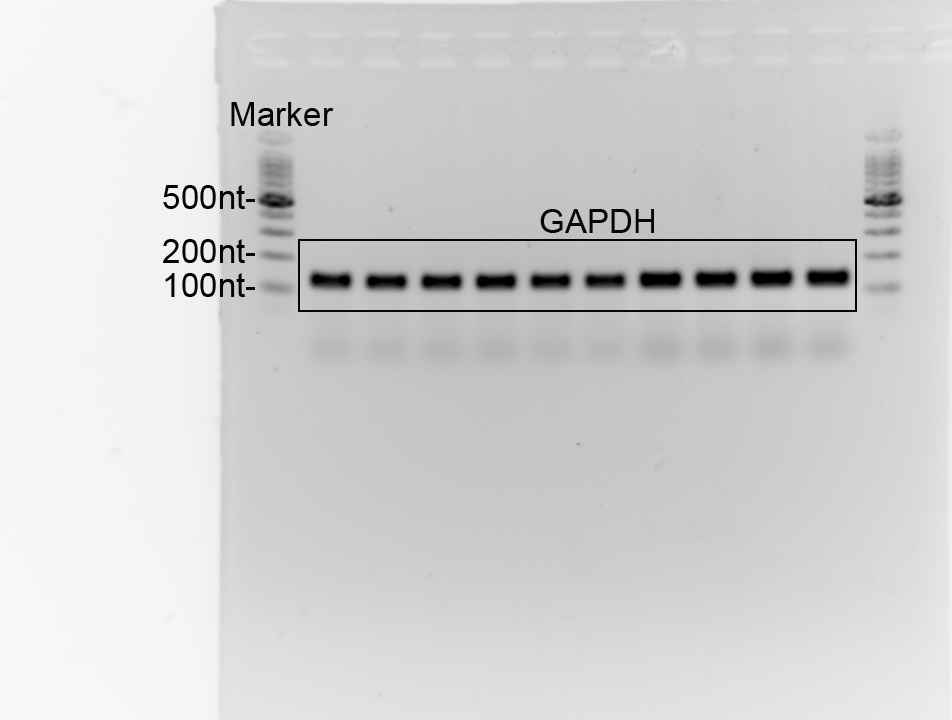

Supplement: Figure 7—source data 3. [file elife-95318-fig7-data3.zip › Figure7-Source data 3/Uncropped RT-PCR gels-Fig7C/GAPDH.tif]

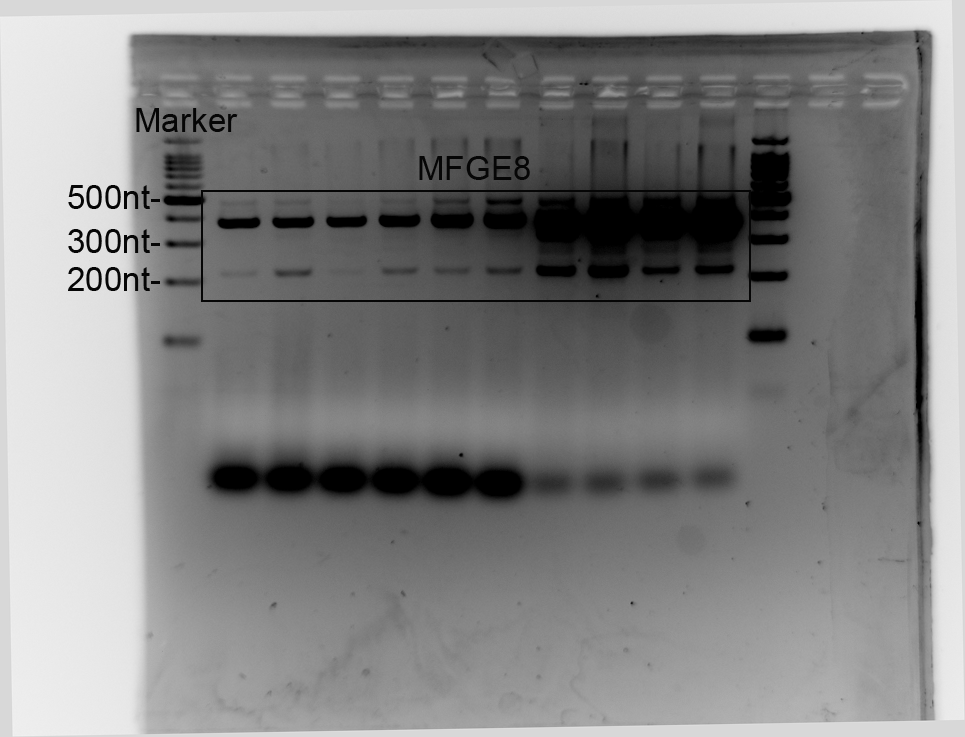

Supplement: Figure 7—source data 3. [file elife-95318-fig7-data3.zip › Figure7-Source data 3/Uncropped RT-PCR gels-Fig7C/MFGE8- LE.tif]

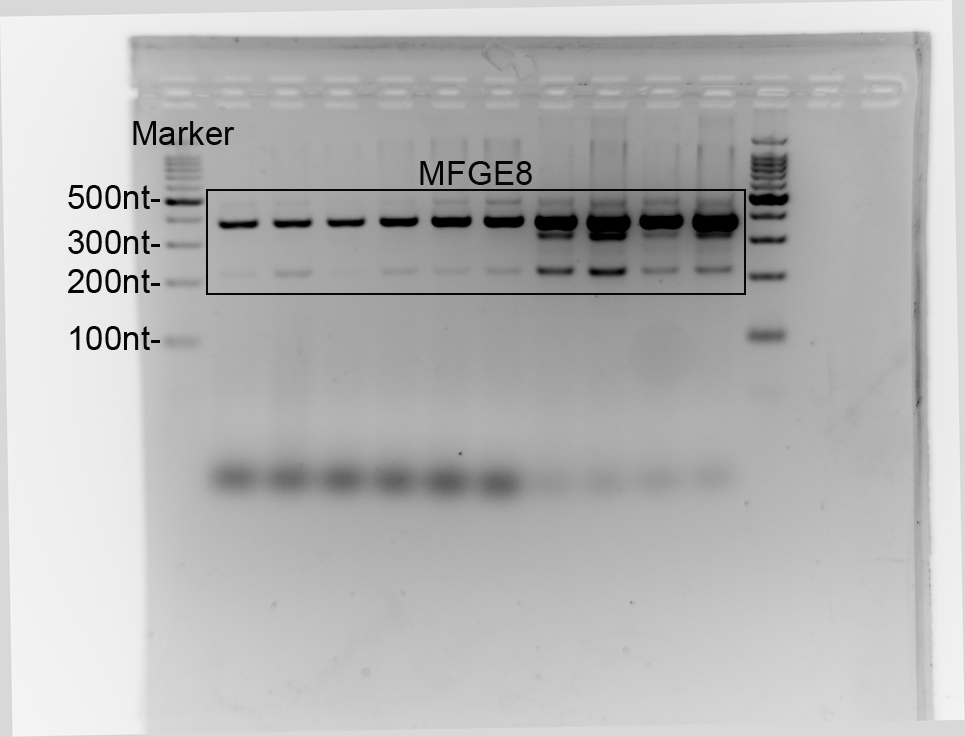

Supplement: Figure 7—source data 3. [file elife-95318-fig7-data3.zip › Figure7-Source data 3/Uncropped RT-PCR gels-Fig7C/MFGE8- SE.tif]

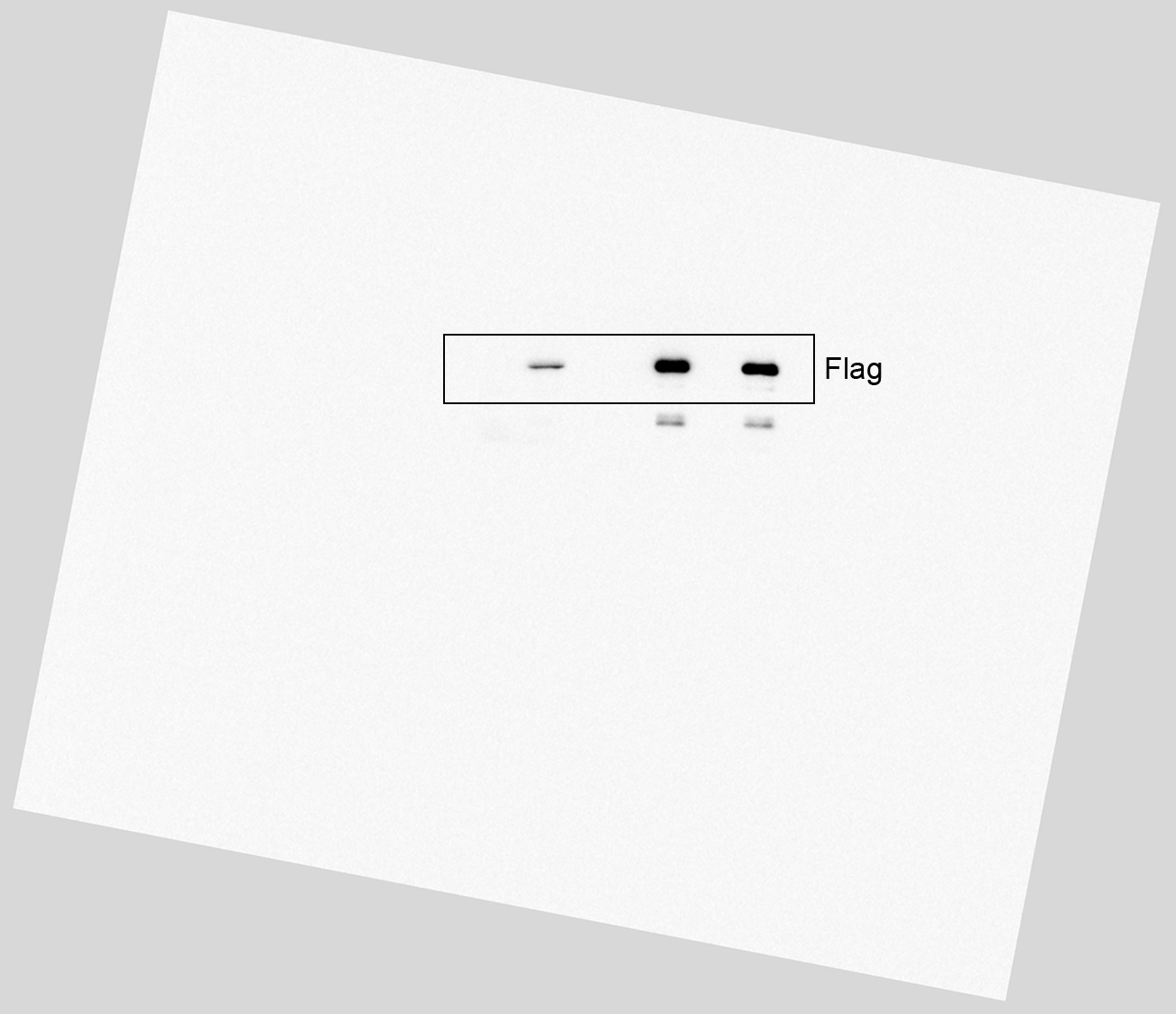

Supplement: Figure 7—figure supplement 1—source data 1. [file elife-95318-fig7-figsupp1-data1.zip › Figure7-figure supplement 1-Source data 1/Uncropped blots-Sup Fig6B/Flag.tif]

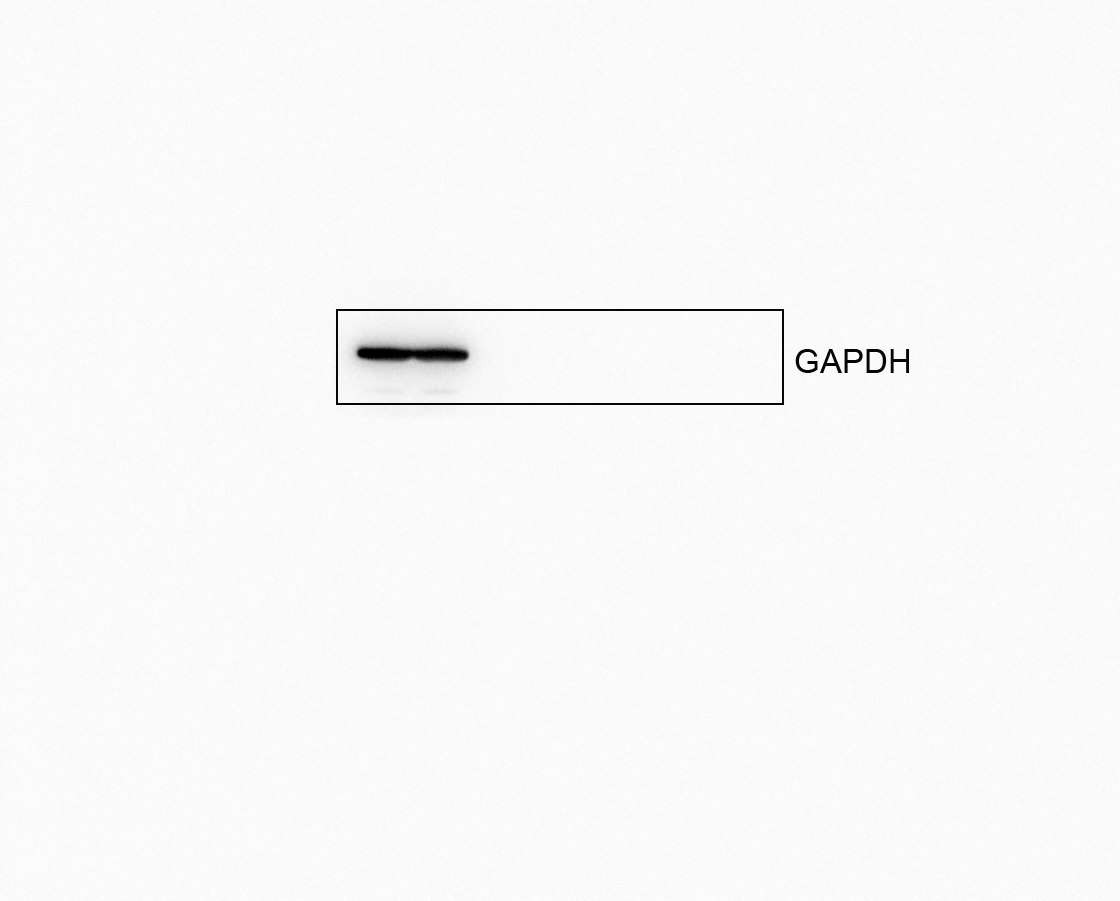

Supplement: Figure 7—figure supplement 1—source data 1. [file elife-95318-fig7-figsupp1-data1.zip › Figure7-figure supplement 1-Source data 1/Uncropped blots-Sup Fig6B/GAPDH.tif]

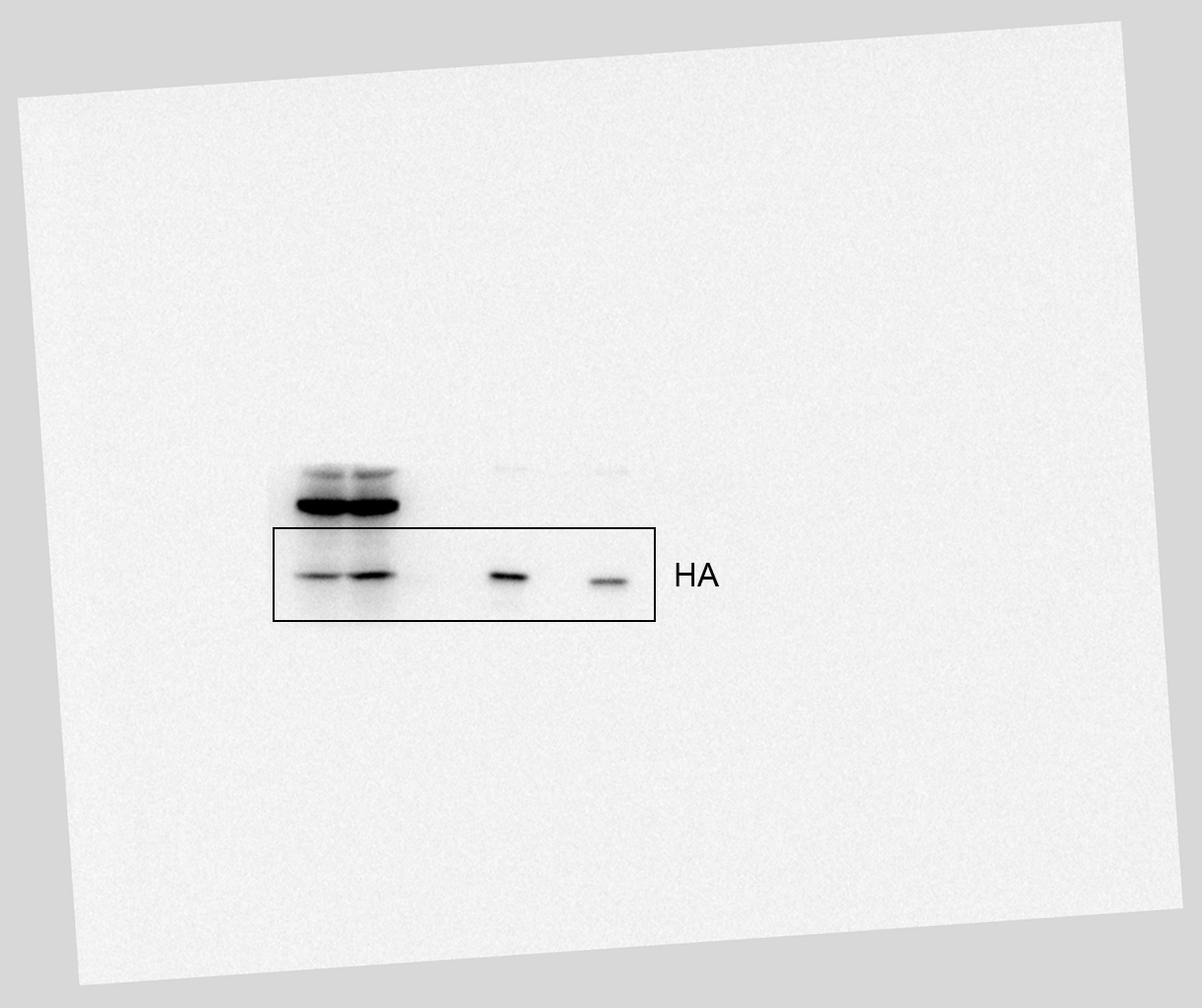

Supplement: Figure 7—figure supplement 1—source data 1. [file elife-95318-fig7-figsupp1-data1.zip › Figure7-figure supplement 1-Source data 1/Uncropped blots-Sup Fig6B/HA.tif]

**Sup Figure 6B**

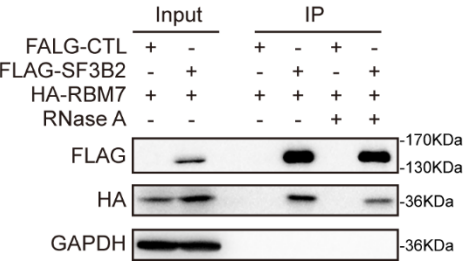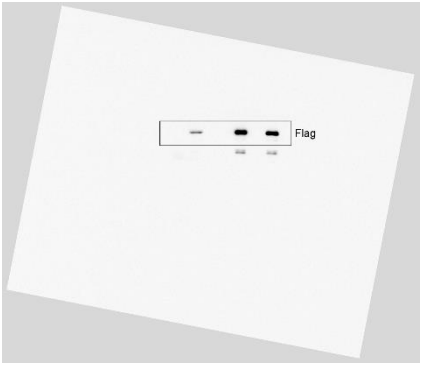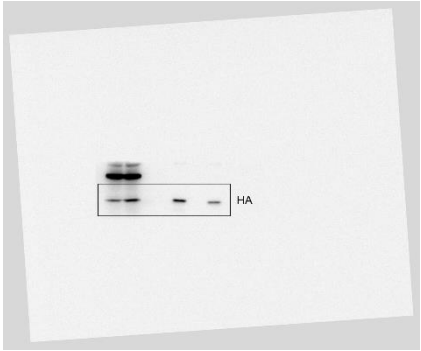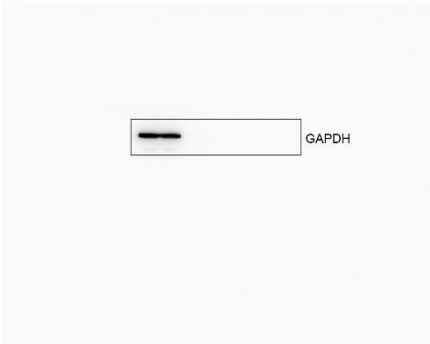

Supplement: Figure 7—figure supplement 1—source data 2. [file elife-95318-fig7-figsupp1-data2.zip › Figure7-figure supplement 1-Source data 2/Sup Fig6B.pdf]

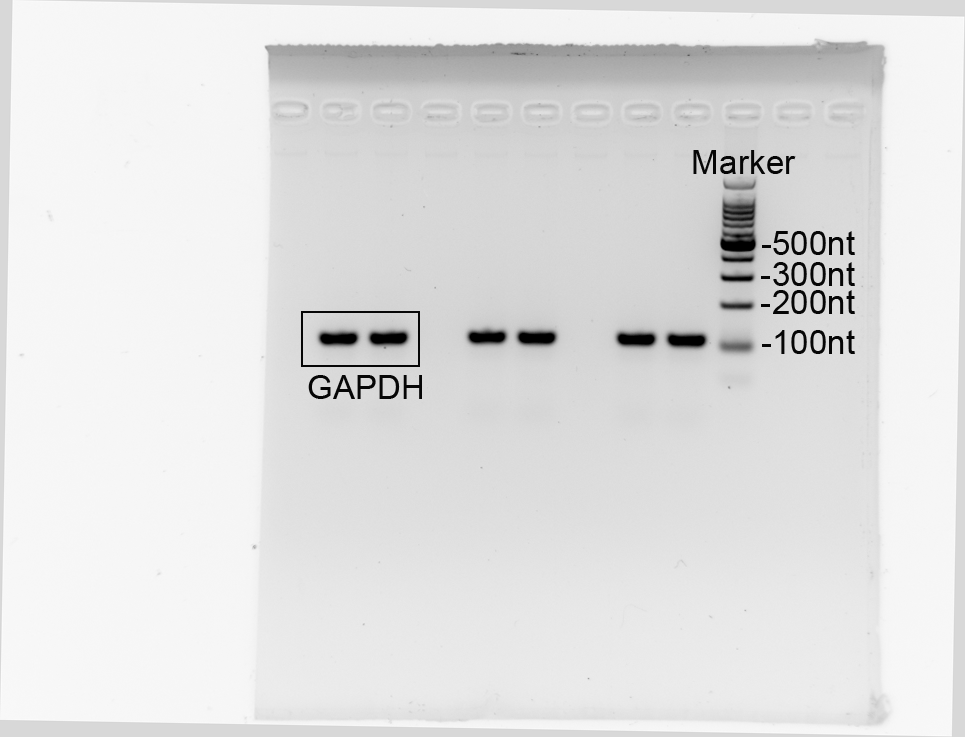

Supplement: Figure 7—figure supplement 1—source data 3. [file elife-95318-fig7-figsupp1-data3.zip › Figure7-figure supplement 1-Source data 3/Uncropped gels-Sup Fig6C/HCC1937 GAPDH.tif]

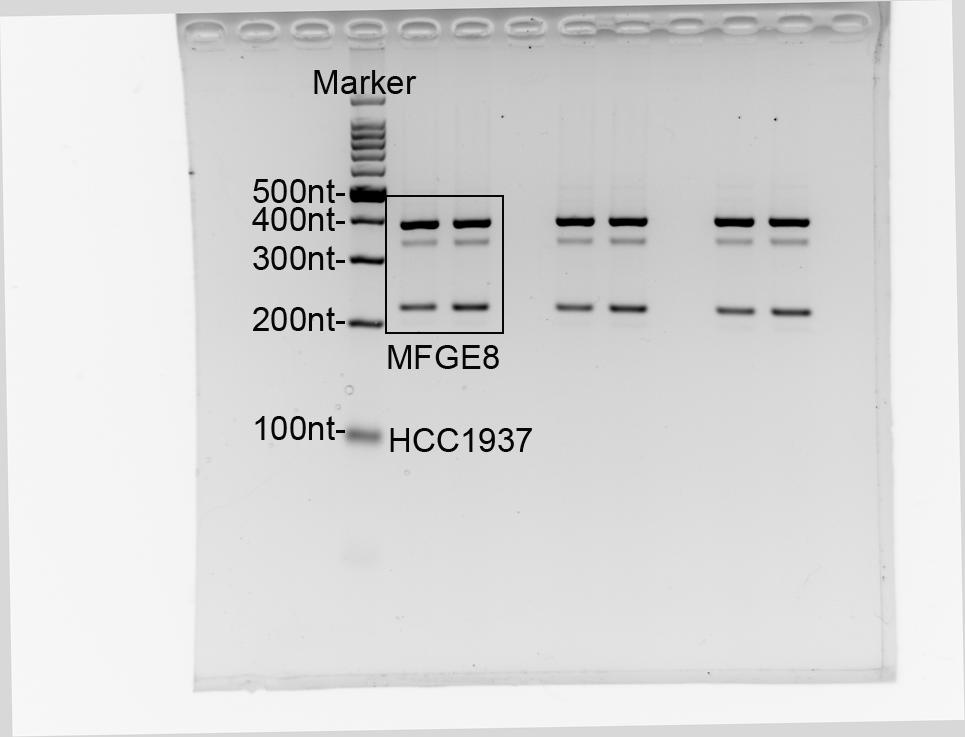

Supplement: Figure 7—figure supplement 1—source data 3. [file elife-95318-fig7-figsupp1-data3.zip › Figure7-figure supplement 1-Source data 3/Uncropped gels-Sup Fig6C/HCC1937 MFGE8.tif]

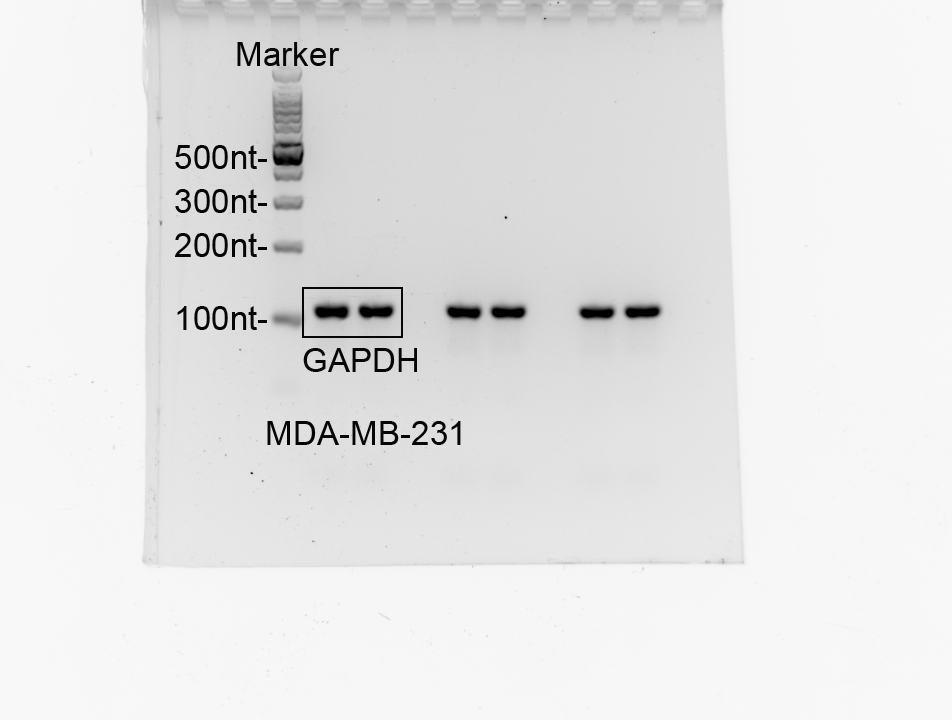

Supplement: Figure 7—figure supplement 1—source data 3. [file elife-95318-fig7-figsupp1-data3.zip › Figure7-figure supplement 1-Source data 3/Uncropped gels-Sup Fig6C/MDA-MB-231 GAPDH.tif]

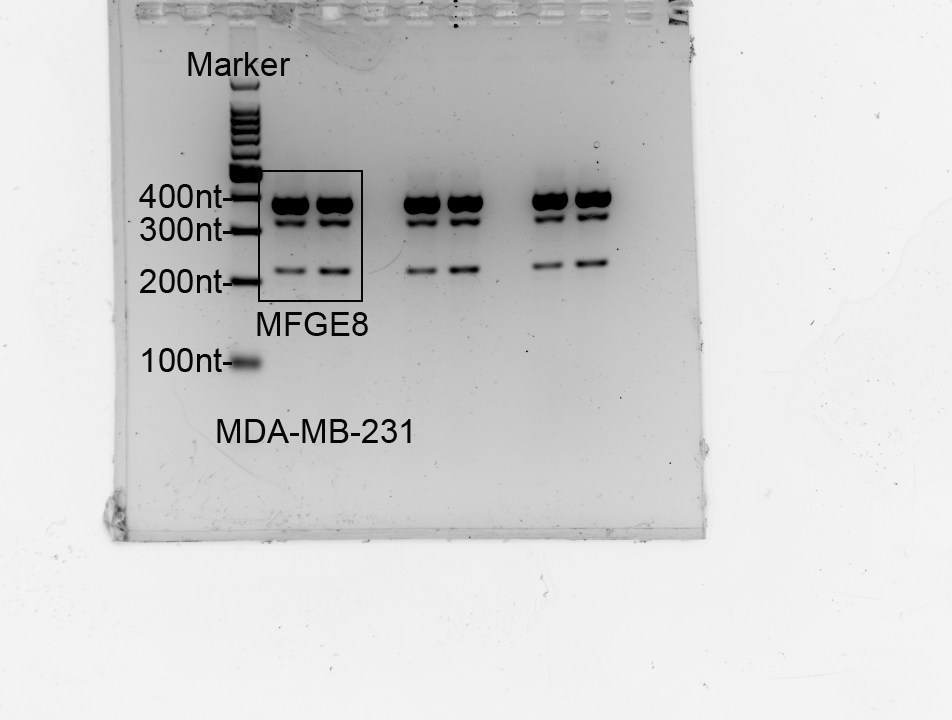

Supplement: Figure 7—figure supplement 1—source data 3. [file elife-95318-fig7-figsupp1-data3.zip › Figure7-figure supplement 1-Source data 3/Uncropped gels-Sup Fig6C/MDA-MB-231 MFGE8.tif]

## Sup Figure 6C

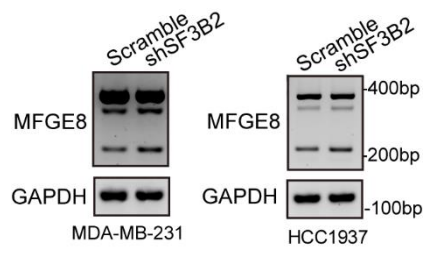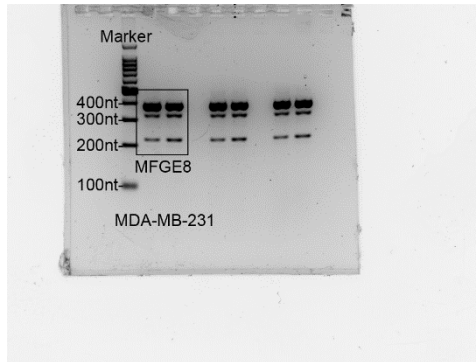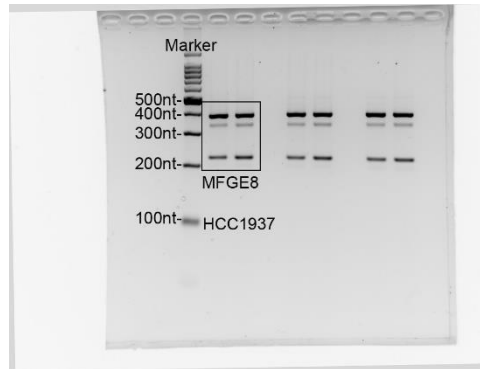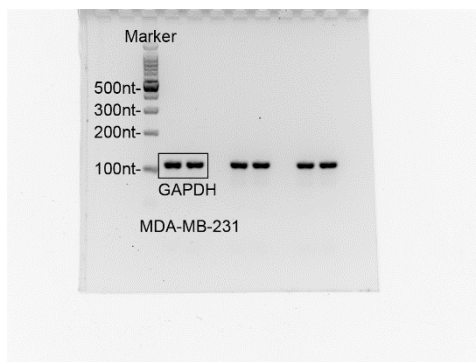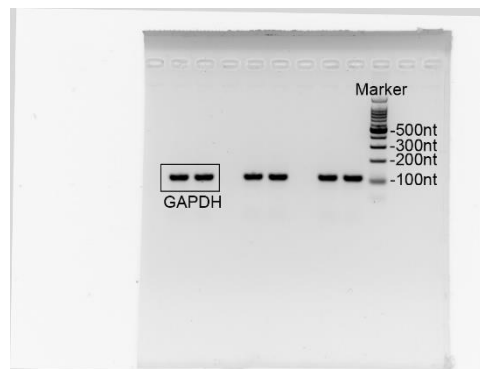

Supplement: Figure 7—figure supplement 1—source data 4. [file elife-95318-fig7-figsupp1-data4.zip › Figure7-figure supplement 1-Source data 4/Sup Figure6C.pdf]
