# Supplementary material for: RBM7 deficiency promotes breast cancer metastasis by coordinating MFGE8 splicing switch and NF-kB pathway
Source: eLife. 2024 Jul 12;13:RP95318. doi: 10.7554/eLife.95318 (PMC11245308; doi:10.7554/eLife.95318)
Supplement: Supplementary file 1. [file elife-95318-supp1.docx]

**Supplementary File 1**

| Primers | Gene | primer name | sequence |
| --- | --- | --- | --- |
| Primers for qRT-PCR | RBM7 | RBM7-qF | 5'-AAGCGGATCGCACTCTCTTT-3' |
|  |  | RBM7-qR | 5'-ACGCAAACTGCTTTGGTTTAC-3' |
|  | GAPDH | GAPDH-qF | 5'-ATGGGGAAGGTGAAGGTCG-3' |
|  |  | GAPDH-qR | 5'-GGGGTCATTGATGGCAACAATA-3' |
|  | CCL28 | CCL28-qF | 5'-AGAGCTGATGGGGATTGTG-3' |
|  |  | CCL28-qR | 5'-GGTGTTTCTTCCTGTGGCA-3' |
|  | BMP2 | BMP2-qF | 5'-TCGCAGGCACTCAGGTCAG-3' |
|  |  | BMP2-qR | 5'-TTCCCACTCGTTTCTGGTAGTT-3' |
|  | IL-1A | IL-1A-qF | 5'-GGTTGAGTTTAAGCCAATCCA-3' |
|  |  | IL-1A-qR | 5'-TGCTGACCTAGGCTTGATGA-3' |
|  | IL-1B | IL-1B-qF | 5'-GGGCCTCAAGGAAAAGAATC-3' |
|  |  | IL-1B-qR | 5'-TTCTGCTTGAGAGGTGCTGA-3' |
|  | LIF | LIF-qF | 5'-CGGGACCAGAAGATCCTCAA-3' |
|  |  | LIF-qR | 5'-ACAGCCCAGCTTCTTCTTCT-3' |
|  | EBI3 | EBI3-qF | 5'-TCATCAAGCCCGACCCTCC-3' |
|  |  | EBI3-qR | 5'-CCAGTCACTCAGTTCCCCG-3' |
|  | MMP9 | MMP9-qF | 5'-AACTTTGACAGCGACAAGAAGT-3' |
|  |  | MMP9-qR | 5'-ATTCACGTCGTCCTTATGCAAG-3' |
|  | ITGB3 | ITGB3-qF | 5'-GGGGTAGGTTGGGAGAATGT-3' |
|  |  | ITGB3-qR | 5'-TCTGGGACAAAGGCTAAGGA-3' |
|  | ZEB2 | ZEB2-qF | 5'-GCTGTTTCTTCGCTTCCACCT-3' |
|  |  | ZEB2-qR | 5'-ATTGATAAGAGCGGATCAGATGG-3' |
|  | EGFR | EGFR-qF | 5'-CTGGAGAAAGGAGAACGCC-3' |
|  |  | EGFR-qR | 5'-TTCATCCCCCTGAATGACA-3' |
|  | MMP24 | MMP24-qF | 5'-ATCTGCTTCCCTATGACTCACG-3' |
|  |  | MMP24-qR | 5'-CGCTTGTTTCTCCGCCTAC-3' |
|  | IL-6 | IL-6-qF | 5'-GAAAGCAGCAAAGAGGCAC-3' |
|  |  | IL-6-qR | 5'-GCTCTGGCTTGTTCCTCACTAC-3' |
|  | IL-8 | IL-8-qF | 5'-GTGCAGTTTTGCCAAGGAGT-3' |
|  |  | IL-8-qR | 5'-CTCTGCACCCAGTTTTCCTT-3' |
|  | mGAPDH | mGAPDH-qF | 5'-CCTTCCGTGTTCCTACCCC-3' |
|  |  | mGAPDH-qR | 5'-GCCCTCAGATGCCTGCTTC-3' |
|  | mRBM7 | mRBM7-qF | 5'-GTGTCTGTTCCCTATGCC-3' |
|  |  | mRBM7-qR | 5'-CACGTTACCCACTGTCCT-3' |
|  | MFGE8 | MFGE8-qF | 5'-TTCCCAAGAAGTGCGAGGAGATGT-3' |
|  |  | MFGE8-qR | 5'-ATGCTGCAAACCCAAGAAGGTCAC-3' |
|  | ZEB1 | ZEB1-qF | 5'-ATGGCGGATGGCCCCAGGTGTAAGC-3' |
|  |  | ZEB1-qR | 5'-GCTTACACCTGGGGCCATCCGCCAT-3' |
|  | TGFB1 | TGFB1-qF | 5'-AGTTGTGCGGCAGTGGTTGAG-3' |
|  |  | TGFB1-qR | 5'-GGCCATGAGAAGCAGGAAAGG-3' |
|  | SF3B2 | SF3B2-qF | 5'-CCCACTCCTACAGTTTTGCC-3' |
|  |  | SF3B2-qR | 5'-TCCAAAGCCTGGGGGATCTT-3' |
| Primers for alternative splicing | MFGE8 | MFGE8-SE-F | 5'-TGGGCCTGAA GAATAACA-3' |
|  |  | MFGE8-SE-R | 5'-CAAGTTCTTCTTGTGGGA-3' |
|  | AKT2 | AKT2-SE-F | 5'-GCGTGTCTTCACAGAGGAGC-3' |
|  |  | AKT2-SE-F | 5'-AGCCCAGCAAGCAGGGACT-3' |
|  | ZMYND8 | ZMYND8-SE-F | 5'-ACCCTTGACCTTTCTGGCT-3' |
|  |  | ZMYND8-SE-R | 5'-CCAGCCGAGACTCTTTCG-3' |
|  | MBNL1 | MBNL1-SE-F | 5'-TACCAACGTG GCAATTGC-3' |
|  |  | MBNL1-SE-R | 5'-TAATGGGGGAAGTACAGCTT-3' |
|  | MAP7D1 | MAP7D1-RI-F | 5'-ATCGCAGCCTGCAGCTGA-3' |
|  |  | MAP7D1-RI-R | 5'-TGCACGCTTCGGGTGACGCT-3' |
|  | HNRNPC | HNRNPC-A5E-F | 5'-GGCTTTGCCTTCGTTCAG-3' |
|  |  | HNRNPC-A5E-R | 5'-CATCCTATCATAATAGTCCCGTTG-3' |
